# Generation of membrane-permeable cyclic peptides inhibiting protein–protein interaction

**DOI:** 10.1038/s41589-026-02237-7

**Published:** 2026-06-01

**Authors:** Xinjian Ji, Lluc Farrera-Soler, Jiajun Li, Gontran Sangouard, Nathan De Sadeleer, Alexander L. Nielsen, Ganesh K. Mothukuri, Anne Zarda, Edward J. Will, Florence Pojer, Kelvin Lau, Christian Heinis

**Affiliations:** 1 Institute of Chemical Sciences and Engineering, School of Basic Sciences, École Polytechnique Fédérale de Lausanne (EPFL), Lausanne, Switzerland; 2 Protein Production and Structure Core Facility (PTPSP), School of Life Sciences, École Polytechnique Fédérale de Lausanne (EPFL), Lausanne, Switzerland

**Keywords:** High-throughput screening, Combinatorial libraries, Peptides, Drug delivery

## Abstract

Small, nonpolar cyclic peptides can both bind challenging targets and cross cell membranes, making them attractive for addressing currently undruggable targets such as many protein–protein interactions (PPIs). However, developing such compounds de novo without prior information about lead structures such as natural ligands or fragments remains a notable challenge. Here we show that functional screening of structurally highly diverse cyclic peptide libraries synthesized at nanomole scale allows identification of sub-kDa inhibitors of a PPI. By screening 15,360 fully random cyclic peptides, we were able to identify an inhibitor of the E3 ligase adaptor Keap1 and its substrate Nrf2. Optimization by rapid design–build–test cycles produced a membrane-permeable compound active in live cells. This study demonstrates that large, diverse cyclic peptide libraries can enable the discovery of cell-permeable PPI inhibitors from the ground up, providing a way to harness the powerful modality of small cyclic peptides to address often difficult-to-target intracellular interactions.

**Figure:**
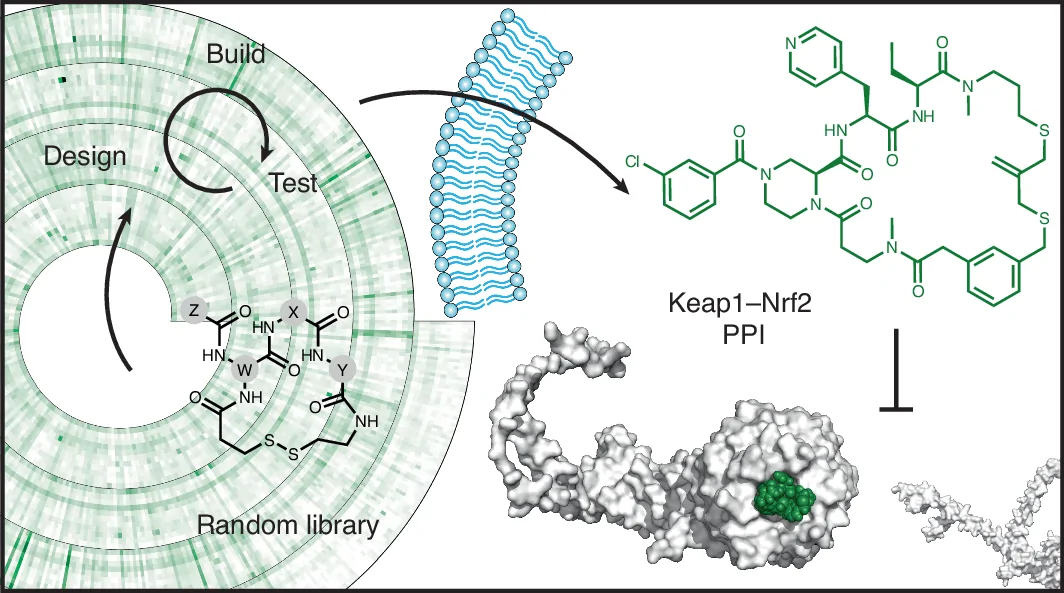

## Abstract (web summary)

Membrane-permeable cyclic peptides offer access to difficult intracellular targets but discovery remains challenging. Here the authors show that cell-active cyclic peptides can be identified by screening sufficiently large and diverse libraries of small synthetic peptides.

## Main

Small cyclic peptides that can passively traverse cell membranes offer great promise for modulating challenging intracellular targets that are often refractory to classical small molecules^1–4^. The flexibility-reducing ring-shaped structure of such peptides enables binding to difficult targets including potentially many protein–protein interactions (PPIs)^5–7^. While the development of cell-permeable and orally available drugs has long been guided by the rule of five (Ro5) that recommends a molecule size below 500 Da, cyclic peptides from nature have shown that substantially larger molecules, such as the prototype peptide cyclosporine (1,203 Da), can efficiently cross cell membranes^8–10^. Having a molecular size of 1 kDa or even more, these membrane-permeable peptides have a substantially larger number of atoms with which to form molecular contacts for target engagement, which greatly facilitates the development of ligands for challenging proteins. The new opportunities offered by cyclic peptides in the so-called ‘beyond Ro5’ space open up the exciting possibility that ligands can be developed for a wide range of challenging intracellular targets including a large number of PPIs implicated in currently untreatable diseases^11–14^.

The development of membrane-permeable cyclic peptides and macrocyclic compounds in general has relied heavily on existing ligands such as natural products, peptide-binding motifs or surface regions of protein interaction partners such as α-helices in PPIs, which may be transformed into membrane-permeable mimics using classical medicinal chemistry strategies^15–17^. However, for many targets, large ligands cannot be converted into membrane-permeable compounds and, for most targets, ligands or other prior knowledge suitable for ligand design is unavailable. Screening vast libraries of random molecules using genetically encoded libraries is a promising strategy for the de novo development of cell-permeable cyclic peptides^18–21^. Recently, great progress has been made with various such techniques including mRNA display of heavily *N*-methylated cyclic peptides that are membrane permeable^22,23^ or synthesis of highly pure DNA-encoded peptide libraries^24,25^. Our lab has advanced methods for high-throughput synthesis and screening cyclic peptide libraries at nanomole scale using solid-phase peptide synthesis (SPPS) in 384-well plates^26^ and high-throughput experimentation (HTE) for further library diversification by acoustic liquid transfer at nanomole scale in 1,536-microwell plates^27^. Screening target-tailored libraries of cyclic peptides using functional assays, we identified binders of several targets^27–29^. Summarizing the recent technological advances, there has been impressive progress with techniques such as mRNA display, DNA-encoded libraries and HTE but a broadly applicable method for de novo discovering membrane-permeable peptides targeting proteins has yet to be described.

An example of a challenging intracellular target is the PPI between the E3 ligase adaptor protein Keap1 and the transcription factor Nrf2 that maintains redox and metabolic homeostasis and is implicated in various diseases involving oxidative stress and inflammation, as well as neurodegenerative conditions and cancer^30^. This interaction has proven difficult to drug because the interaction involves a large surface (around 650 Å^2^) and is based on mostly electrostatic and polar contacts between the positively charged Kelch domain of Keap1 and a negatively charged peptide region of Nrf2 (DEETGEFL; negatively charged residues underlined). High-affinity small-molecule and peptide-based inhibitors of Keap1–Nrf2 reported to date contain at least one negative charge, which can reduce membrane permeability and pose problems because of type II metabolism^31–33^. The Keap1–Nrf2 structure and its PPI motif are shared by many key intracellular targets including other Kelch-domain proteins and WD40-repeat domains with β-propeller folds and peptide-binding pockets^34^, which makes this PPI an especially attractive target, as successful ligand development strategies may be transferable to a broader class of structurally related, disease-relevant proteins^12,35^.

Herein, we aimed at establishing a library screening strategy for developing membrane-permeable cyclic peptide ligands for targets from scratch and, thus, without information about existing ligands for library design. Toward this end, we built on recently developed HTE methods but aimed at generating larger and more diverse libraries to increase the chance of finding ligands. In addition, we propose to apply a fast-paced, iterative approach to library synthesis and screening, designed to optimize both target binding and membrane permeability in tandem. As our target, we selected the Keap1–Nrf2 PPI described above, for which no binding fragments were known other than negatively charged groups, which we aimed to avoid. Synthesis and screening of a library of around 15,000 random cyclic peptides led to the identification of hits that inhibited Keap1–Nrf2 and could be improved in three iterative rounds into a membrane-permeable cyclic peptide that blocked this PPI in live cells, demonstrating that cell-active cyclic peptides can be developed from scratch without the need for structural information from existing binders.

## Results

### Synthesis and screening of 15,360 random cyclic peptides

**Fig. 1:**
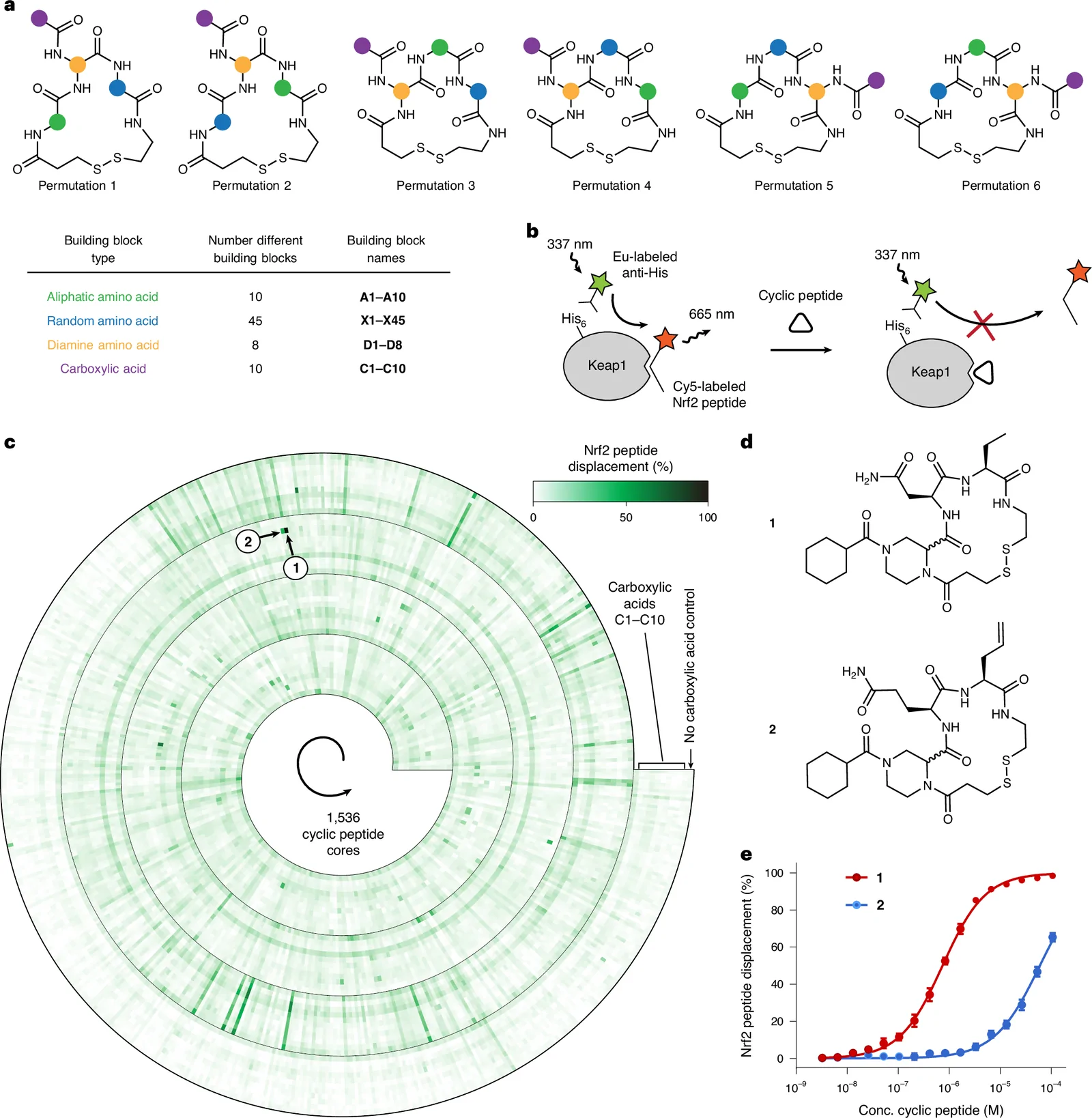
Synthesis and screening of a random sub-kDa cyclic peptide library. **a**, Format of library 1 compounds and building blocks. A total of 1,536 random cyclic peptides were combinatorially acylated with the 10 indicated carboxylic acids (purple) to generate 15,360 compounds. Carboxylic acids were conjugated to the diamino acids in peptides (yellow) by free amino groups. **b**, Schematic representation of the TR-FRET assay used in the HTS for PPI inhibitors of the Keap1 Kelch domain and a 9-aa region of Nrf2 (Cy5-labeled Nrf2 peptide). **c**, Result of HTS screen with library 1. Extent of Nrf2 peptide displacement from Keap1 is indicated as a percentage for each library member tested at a concentration of 25 μM. The 1,536 cyclic peptide cores are arranged on the *y* axis (spiral) with the 10 carboxylic acids on the *x* axis. Darker colors indicate higher PPI inhibition activity. **d**, Chemical structures of the cyclic peptide hits **1** and **2** indicated in **c**. **e**, Displacement of the Nrf2 peptide from the Kelch domain of Keap1 by nonpurified (crude) compounds **1** and **2** measured by TR-FRET assay. The indicated concentration is based on the cyclic peptide core and assumes quantitative acylation. The mean and s.d. values from three technical replicates are shown. Source data

**Extended Data Fig. 1:**
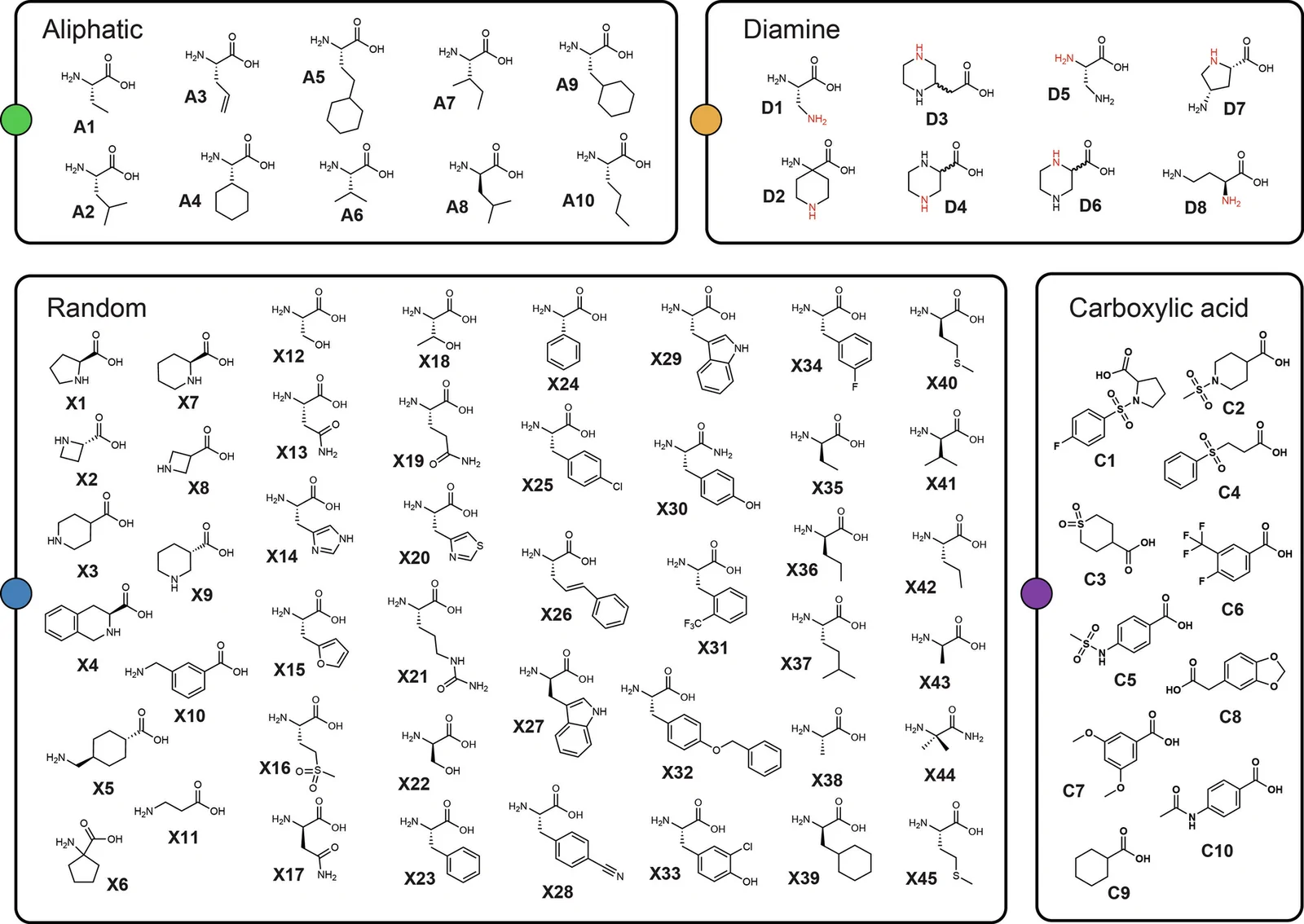
Building blocks used for the synthesis of Library 1. The format of the cyclic peptides is shown in Fig. 1a. Carboxylic acids (purple) were conjugated to the diamino acids (yellow) via free amino groups (highlighted in red). Source data

To identify lead structures as starting points for the development of intracellular PPI inhibitors, we designed and synthesized a large library of fully random cyclic peptides for high-throughput screening (HTS) (Fig. 1a). This library 1 is based on 1,536 disulfide-cyclized peptide cores each consisting of three random amino acids, chosen from a total of 63 chemically and structurally diverse amino acids (one of each group colored in green, yellow and blue in Fig. 1a and Extended Data Fig. 1). The cyclic peptide cores were combinatorially diversified by acylation of a peripheral amino group (red in Extended Data Fig. 1) with a carboxylic acid selected from ten random structures (colored in purple) (Supplementary Tables 1–4). The resulting 15,360 cyclic peptides have fully random compositions not tailored to a specific target. All library members are uncharged and lack highly polar groups and the majority have a molecular weight of 600–800 Da, all ideal characteristics for the development of membrane-permeable PPI inhibitors (Supplementary Fig. 1).

**Extended Data Fig. 2:**
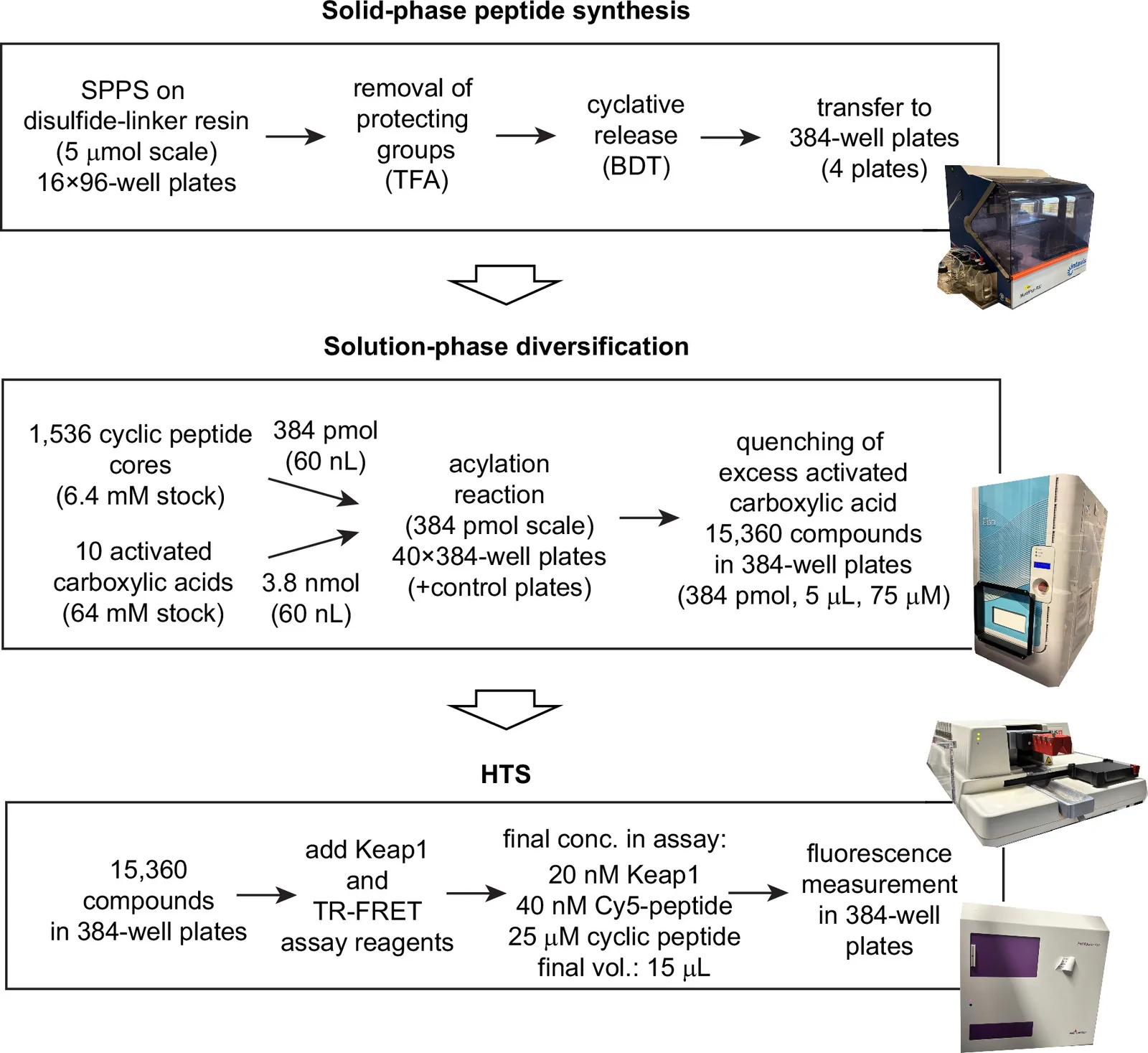
Workflow for the synthesis of Library 1 and HTS. Overview of the experimental steps, divided into SPPS, in solution diversification and activity screening. Microwell plate formats, scale and concentrations are indicated. Photos of key equipment used for the different steps are shown (parallel peptide synthesizer, acoustic dispenser, bulk dispenser and fluorescence plate reader). Fotos of the instruments were taken by the author C.H.

We produced library 1 in two steps, using recently established methods for high-throughput SPPS^26,36^ and combinatorial nanomole-scale diversification^27^ (Extended Data Fig. 2). In the SPPS step, linear peptides were synthesized on a disulfide linker solid support in microwell plates and released by a cyclative reaction to yield crude disulfide-cyclized peptides^36^. Synthesis in 16 96-well plates yielded millimolar peptide stocks (average concentration: 8.8 mM; Supplementary Fig. 2) with an average purity of >80%, as assessed for 48 randomly selected peptides (Supplementary Fig. 3 and Supplementary Table 5), which we considered sufficiently pure to allow omission of a time-consuming chromatographic purification step. The most frequent impurities were peptide dimers (average abundance: 7.6%) and peptide carrying a *tert*-butyl group (average abundance: 5.9%). We diversified the 1,536 cyclic peptide cores at the subnanomole scale (380 pmol) by combinatorial acylation with carboxylic acids in 384-well plates and using acoustic liquid transfer for reagent transfer (tenfold molar excess over peptide; purple in Fig. 1a and Extended Data Fig. 1). Target protein and assay reagents were added directly to the 384-well synthesis plates, resulting in 15-µl reaction volumes with final HTS compound concentrations of 25 µM.

For PPI inhibition screening, we established a time-resolved fluorescence resonance energy transfer (TR-FRET)-based assay in which the Kelch domain of human Keap1 bound to a europium-labeled antibody forms a complex with an Nrf2-based peptide labeled with Cy5 and inhibition of the interaction by a cyclic peptide is detected by a reduced FRET signal (Fig. 1b, Supplementary Fig. 4 and Supplementary Table 6). We identified optimal concentrations of Keap1 (20 nM), Nrf2 peptide (40 nM) and Eu-labeled anti-His (0.5 nM) that yielded a large dynamic range with a more than 30-fold reduction in FRET upon full PPI inhibition, all while using only moderate amounts of protein and probe per well (Supplementary Fig. 5). HTS of the 15,360 cyclic peptides of library 1 showed several compounds that reduced the FRET signal substantially (Fig. 1c and Supplementary Fig. 6), including the most active peptides **1** and **2** that lowered FRET by 95% and 48%, respectively. Comparison of these two hits revealed a striking structural similarity (Fig. 1d). Repetition of the acylation reactions for the two hits and TR-FRET assays at various dilutions of the crude products showed a clear dose response, with compound **1** (half-maximal inhibitory concentration (IC_50_) of approximately 1 μM) being around 50-fold more active than **2** (Fig. 1e).

### Identification of noncharged Keap1 ligand

**Fig. 2:**
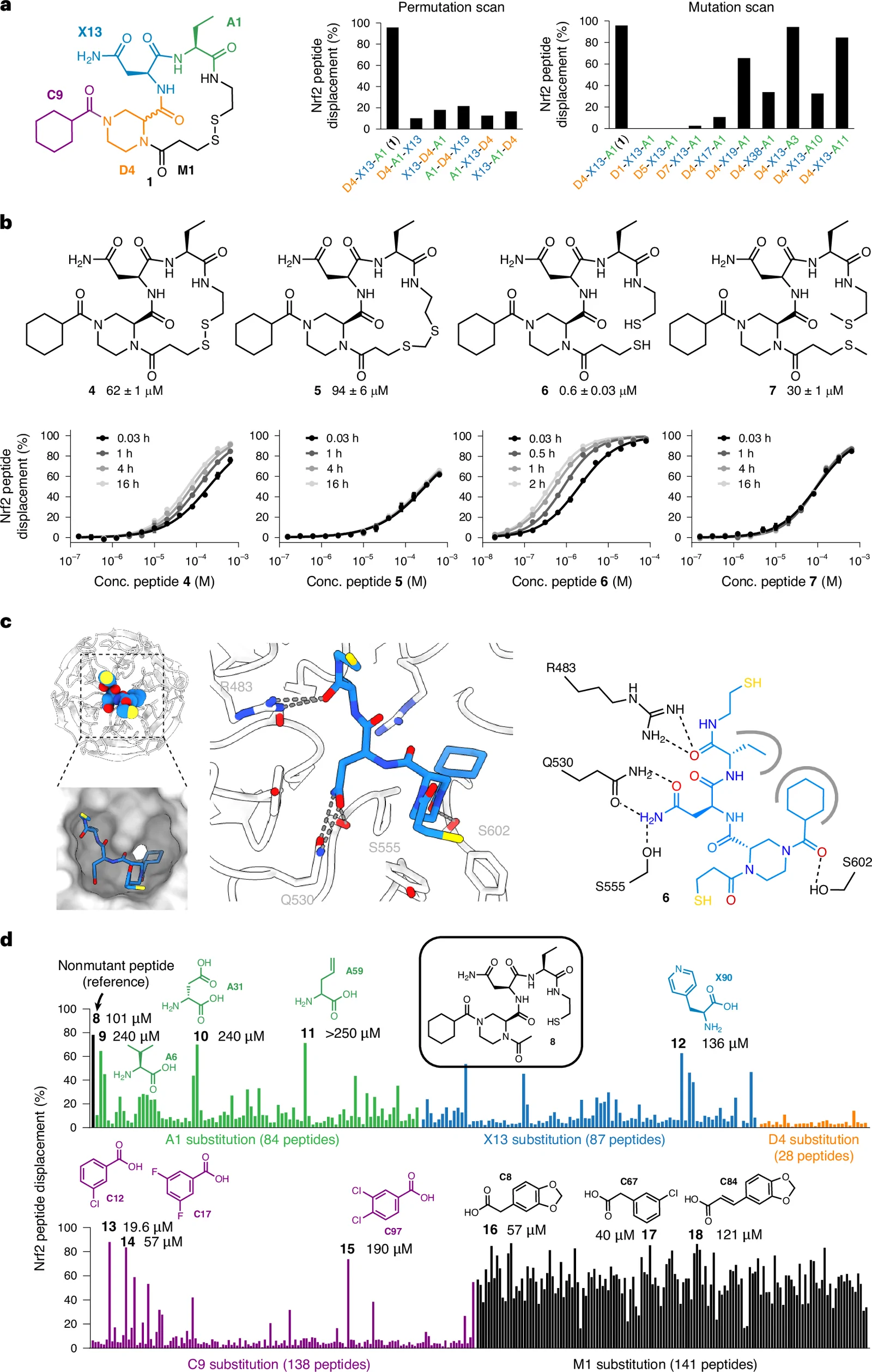
Structure and SAR analysis of peptide **1**. **a**, Chemical structure of cyclic peptide **1** and activity of all possible sequence permutations (left) and substitutions of amino acids D4, X13 and A1 (three substitutions for each; right). Amino acid structures are shown in Extended Data Fig. 1. Activity of crude products was measured by TR-FRET. **b**, Comparing the activity of redox-labile (disulfide bridges or free sulfhydryl groups) and redox-stable peptides (thioether-cyclized or methyl-capped) and of linear and cyclic variants. All peptides were HPLC purified. Single measurements at different time points are shown. Indicated *K*_i_ values are the mean and s.d. values from three technical replicates. **c**, X-ray structure of linear peptide **6** bound to the Kelch domain of Keap1 at 1.70-Å resolution (PDB 9IH9). H-bond interactions are shown in the zoomed-in view. **d**, SAR study of peptide **4**, with peptide variants synthesized as linear peptides. The structure of reference peptide **8** is shown. One building block was altered at a time and the PPI inhibition activity of the 478 crude peptides (library 2) was measured by TR-FRET. Source data

**Extended Data Fig. 3:**
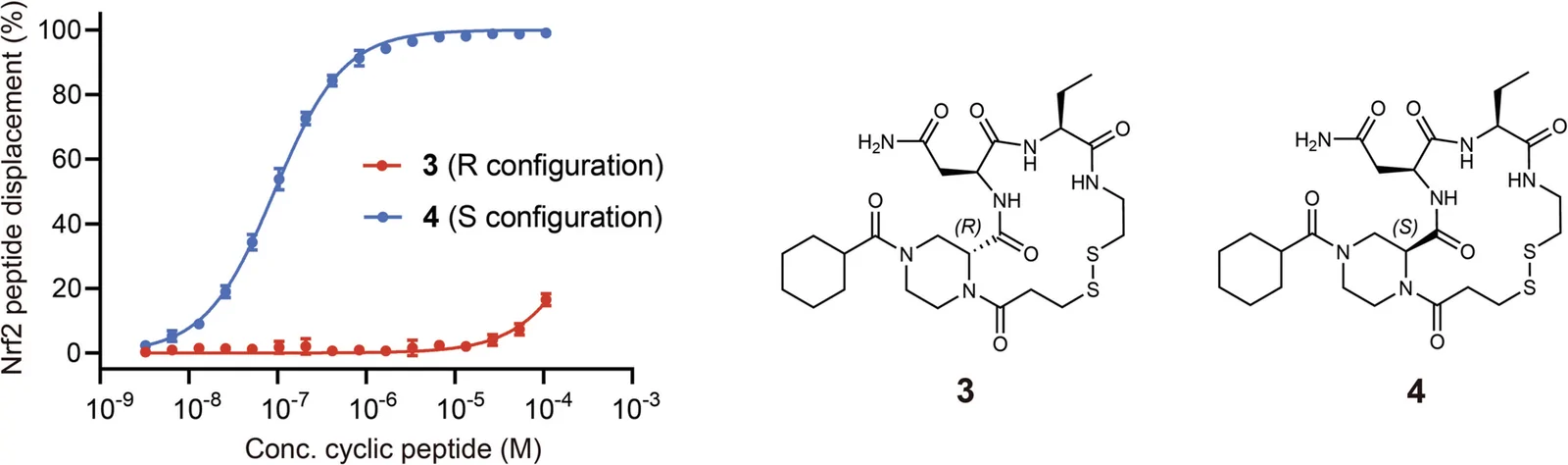
Diastereoisomers of peptide 1. Identification of active epimer of cyclic peptide **1** by synthesizing cyclic peptides **3** and **4** using enantiopure D4 amino acids having either *R* (for peptide **3**) or *S* configuration (for peptide **4**). Activities were measured using crude peptide preparations. Average values and SDs of three technical replicates are shown.

To assess whether the observed PPI inhibition activity was derived from the desired cyclic peptide or from reaction side-products that are present because of screening with nonpurified compounds, we repeated the acylation reactions for hits **1** and **2** on a ~100-fold larger scale than in library 1 synthesis (64 nmol), generating chromatographically separated products and side-products. Measurements showed activity for the desired cyclic peptide products but even higher activity was observed for byproducts that were identified as a mixture of cyclic peptide dimer and trimer (Supplementary Fig. 7). This result prompted us to further investigate whether the observed activity was driven by binding from the specific building block composition and sequence or merely resulted from nonspecific interactions caused by dimers and trimers that are twofold and threefold larger and, thus, potentially able to make many more contacts. To this end, we synthesized variants of the more active cyclic peptide **1** by scrambling the amino acid sequence, testing all possible permutations, showing that only the exact sequence identified by HTS displayed notable probe displacement (>90%; Fig. 2a). A rapid substitution scan showed that all amino acids contributed to the binding (Fig. 2a). For the building block D4, a racemate of D/L-Fmoc amino acids was used in the library preparation, meaning that the cyclic peptide **1** was a mixture of diastereomers. Synthesis of the cyclic peptides **3** and **4** having defined stereocenters in D4 showed that only the peptide with *S* configuration was active (peptide **4**), further supporting a specific interaction (Extended Data Fig. 3).

Characterization of peptide **4** after high-performance liquid chromatography (HPLC) purification showed a substantially weaker activity compared to unpurified product (*K*_i_ = 62 ± 1 μM), which we attributed to the removal of multimeric side-products that dominated the activity in crude product, as described above. Measurement of TR-FRET after prolonged incubation times showed an activity increase (up to threefold; Fig. 2b and Supplementary Fig. 8), which we speculated could have resulted from disulfide exchange reactions of peptide **4** such as multimerization or even disulfide bridging to a surface cysteine of Keap1. Thus, we synthesized and tested a variant of **4** in which the thiol groups were connected by a methylene group that could not undergo disulfide exchange (peptide **5**; Fig. 2b). This variant inhibited the PPI with a *K*_i_ value of 94 ± 6 μM and did not change its activity at prolonged incubation times. To study the contribution of macrocyclization to the activity, we prepared two linearized analogs, with peptide **6** being the reduced form of the disulfide-cyclized peptide **4** and peptide **7** having thiol groups that were methyl-capped so that they could not undergo disulfide exchange (Fig. 2b). Peptide **6** displayed an initial *K*_i_ of 0.6 ± 0.03 μM that improved up to fivefold at extended incubation times, again likely because of disulfide exchange reactions. The redox-stable linear peptide **7** had a *K*_i_ of 30 ± 1 μM that remained unchanged at longer incubation times (Fig. 2b). These results together showed that peptide with a free thiol or disulfide bridge bind best, whereas redox-stable cyclic and linear peptides bind with weaker affinity. Importantly, we identified a relatively small peptidic structure that inhibits the PPI with micromolar affinity through defined molecular contacts. The peptide contained no charges and had a limited polar surface area, providing a good starting point for developing a membrane-permeable PPI inhibitor.

### Structure–activity relationship and X-ray structure reveal site and mode of binding

To study the binding site and molecular interactions in detail, we cocrystallized peptide **4** with the recombinant Keap1 Kelch domain containing two amino acid substitutions conducive to crystallization^37^ (Supplementary Fig. 9). Because of the presence of reducing agent DTT in conditions that yielded diffraction-quality crystals, we obtained a structure of the bound linear analog (**6**) rather than the cyclic peptide (PDB 9IH9, 1.70-Å resolution) (Fig. 2c and Supplementary Table 7). Attempts to solve the structures of cyclic peptide **4** or peptide **5** with nonreducible thioether bonds were not successful (Supplementary Table 8). The linear peptide **6** occupied the same space as the region of Nrf2 that interacts with Keap1 but formed completely different molecular contacts. The electron density map of peptide **6** showed that the four building blocks identified by HTS were well defined (Supplementary Fig. 10) and that backbone and side-chain groups formed defined contacts, including six hydrogen bonds (H-bonds) and two hydrophobic contacts (Supplementary Table 9). Unlike Nrf2, peptide **6** did not rely on charged interactions and used mostly H-bond acceptor groups for H-bonding interactions, offering an ideal scaffold for developing membrane-permeable PPI inhibitors. The thiol groups of the linear peptide **6** were relatively distant from each other (~11.4 Å) and these terminal regions were, thus, expected to adapt different conformations in cyclic peptides **4** and **5**. A computational modeling of cyclic peptides **4** and **5** on the basis of **6** suggested that the thiol ends can be connected by a short linker without disrupting the binding face (Supplementary Fig. 11).

We complemented the structural analysis with a comprehensive structure–activity relationship (SAR) analysis of peptide **4** to further study the importance of each chemical group in the ligand and to potentially identify structural changes that improve the binding. To avoid conformational constraints imposed by macrocyclization, we synthesized linear peptide variants, using peptide **8** as a benchmark for activity comparison (Fig. 2d, Supplementary Fig. 12 and Supplementary Table 10). Altering one building block at a time we synthesized a library of 478 peptide variants (library 2), using again the rapid synthesis on disulfide linker solid support but this time cleaving the peptide by reduction and not cyclization, yielding linear peptides with a sulfhydryl group at the C terminus^38^. For the four building block positions randomized in the initial library 1, most substitutions abolished binding completely. For the diamino acid substitutions (yellow), even all peptide variants were inactive, indicating a particular importance for this building block. Overall, the SAR findings closely mirrored the key interaction points observed in the X-ray structure, reinforcing the importance of the original contact map.

To validate and refine our SAR findings, we measured the binding affinities of the ten most active peptide variants **9**–**18** (chemical structures of new building blocks in Fig. 2d) using a fluorescence polarization (FP) assay. This assay quantified the displacement of a fluorescein-labeled peptide (fluorescein-LDEETGEFL-NH_2_, *K*_d_ = 18 nM) bound to the Keap1 Kelch domain (~85% complexed). We opted for FP over TR-FRET because the assay was compatible with the reducing agent that we added to prevent disulfide exchange reactions. Among the peptide variants **9**–**18** that showed similar or better *K*_i_ values compared to reference peptide **8** (crude product *K*_i_ = 101 ± 3 µM), peptide **13** stood out because of a ~5-fold increase in activity (crude *K*_i_ = 19.6 ± 0.5 µM). Because of its enhanced potency, we carried this substitution forward in all subsequent designs (Fig. 2d and Supplementary Fig. 12).

### Cysteine near to binding site lends itself to covalent inhibition

**Fig. 3:**
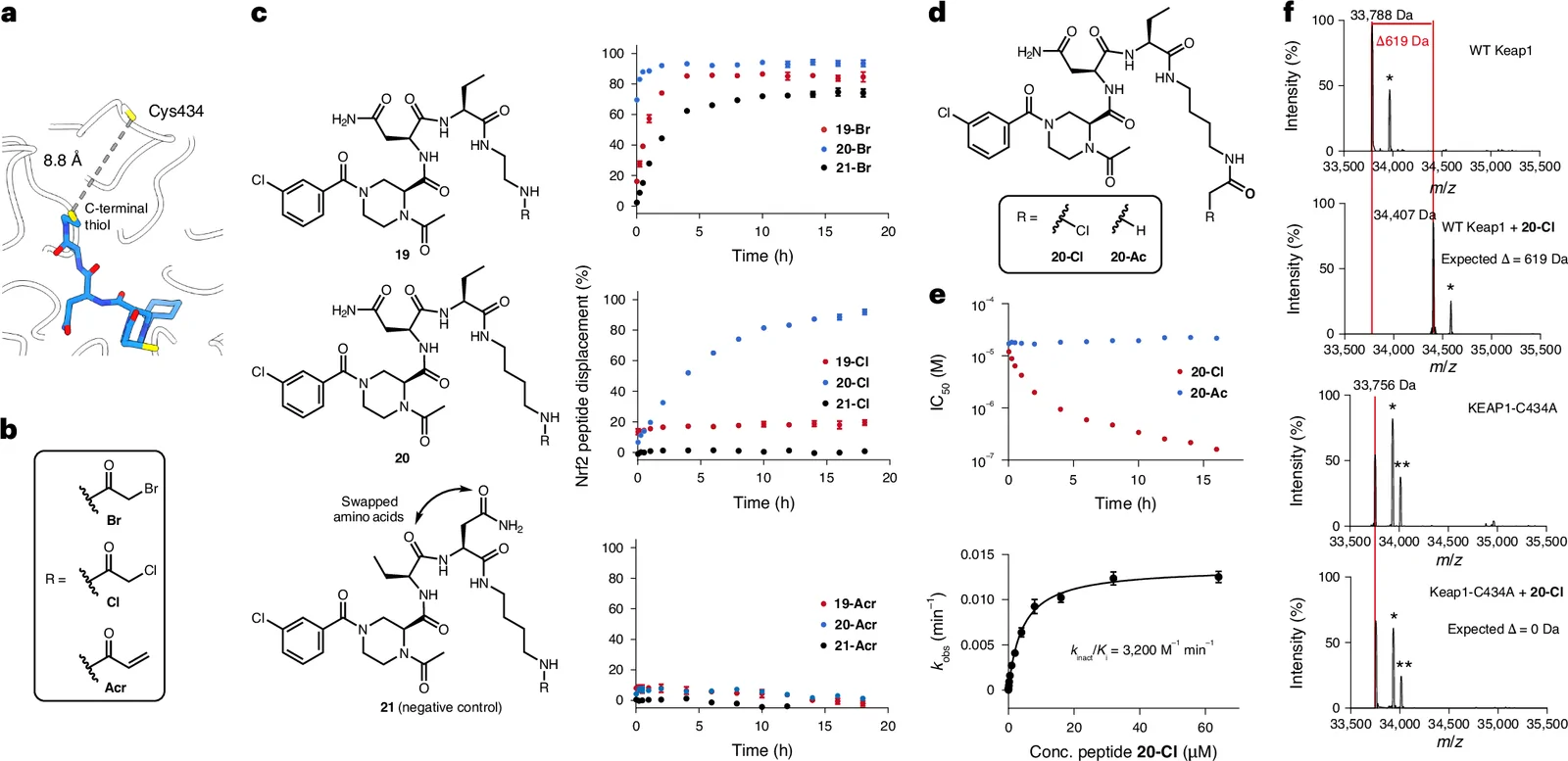
Structure-guided covalent PPI inhibitor development. **a**, Distance between C-terminal thiol of **6** and C434 of Keap1, indicated in the X-ray structure (PDB 9IH9). **b**, Chemical structures of applied thiol-reactive warheads. **c**, Peptides designed for covalent inhibition based on linear peptide **13** of SAR study. The three warheads in **b** were conjugated by a short (peptide **19**) or long (peptide **20**) linker. Inactive peptide **21** served as a negative control. Activities of 9 purified covalent peptides (1 μM) were assessed in the FP competition assay, measuring Nrf2 peptide displacement at time points ranging from 2 min to 16 h. The mean and s.d. values of three technical replicates are shown. **d**, Chemical structures of the selected covalent inhibitor **20-Cl** and its warhead-free analog **20-Ac**. **e**, Top: PPI IC_50_ of peptide **20-Cl** determined after different incubation times (Supplementary Fig. 14). Warhead-free **20-Ac** was included as control. The mean values of three technical replicates are shown. Bottom: the covalent efficiency constant (*k*_inact_/*K*_i_) of **20-Cl** was determined by measuring the *k*_obs_ at different peptide concentrations (Supplementary Fig. 15). The mean and s.d. values of three technical replicates are shown. **f**, Intact MS analysis of Keap1 Kelch domain (top two) and Keap1-C434A Kelch (bottom two) before and after 14 h of incubation with **20-Cl** at room temperature, showing a mass shift consistent with **20-Cl** adduct formation. Source data

Given the potential covalent inhibition mechanism described above and the proximity of C434 (~9 Å from the C-terminal thiol of the peptides; Fig. 3a), we hypothesized that our peptide binders could serve as scaffolds for covalent inhibitor design using thiol-reactive warheads. To test this, we retained the protein-binding core of peptide **13** and appended amino-functionalized linkers of varying lengths at the C terminus to strategically position electrophilic warheads such as bromoacetamide, chloroacetamide and acrylamide for optimal reactivity (Fig. 3b,c and Supplementary Fig. 13). Among the tested constructs, peptide **20-Cl**, bearing a chloroacetamide warhead on a longer linker, emerged as the most efficient and selective covalent inhibitor of the Keap1–Nrf2 PPI (*k*_inact_/*K*_i_ of 3,200 M^−1^ min^−1^). A direct comparison to its nonreactive analog, **20-Ac** (in which the chloroacetamide was replaced by an unreactive acetyl group), revealed a substantial contribution of the covalent mechanism to its inhibitory effect (Fig. 3d,e). Mass spectrometry (MS) analysis of intact protein using wild-type Keap1 Kelch domain and the C434A mutant control confirmed C434 as the site of covalent modification by **20-Cl** (Fig. 3f and Supplementary Fig. 16), providing direct evidence for the covalent engagement. Taken together, these findings demonstrate the feasibility of converting our noncovalent peptide leads into covalent inhibitors, although further rounds of medicinal chemistry will likely be required to optimize potency and to achieve a *k*_inact_/*K*_i_ value on the order of 10^4 ^M^−1^ s^−1^ that is typically needed for covalent drugs^39^.

### Screening 504 different linkers for macrocyclization

**Fig. 4:**
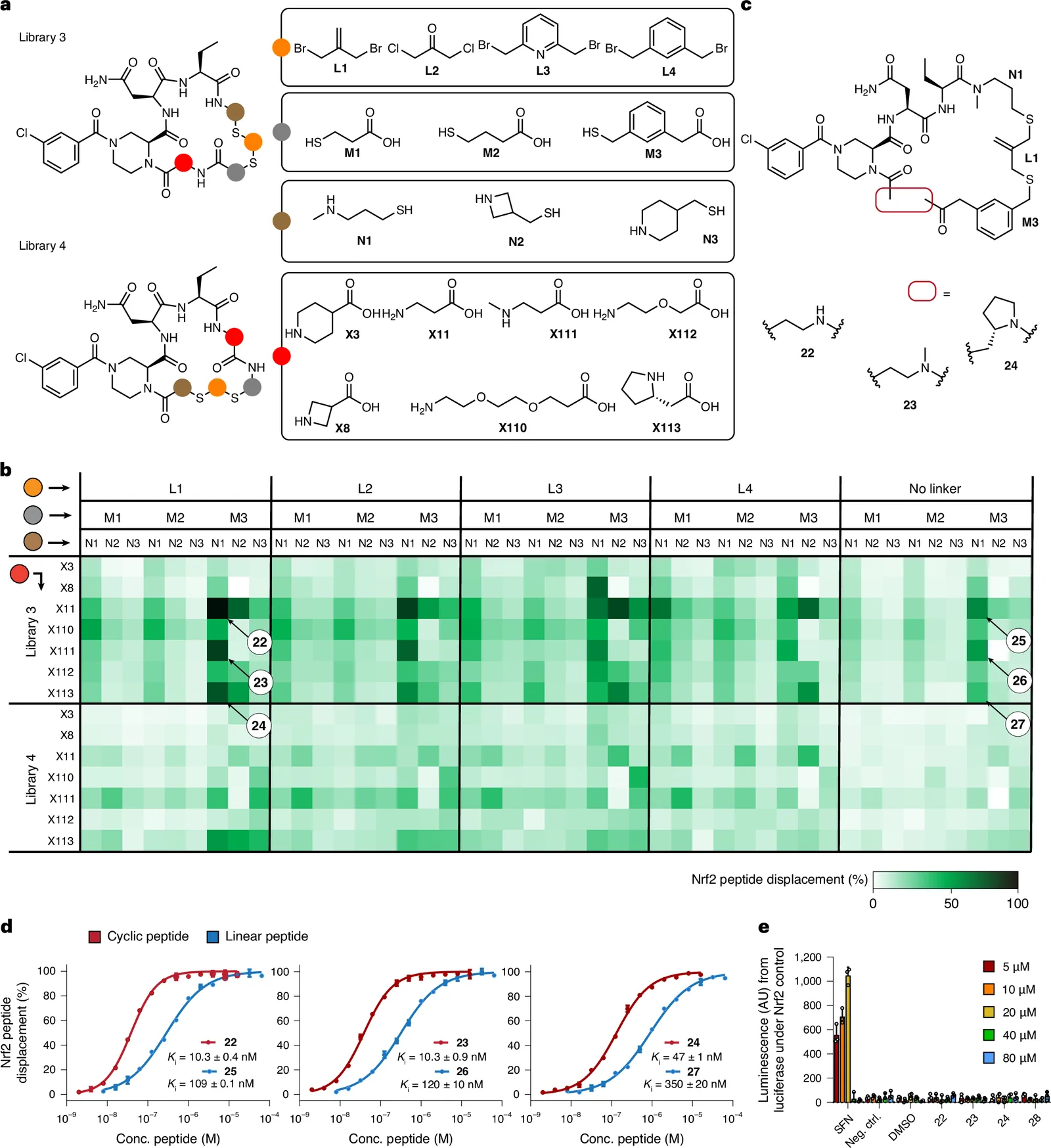
Macrocycle linker optimization. **a**, Library 3 and 4 formats designed on the basis of peptide **13** and generation of all possible 252 combinations with the indicated building blocks. **b**, PPI inhibition activity measured at a cyclic peptide concentration of 1 μM using a FP competition assay. **c**, Chemical structures of hit compounds **22**–**24** indicated in **b**. **d**, Activity of purified cyclic peptides **22**–**24** (red) and comparison to linear analogs **25**–**27** (blue). The mean and s.d. values from three technical replicates are shown. **e**, Cellular activity of cyclic peptides **22**, **23**, **24** and **28** in HEK293-ARE luciferase reporter cells. The nonspecific inhibitor SFN was used as a positive control. Inactive peptides and DMSO served as negative controls (neg. ctrl.). The mean and s.d. values from three technical replicates are shown. Source data

Turning our attention again to noncovalent PPI inhibition, we next aimed to optimize the macrocyclic backbone of the cyclic lead to improve binding affinity. To enable an optimal conformation for target binding and to form additional molecular contacts, we explored a wide chemical space of alternative linkers for connecting the termini of the ligand core structure of peptide **13** (Fig. 4a). We designed two focused libraries in which an additional random amino acid was added to the N terminus (library 3) or the C terminus (library 4) of the constant binding motif (building block X in Fig. 4a) and carried out another round of SPPS followed by HTS of crude products. All library peptides were designed with two thiol-containing building blocks (M and N; Fig. 4a) for cyclization with bis-electrophile reagents (L; Fig. 4a). We generated 252 different macrocyclic peptides per library and screened them with an FP competition assay under reducing conditions to exclude covalent binding of potential byproducts. Despite the lower screening concentration (1 µM versus 25 µM in the initial HTS), several peptide–linker combinations exhibited strong inhibition of the Keap1–Nrf2 PPI (Fig. 4b).

To maximize the chances of membrane permeability, we focused on peptides cyclized using the smallest and least polar linker L1 (peptides **22**–**24**) and compared them to their linear counterparts (peptides **25**–**27**; Fig. 4c and Supplementary Fig. 17). The purified macrocyclic peptides demonstrated potent binding, with the best ones having *K*_i_ values of 10 nM, representing a ~1,000-fold improvement over the lead peptide **13** and being ~10-fold better than the corresponding linear analogs (Fig. 4c). We tested the PPI inhibitors **22**–**24** and a variant of **23** with a covalent warhead (**28**; Supplementary Fig. 18) in a cellular assay in which Keap1–Nrf2 inhibition results in reduced Keap1-mediated Nrf2 degradation. However, none of the inhibitors showed any activity in this assay, which we attributed to limited membrane permeability, highlighting a key area for further optimization (Fig. 4e).

### Synthesis of 252 compounds to enhance membrane permeability

**Fig. 5:**
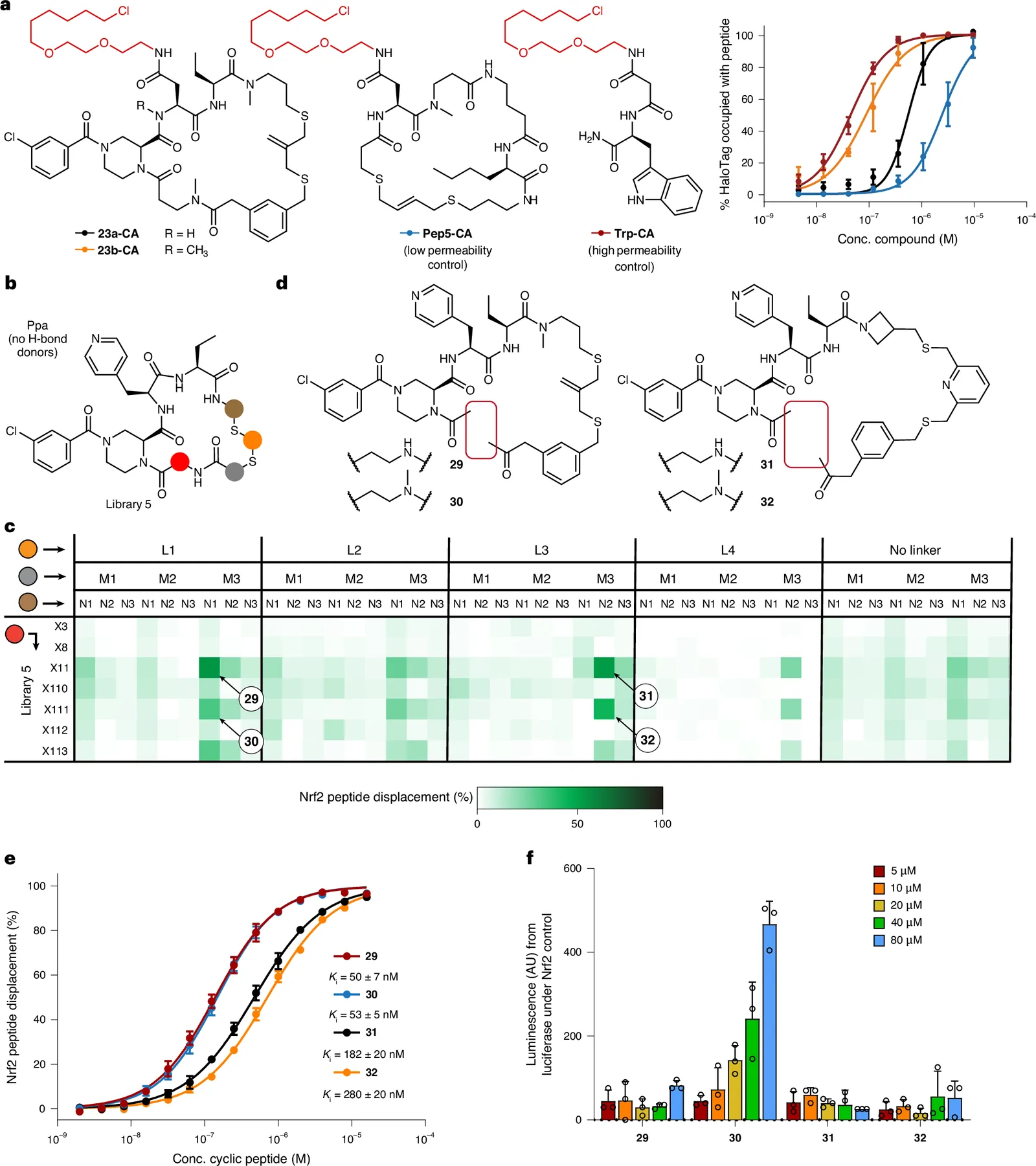
Membrane permeability study and engineering. **a**, Quantification of cyclic peptide reaching the cytosolic space of live HeLa cells using the CAPA assay. Chemical structures of cyclic peptides and assay controls containing a CA tag (red). The extent of HaloTag labeling (as a percentage of total HaloTag) after exposure of cells to the compounds for 4 h is shown. The mean and s.d. values of three experiments performed on different days are shown. Here **23b-CA** is a methylated analog that has one fewer H-bond donor because of *N*-methylation of the asparagine backbone amide bond. Although this asparagine modification eliminated Keap1 binding, it allowed us to test the impact of reduced polarity on permeability. **b**, Format of library 5 based on H-bond donor-free pyridine amino acid (Ppa) and building blocks shown in Fig. 4a. **c**, Activity of 252 cyclic peptides of library 5 screened at 1 μM. Extent of Nrf2 peptide displacement, measured by FP, is shown. **d**, Chemical structures of selected hits **29**–**32** indicated in **c**. **e**, Activity of purified cyclic peptides **29**–**32** determined with FP assay. The mean and s.d. values of three technical replicates are shown. **f**, Cellular activity of cyclic peptides **29**–**32** in HEK293-ARE luciferase reporter cells. The individual, mean (bars) and s.d. values are shown for three technical replicates measured on the same plate. Data from a replicate experiment performed by a different person are shown in Supplementary Fig. 20. Source data

To assess membrane permeability independently of activity and stability, we used the chloroalkane (CA) penetration assay (CAPA)^40,41^ that allows quantification of membrane permeability in live cells. In this assay, CA-tagged molecules that reach the cytosol covalently bind to a HaloTag enzyme anchored on the cytosolic face of cells. Residual unbound HaloTag is then labeled with a fluorescent, cell-permeable CA-chase probe and membrane permeability is quantified by flow cytometry. The key metric, CP_50_, represents the concentration of CA-tagged compound needed to occupy half of the cytosolic HaloTag. To preserve the structure of peptide **23**, we attached the CA tag to the side chain of its asparagine residue, creating **23a-CA** (Fig. 5a and Supplementary Fig. 19). The peptide exhibited moderate membrane permeability (CP_50_ = 0.58 ± 0.04 μM) but crossed the membrane much better than the low-permeability control **Pep5-CA** (CP_50_ = 2.6 ± 0.2 μM), which has the same ring size but five H-bond donors that impair membrane permeability. However, it was still far less permeable than the high-permeability small-molecule **Trp-CA** control (CP_50_ = 0.046 ± 0.002 μM). A less polar analog of **23a-CA** in which we *N*-methylated asparagine (**23b-CA**) showed excellent permeability (CP_50_ = 0.091 ± 0.009 μM), nearly matching **Trp-CA**. These results suggested that peptide **23** was already close to achieving efficient cell penetration and that fine-tuning its polarity could unlock its intracellular activity (Fig. 5a).

Building on our SAR data, we hypothesized that swapping the polar asparagine side chain in peptide **23** for a pyridine, an exchange that previously led to only a modest 1.5-fold drop in binding affinity for linear peptides, could enhance membrane permeability. To test this, we designed and rapidly synthesized library 5, comprising 252 cyclic peptides that all incorporated the pyridine-containing amino acid X90 (Fig. 5b). Screening the library with the FP assay at a compound concentration of 1 µM identified essentially the same building blocks in the linker region as in the library 3 screen (Fig. 5c). We selected four promising peptides **29**–**32** (Fig. 5d) that displayed *K*_i_ values ranging from 50–280 nM and, thus, were only slightly weaker than peptide **23** (Fig. 5e). Excitingly, peptide **30**, which displayed the best combination of activity (*K*_i_ = 53 ± 5 nM) and polar surface (only two H-bond donors) exhibited a robust, dose-dependent activity in the cellular model with an IC_50_ of around 50 μM (Fig. 5f and Supplementary Fig. 20). The lower activity of peptide **30** in cells compared to the in vitro assays prompted us to further study its membrane permeability. In the parallel artificial membrane permeation assay (PAMPA), peptide **30** (without CA tag) displayed a decent passive membrane permeability *P*_app_ of 1.2 × 10^−6 ^cm s^−1^ (Supplementary Fig. 21a). In assays measuring membrane permeability across cultured Caco-2 cell monolayers, peptide **30** had a permeability below the detection limit in the apical-to-basal direction (*P*_app_ = 0.07 × 10^−6 ^cm s^−1^ detection limit) but good permeability in the opposite direction (*P*_app_ = 6.6 × 10^−6 ^cm s^−1^) (Supplementary Fig. 21b). This indicated removal of **30** by efflux pumps (efflux ratio > 90), which could explain the lower activity found in the cellular assay compared to in vitro inhibition. Taken together, these results showed that efflux pumps may reduce levels in live cells but that the membrane permeability was efficient enough to achieve cellular activity. This makes peptide **30** a fully de novo developed cyclic peptide that successfully inhibits a PPI inside living cells.

### X-ray structure reveals key molecular interaction types

**Fig. 6:**
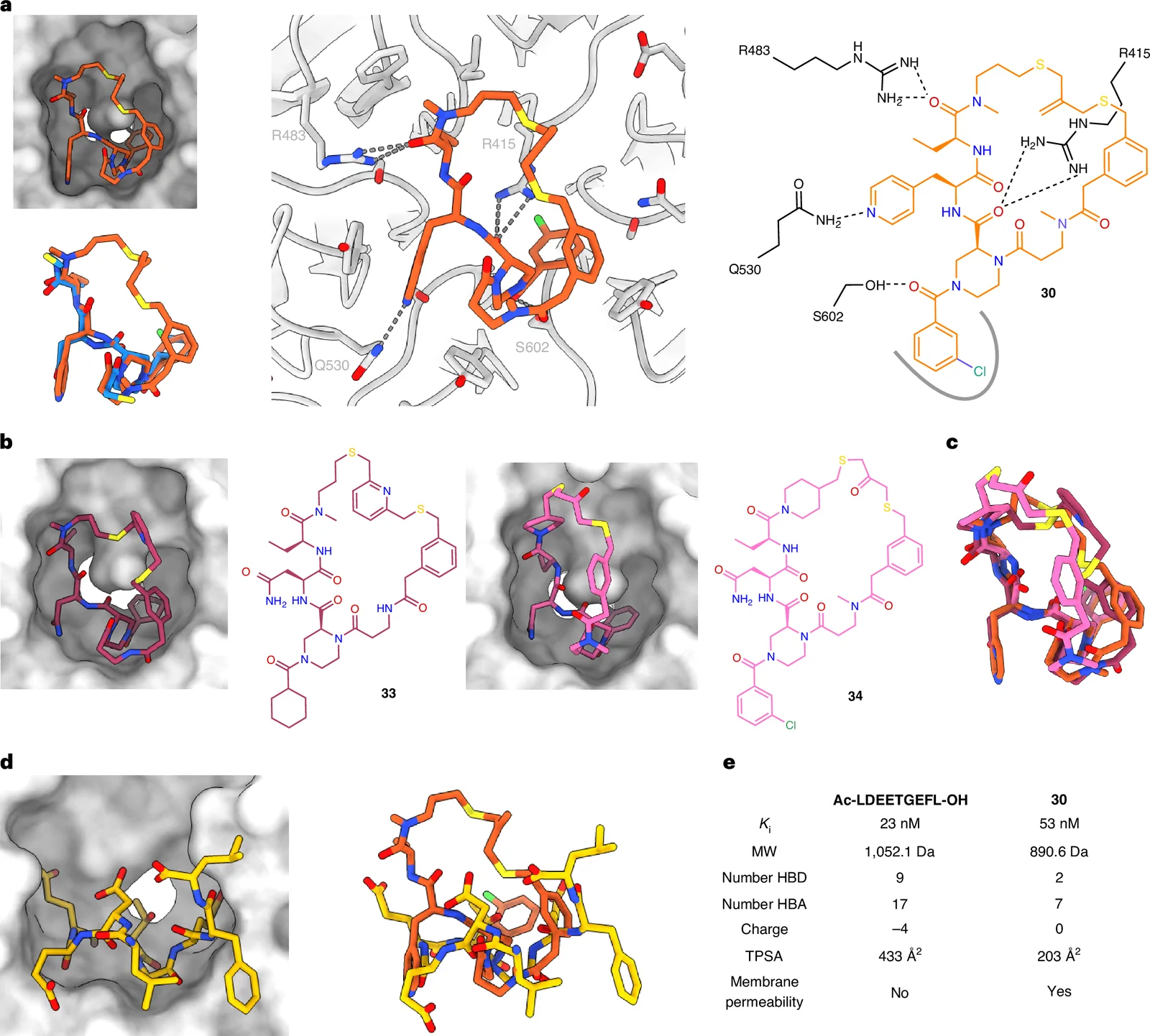
Structural analysis and physicochemical properties. **a**, X-ray structure of cyclic peptide **30** bound to Keap1 at 2.35-Å resolution (PDB 9QDU). Bottom left: structure showing an overlay of cyclic peptide **30** (orange) and linear peptide **6** (blue). H-bonds formed between **30** and Keap1 are shown in the zoomed-in view and in a chemical drawing. **b**, X-ray structures of cyclic peptides **33** (merlot-brown) and **34** (pink) bound to Keap1 at 1.95-Å (PDB 9QBS) and 2.33-Å (PDB 9QBT) resolutions, respectively. **c**, Superimposition of Keap1-bound cyclic peptides **30** (orange), **33** (merlot-brown) and **34** (pink). **d**, Superimposition of cyclic peptide **30** (orange) to known X-ray structure of the native Nrf2 peptide (yellow) bound to Keap1 (PDB 1X2R). **e**, Comparison of physicochemical properties between cyclic peptide **30** and the Nrf2-derived peptide ligand. MW, molecular weight; HBD, H-bond donor; HBA, H-bond acceptor.

To gain insight into how cyclic peptide **30**, despite having just two H-bond donors, binds tightly to the highly polar surface of Keap1, we solved its crystal structure in complex with the Keap1 Kelch domain (PDB 9QDU, 2.35-Å resolution; Fig. 6a and Supplementary Table 11). Structural superimposition with the Keap1–peptide **6** complex revealed that the core binding motifs of the two peptides align closely in space (Fig. 6a), confirming that key molecular interactions are preserved despite macrocyclization and polarity reduction. Peptide **30** engages Keap1 through six H-bonds, all formed by four of its H-bond acceptor groups, while none of its donor groups are involved (Fig. 6a and Supplementary Table 12). Compared to the asparagine residue in peptide **6**, the pyridine ring in peptide **30** forms fewer polar interactions, explaining its slightly reduced affinity. Strong shape complementarity and tight packing of nonpolar features, including the Abu ethyl side chain, the chlorophenyl group and the pyridine ring, highlight the important role of hydrophobic interactions in driving the high-affinity binding. The contributions of these various interactions were quantified in an SAR study of peptide **30** in which we synthesized eight variants with key groups such as amino acid side chains truncated or deleted (Supplementary Fig. 22).

Surprisingly, none of the optimized linkers in peptide **30** engaged in H-bonding, despite contributing to a ~1,000-fold increase in binding affinity during linker optimization. To further explore the structural role of macrocyclization linkers, we determined crystal structures for two additional cyclic peptides, **33** (*K*_i_ = 194 ± 2 nM; PDB 9QBS, 1.95 Å) and **34** (*K*_i_ = 167 ± 4 nM; PDB 9QBT, 2.33 Å; Fig. 6b). Structural overlays with peptide **30** revealed notable differences in the backbone trajectories of the linker regions. Overall, the X-ray structures suggested that the developed linker regions form mostly hydrophobic contacts that vary largely in the three studied cyclic peptides.

A comparison of the interaction formed by peptide **30** and the peptide region LDEETGEFL (L76–L84) of Nrf2 showed completely different backbone trajectories with no spatial overlap despite the peptidic nature of the two ligands, which engage Keap1 through entirely distinct sets of molecular contacts (Fig. 6d). This underscores the necessity of de novo design rather than trying to modify the native Nrf2 sequence, as it would have been impossible to transform Nrf2 into a ligand that mimics the interaction pattern achieved by peptide **30**. Notably, while the two peptides are similar in molecular weight (890.6 Da for **30** versus 1,052.1 Da for Nrf2) and binding affinity (53 nM versus 23 nM), peptide **30** is far less polar, having no charges (versus four negative charges in the Nrf2 sequence), featuring only two H-bond donors (versus nine) and a substantially lower topological polar surface area (TPSA of 203 Å^2^ versus 433 Å^2^). This reduction in polarity proved critical for enabling membrane permeability and intracellular PPI inhibition, capabilities that the native Nrf2 peptide lacks (Fig. 6e).

## Discussion

We successfully discovered a membrane-permeable PPI inhibitor based on a cyclic peptide by screening fully random libraries built from more than 100 chemically diverse building blocks. This unbiased approach, in which we did not incorporate any prior knowledge of the target or its natural ligands into the library design, marks a departure from previous strategies, where libraries were tailored by including building blocks known to interact with specific targets or were guided by structural insights. That potent, cell-permeable ligands can emerge from such a nonfocused library is promising, highlighting the potential of this approach for generating drug candidates against more difficult targets, especially those with no known binding fragments—a frequent challenge in the discovery of PPI inhibitors.

Interestingly, our identification of the two initial Keap1–Nrf2 inhibitors **1** and **2** from library 1 benefited from some unexpected allies: dimeric and trimeric peptide byproducts that showed enhanced activity, as well as potential covalent interactions through disulfide exchange with a target protein cysteine residue. The latter mechanism was intentionally used in the noughties in an elegant disulfide tethering strategy pioneered by J. Wells and colleagues, which exploits reversible covalent interactions to screen for ligandable cysteines^42,43^, a concept that led to landmark discoveries such as the identification of a covalent KRAS-G12C by Shokat and Wells^44^. In our case, this reactivity may have unintentionally influenced hit identification, revealing that our disulfide-cyclized peptide libraries may, in fact, be well suited for intentional disulfide tethering applications. Whether compound **1** would have emerged without contributions from multimeric byproducts that can form more contacts and/or disulfide tethering to C434 is unclear, especially at the 25 µM screening concentration used. For future efforts targeting more challenging proteins, we recommend increasing the screening concentration to 50 or even 100 µM, which is feasible given the scalable nanomole-scale synthesis used here, to better capture weaker interactions that might otherwise be missed.

We believe the strategy outlined here holds broad potential for tackling a wide range of disease-relevant targets. As we move toward more challenging proteins, expanding both the chemical diversity and the size of the libraries will be essential. The diversity of building blocks can be readily scaled as there are over 2,000 commercially available Fmoc-protected amino acids, of which around 500 are especially suitable for library synthesis, and an even greater number of carboxylic acids. In library 1, we used just ten carboxylic acids across all 1,536 peptide cores but this can be easily diversified to produce libraries with far more distinct chemical structures. Realistically, libraries comprising around 100,000 synthetic cyclic peptides are well within reach using the presented workflow. Computational methods, including machine learning, may be used to design libraries with a higher chance of target engagement or membrane permeation than randomly chosen sequences. With such further improvements, the door might be opened for the discovery of ligands for intracellular PPIs that have long been considered undruggable.

## Methods

### Calculation of physicochemical properties of cyclic peptides

The structures of cyclized peptides containing free amines and carboxylic acids were generated in ChemDraw 20.1 and exported as two separate ChemDraw files in SD format. The acylated cyclic peptide structures (that is, all possible combinations of cyclic peptides and carboxylic acids) were generated using the ‘enumerate combinatorial library’ tool in the DataWarrior software (version 5.2.1; www.openmolecules.org). We defined the formation of an amide bond to take place between the nitrogen atoms of the cyclic peptides and the carboxylic acid groups of the carboxylic acids. The nitrogen atom and carboxylic acid definitions were previously described^28^. The physical and chemical properties, including molecular weight, water–octanol partitioning coefficient (cLogP), TPSA, number of H-bond donors and number of H-bond acceptors, were calculated directly from the compound library using the prediction tools provided by DataWarrior.

### Cloning, expression and purification of Keap1 and mutants

Recombinant human Kelch domain of Keap1 (321–609) with an N-terminal addition of His_6_ tag and tobacco etch virus (TEV) cleavage site for tag removal was cloned into the vector pET29b(+) (Novagen) for bacterial expression. The DNA coding these elements was synthesized (GenScript) and inserted using the restriction enzymes NdeI and XhoI. The ligated vector was transformed into NEB 5-alpha competent *Escherichia* *coli* cells and the cells were plated on kanamycin-selective 2×YT agar plates. The DNA coding for the Keap1 protein was sequenced to verify the correctness of the sequence and the plasmid was transformed into BL21(DE3) competent *E*. *coli* cells (New England Biolabs) for expression. The plasmids encoding Keap1 mutants (Keap1-E540A;E542A and Keap1-C434A) were prepared following the same procedure.

For expression, cells from colonies grown on 2×YT agar plates with 50 µg ml^−1^ kanamycin were used to inoculate a 20-ml starter culture of 2×YT with 50 µg ml^−1^ kanamycin and grown overnight at 37 °C while being shaken. A volume of 800 ml of 2×YT in a 2.5-L shaking flask was inoculated with the starter cultures by 1:100 dilution and grown at 37 °C under shaking at 200 rpm until the logarithmic growth phase was reached. Protein expression was induced by adding a final concentration of 0.5 mM IPTG and the culture was incubated at 20 °C while being shaken overnight (200 rpm). Cells were pelleted by centrifugation at 4,000*g* for 10 min. Cells were lysed by sonication in 80 ml of lysis buffer (50 mM Tris, 500 mM NaCl, 20 mM imidazole and 10% ethylene glycol, pH 8.0). The lysate was centrifuged at 16,000*g* for 1 h at 4 °C to remove insoluble material. The desired protein was purified from the culture supernatant by Ni-NTA affinity chromatography. Imidazole in the eluted protein was removed by PD-10 column (Cytiva) using Tris buffer (25 mM Tris and 20 mM NaCl, pH 8.0) and the protein was purified by size-exclusion chromatography (Cytiva Superdex 75 10/300 GL) using the same buffer. Purified protein was concentrated using a spin filter (Microsep Advance centrifugal device with Omega membrane 10k, Pall) and the concentration was quantified by absorbance measurement at 280 nm using a NanoDrop 8000 Spectrophotometer. The purified protein concentrate was portioned into aliquots, snap-frozen in liquid nitrogen and stored at −80 °C until needed.

### HTS for Keap1–Nrf2 inhibition by TR-FRET

The binding of the peptides of library 1 to the Nrf2-binding site of Keap1 was assessed using a TR-FRET competition assay with Sulfo-Cy5-labeled Nrf2 peptide (sequence: Ac-propargylglycine-Peg2-LDEETGEFL-NH_2_; Cy5-labeled Nrf2 peptide) as the probe that was to be displaced by cyclic peptides. For the HTS, the N-terminally His-tagged Keap1 Kelch domain, a europium-labeled anti-His antibody (LANCE-Eu-W1024 anti-His_6_, PerkinElmer; Eu-labeled anti-His) and the above peptide probe were premixed to form a FRET donor and acceptor complex before adding to wells containing library compounds. The screen was performed in 384-well plates (Thermo Fisher NUNC 384 shallow plates, 264705) that already contained 5 µl of 77 µM acylated cyclic peptides with a DMSO content of 4.5% (v/v) in Tris buffer. To each well of the plates, 10 μl of a mixture of Eu-labeled anti-His:His–Keap1:Cy5-labeled Nrf2 peptide (25 mM Tris, 20 mM NaCl, 0.015% (w/v) Tween-20 and 0.75% (w/v) BSA, pH 7.4, supplemented with 0.75 nM Eu-labeled anti-His, 30 nM Keap1 and 60 nM Cy5-labeled Nrf2 peptide) was added by bulk dispensing (Certus) to reach a final volume of 15 μl and the following final concentrations: 25 mM Tris, 20 mM NaCl, 0.01% (w/v) Tween-20 and 0.5% (w/v) BSA, pH 7.4, supplemented with 0.5 nM Eu-labeled anti-His, 20 nM Keap1, 40 nM Cy5-labeled Nrf2 peptide, 25 µM cyclic peptide library compounds and 1.5% DMSO. In each plate, eight wells were used for a positive control (omission of Keap1) and eight wells were used for negative controls (no cyclic peptide). The plates were sealed with plastic films (Greiner, 676001), centrifuged at 500*g* for 2 min in a centrifuge (around 2,000 rpm with SIGMA 4-16KS) to remove air bubbles and incubated for 2 h at room temperature; the TR-FRET signal measured using a plate reader (PHERAstar FSX, BMG, SX software, version 5.70, edition 8) that excited at 337 nm with a laser and measured with a 60-μs emission delay at both 620 nm (from europium) and 665 nm (from Cy5) with a 400-μs measurement window. The percentage of probe displacement was calculated using equation (1).

1 $$\% \,\mathrm{peptide}\,\mathrm{displacement}=\frac{N-X}{N-P}\times 100$$

where *N* is the average FRET signal of the eight negative control wells, *X* is the FRET signal of library cyclic peptide compounds and *P* is the FRET signal of the eight positive control wells. The FRET signals were calculated by forming a ratio of the Cy5 and Europium emission multiplied by 10,000.

### Crude peptide IC_50_ and active species identification

The identified hits from the TR-FRET screen were newly synthesized on a 160-nmol scale by reacting 25 μl of 6.4 mM cyclic peptide in DMSO containing 150 mM DABCO with 25 μl of 64 mM carboxylic acid, 64 mM HBTU and 64 mM DABCO for 5 h at room temperature. The remaining activated ester was quenched by adding 1 ml of 60 mM NH_4_HCO_3_ buffer, followed by overnight incubation. The reaction mixture was dried by vacuum centrifugal evaporation (Christ RVC 2-33 CD plus IR instrument; 30 °C, 1,500 rpm, 0.1 mbar). The dried product was dissolved in 16 μl of DMSO to obtain an estimated concentration of 10 mM crude peptide.

The activity of crude peptides was measured using the TR-FRET assay described above. A twofold dilution series of the crude peptides was prepared in a LDV ECHO source plate using DMSO. Using acoustic droplet transfer, 160 nl of each dilution was transferred to a 384-well destination plate (Nunc, 264705). To each well, 15 μl of premixed TR-FRET complex in HEPES buffer (10 mM HEPES, 150 mM NaCl, 0.005% (w/v) Tween-20 and 0.5% (w/v) BSA, pH 7.4, supplemented with 0.5 nM Eu-labeled anti-His, 20 nM Keap1, 40 nM Cy5-labeled Nrf2 peptide) was added using bulk dispensing. The probe displacement was measured and calculated as described for the HTS. The IC_50_ was determined by plotting the percentage of peptide displacement against the corresponding peptides concentration, followed by curve fitting to the data using equation (2).

2 $$y=\frac{{x}^{\mathrm{Hill}\,\mathrm{slope}}\times 100}{{x}^{\mathrm{Hill}\,\mathrm{slope}}+{c}^{\mathrm{Hill}\,\mathrm{slope}}}$$

where parameter *x* is the peptide concentration, *y* is the percentage of peptide probe that is displaced at each peptide concentration and *c* is the IC_50_ value to be determined.

For the identification of active species in the crude products, 15 μl of crude product (around 10 mM cyclic peptide in DMSO) was mixed with 1.5 ml of a 1:2 acetonitrile and double distilled (dd)H_2_O (v/v) solution and subjected to preparative reversed-phase HPLC using a C18 column. The separation was performed with a 10–70% gradient of solvent B over 24 min, with fractions collected at 1-min intervals. The collected fractions were dried by vacuum centrifugal evaporation (30 °C, 1,500 rpm, 0.1 mbar) and subsequently dissolved in 15 μl of DMSO to prepare fraction stocks. Volumes of 37.5 nl from each fraction stock were transferred to 384-well destination plates and their activities were assessed using the same TR-FRET assay as described above.

### Cocrystallization of Keap1 with peptides

The Keap1 Kelch domain with the two substitutions (E540A and E542A)^37^ was used for X-ray crystallization. Before crystallization, the His tag was cleaved by TEV enzyme in a 1:100 molar ratio (TEV:Keap1) overnight at 4 °C. The cleaved protein was purified by size-exclusion chromatography as described above, supplemented with DTT to reach 2 mM and concentrated to a final concentration of 8–12 mg ml^−1^ in buffer (25 mM Tris, 10 mM NaCl and 2 mM DTT, pH 8.0) using a centrifugation device with a 10-kDa cutoff. Cocrystallization was performed by mixing Keap1 at 12 mg ml^−1^ with an eightfold molar excess of peptide **6**, **30**, **33** and **34**. The recombinant Keap1 Kelch domain bound to the peptide was crystallized at 18 °C using the sitting-drop vapor diffusion approach. Initial screening of 4 × 96 crystallization conditions was performed with a Mosquito automated crystallization robot (SPT Labtech). Each drop contained 200 nl of protein solution mixed with 200 nl of precipitant solution and was equilibrated against 50 μl of reservoir solution in a 96-well plate. Crystals generally formed within a few days. Suitable crystals were collected, cryoprotected using the mother liquor supplemented with 25% glycerol and rapidly frozen in liquid nitrogen.

### X-ray structure determination

Diffraction data for crystals of the Keap1 Kelch domain bound to peptides **6** and **30** (originally recorded in the lab notebook as P1Thro and AsR5-L12) were collected at the MASSIF-1 and MASSIF-3 beamlines of the European Synchrotron Radiation Facility (ESRF). Data for complexes with peptides **33** and **34** (lab notebook identifiers P3 and P6) were acquired at beamline PX-I of the Swiss Light Source at the Paul Scherrer Institute (SLS). Raw data were processed with autoprocess pipeline^45^, implementing the program XDS (XDS Program Package)^46^, along with Pointless and Aimless from the CCP suite^47–49^. The structure was solved by molecular replacement using PHENIX (version 1.20.1)^50^ with the atomic coordinates of Keap1 (PDB 1U6D) as a search model. The peptide was manually modeled into the extra density using Coot (version 0.9.4.1)^51^. The structures were finalized through iterative cycles of refinement in PHENIX and manual model building in Coot. Data collection and refinement parameters are summarized in Supplementary Tables 7 and 11. The atomic coordinates and corresponding structure factors were deposited to the Protein Data Bank under accession codes 9IH9, 9QDU, 9QBS and 9QBT for Keap1 complexes with peptides **6**, **30**, **33** and **34**, respectively. Figures were produced using ChimeraX (version 1.8; https://www.cgl.ucsf.edu/chimerax/).

### Peptide quantification by Ellman’s reagent

The concentration of peptides containing thiol groups was determined using Ellman’s reagent, wherein the reducing agent BDT was previously removed by vacuum centrifugal evaporation as follows. Typically, 40 μl of thiol peptide released from the solid support was transferred to wells of a 384-well source plate (Echo qualified 384-well PP, clear). A volume of 15 μl of 5% TFA in water (v/v) (675 mM) was added to neutralize TEA. The sample was then subjected to vacuum centrifugal evaporation to remove solvent and reducing reagent (BDT). Upon completion, 30 μl of DMSO was added to each well by bulk dispensing (Certus) to redissolve the peptides. A volume of 50 nl of peptide was transferred by acoustic droplet ejection to a 384-well black microplate with a transparent bottom (Greiner, 781096). Ellman’s reagent (DTNB) was dissolved in assay buffer (150 mM NH_4_HCO_3_ in ddH_2_O and DMSO (90:10, v/v), pH 8) to a concentration of 10 mM. Assay buffer (40 µl) was added by bulk dispensing (Certus) before the addition of DTNB solution (10 μl). The plates were centrifuged at 500*g* in a centrifuge (around 2,000 rpm with Sigma 4-16KS) for 2 min and absorbance (412 nm) was measured on a TECAN M200 plate reader (iControl software, version 1.12). Peptide concentrations were determined using a calibration curved prepared with dilutions of cysteine or DTT.

### Keap1–Nrf2 FP competition binding assay

An FP competition assay was used to measure the binding of peptides to the Nrf2-binding site of the Keap1 Kelch domain. The peptides displaced the fluorescein-labeled Nrf2 peptide (sequence: FAM-LDEETGEFL-NH_2_). In contrast to the TR-FRET assay, this method allowed measuring binding in presence of reducing agent. The assay was performed in 384-well plates using HEPES buffer (10 mM HEPES, 150 mM NaCl and 0.005% (w/v) Tween-20, pH 7.4) containing 1 mM TCEP, 10 nM fluorescein-labeled Nrf2 peptide and 30 nM Keap1. A series of twofold dilutions of selected linear peptides from DMSO stocks (6 mM) were prepared in Echo qualified 384-well LDV source plates and transferred to 384-well destination plates by acoustic droplet ejection to obtain volumes of 160 nl per well. A volume of 15 μl of premixed FP assay reagent mixture described above was added to each well using a liquid bulk dispenser (Certus) and incubated for 15 min in the dark at room temperature. FP was measured with a PHERAstar FSX microplate reader (excitation: 485 nm, emission: 535 nm) at 25 °C. The data were analyzed by Excel (version 2016). On each plate, wells with a positive control (displacement of fluorescein-labeled Nrf2 peptide by Keap1 inhibitor KI696; MedChemExpress, HY-101140) and wells with a negative control (no peptide) were included. The percentage of probe displacement was calculated using equation (1).

### Quantifying Keap1 reacted with covalent inhibitor

The percentage of Keap1 protein modified with covalent inhibitor at C434 was determined by quantifying the amount of free Keap1 using the fluorescein-labeled Nrf2 peptide as probe and measuring FP. A volume of 2.5 nl of peptide (6 mM in DMSO) was transferred from a well of a 384-well PP source plate to a well of a 384-well destination plate by acoustic droplet ejection. A volume of 15 μl of solution containing HEPES buffer (10 mM HEPES, 150 mM NaCl and 0.005% (w/v) Tween-20, pH 7.4), 50 μM TCEP, 10 nM fluorescein-labeled Nrf2 peptide and 30 nM Keap1 was added to the well using bulk dispensing. The mixture was incubated in the dark at room temperature and the FP was measured after 2 min, 15 min, 30 min, 1 h, 2 h, 4 h, 6 h, 8 h, 10 h, 12 h, 14 h and 16 h in the dark at room temperature. The FP readings and result calculation were performed as described above.

### Time-course effects of 20-Cl and 20-Ac on Keap1-binding activity

The percentage of Keap1 protein modified with the inhibitor or control was determined by the FP competition assay using the conditions described for covalent inhibitors above. A series of twofold dilutions of covalent peptides from 6 mM DMSO stock were prepared in Echo qualified 384-well LDV source plates and 160 nl was transferred to 384-well destination plates by acoustic droplet ejection. A volume of 15 μl of premixed buffer containing Keap1, fluoresceine-labeled Nrf2 peptide and TCEP at concentrations described above was added to each well using the bulk dispenser and incubated for 2 min, 15 min, 30 min, 1 h, 2 h, 4 h, 6 h, 8 h, 10 h, 12 h, 14 h and 16 h in the dark at room temperature. An FP reading was taken at each time point and the data were processed as described above to determine the IC_50_ values for all time points.

### Determination of *k*_inact_ and *K*_i_ of covalent inhibitors

A dilution series (twofold) was prepared for the inhibitors using DMSO as solvent. Of each dilution, 160 nl was transferred by acoustic dispensing to 15 μl of premixed FP assay buffer containing 50 μM TCEP. Reactions were incubated in the dark at room temperature and FP was measured after 1 h, 2 h, 4 h, 6 h, 8 h, 10 h, 12 h, 14 h and 16 h. The data of FP readings for each time point were processed as described above to calculate the percentage of peptide displacement. The *k*_obs_ value, an observed first-order rate constant, was determined at each inhibitor concentration using GraphPad Prism (version 9.4.1) and equation (3).

3 $$\% \,\mathrm{peptide}\,\mathrm{displacement}=a+\left(100-a\right)\times \left(1-{e}^{(-{k}_{\mathrm{obs}}\times t)}\right)$$

where *α* is an initial percentage of peptide probe displacement and *t* is the incubation time.

The *k*_obs_ values were then fitted to equation (4) to determine *k*_inact_ and K_*i*_.

4 $${k}_{\mathrm{obs}}=\frac{{k}_{\mathrm{inact}}\times I}{{K}_{\mathrm{i}}+I}$$

where *k*_inact_ is a first-order rate constant describing the maximum potential rate of covalent bond formation, *K*_i_ is the concentration of peptide required for half of the maximal potential rate of covalent bond formation and *I* is the concentration of the inhibitor.

### MS analysis of Keap1 Kelch domain treated with covalent inhibitor

Purified recombinant Kelch domain of Keap1 (321–609) with a His tag or its mutant variant, Keap1-C434A, at a final concentration of 2.5 μM was incubated with 25 μM final concentration of covalent inhibitor in PBS (10 mM Na_2_HPO_4_, 1.8 mM KH_2_PO_4_, 137 mM NaCl and 2.7 mM KCl, pH 7.4) in a volume of 50 μl. After 14 h, the reaction mixture was run over a Zeba spin desalting column (7-kDa molecular weight cutoff, Thermo Fisher Scientific; equilibrated three times with PBS buffer) to remove unreacted inhibitor. In parallel, Keap1 and Keap1-C434A not incubated with inhibitor were prepared as controls using the same procedure. The proteins were analyzed by LC–MS using reversed-phase separation and high-resolution MS analysis of denatured protein, as described below. The samples were diluted 100-fold with MilliQ water containing 0.1% formic acid and 2 μl of diluted samples (around 100 ng protein) were injected into the LC–MS. The UPLC (Vanquish Horizon, Thermo Fisher Scientific) was equipped with an Aquity UPLC Protein BEH C4 precolumn (2.1 × 5 mm, 1.7 µm, 300 Å, Waters) in an oven at 45 °C. The samples were run at a flow rate of 400 μl min^−1^ using a linear gradient of 15–50% (v/v) solvent B (99.9% (v/v) acetonitrile + 0.1% (v/v) HCOOH) over solvent A (99.9% (v/v) H_2_O + 0.1% (v/v) HCOOH) within 2.5 min. Eluting proteins were analyzed using an Exploris 240 Orbitrap FT-MS instrument (Thermo Fisher Scientific) operated in the intact protein mode with the low-pressure option activated. Mass spectra were acquired as full scans in the range of 700–4,000 *m*/*z* at a resolution of 15,000, with the radiofrequency lens set to 120%, a normalized automatic gain control target of 200% and a surface-induced dissociation of 15 eV. Chipsoft software (Advion BioScience, version 8.3.1) was used. The mass spectra were deconvolved using Biopharma Finder 4.1 (Thermo Fisher Scientific) using the Respect algorithm.

### Determination of IC_50_ and *K*_i_ by FP competition assay

A dilution series (twofold) was prepared for the inhibitors using DMSO as solvent, starting from 3 mM (cyclic peptides) or 6 mM DMSO stocks (linear peptides), and 160 nl of each dilution was transferred to 384-well destination plates using acoustic droplet ejection. A volume of 15 μl of premixed FP assay reagent containing 1 mM TCEP was added to each well using a bulk dispenser and incubated for 15 min in the dark at room temperature. FP was measured and the IC_50_ values were determined as described above. *K*_i_ values were calculated using equations (5) and (6) (ref. ^52^).

5 $${I}_{50}={\mathrm{IC}}_{50}\,-{P}_{\mathrm{T}}+{K}_{\mathrm{d}}\times \frac{{\mathrm{PL}}_{50}}{{L}_{50}}+{\mathrm{PL}}_{50}$$

where *I*_50_ is the concentration of the free test peptide at 50% inhibition, *P*_T_ is the concentration of the total Keap1 in the assay, *K*_d_ is the dissociation constant of the Keap1–Nfr2 peptide complex, PL_50_ is the concentration of Keap1–Nfr2 peptide complex when 50% inhibition is achieved and *L*_50_ is the concentration of the free labeled Nrf2 peptide at 50% inhibition.

6 $${K}_{{\rm{i}}}=\frac{{I}_{50}}{\frac{{L}_{50}}{{K}_{\mathrm{d}}}+\frac{{P}_{0}}{{K}_{\mathrm{d}}}+1}$$

where *P*_0_ is the concentration of the free Keap1 at 0% inhibition.

### Measuring Keap1–Nrf2 inhibition with cellular assay

The cellular activity of cyclic peptides was determined using the Nrf2/ARE responsive luciferase reporter HEK293 cell assay (Signosis, SL-0042), in which firefly luciferase is expressed under the control of Nfr2/ARE response element for monitoring the activation of Nrf2 pathway triggered by the inhibition of Keap1–Nrf2 interaction resulting in reduced Nrf2 degradation. The cells were seeded in 96-well plates (Greiner, 655098) at a density of 4 × 10^4^ cells per well in 100 μl of high-glucose DMEM that was supplemented with 10% FBS, 100 U per ml penicillin and 100 μg ml^−1^ streptomycin. The cells were grown for 24 h in a humidified incubator at 37 °C with 5% CO_2_. For each cyclic peptide, five solutions with peptide concentrations of 25, 50, 100, 200 and 400 μM in culture medium and 4% DMSO were prepared. A volume of 25 μl of each peptide dilution was added per well already containing 100 μl of medium, achieving a final peptide concentration of 5, 10, 20, 40 and 80 μM and the same quantity of DMSO of 0.8% in each well. The cells were incubated for an additional 14 h. The Nrf2-inducing agent sulforaphane (SFN) was used as a positive control, an inactive peptide (two amino acids swapped) served as a negative control and 0.8% DMSO was included as a solvent control. The plate was gently moved before being incubated for 14 h at 37 °C with 5% CO_2_. For measuring luciferase expression, the plates were equilibrated to room temperature, 100 μl of Steady-Glo luciferase assay reagent (Promega, E2510) was added to each well and the plate was gently centrifuged at 350*g* for 1 min (around 1,000 rpm with Sigma 4-16KS) to allow sufficient cell lysis. Luminescence was measured with a Tecan Infinite F200 Pro plate reader.

### CAPA

CAPA was conducted as reported by Kritzer et al.^40^. The HeLa cell line that stably expresses the HaloTag–GFP–mito complex was cultured in DMEM that was supplemented with 10% FBS, 100 U per ml penicillin, 100 μg ml^−1^ streptomycin and 1 μg ml^−1^ puromycin. The cells were incubated at 37 °C with 5% CO_2_ and passaged every 2–3 days. Once the cells were near full confluency, they were seeded into a 96-well plate (Brandplates, 781662) at a density of 4 × 10^4^ cells per well 1 day before the experiment. On the day of the experiment, the culture medium was aspirated and replaced with 100 μl of Opti-MEM (Gibco, 11058-021). Then, 10 μl of CA peptide stocks (1 mM) in DMSO were diluted with 190 μl of Opti-MEM to prepare the initial dilution; then, serial threefold dilutions were prepared from the first dilution using a 95:5 Opti-MEM/DMSO (v/v) solution, ensuring a constant DMSO concentration of 5% throughout all samples. Subsequently, 25 μl of peptide serial dilutions were added to each well, achieving a final concentration from 10 μM to 4.6 nM with a constant final DMSO concentration of 1%. The plate was gently moved to allow optimal mixing and incubated for 4 h at 37 °C with 5% CO_2_. Following aspiration of medium and compound from the wells, the cells were incubated with 5 μM CA-TAMRA in Opti-MEM (50 μl) for 15 min. After incubation, the medium and chase probe were aspirated and the cells were washed with 80 μl of fresh Opti-MEM for 30 min to remove the excess dye. After aspirating the Opti-MEM from the wells, the cells were washed again with 80 μl of PBS buffer (pH 7.4) for 1 min, before being trypsinized with 40 μl of 0.05% trypsin–EDTA (Gibco, 15400-054) for 5 min. After adding a volume of 180 μl of PBS buffer (pH 7.4), the cells were analyzed by flow cytometry (Thermo Fisher, Attune NxT). Gating strategies are depicted in Supplementary Fig. 23. For each well, cells were gated for live and single-cell events on the basis of their size in the forward scatter and their complexity in the side scatter. The single-cell population was then gated for the double-positive labeling signal, for which 2,000 GFP-positive single cells were analyzed. Raw data were processed using FlowJo (version 10.8). The measured fluorescence intensity of TAMRA was normalized on the basis of the GFP signal using equation (7).

7 $$\begin{array}{l}{\rm{Sample}}\,{\rm{fluorescence}}\,{\rm{ratio}}\\ =\displaystyle \frac{{\mathrm{Median}}\,{\mathrm{TAMRA}}\,{\mathrm{fluorescence}}\,{\mathrm{of}}\,{\mathrm{gated}}\,{\mathrm{GFP}}^{+}\,{\mathrm{distribution}}}{{\mathrm{Median}}\,{\mathrm{GFP}}\,{\mathrm{fluorescence}}\,{\mathrm{of}}\,{\mathrm{gated}}\,{\mathrm{GFP}}^{+}\,{\mathrm{distribution}}}\end{array}$$

This calculated raw fluorescence fraction was then normalized against minimal TAMRA fluorescence (10 mM Trp-CA, positive control) and maximal TAMRA fluorescence (DMSO, negative control) using equation (8).

8 $$\% \mathrm{HaloTag}\,\mathrm{labeling}=\frac{\mathrm{Negative}\,\mathrm{control}-\mathrm{Sample}\,\mathrm{fluorescence}\,\mathrm{ratio}}{\mathrm{Negative}\,\mathrm{control}-\mathrm{Positive}\,\mathrm{control}}\times 100$$

Finally, the data were plotted as dose–response curves in GraphPad Prism (version 9.4.1) to determine the CP_50_ value, which represents the concentration at which 50% of the Halo protein is occupied.

### Passive artificial membrane permeability

Acceptor wells were prepared by adding 300 µl of PBS buffer (pH 7.4) containing 5% DMSO to a Teflon acceptor plate (Sigma-Millipore, MSSACCEPT0R) and cyclic peptide or reference compound solutions were prepared at a concentration of 50 μM in PBS buffer (pH 7.4) containing 5% DMSO. Volumes of 150 μl of these solutions were placed into wells of a 96-well precoated PAMPA plate (Corning, 353015), which were then inserted into the acceptor plate. After overnight incubation (12 h) at room temperature, cyclic peptide concentrations in the acceptor solutions were determined by LC–MS analysis. Ions detected in the positive ion mode were counted over a mass of [*M* + 0.5] to [*M* + 3.5]. Concentrations and ion counts at equilibrium were determined by LC–MS analysis of 16.7 μM solutions of cyclic peptides in PBS buffer containing 5% DMSO. All samples were prepared and measured in triplicates on the same plate. The apparent permeability coefficient *P*_app_ was calculated using equation (9).

9 $${P}_{\mathrm{app}}=\frac{{V}_{{\rm{D}}}\times {V}_{{\rm{A}}}}{\left({V}_{{\rm{D}}}+{V}_{{\rm{A}}}\right)\times A\times t}\times -\mathrm{ln}\left(1-\frac{\left[{\mathrm{Peptide}}_{\mathrm{Acceptor}}\right]}{\left[{\mathrm{Peptide}}_{\mathrm{Equilibrated}}\right]}\right)$$

The parameter [Peptide_Acceptor_] represents the AUC measured by LC–MS for the acceptor wells and [Peptide_Equilibrated_] is the AUC measured for the samples diluted to the theoretical equilibrium. *V*_D_ and *V*_A_ are the donor and acceptor well volumes, respectively; *A* is the area of the membrane (0.3 cm^3^) and *t* is the incubation time in seconds.

### Caco-2 permeability assay

The permeability of designed cyclic peptides was evaluated using a Caco-2 permeability assay that was performed by Medicilon Preclinical Research. In brief, cells were seeded into the 96-well Transwell plates with 0.4-μm-pore polycarbonate membrane inserts (Corning, 3391) at 1 × 10^5^ cells per cm^2^ for up to 14–28 days. The medium contained DMEM supplemented with 10% FBS, 2 mM L-glutamine, 100 U per ml penicillin and 100 μg ml^−1^ streptomycin. The medium in plates was renewed every 3–4 days. The inserts received 75 μl of assay medium in the upper compartment (apical chamber) and 250 μl in the bottom compartment (basolateral chamber) individually. When the transepithelial electrical resistance exceeded 500 Ω cm^2^, indicating an intact cell layer, the medium in the apical and basolateral chamber was separately replaced with peptide solutions at 10 μM or Hanks’ balanced salt solution. The plates were incubated for 120 min at 37 °C. The peptide concentration of solutions in the apical and basolateral compartments was subsequently quantified using LC–MS to evaluate the transmembrane flux of peptides in the apical-to-basolateral direction or the basolateral-to-apical direction. Membrane integrity of the Caco-2 cell monolayer was validated using Lucifer yellow paracellular permeability testing after the assays. Nadolol, propranolol and digoxin were used as controls. The apparent permeability coefficient *P*_app_ was calculated using equation 10.

10 $${P}_{\mathrm{app}}=\frac{{\rm{d}}{C}_{\mathrm{r}}}{{\rm{d}}t}\times \frac{{V}_{\mathrm{r}}}{A\times {{C}}_{0}}$$

where d*C*_r_/d*t* is the cumulative concentration of compound in the receiver chamber as a function of time in seconds, *V*_r_ is the solution volume in the receiver chamber (0.1 ml on the apical side and 0.3 ml on the basolateral side), *A* is the area of the monolayer (0.143 cm^2^) and *C*_0_ is the initial concentration in the donor chamber.

### Reporting summary

Further information on research design is available in the Nature Portfolio Reporting Summary linked to this article.

## Online content

Any methods, additional references, Nature Portfolio reporting summaries, source data, extended data, supplementary information, acknowledgements, peer review information; details of author contributions and competing interests; and statements of data and code availability are available at https://doi.org/10.1038/s41589-026-02237-7.

## Supplementary information

Supplementary Information Supplementary Figs. 1–25, Tables 1–12 and Methods (chemical synthesis methods).

Reporting Summary

## Source data

Source Data Figs. 1–5, Extended Data Fig. 1 and Supplementary Figs. 1–22 Raw data.

## Data Availability

Additional figures, tables and chemical synthesis methods are provided in Supplementary Information. The X-ray structure data of peptides bound to Keap1 were deposited to the Protein Data Bank under accession codes 9IH9, 9QDU, 9QBS and 9QBT. Data are available from the corresponding author upon request. Source data are provided with this paper.
