# Supplementary material for: Generation of membrane-permeable cyclic peptides inhibiting protein–protein interaction
Source: Nat Chem Biol. 2026 Jun 1;22(8):1351–61. doi: 10.1038/s41589-026-02237-7 (PMC13661285; doi:10.1038/s41589-026-02237-7)

nature chemical biology

Supplementary information https://doi.org/10.1038/s41589-026-02237-7

Generation of membrane-permeable cyclic peptides inhibiting protein–protein interaction

In the format provided by the authors and unedited

Table of Contents

Supplementary Figures

Supplementary Figure 1. Physico-chemical properties of Library 1. 3

Supplementary Figure 2. Concentration of cyclic peptide cores of Library 1 4

Supplementary Figure 3. Quality of cyclic peptide cores of Library 1 5

Supplementary Figure 4. Keap1 cloning and expression for TR-FRET assay 13

Supplementary Figure 5. TR-FRET-based Keap1–Nrf2 inhibition assay 14

Supplementary Figure 6. Additional hits of HTS 15

Supplementary Figure 7. Active species in crude reaction of hit 1 and 2 16

Supplementary Figure 8. HPLC-purified peptide 4 and analogs 17

Supplementary Figure 9. Keap1 cloning and expression for X-ray structure 18

Supplementary Figure 10. X-ray structure of peptide 6 bound to Keap1 19

Supplementary Figure 11. Models of cyclic peptides based on peptide 6 20

Supplementary Figure 12. Structure and activity of peptides 8-18 21

Supplementary Figure 13. Purity of peptides 19-21 containing warheads 24

Supplementary Figure 14. Inhibition of Keap1–Nrf2 by covalent inhibitor 25

Supplementary Figure 15. K_{obs} of covalent inhibitor peptide 20-Cl 27

Supplementary Figure 16. Keap1 cloning and expression for mass spectrometry 28

Supplementary Figure 17. Purity of cyclic peptides 22-28 29

Supplementary Figure 18. Covalent kinetic parameters of peptide 28 30

Supplementary Figure 19. Purity of cyclic peptides 23a-CA1, 23b-CA, 29-34 31

Supplementary Figure 20. Repetition of cellular activity assays 32

Supplementary Figure 21. Membrane permeability measurements 33

Supplementary Figure 22. Structure-activity study of peptide 30 34

Supplementary Figure 23. Gating strategies for CAPA assay analysis 36

Supplementary Figure 24. NMR spectra of M2 37

Supplementary Figure 25. NMR spectra of M3 38

Supplementary Tables

Supplementary Table 1. Amino acids with A code 39

Supplementary Table 2. Amino acids with D code 43

Supplementary Table 3. Amino acids with X code 45

Supplementary Table 4. Carboxylic acids with C code 50

Supplementary Table 5. Quality analysis of cyclic peptide of Library 1 57

Supplementary Table 6. Overview of HTS conditions 59

Supplementary Table 7. X-ray data for peptide 6 bound to Keap1 62

Supplementary Table 8. Crystallization attempts and results 62

Supplementary Table 9. Interactions between peptide 6 and Keap1 65

Supplementary Table 10. Structure-activity relationship study of peptide 6 66

Supplementary Table 11. X-ray data of bound peptides 30, 33 and 34 120

Supplementary Table 12. Interactions between peptide 30 and Keap1 121

Supplementary Methods

Quality of reagents 122

LC-MS analysis 122

Preparative HPLC 122

Sequences of synthetic DNA 123

Random cyclic peptide library synthesis 125

Peptide quantification by absorption 126

Synthesis of disulfide-cyclized peptide and purification 126

High-throughput synthesis of linear peptides for SAR analysis 127

Synthesis of peptides with warheads 128

Chemical synthesis of library building blocks 129

High-throughput synthesis of linker-cyclized peptide library 130

Synthesis and purification of linker-cyclized peptides 131

Supplementary Figures

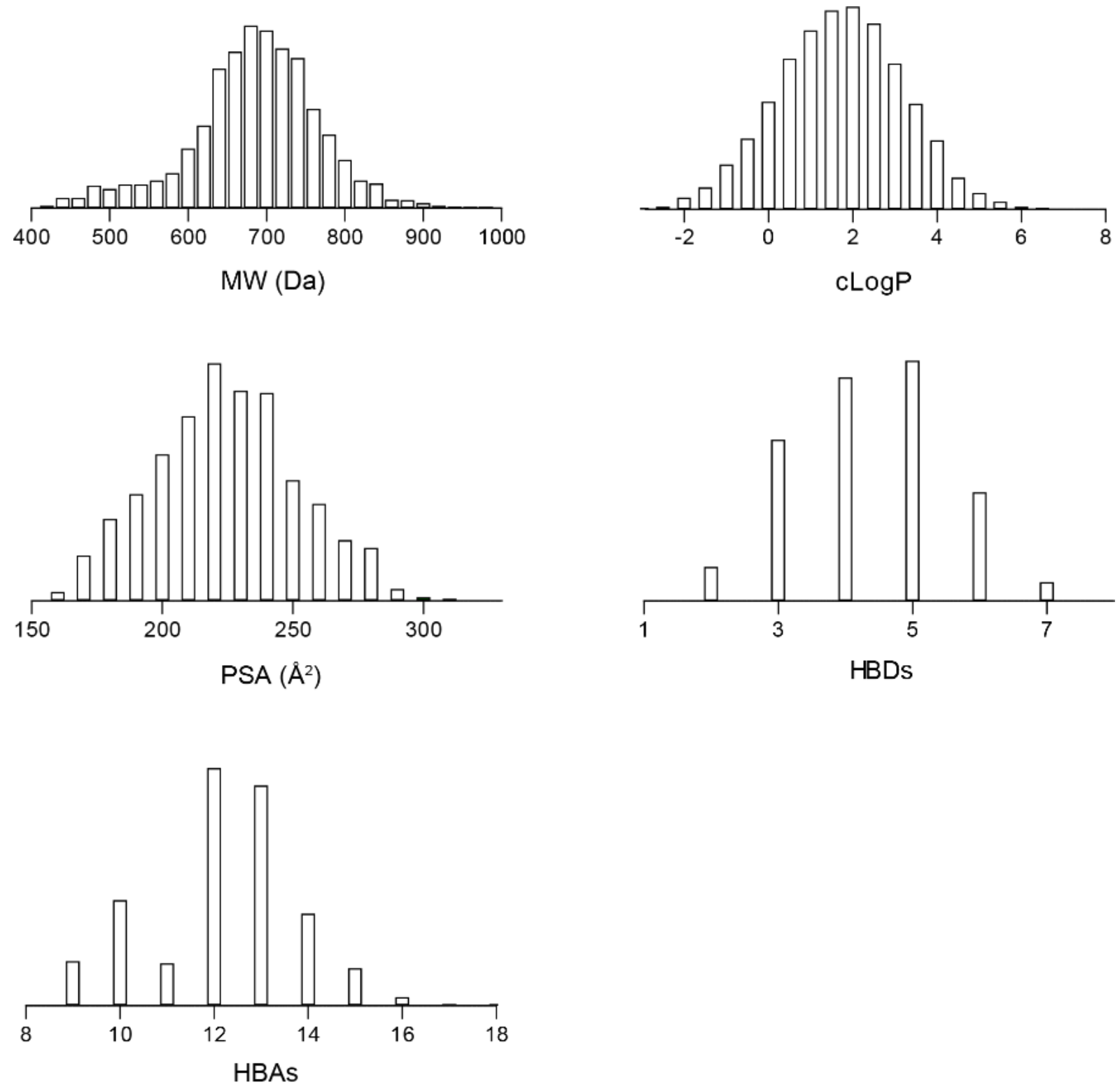

Supplementary Fig. 1. Physico-chemical properties of cyclic peptides of Library 1. DataWarrior software was used to calculate the physico-chemical properties. MW represents the molecular weight, cLogP the calculated lipophilicity (octanol/water partition coefficient), TPSA the polar surface area, HBA the number of hydrogen bond acceptors, and HBD the number of hydrogen bond donors.

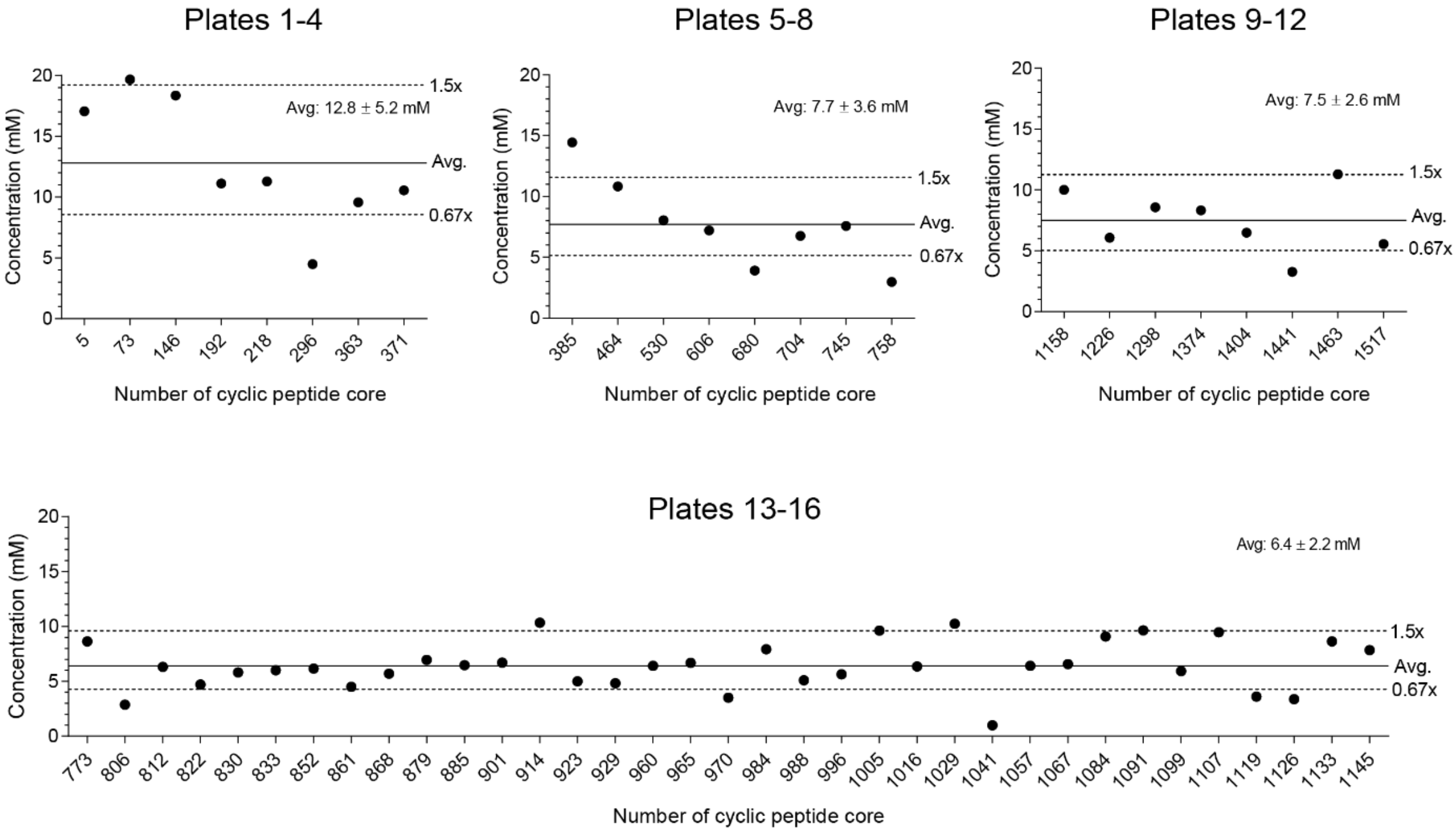

Supplementary Fig. 2. Concentration of cyclic peptide cores of Library 1. The concentrations of cyclic peptides were determined by measuring absorbance at 280nm. From the 96-well plates 1-4 (1st synthesis run), 5-8 (2nd synthesis run) and 9-12 (3rd synthesis run), all peptides containing a Trp were analyzed. From plates 13-16 (4th synthesis run), that contain many peptides with Trp, 35 peptides were randomly picked for analysis.

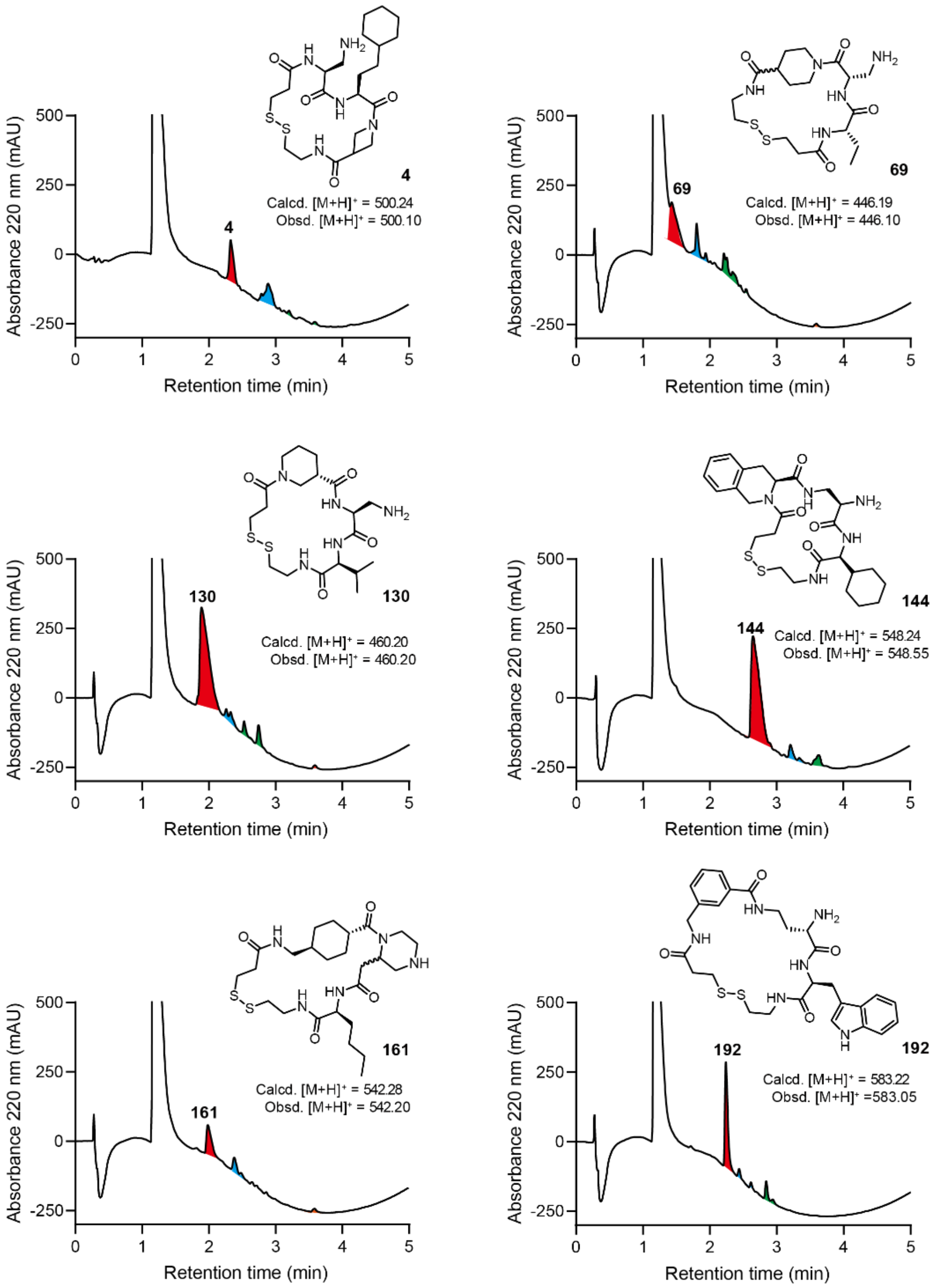

Supplementary Fig. 3. Quality of cyclic peptide cores of Library 1. The presence and purity of cyclic peptides were assessed by LC-MS analysis. Twelve peptides were randomly picked from each set of four 96-well plates synthesized in one run. Peaks of the desired cyclic peptide are highlighted in red. Dimeric peptides are shown in blue, *t*-Bu-modified peptides in green, and non-identified peptide species in orange.

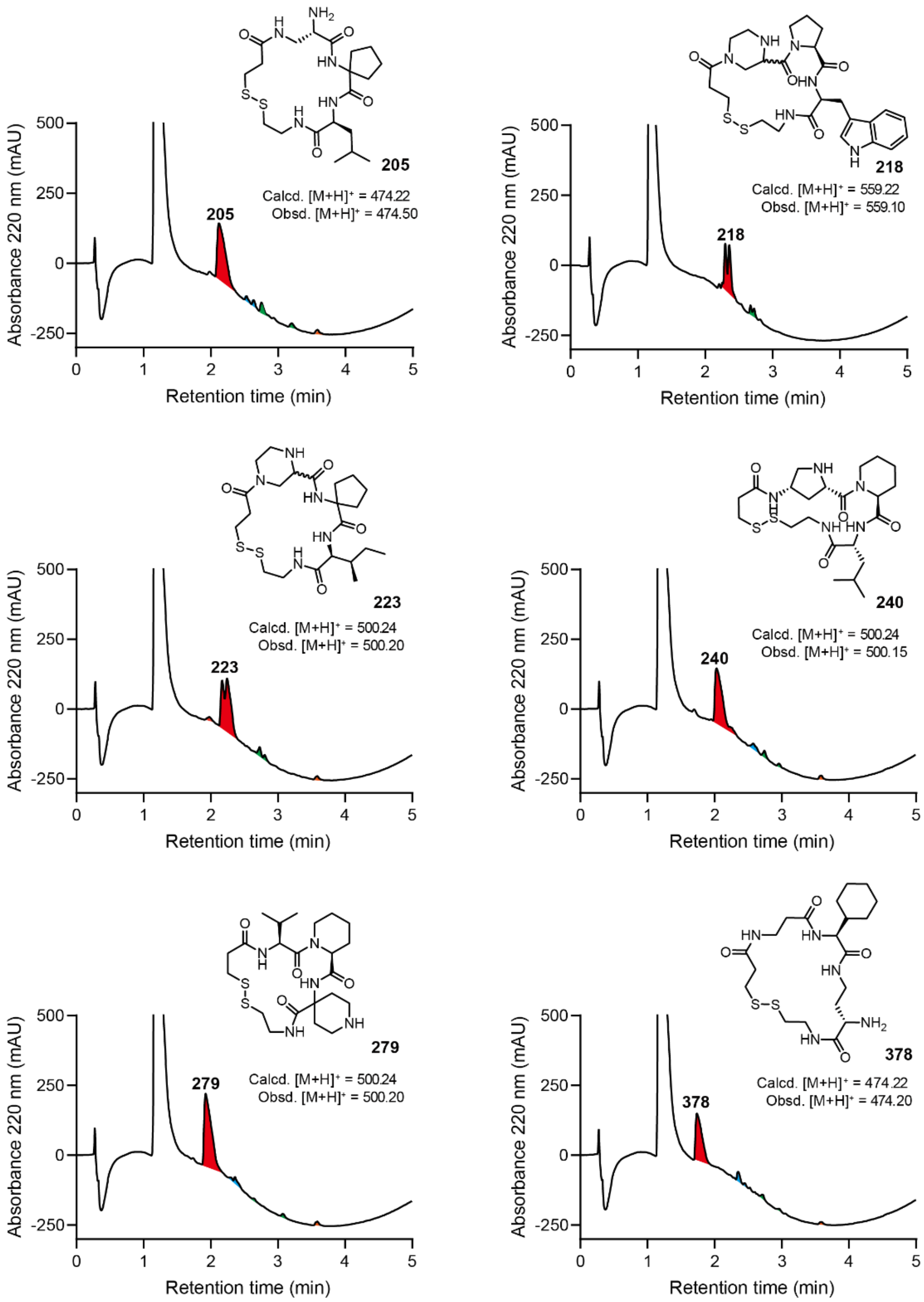

Supplementary Fig. 3. Continued

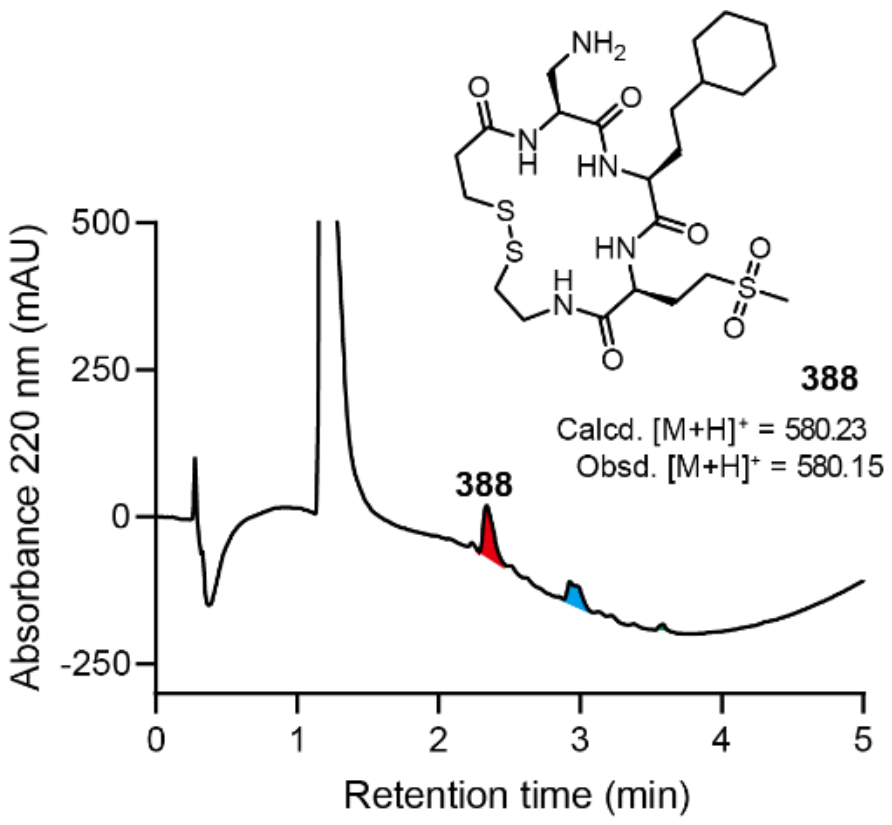

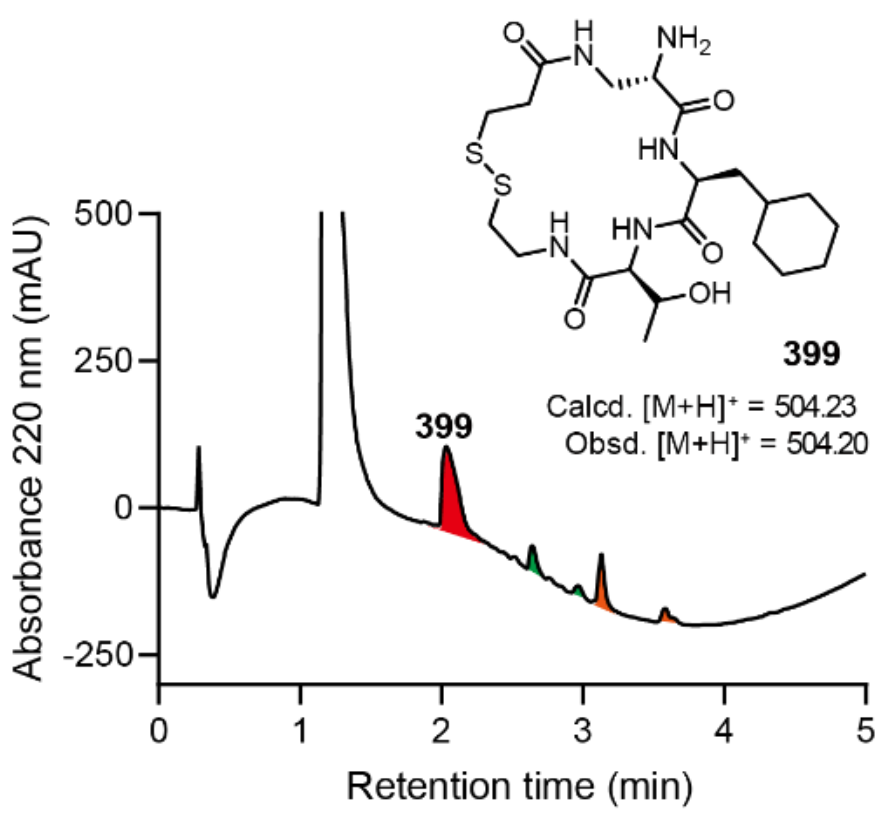

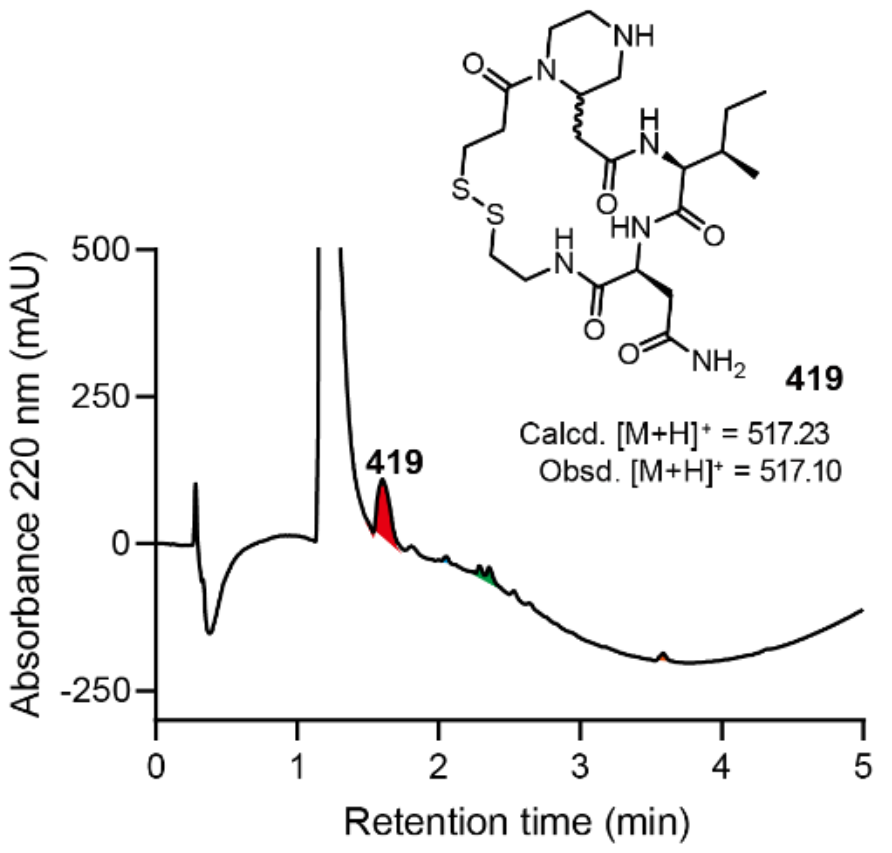

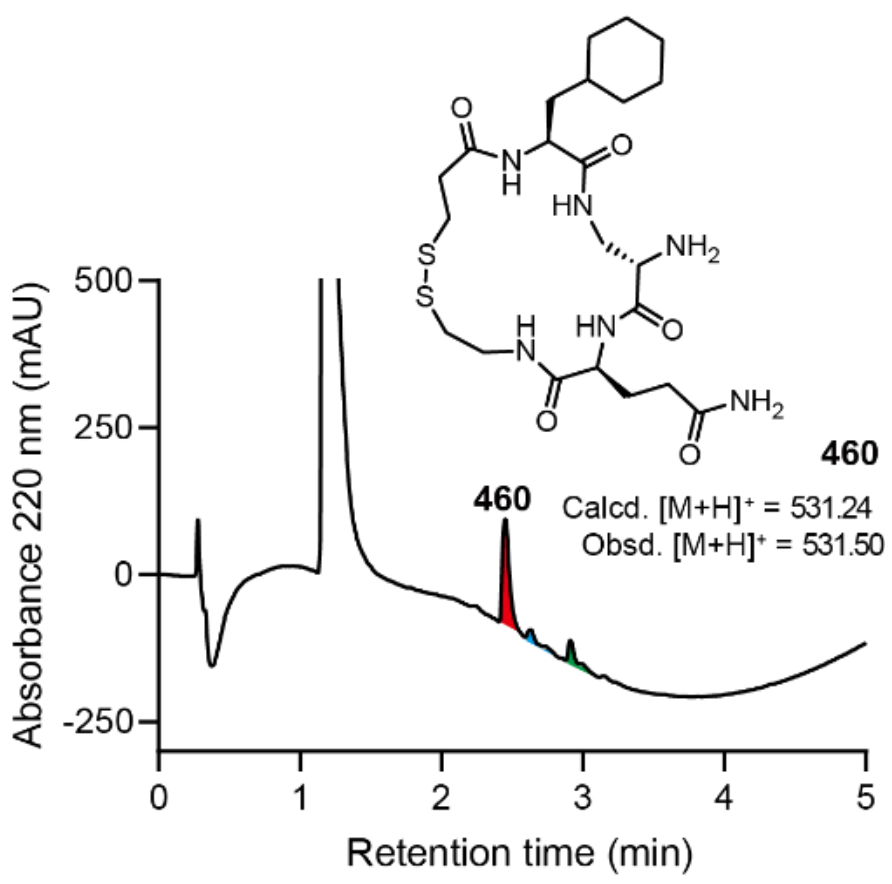

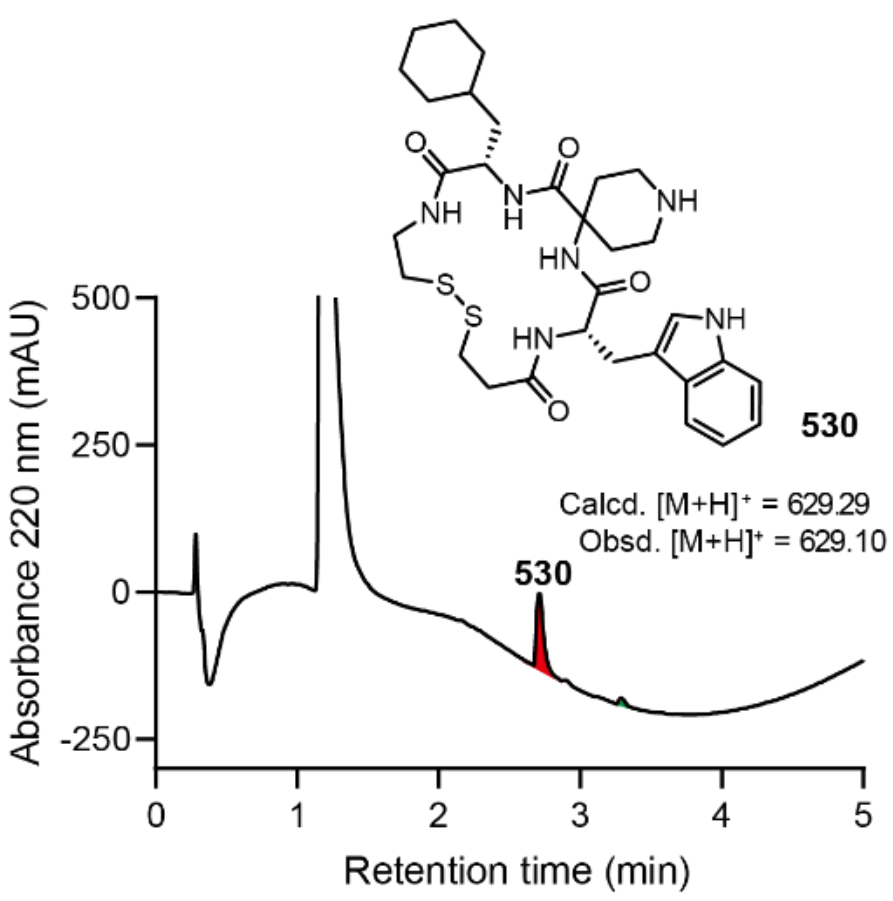

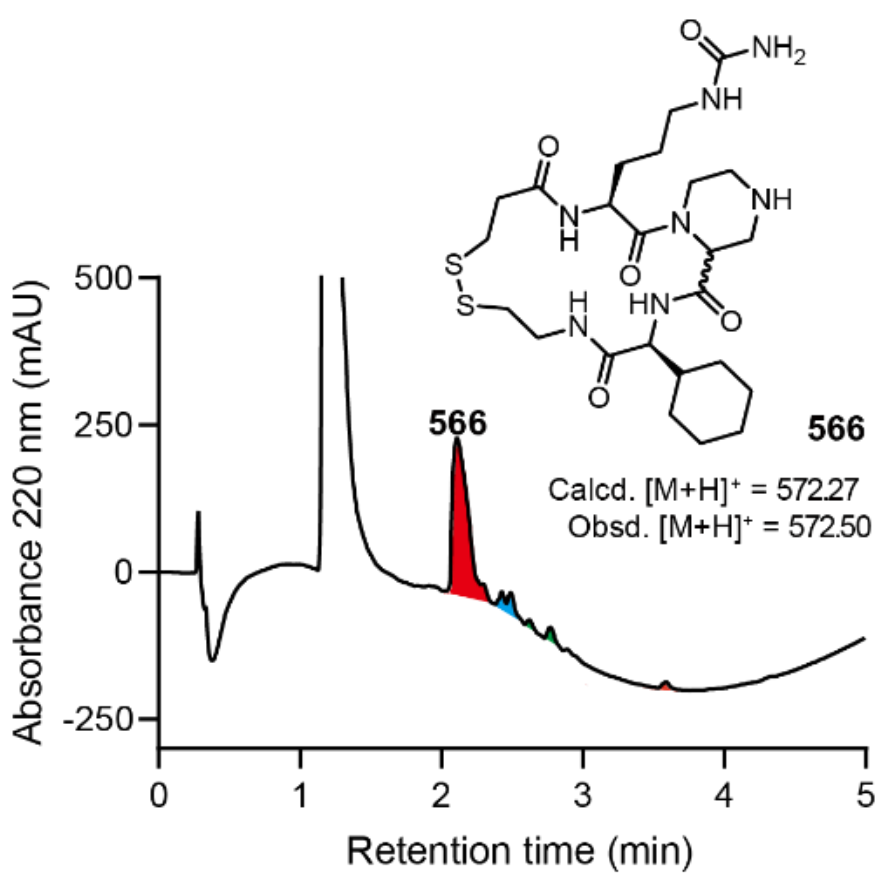

Supplementary Fig. 3. Continued

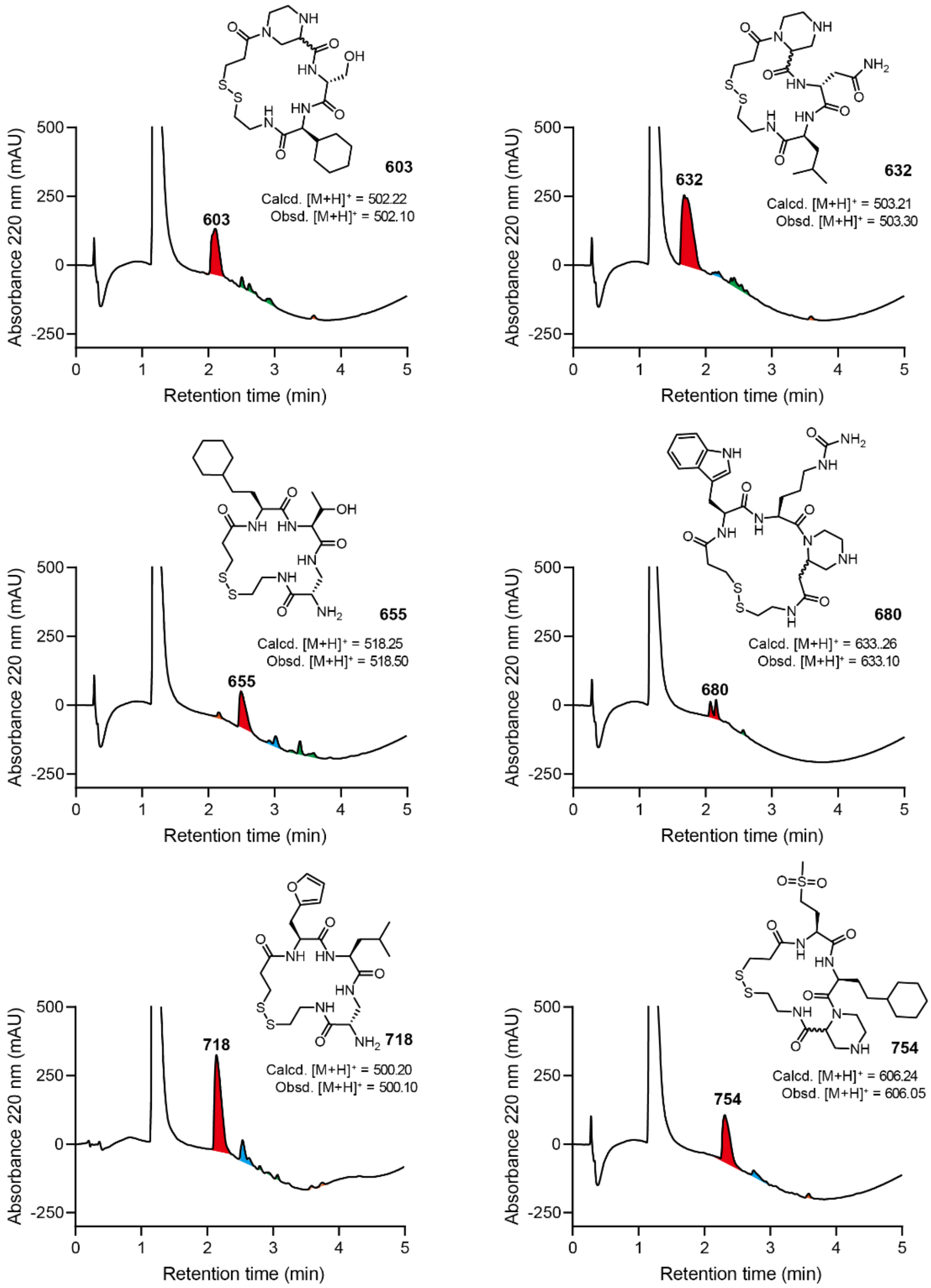

Supplementary Fig. 3. Continued

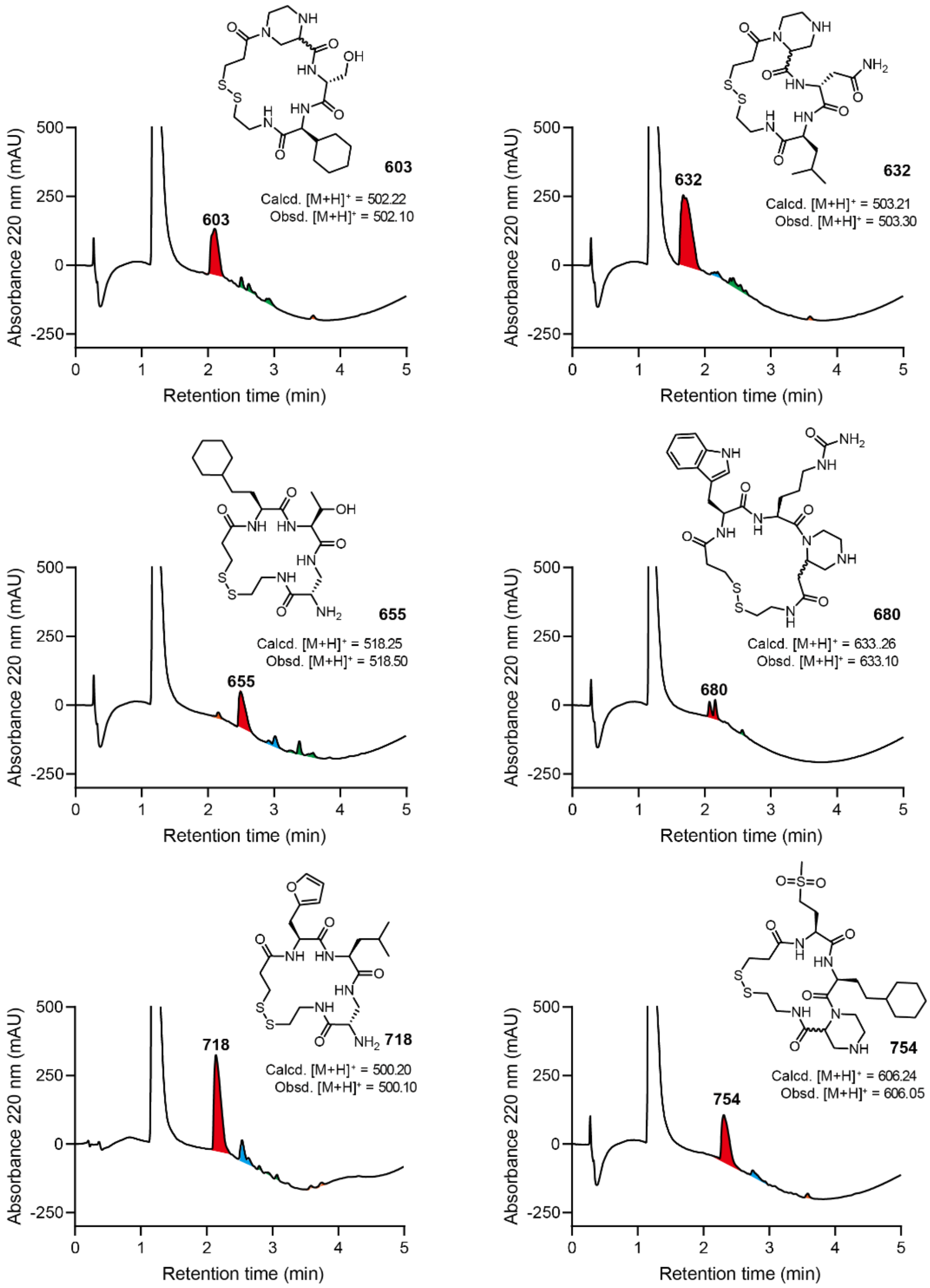

Supplementary Fig. 3. Continued

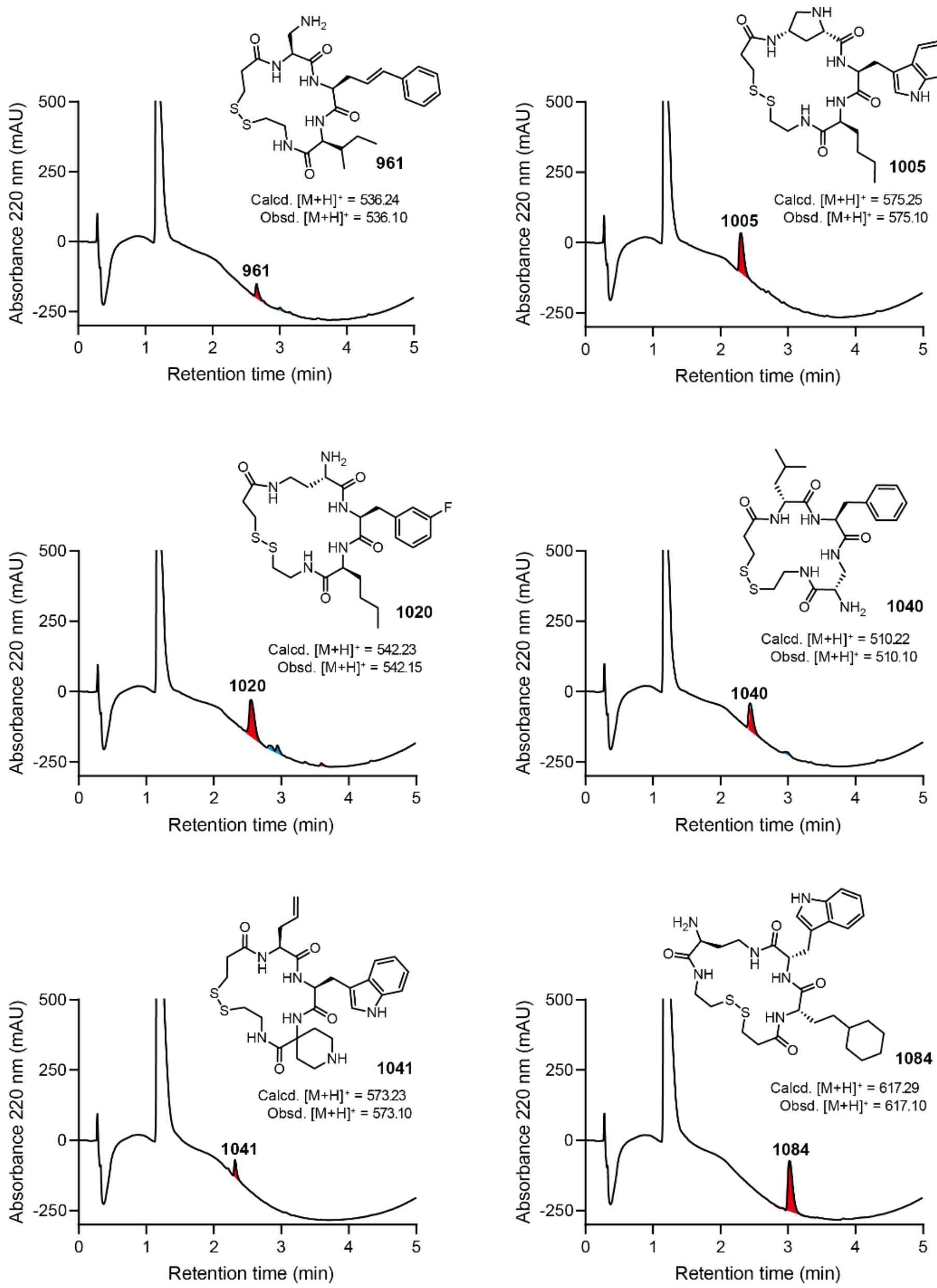

Supplementary Fig. 3. Continued

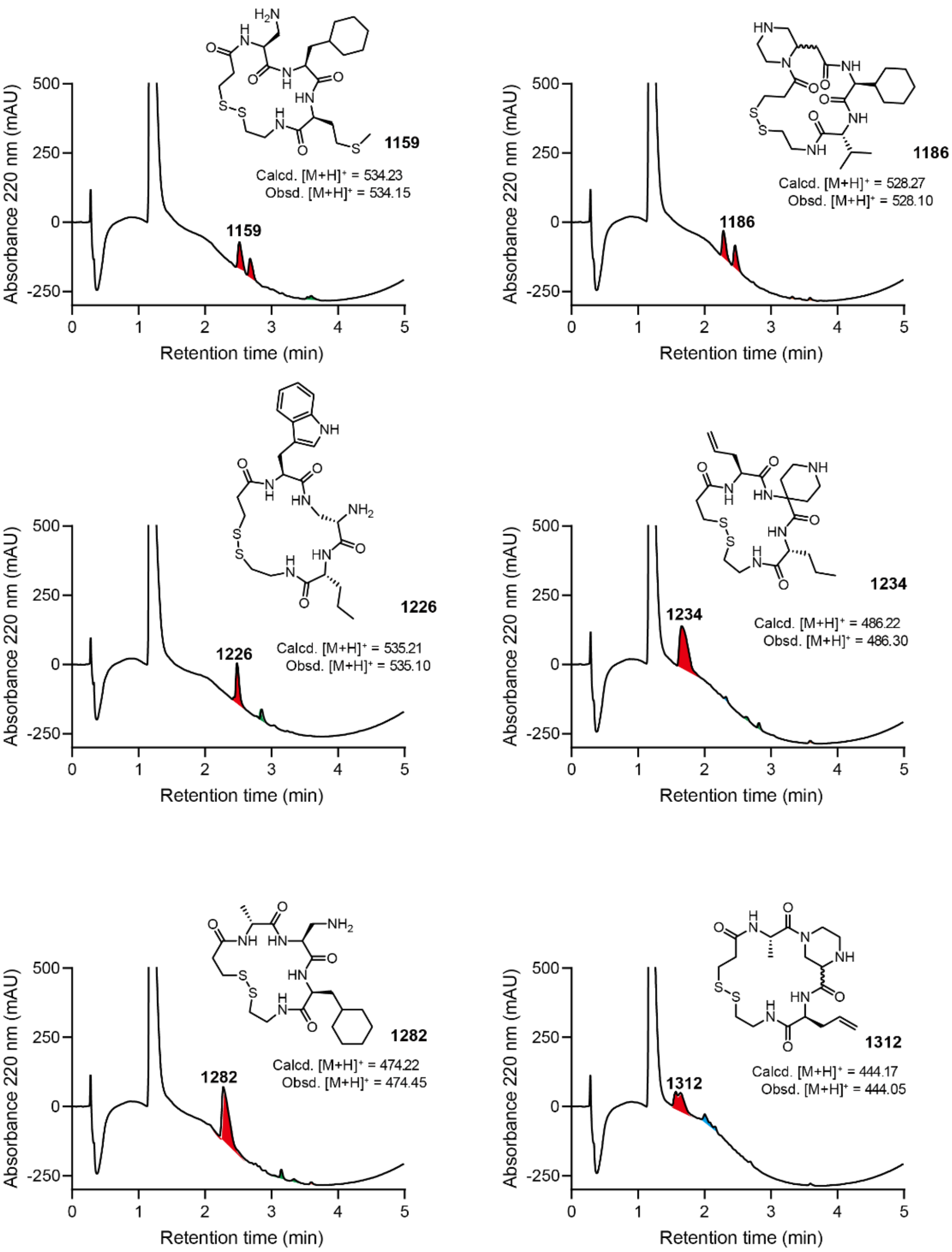

Supplementary Fig. 3. Continued

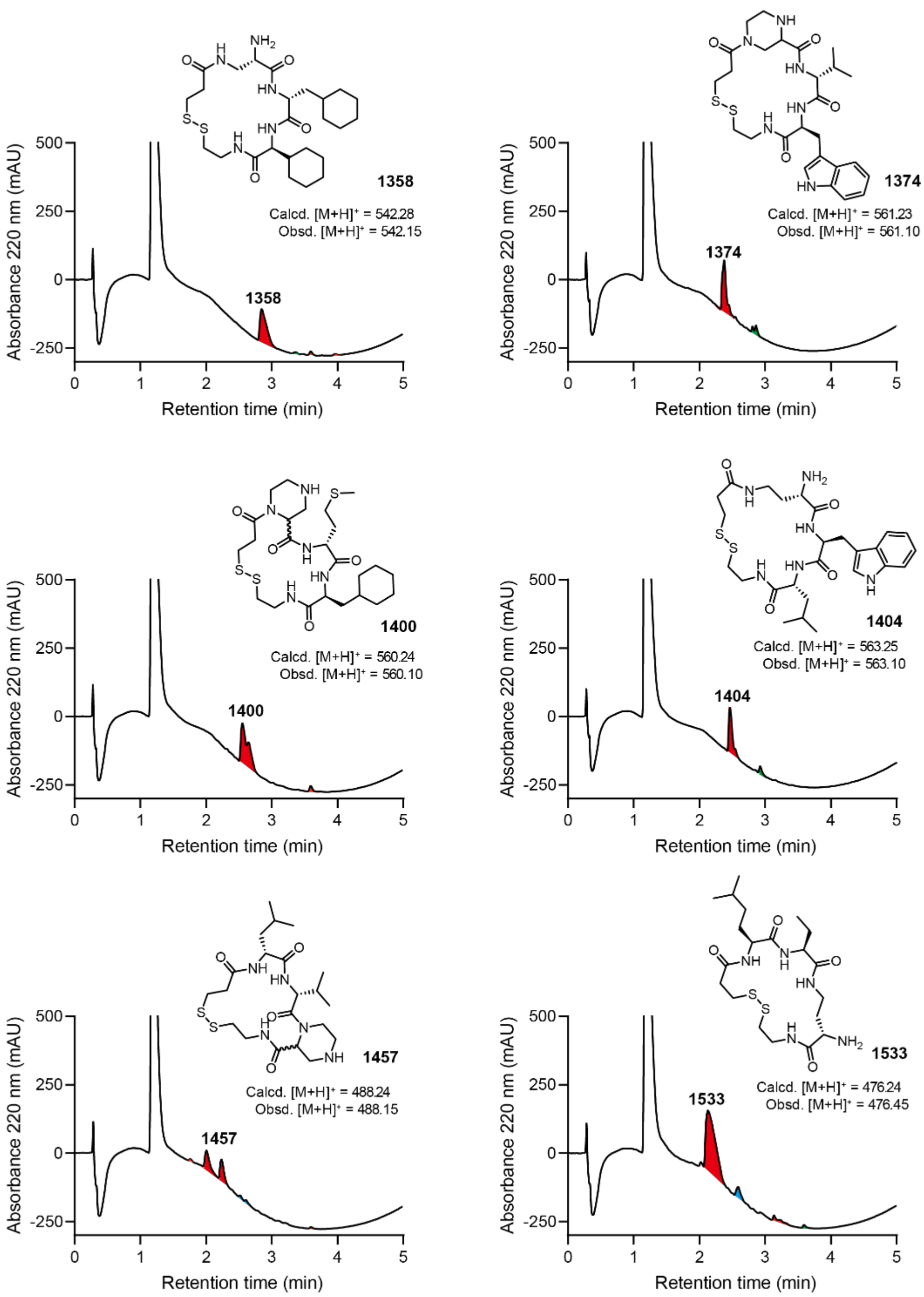

Supplementary Fig. 3. Continued

a

Protein sequence:

```
                                                  GSSHHHHHH SSGENLYFQS
321
APKVGRLIYT AGGYFRQSLS YLEAYNPSDG TWLRLADLQV PRSGLAGCVV
GGLLYAVGGR NNSPDGNTDS SALDCYNPMT NQWSPCAPMS VPRNRIGVGV
IDGHIYAVGG SHGCIHHNSV ERYEPERDEW HLVAPMLTRR IGVGVAVLNR
LLYAVGGFDG TNRLNSAECY YPERNEWRMI TAMNTIRSGA GVCVLHNCIY
AAGGYDGQDQ LNSVERYDVE TETWTFVAPM KHRRSALGIT VHQGRIYVLG
                                                    609
GYDGHTFLDS VECYDPDTDT WSEVTRMTSG RSGVGVAVT
```

b

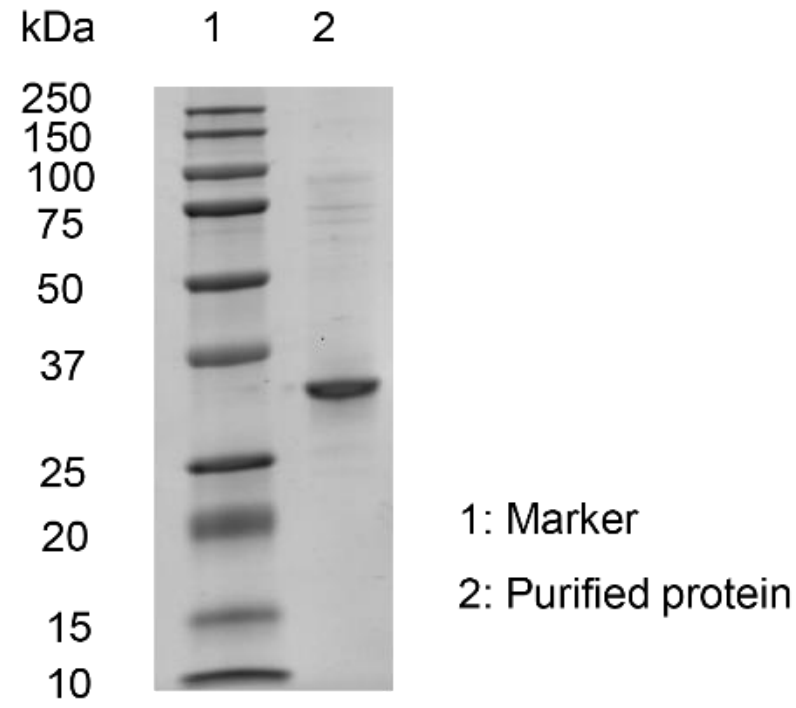

Supplementary Fig. 4. Keap1 cloning and expression for TR-FRET HTS assay. a, Amino acid sequence of the Kelch domain of human Keap1 protein. Amino acids appended at the N-terminus containing His-tag are shown in italics, the TEV cleavage site is underlined, and the Kelch domain of Keap1 (amino acid 321-609) is in bold. **b,** SDS-PAGE analysis of the purified Keap1 protein.

a

TR-FRET signal ratio

0 6,000 12,000

Conc. Nfr2-peptide (nM)	160	80	40	20	10	5	0	
	10,472	7,187	4,342	2,540	1,889	1,105	106	**80**
	11,571	10,225	6,784	4,295	3,253	2,599	107	**40**
	6,308	10,188	9,373	6,865	5,430	6,232	107	**20**
	5,687	9,595	8,321	6,922	6,276	3,692	104	**10**
	4,813	7,097	5,350	4,283	3,924	4,724	101	**5**
	4,573	7,554	4,190	3,108	2,910	2,716	105	**2.5**
	138	119	112	119	112	108	106	**0**
								**Conc. Keap1 (nM)**

b

Signal to background ratio

0 50 100

Conc. Nfr2-peptide (nM)	160	80	40	20	10	5	0	
	75.82	60.15	38.60	21.35	16.83	10.27	1.01	**80**
	83.78	85.58	60.31	36.10	28.98	24.15	1.01	**40**
	45.67	85.27	83.32	57.71	48.37	57.92	1.01	**20**
	41.17	80.31	73.97	58.19	55.91	34.31	0.98	**10**
	34.85	59.40	47.56	36.00	34.96	43.91	0.96	**5**
	33.11	63.23	37.24	26.13	25.93	25.24	0.99	**2.5**
	1.00	1.00	1.00	1.00	1.00	1.00	1.00	**0**
								**Conc. Keap1 (nM)**

Supplementary Fig. 5. Establishment of TR-FRET-based Keap1-Nrf2 inhibition assay. a, TR-FRET signal ratio (Cy5 emission / Eu emission) for different concentration combinations of His-tagged Keap1 and Cy5-labeled Nrf2 peptide. Values of the TR-FRET signal ratio are indicated after multiplication by 10,000. **b,** Factor by which the TR-FRET signal ratio (Cy5 emission / Eu emission) changes upon addition of His-tagged Keap1 protein. These values were calculated by dividing the TR-FRET signal ratio (Cy5 emission / Eu emission) value obtained in presence of Keap1 by the TR-FRET signal ratio (Cy5 emission / Eu emission) value obtained in absence of Keap1. The condition indicated by the red arrow was selected for the Keap1–Nrf2 inhibition screen. This condition achieved the highest possible signal-to-background ratio while ensuring a moderate consumption of protein.

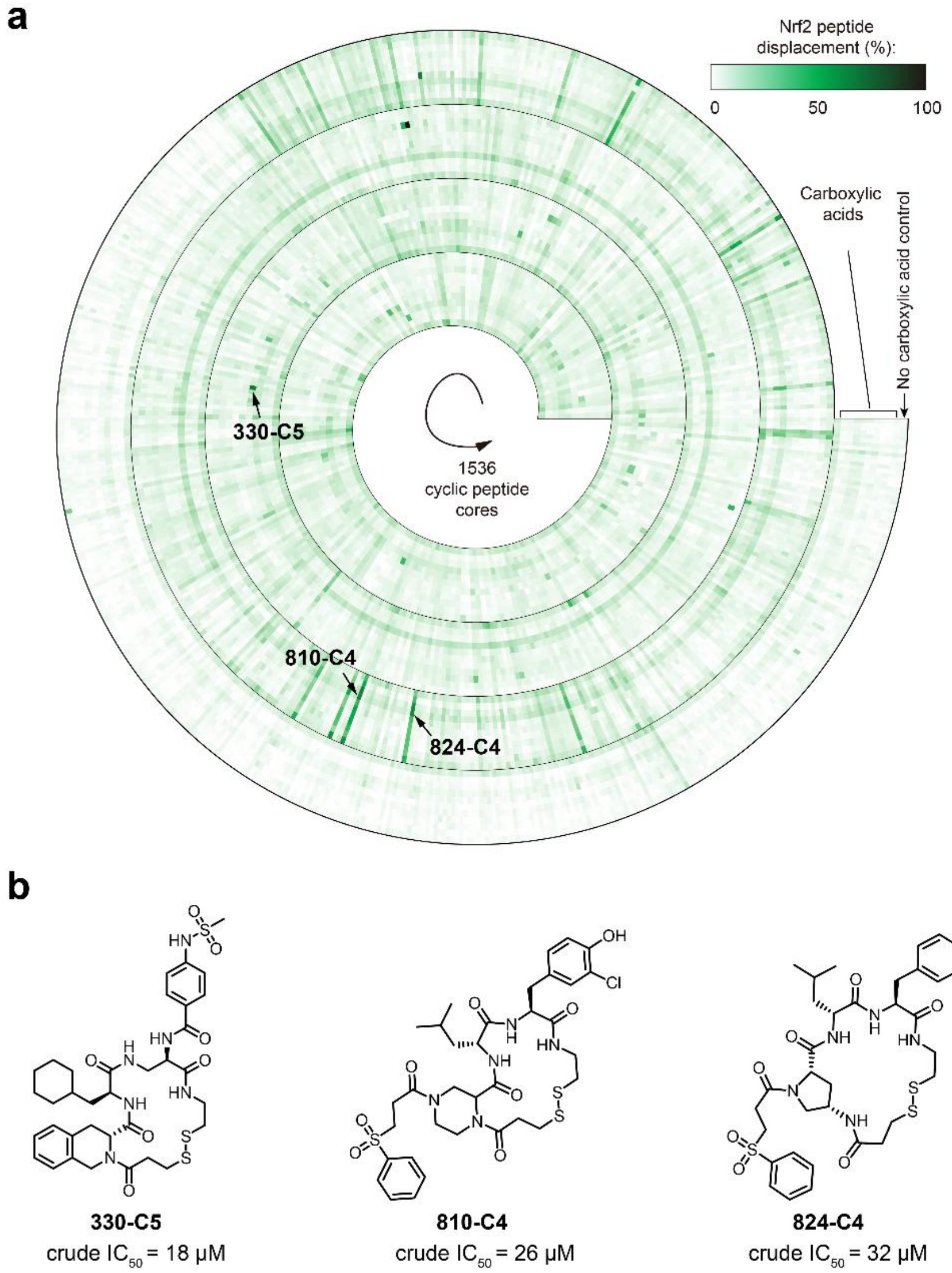

Supplementary Fig. 6. Additional hits of HTS. a, Result of HTS screen with Library 1 as shown in Figure 2c, with three peptides indicated that were tested in a confirmation screen in addition to hit compounds **1** and **2**. **b,** Chemical structures of the three cyclic peptides and their IC_{50} assessed with crude peptide.

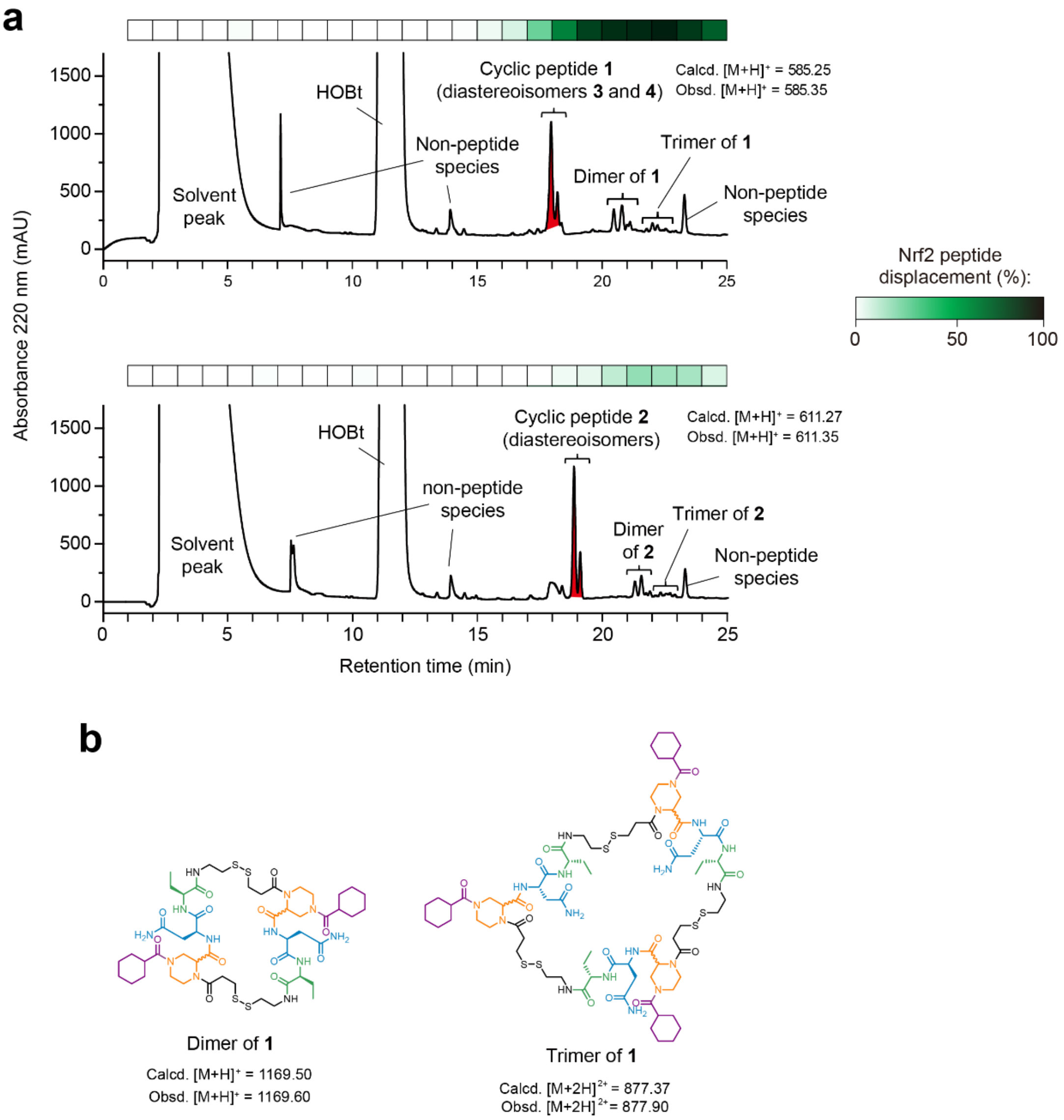

Supplementary Fig. 7. Identification of active species in crude reaction of hits 1 and 2. **a,** The acylation reactions of peptides cyclo(D4-X13-A1) and cyclo(D4-X19-A3) with carboxylic acid C9 were repeated using the same conditions as for the Library 1 synthesis but at a larger scale (64 nmol), the products and side-products separated by reversed-phase HPLC collecting fractions for one minute, and the activity of all fractions measured using the TR-FRET Keap1–Nrf2 peptide displacement assay. While the fractions of the monocyclic peptides showed some activity, substantially higher activities were observed for dimeric and trimeric species of the acylated peptides **1** and **2**. **b,** Chemical structures of disulfide-cyclized dimer and trimer of peptide **1**. The peptides can be connected in different orientations to form dimers and trimers, and only one example for each multimer is shown.

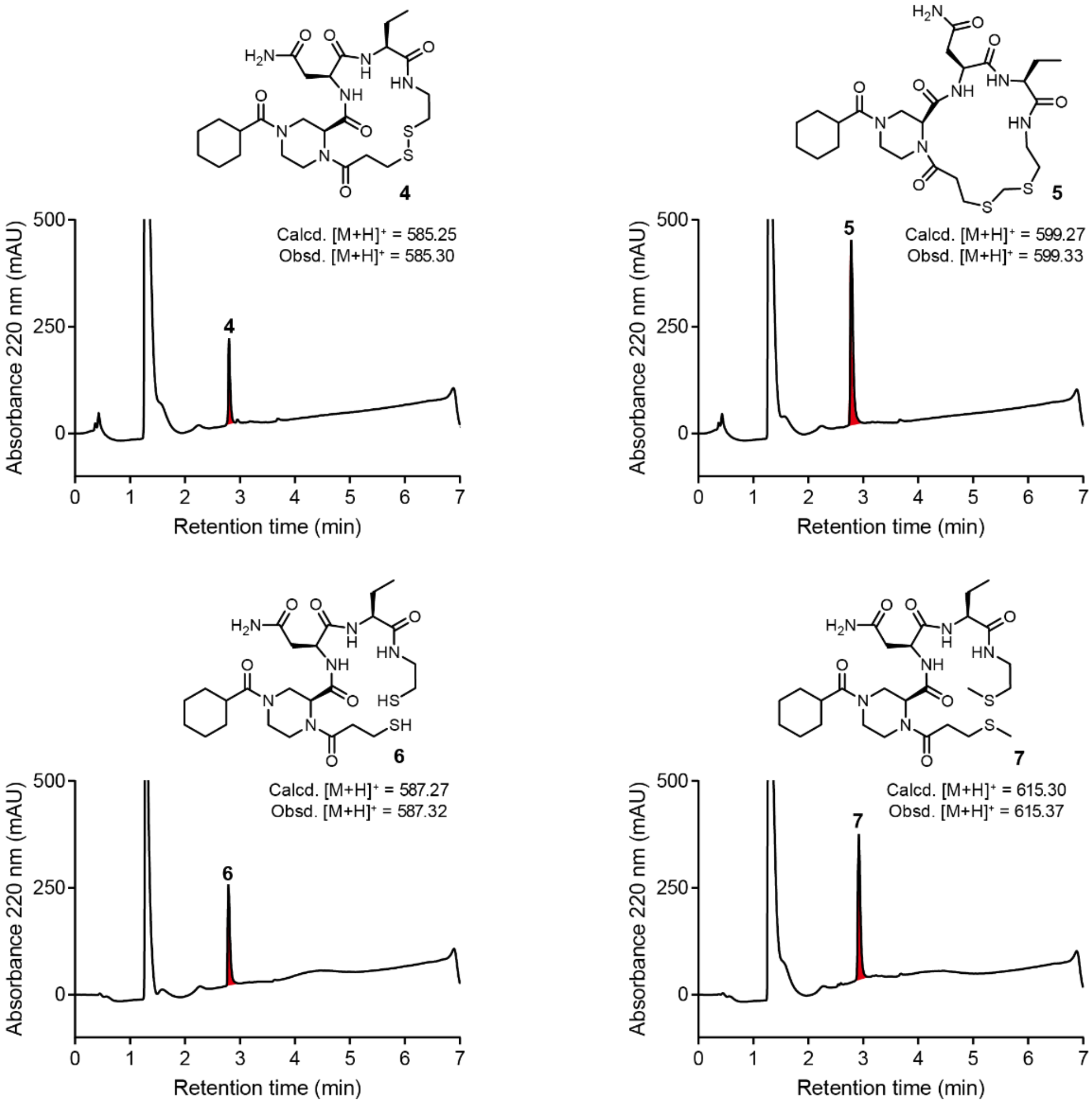

Supplementary Fig. 8. Purity and identity of HPLC-purified peptide 4 and analogs. Chemical structures of the peptides are shown. The purity and identity of the peptides were assessed by LC-MS.

a

Protein sequence:

GSSHHHHHHH SSG<u>ENLYFQS</u>

321

APKVGRLIYT AGGYFRQSLS YLEAYNPSDG TWLRLADLQV PRSGLAGCVV
GGLLYAVGGR NNSPDGNTDS SALDCYNPMT NQWSPCAPMS VPRNRIGVGV
IDGHIYAVGG SHGCIHHNSV ERYEPERDEW HLVAPMLTRR IGVGVAVLNR
LLYAVGGFDG TNRLNSAECY YPERNEWRMI TAMNTIRSGA GVCVLHNCIY
AAGGYDGQDQ LNSVERYDVE TETWTFVAPM KHRRSALGIT VHQGRIYVLG
GYDGHTFLDS VECYDPDTDT WSEVTRMTSG RSGVGVAVT

609

b

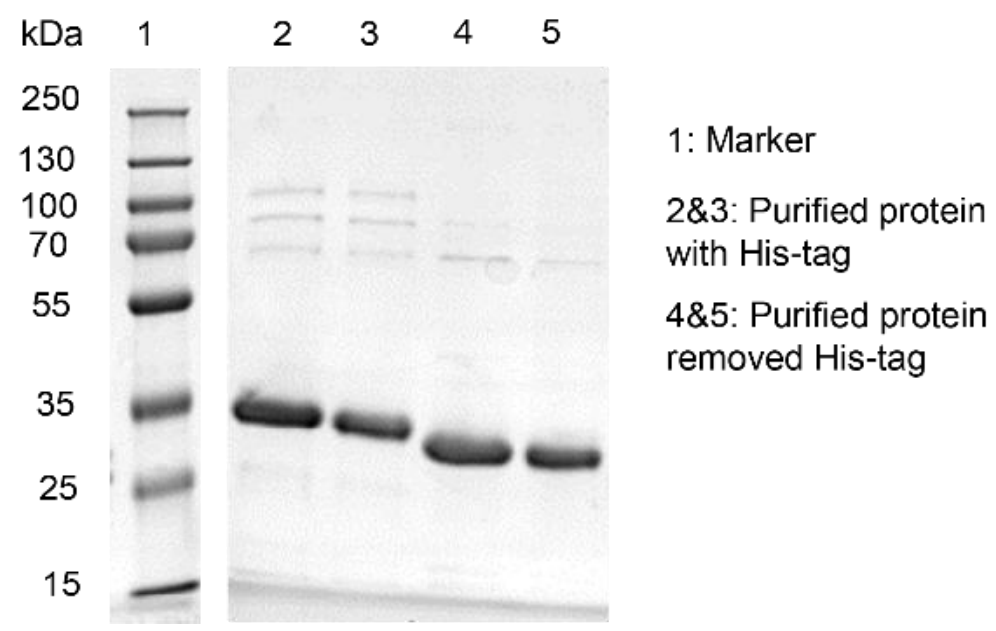

Supplementary Fig. 9. Keap1 cloning and expression for structure determination by X-ray crystallography. a, Amino acid sequence of the Kelch domain of the Keap1 protein with the mutations E540A and E542A. Amino acids appended at the N-terminus containing His-tag are shown in italics, the TEV cleavage site is underlined, and the Kelch domain of Keap1 (amino acid 321-609) is in bold. The two mutation sites are highlighted in red. **b,** SDS-PAGE analysis of the purified Keap1 protein. For crystallization screening, Keap1 protein without His-tag was obtained through TEV protease cleavage.

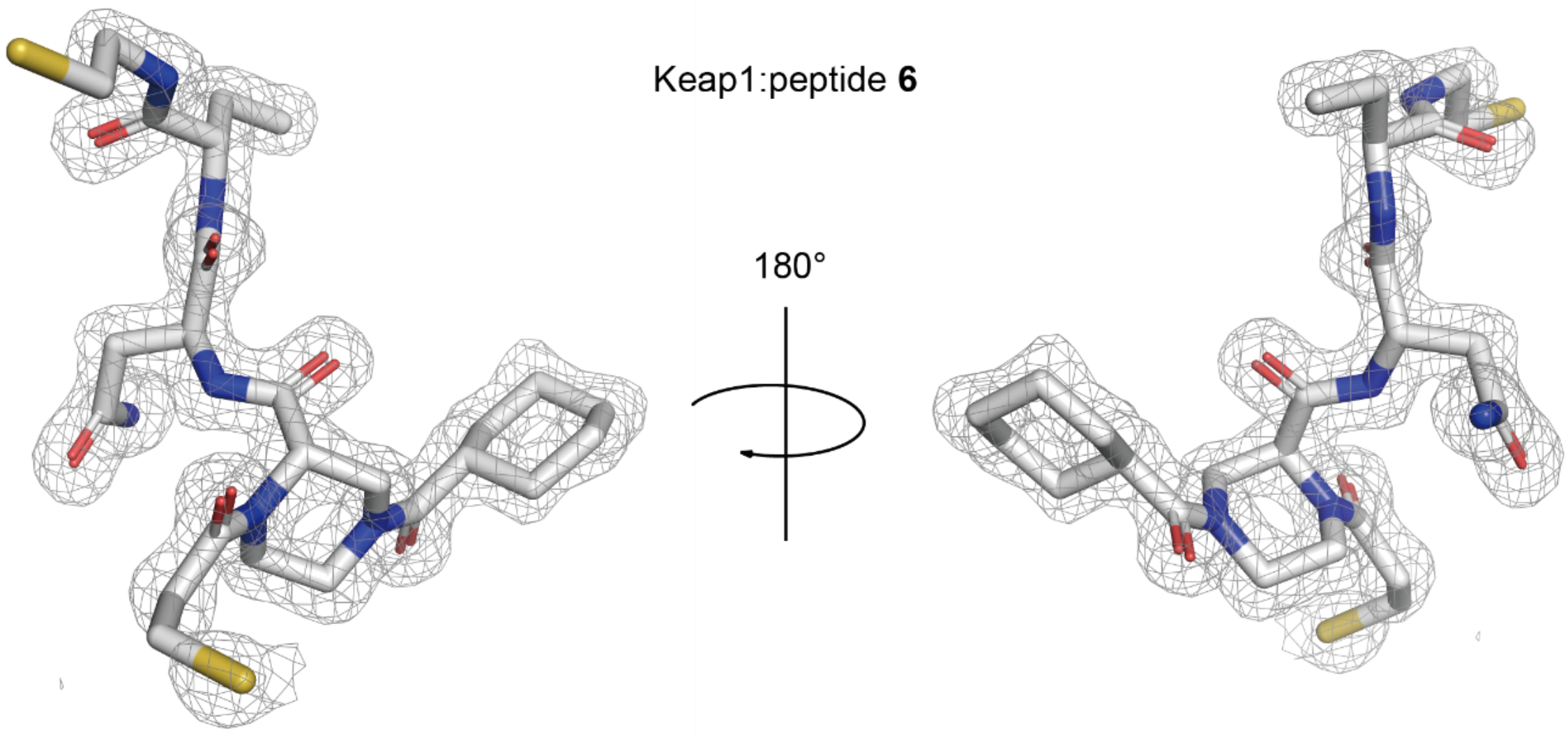

Supplementary Fig. 10. X-ray structure of peptide 6 bound to Keap1. The 2Fo-Fc electron-density map contoured at 1.5 σ (gray mesh) is superimposed.

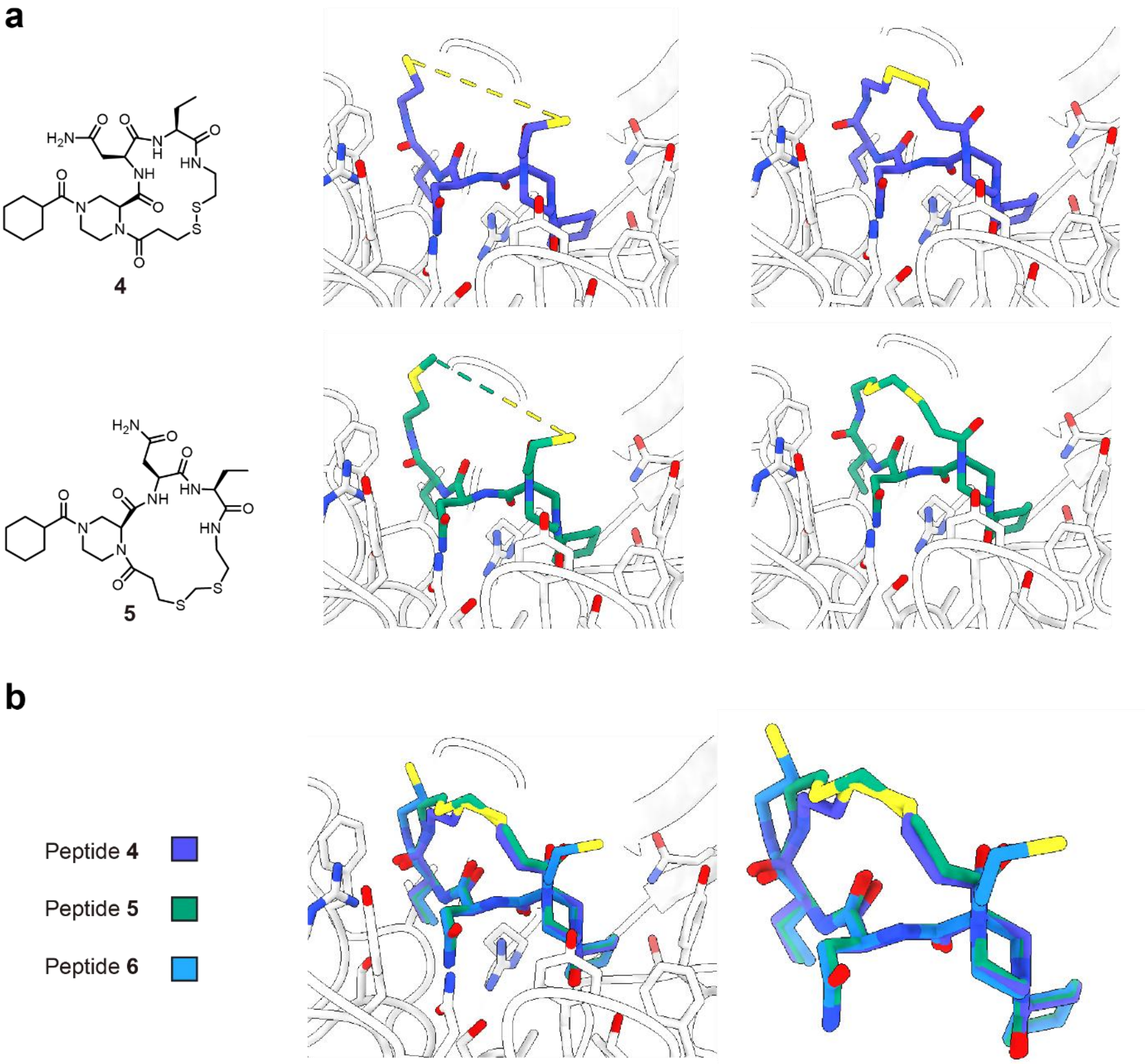

Supplementary Fig. 11. Models of cyclic peptides based on linear peptide 6. a, Starting from the crystal structure of the linear peptide **6** (PDB ID: 9IH9), the corresponding C–C and disulfide bonds in cyclic peptides **4** and **5** were manually constructed in Maestro (version 13.7.125) to achieve peptide cyclization. The artificially cyclized intermediates, which initially violated standard chemical and physical constraints, were subsequently subjected to energy minimization to yield chemically and physically reasonable cyclic conformations. The optimized cyclic peptide–protein complex models were used for figure preparation. **b,** Superimposition of peptide **4** (violet), **5** (green), and **6** (blue).

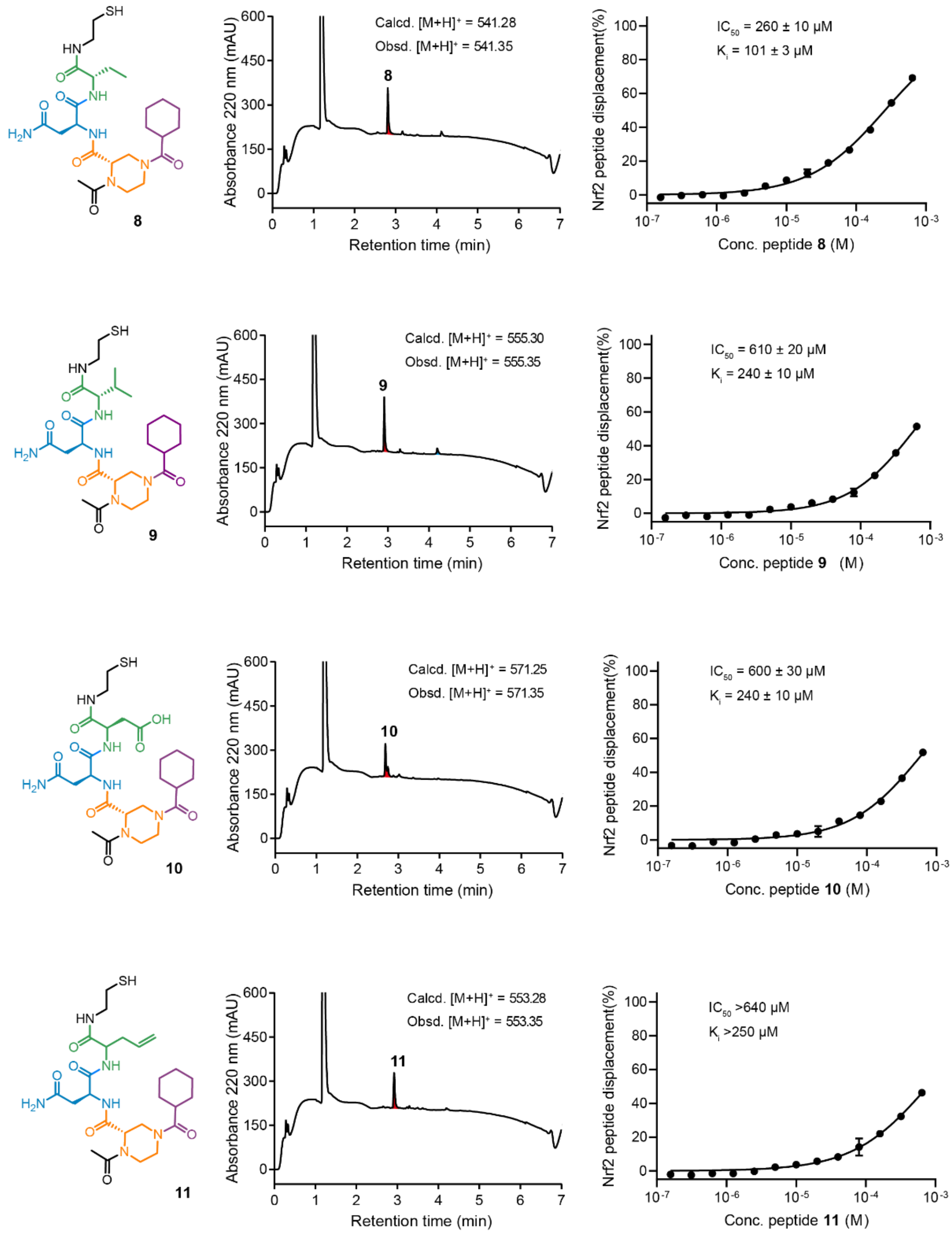

Supplementary Fig. 12. Structure and activity of peptides 8–18. Chemical structures of the peptides are shown, with the different building blocks highlighted in color. The purity and identity of the peptides were assessed by LC-MS. The activity of the peptides was measured using a competition fluorescence polarization assay under reducing conditions.

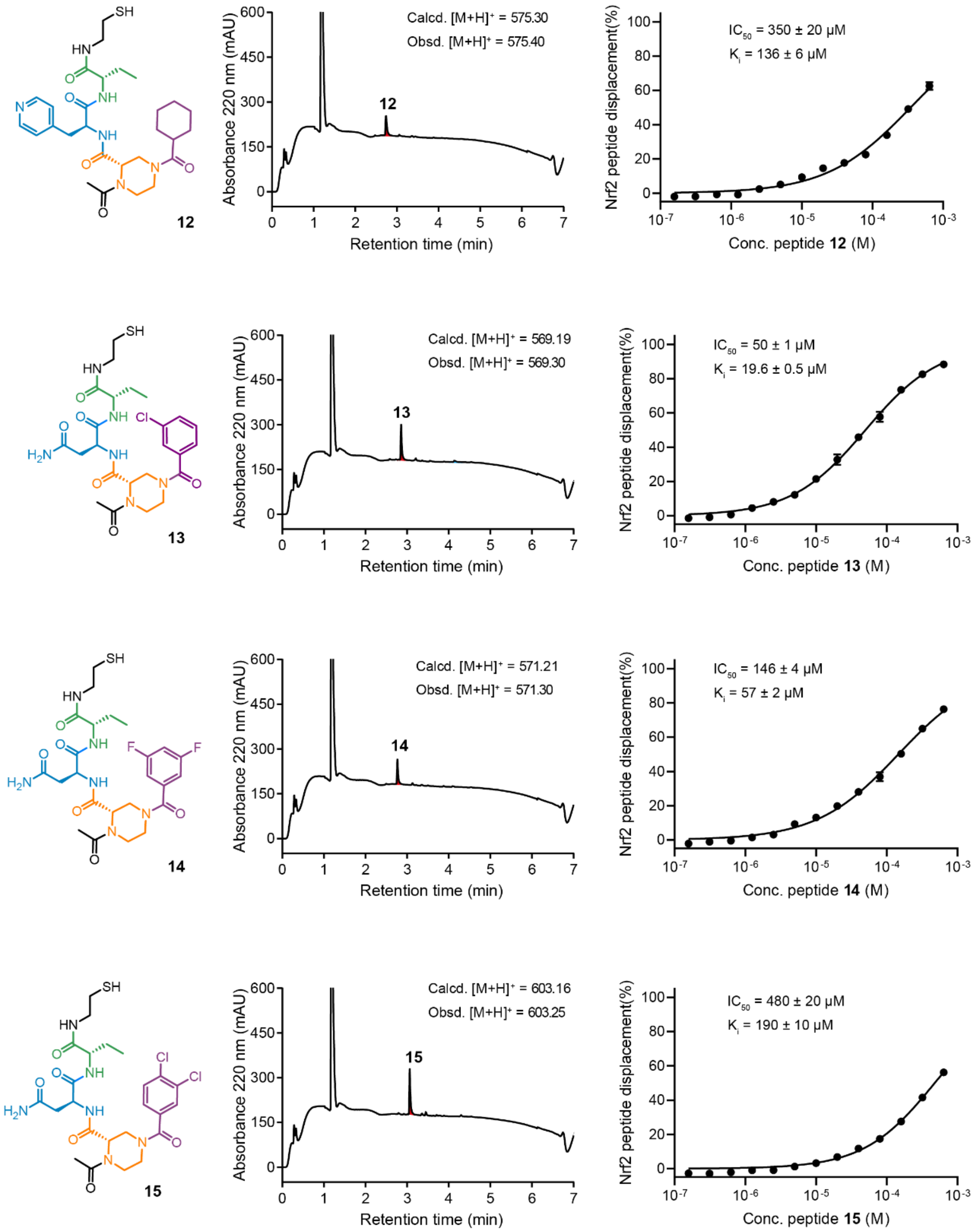

Supplementary Fig. 12. Continued

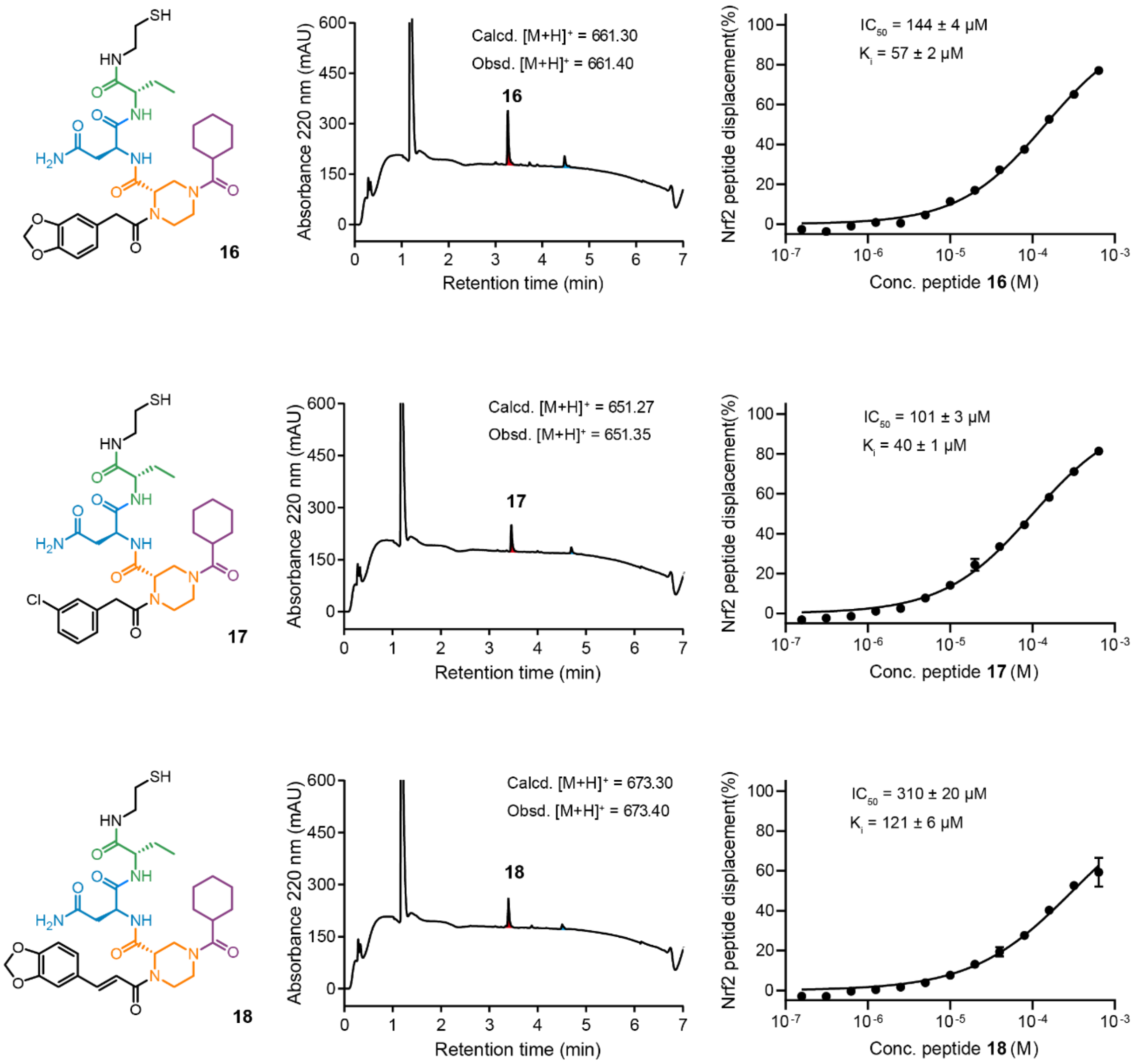

Supplementary Fig. 12. Continued

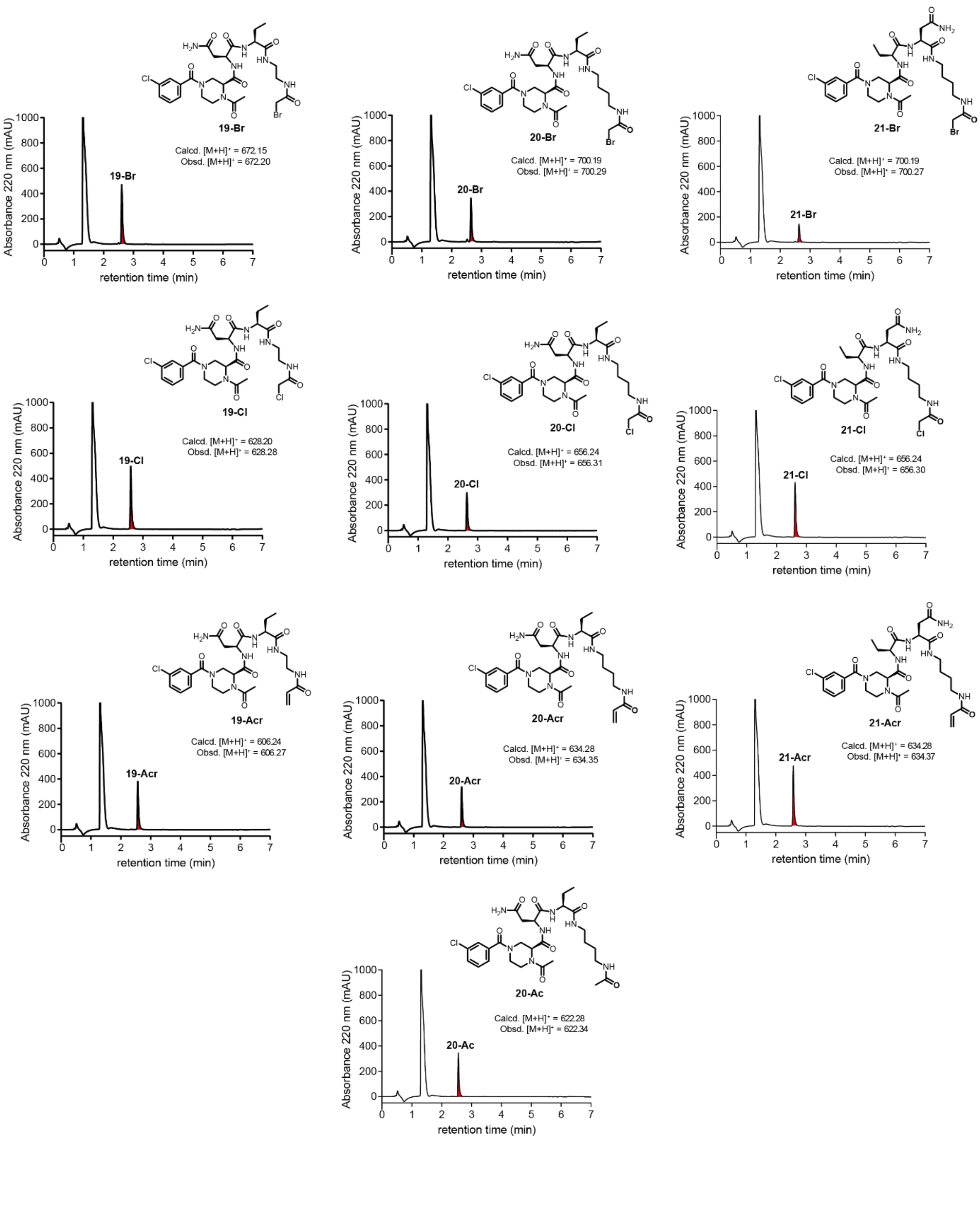

Supplementary Fig. 13. Purity and identity of peptides 19–21 containing warheads. Chemical structures of the peptides are shown. The purity and identity of the peptides were assessed by LC-MS.

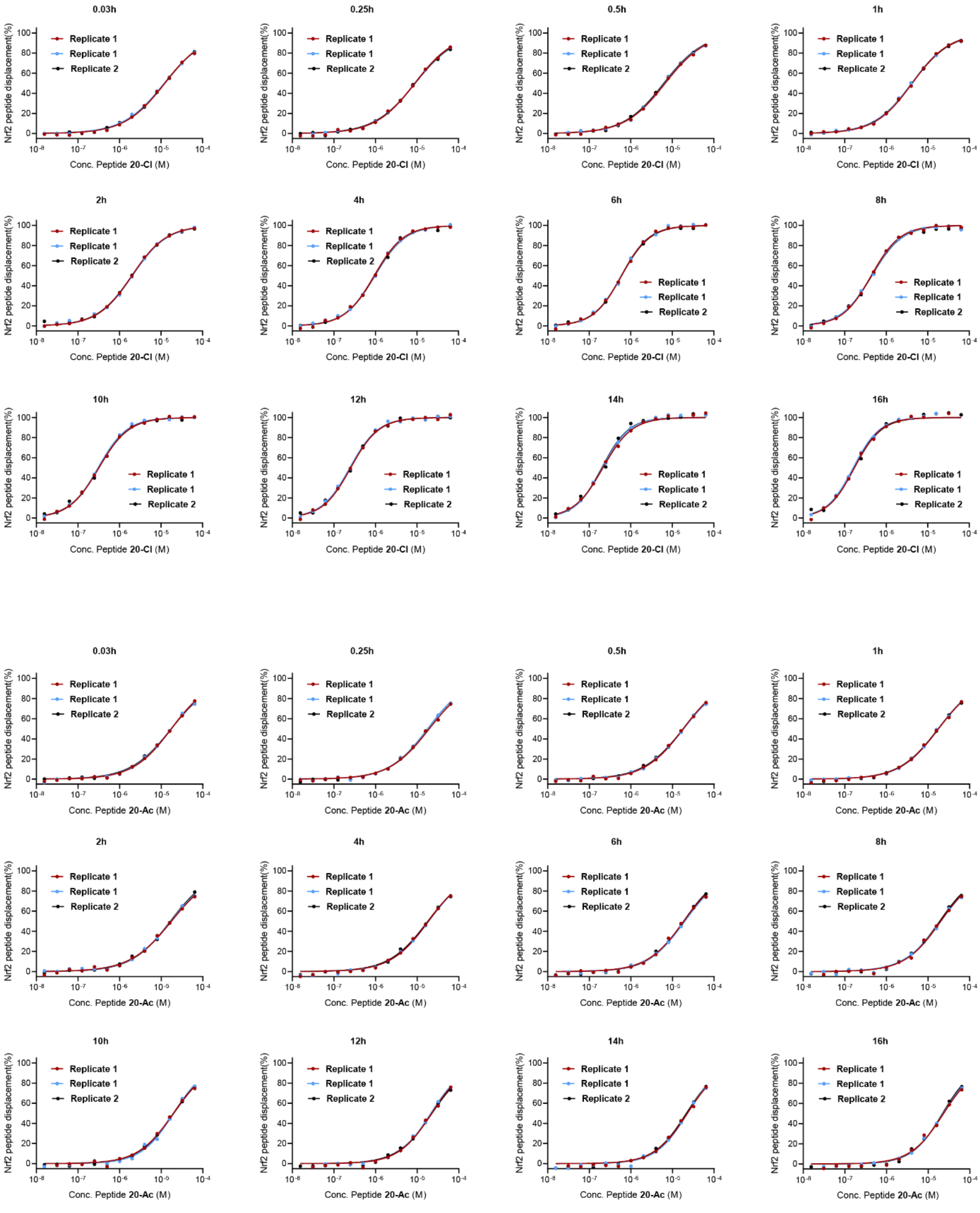

Supplementary Fig. 14. Inhibition of Keap1–Nrf2 by covalent inhibitor assessed after different incubation times. Keap1 was incubated with peptide **20-Cl** or the control **20-Ac** without warhead and the inhibition of the protein–protein interaction was measured in a

fluorescence polarization competition assay. For each peptide and time point, three measurements were performed (replicates performed in the same microwell plate).

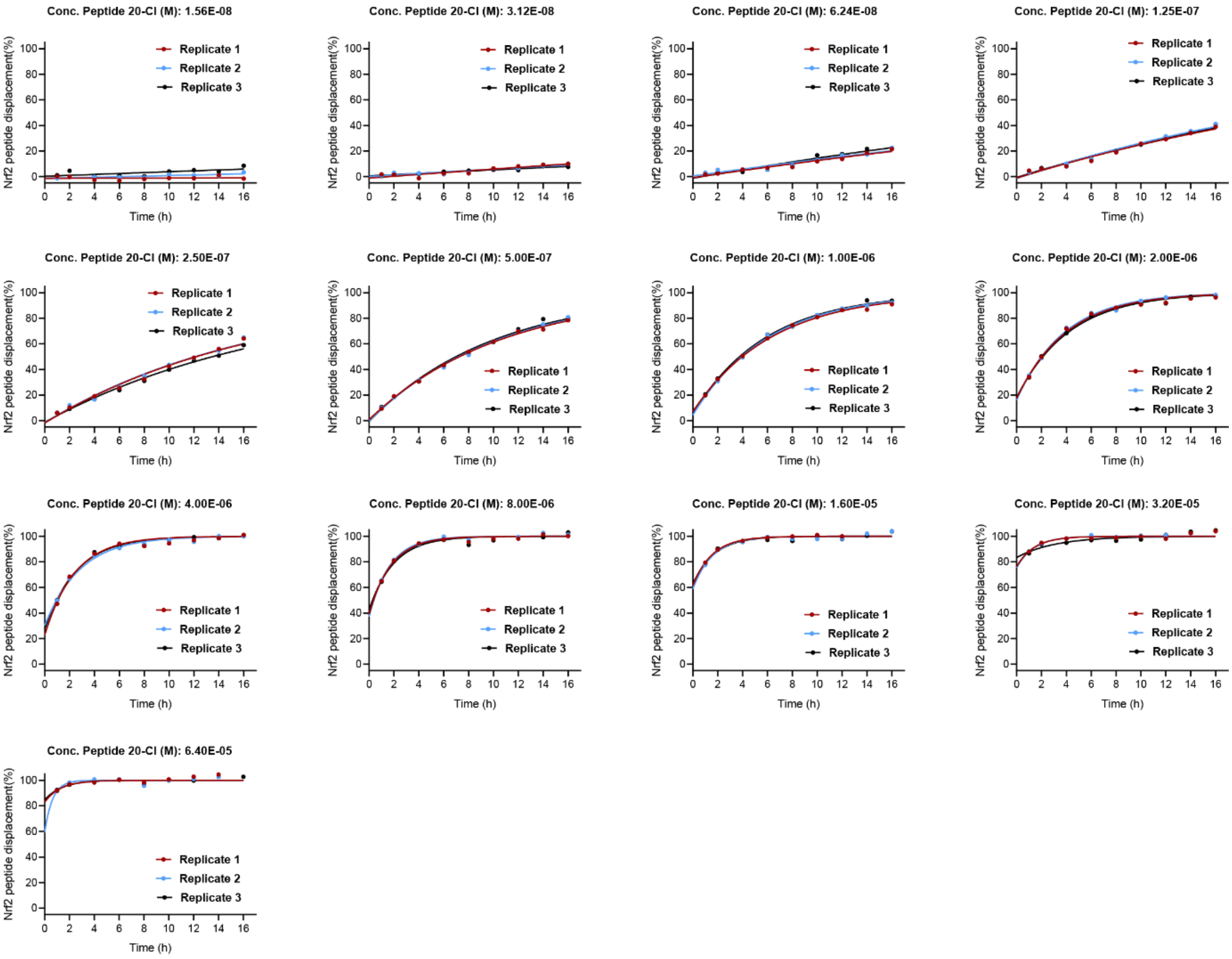

Supplementary Fig. 15. Determination of k_{obs} for covalent inhibitor peptide 20-Cl. Keap1 was incubated with different concentrations of peptide **20-Cl** and the inhibition of the protein–protein interaction was measured in a fluorescence polarization competition assay. For each inhibitor concentration and time point, three measurements were performed (replicates performed in the same microwell plate).

a

Protein sequence:

*GSSHHHHHH SSG*ENLYFQS

321
APKVGRLIYT AGGYFRQSLS YLEAYNPSDG TWLRLADLQV PRSGLAGCVV
GGLLYAVGGR NNSPDGNTDS SALDCYNPMT NQWSPCAPMS VPRNRIGVGV
IDGHIYAVGG SHGAIHHNSV ERYEPERDEW HLVAPMLTRR IGVGVAVLNR
LLYAVGGFDG TNRLNSAECY YPERNEWRMI TAMNTIRSGA GVCVLHNCIY
AAGGYDGQDQ LNSVERYDVE TETWTFVAPM KHRRSALGIT VHQGRIYVLG
GYDGHTFLDS VECYDPDTDT WSEVTRMTSG RSGVGVAVT 609

b

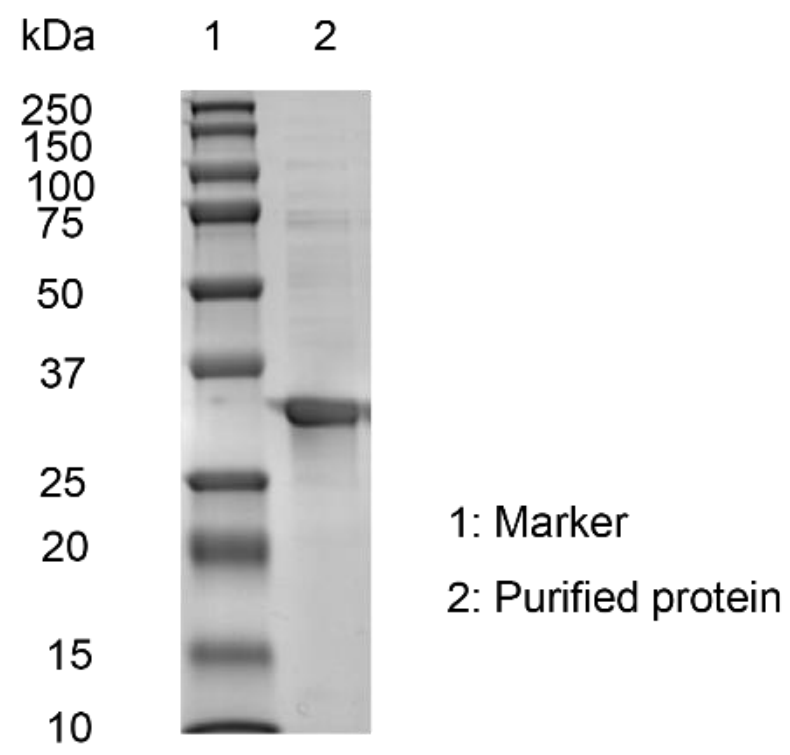

Supplementary Fig. 16. Keap1 cloning and expression for identification of covalently bound peptides by mass spectrometry. a, Amino acid sequence of the Keap1 protein with C434A mutation. Amino acids appended at the *N*-terminus containing His-tag are shown in italics, the TEV cleavage site is underlined, and the Kelch domain of Keap1 (amino acid 321-609) is in bold. The mutation site is highlighted in red. **b,** SDS-PAGE analysis of the purified Keap1 protein.

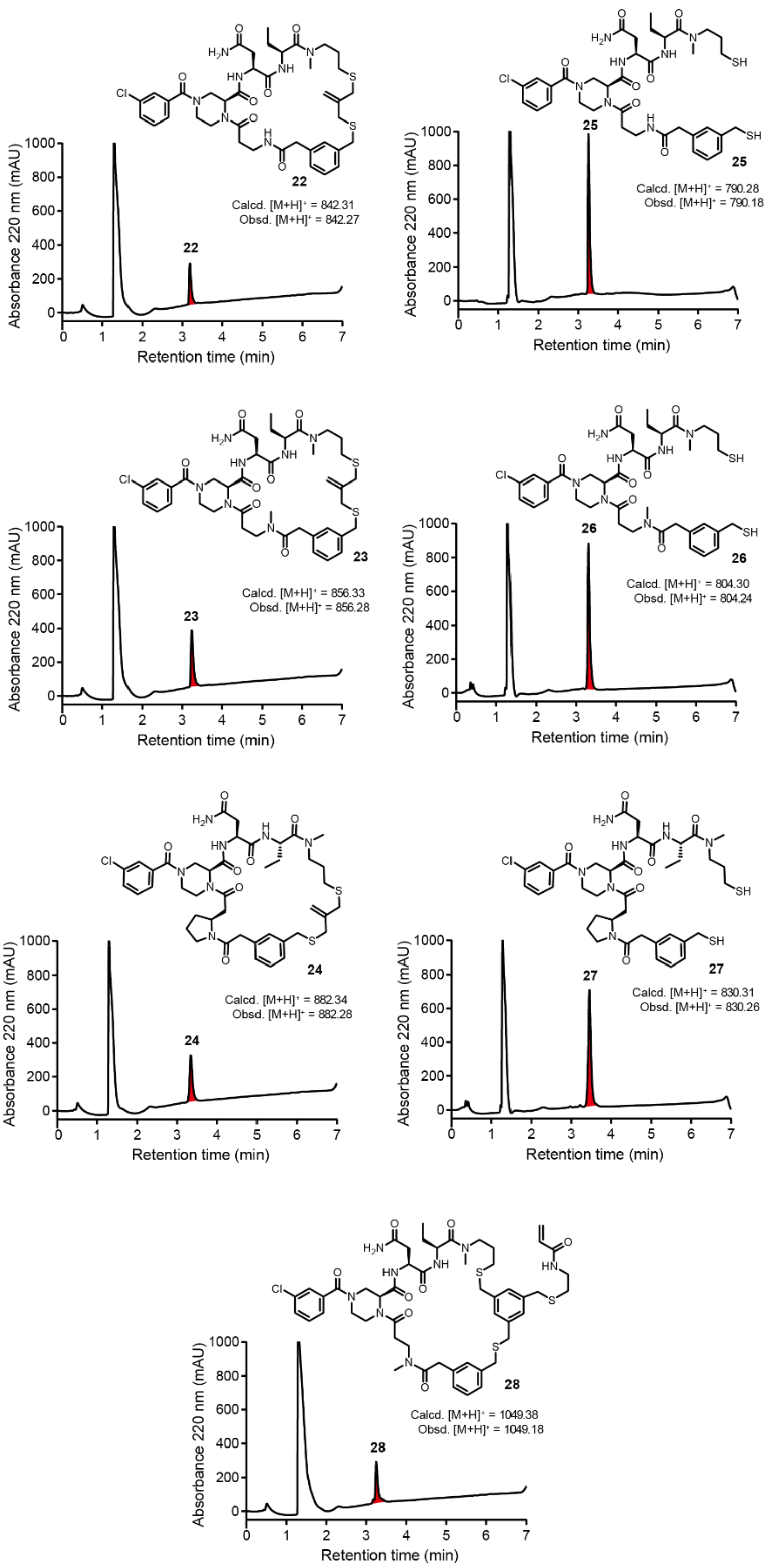

Supplementary Fig. 17. Purity and identity of cyclic peptides with optimized linkers (22–24), linear analogs (25–27), or an electrophilic warhead (28). Chemical structures of the peptides are shown. The purity and identity of the peptides were assessed by LC-MS.

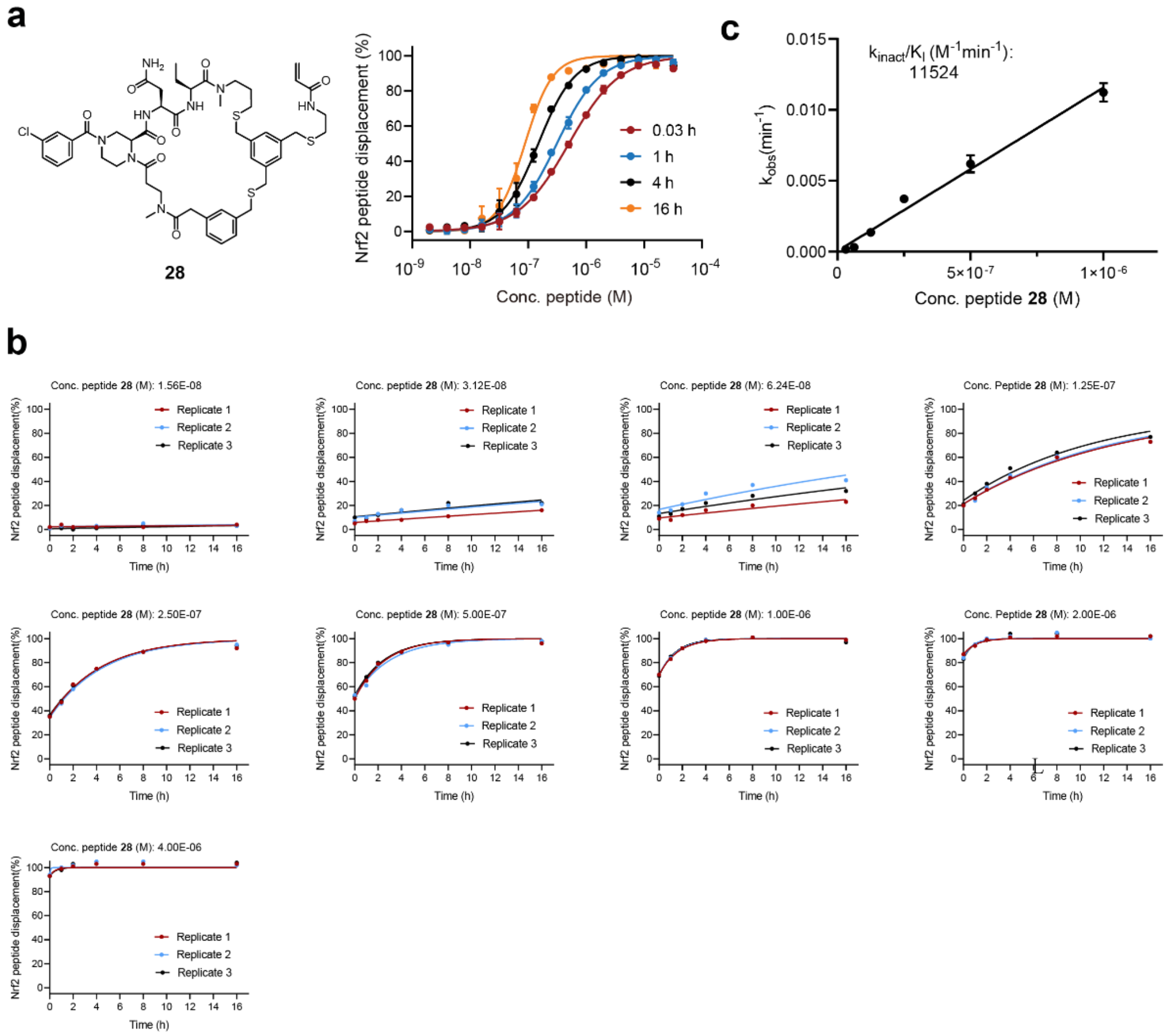

Supplementary Fig. 18. Macrocyclic covalent inhibitor 28. Building on the versatility of the dibromomethylbenzene linker L4 found in peptide **23**, which enables modular attachment of substituents via a third bromomethyl group at the 5-position, we synthesized peptide **28**, incorporating an acrylamide warhead for covalent targeting. This design yielded a k_{inact}/K_I value of 11,500 $M^{-1}min^{-1}$ which represented a 4-fold improvement over the linear covalent inhibitor **20-Cl**. **a,** Chemical structure of covalent inhibitor 26 bearing an acrylamide warhead. Displacement of Nrf2 peptide from Keap1 was measured by an FP assay at different incubation times. **b,** Determination of k_{obs} for covalent inhibitor peptide **28.** Keap1 was incubated with different concentrations of peptide **28** and the inhibition of the protein–protein interaction was measured in a fluorescence polarization competition assay. For each inhibitor concentration and time point, three measurements were performed (replicates performed in the same microwell plate). **c,** Measurement of second-order rate constant k_{inact}/K_I by determining the maximum rate of inactivation (k_{inact}) and the potency (K_I) of the first reversible binding event, based on the obtained K_{obs} values.

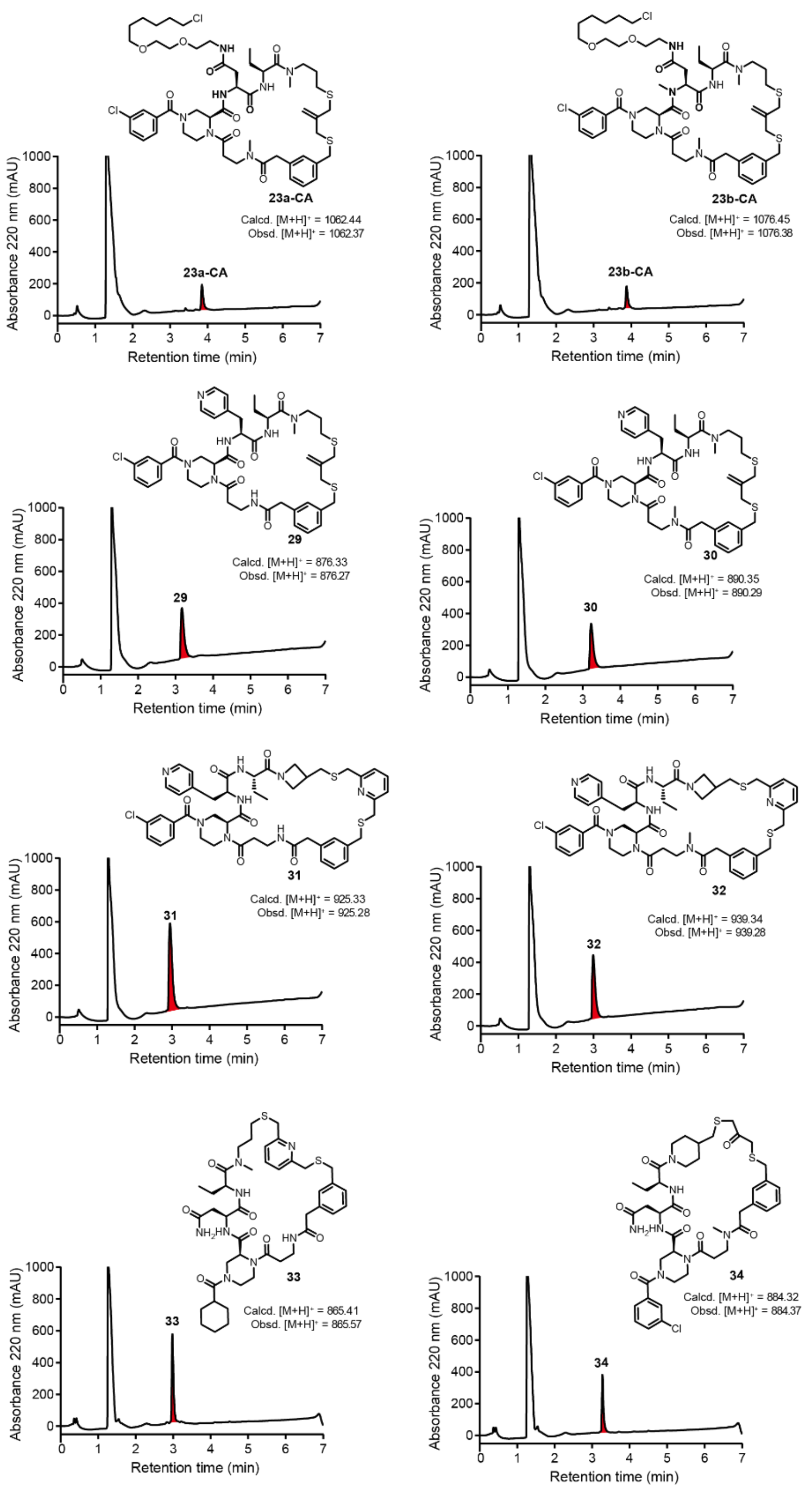

Supplementary Fig. 19. Purity and identity of cyclic peptides with chloroalkane tag (23a-CA, 23b-CA), reduced polar surface (29-32), and cyclic peptides for which the X-ray structure with Keap1 could be determined (33, 34). Chemical structures of the peptides are shown. The purity and identity of the peptides were assessed by LC-MS.

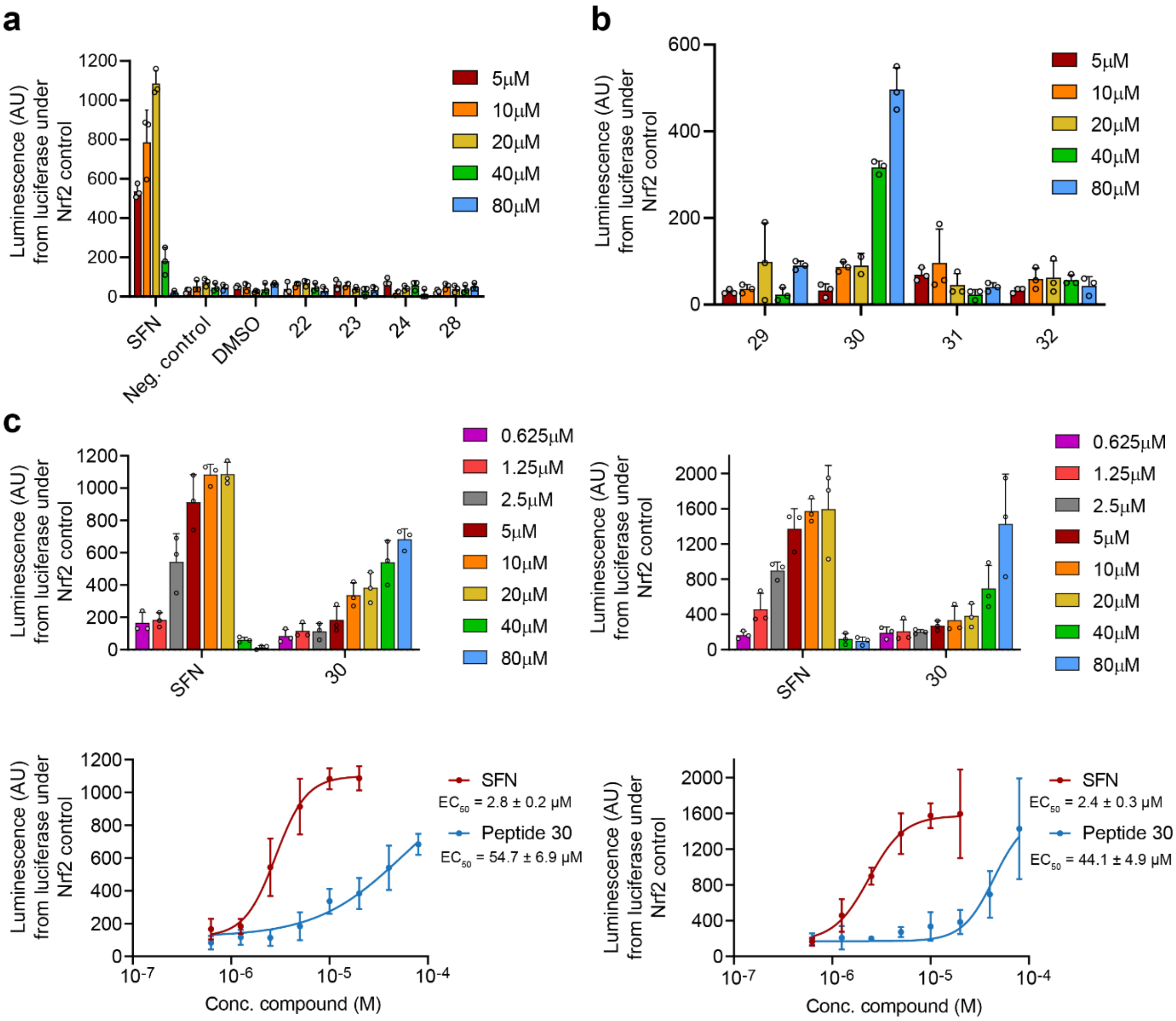

Supplementary Fig. 20. Repetition of cellular activity assays. a, Repetition of cellular activity of cyclic peptides **22**, **23**, **24**, and **28** in HEK293-ARE luciferase reporter cells. The non-specific sulforaphane (SFN) inhibitor was used as a positive control. Inactive peptides and DMSO served as negative controls. Mean values and SDs of three wells in the same plate are indicated (technical replicates). **b,** Repetition of the cellular activity of cyclic peptides **29-32** in HEK293-ARE luciferase reporter cells. Mean values and SDs of three wells in the same microwell plate are shown (technical replicates). **c,** Repetition of the cellular assay at more inhibitor concentrations and estimation of EC_{50}. Luciferase activity at maximal effect was assessed using the non-specific inhibitor SFN. Mean values and SDs of three wells in the same microwell plate are indicated (technical replicates).

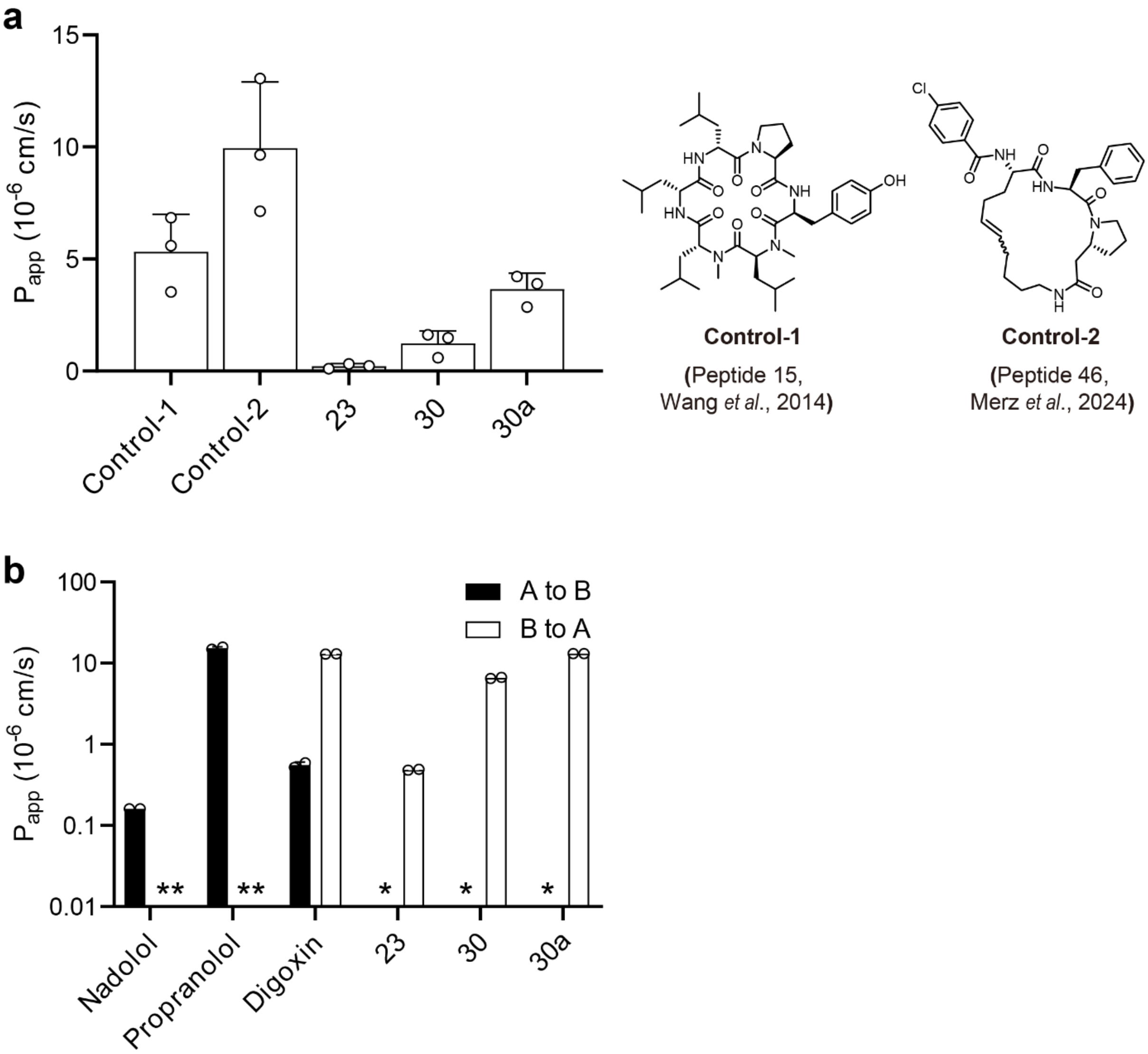

Supplementary Fig. 21. Membrane permeability measured by PAMPA (a) and Caco-2 (b). **a,** Passive membrane permeability of cyclic peptides **23**, **30** and **30a** (50 µM) was measured by the PAMPA assay. Mean and SD values from three technical replicates are shown. Control peptides were synthesized as previously described: control-1 (peptide 15) and control-2 (peptide 46). **b,** Membrane permeability measured by Caco-2 assay. Apparent permeability (P_{app}) of cyclic peptide **23**, **30** and **30a** was measured in the apical to the basal direction (black) and the basal to the apical direction (white). Nadolol and propranolol were used as negative and positive controls, respectively. Digoxin was used as a reference for transporter-mediated efflux. Mean values of two technical replicates are shown. *) No compound was detected. The detection limits were 2 nM for peptides **23** and **30**, and 4 nM for peptide **30a**. **) Permeability was measured only in the A to B direction.

a

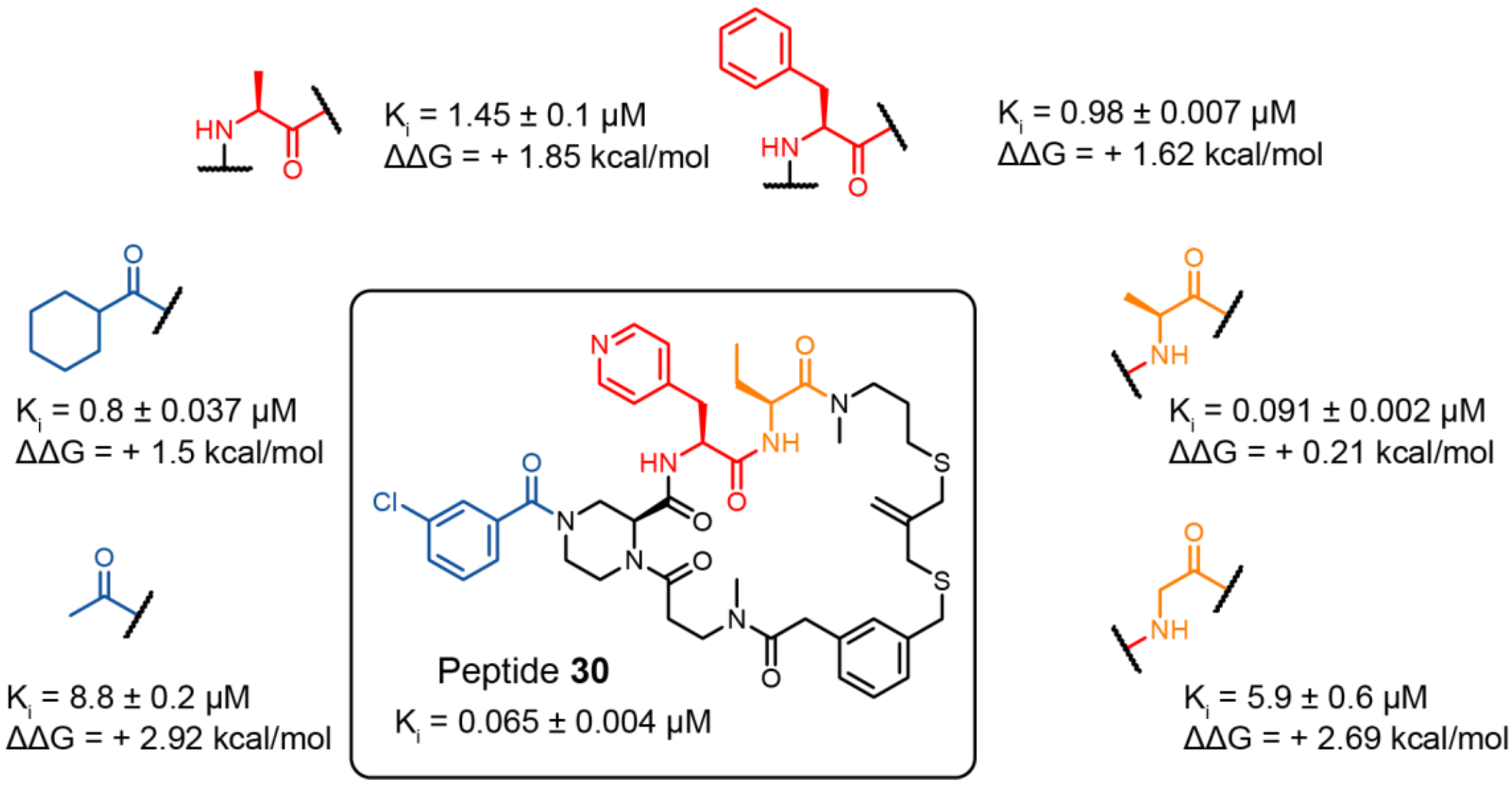

Supplementary Fig. 22. Structure-activity study of peptide 30. a, Amino acids were individually substituted to alanine or related structures. Mean values and SDs of three wells in the same microwell plate are indicated (technical replicates). **b,** Chemical structures of peptide **30** variants, HPLC chromatograms of purified peptides measured at an absorbance wavelength of 220 nm, and Nrf2 peptide displacement (%) measured using the fluorescence polarization competition assay.

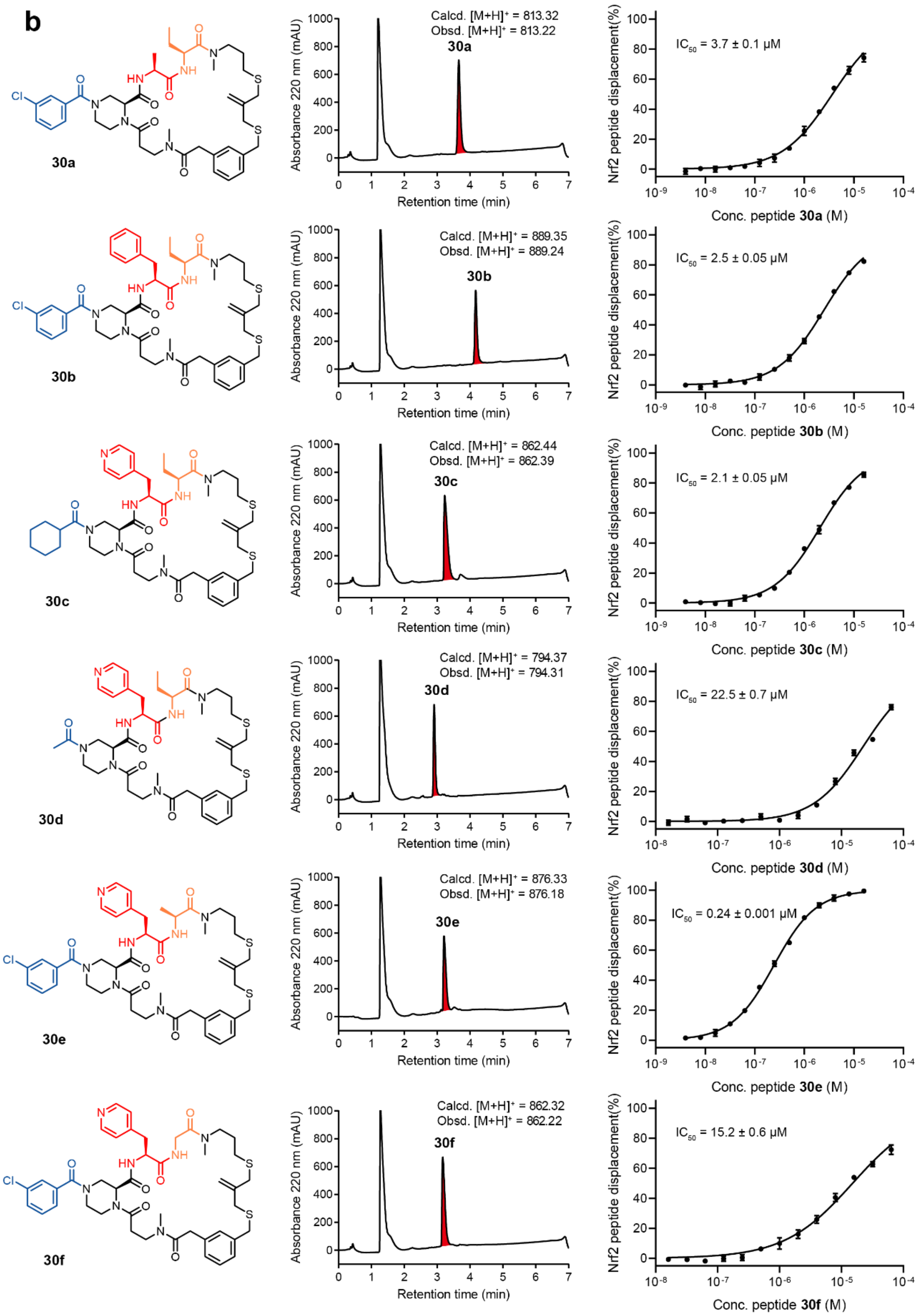

Supplementary Fig. 22. Continued

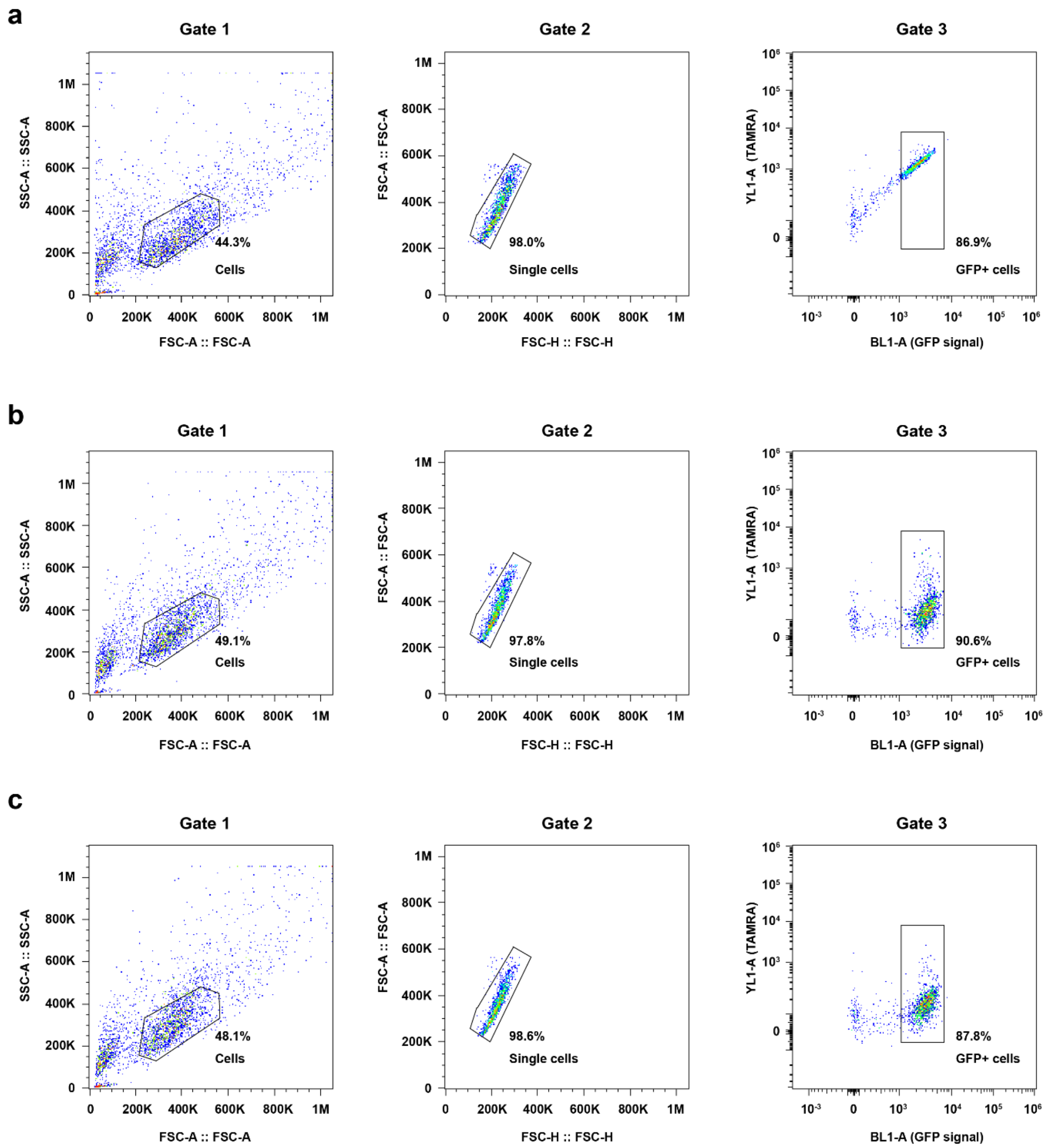

Supplementary Fig. 23. FACS gating strategies for CAPA assay. a, Exemplified gating strategy for maximal TAMRA fluorescence (DMSO, negative control). **b,** Exemplified gating strategy for minimal TAMRA fluorescence (10 mM CA-Trp-NH_2, positive control). **c,** Exemplified gating strategy for testing sample.

a

^{1}H NMR (400 MHz, DMSO-d_6) of M2

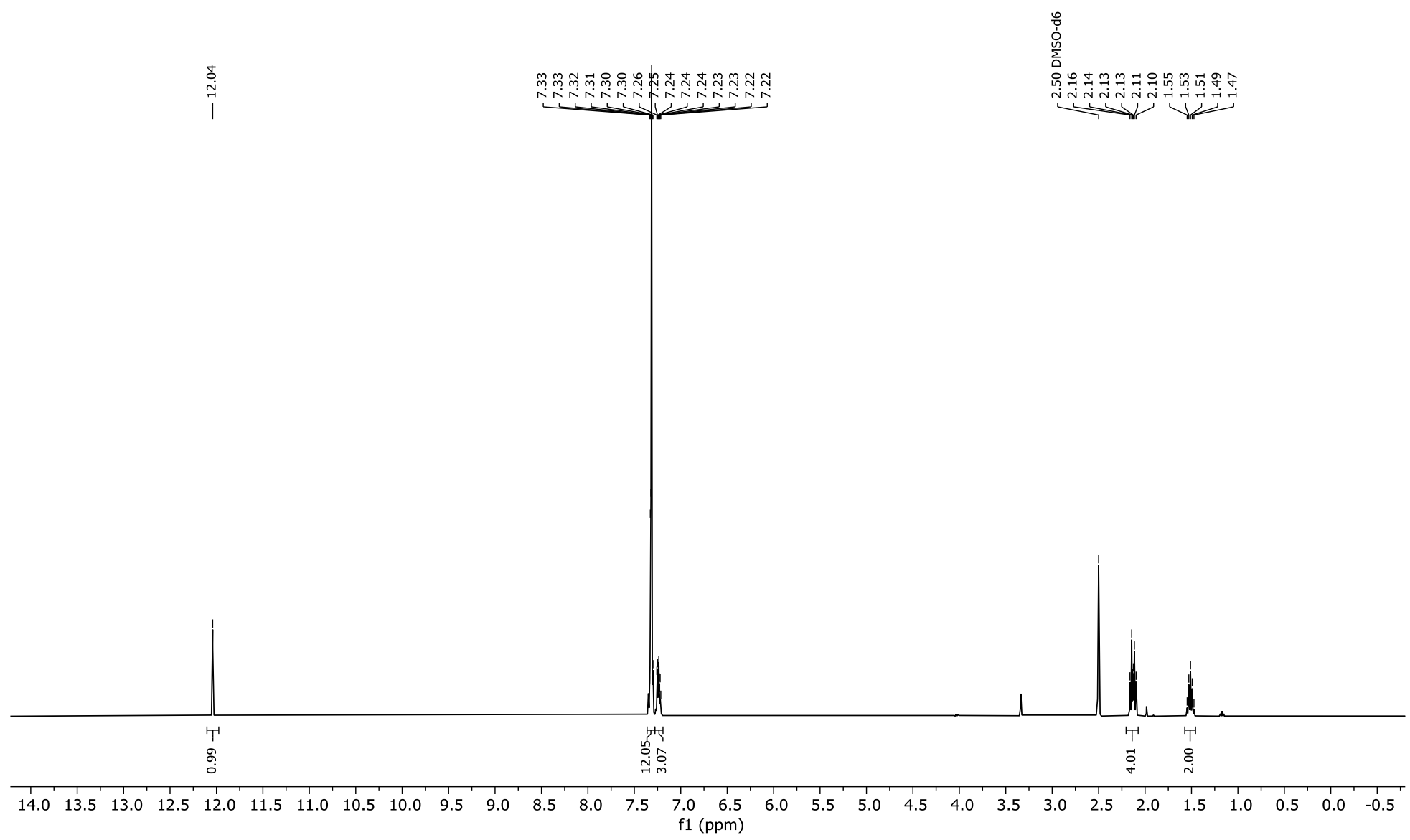

b

^{13}C NMR (101 MHz, DMSO) of M2

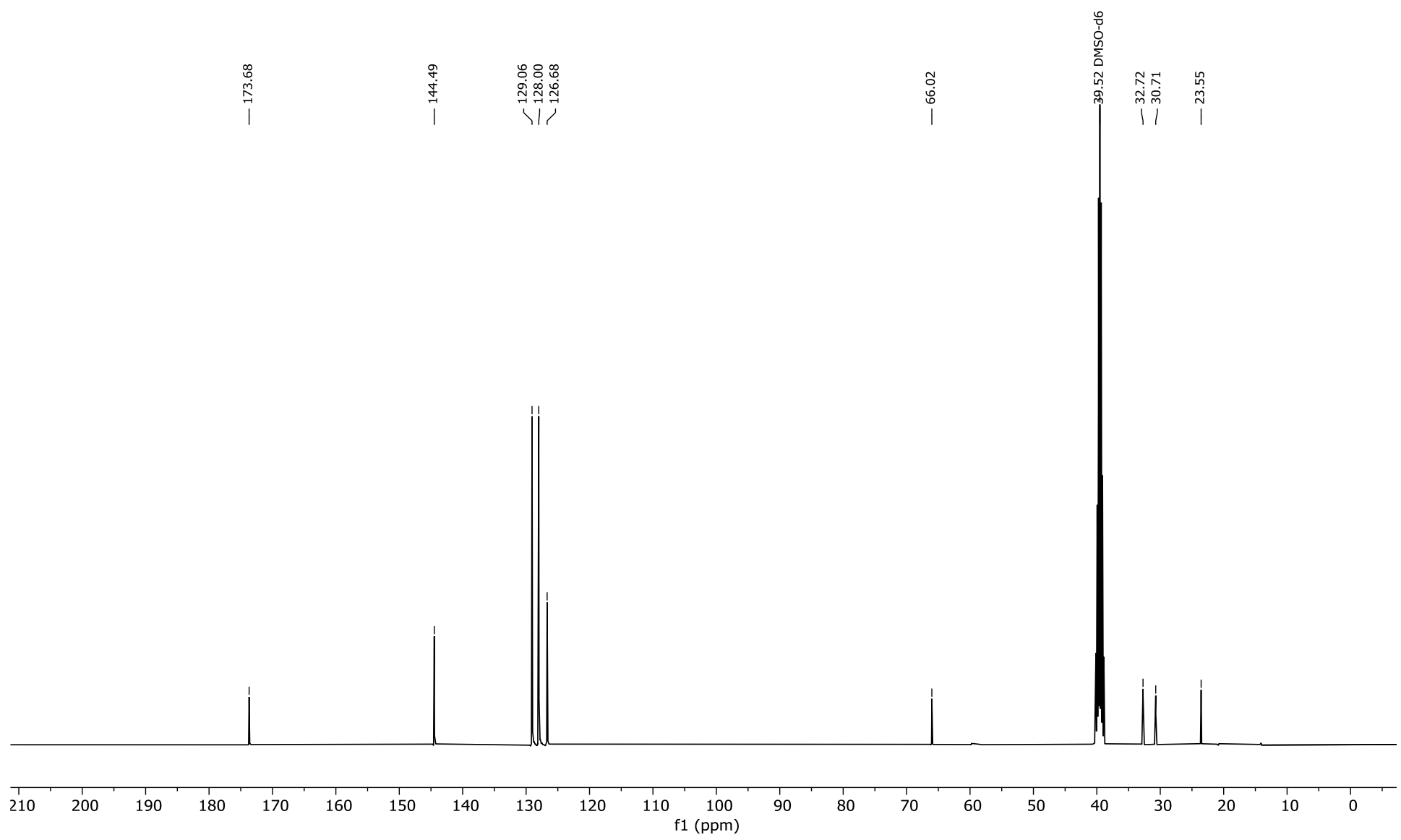

Supplementary Fig. 24. NMR spectra of 4-(tritylthio)-butanoic acid (M2). a, ^{1}H NMR. **b,** ^{13}C NMR.

a

^{1}H NMR (400 MHz, DMSO-d_6) of M3

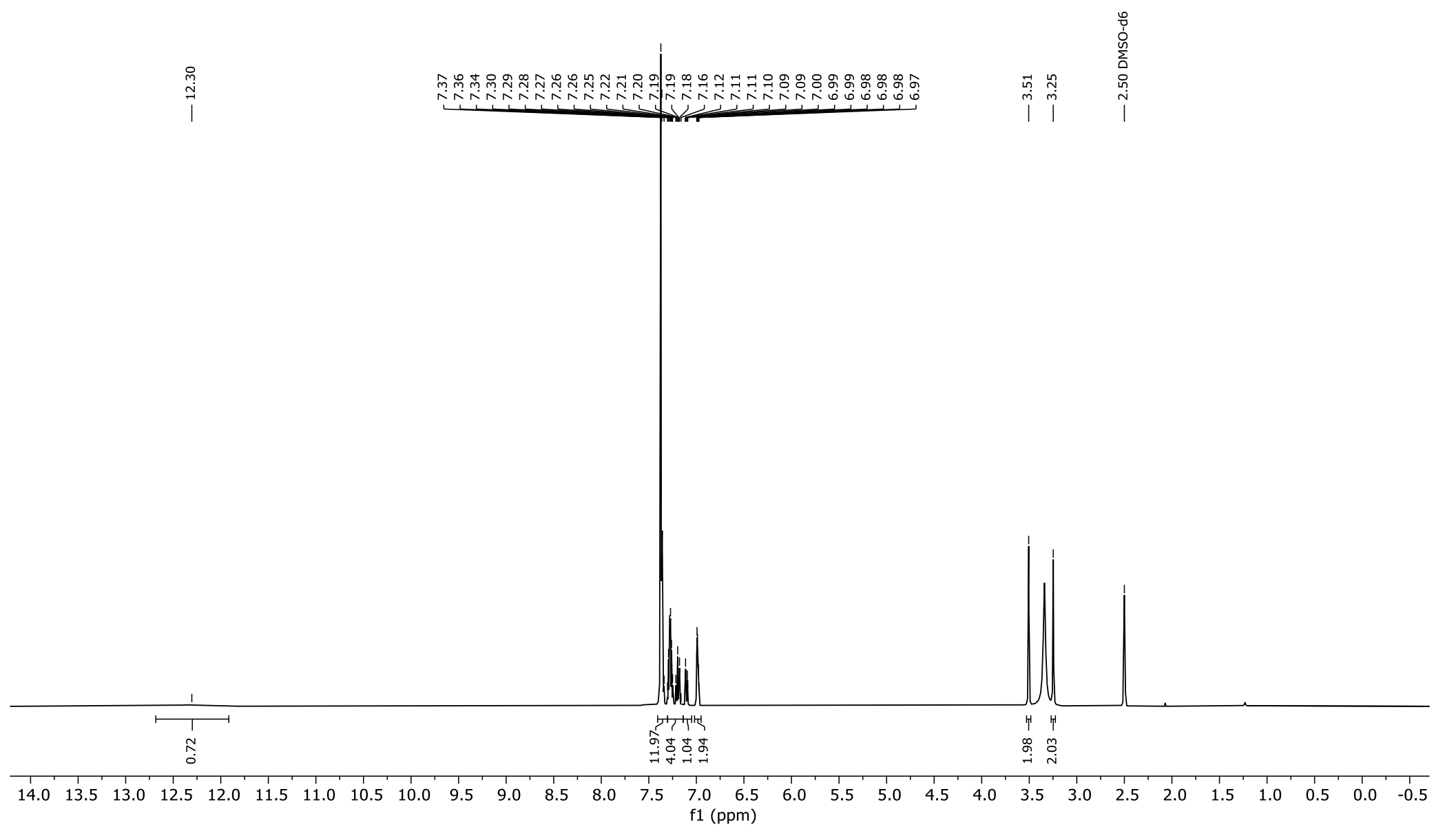

b

^{13}C NMR (101 MHz, DMSO) of M3

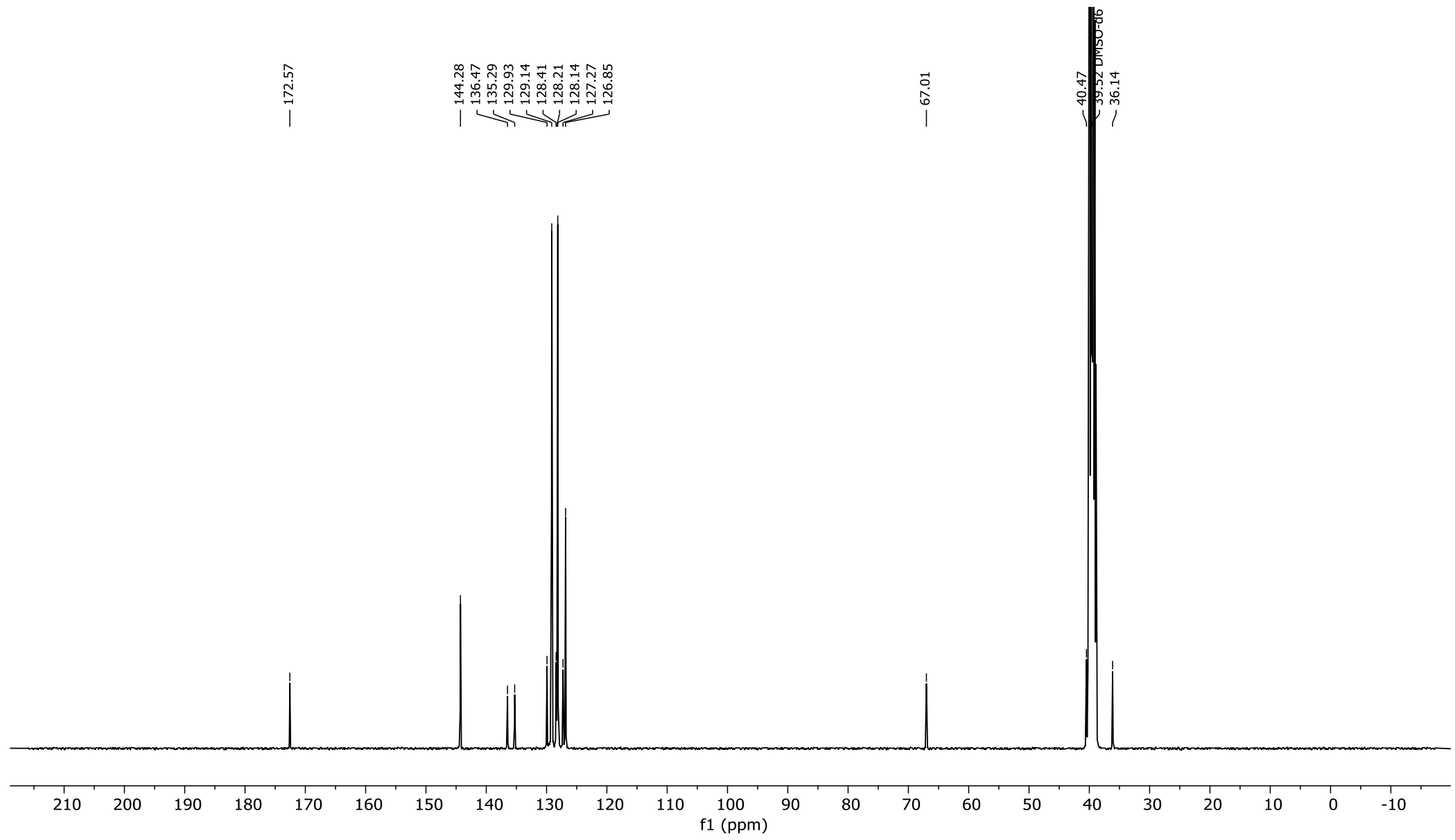

Supplementary Fig. 25. NMR spectra of 2-(3-((tritylthio)methyl)phenyl)-acetic acid (M3).
a, ^{1}H NMR. **b,** ^{13}C NMR.

Supplementary Tables

Supplementary Table 1. Amino acids with A code. The first ten amino acids of this group were chosen as aliphatic amino acids for the synthesis of Library 1. Further amino acids of this group were used for the SAR study where the aliphatic amino acid Abu was substituted.

Amino acid	Name	CAS number
A1	Fmoc-L-Abu-OH	135112-27-5
A2	Fmoc-L-Leu-OH	35661-60-0
A3	Fmoc-L-Gly(allyl)-OH	146549-21-5
A4	Fmoc-L-Chg-OH	161321-36-4
A5	Fmoc-L-HCha-OH	269078-73-1
A6	Fmoc-L-Val-OH	68858-20-8
A7	Fmoc-L-Ile-OH	71989-23-6
A8	Fmoc-D-Leu-OH	114360-54-2
A9	Fmoc-L-Cha-OH	135673-97-1
A10	Fmoc-L-Nle-OH	77284-32-3
A11	Fmoc-L-Ala-OH	35661-39-3
A12	Fmoc-Gly-OH	29022-11-5
A13	Fmoc-L-Lys(Boc)-OH	71989-26-9
A14	Fmoc-L-Arg(Pbf)-OH	154445-77-9
A15	Fmoc-L-Phe-OH	35661-40-6
A16	Fmoc-L-Ser(tBu)-OH	71989-33-8
A17	Fmoc-L-Thr(tBu)-OH	71989-35-0
A18	Fmoc-L-Glu(OtBu)-OH	71989-18-9

A19	Fmoc-L-Asp(OtBu)-OH	71989-14-5
A20	Fmoc-L-Trp(Boc)-OH	143824-78-6
A21	Fmoc-L-His(Trt)-OH	109425-51-6
A22	Fmoc-L-Gln(Trt)-OH	132327-80-1
A23	Fmoc-D-Ala-OH	79990-15-1
A24	Fmoc-D-Val-OH	84624-17-9
A25	Fmoc-D-Ile-OH	143688-83-9
A26	Fmoc-D-Phe-OH	86123-10-6
A27	Fmoc-D-Lys(Boc)-OH	92122-45-7
A28	Fmoc-D-Ser-OH	116861-26-8
A29	Fmoc-D-Thr(Trt)-OH	682800-84-6
A30	Fmoc-D-Glu(OtBu)-OH	104091-08-9
A31	Fmoc-D-Asp(O*t*-Bu)-OH	112883-39-3
A32	Fmoc-D-Trp(Boc)-OH	163619-04-3
A33	Fmoc-D-His(Trt)-OH	135610-90-1
A34	Fmoc-D-Gln-OH	112898-00-7
A35	Fmoc-D-Asn(Trt)-OH	180570-71-2
A36	Fmoc-NMe-L-Asn(Trt)-OH	941296-80-6
A37	Fmoc-NMe-L-Abu-OH	1310575-53-1
A38	Fmoc-L-Phe(4-Bz)-OH	117666-96-3
A39	Fmoc-L-Phe(3,4-difluoro)-OH	198560-43-9
A40	Fmoc-L-Tyr(3-chloro)-OH	478183-58-3
A41	Fmoc-L-HPhe-OH	132684-59-4
A42	Fmoc-L-1Nal-OH	96402-49-2

A43	Fmoc-L-2-Pal-OH	185379-40-2
A44	Fmoc-DL-Trp(5-hydroxy)-OH	351857-99-3
A45	Fmoc-L-Phe(4-carbamoyl)-OH	204716-17-6
A46	Fmoc-L-Phe(3-cyano)-OH	205526-36-9
A47	Fmoc-(3S)-bTyr-OH	501015-33-4
A48	Fmoc-L-3-Pal-OH	175453-07-3
A49	Fmoc-L-Ala(5-bromothiophen-2-yl)-OH	220497-50-7
A50	Fmoc-L-Tyr(3,5-diiodo)-OH	103213-31-6
A51	Fmoc-L-Cit-OH	133174-15-9
A52	Fmoc-L-Tle-OH	132684-60-7
A53	Fmoc-L-Ala(2-furyl)-OH	159611-02-6
A54	Fmoc-Aib-OH	94744-50-0
A55	Fmoc-L-Orn(Boc)-OH	109425-55-0
A56	Fmoc-L-Pro(4-(4R)STrt)-OH	281655-34-3
A57	Fmoc-ThpGly-OH	285996-72-7
A58	Fmoc-L-Thz-OH	133054-21-4
A59	Fmoc-DL-Gly(allyl)-OH	221884-63-5
A60	Fmoc-L-Bta-OH	177966-60-8
A61	Fmoc-L-Ala(cyano)-OH	127273-06-7
A62	1-Boc-4-(Fmoc-amino)piperidine-4-carboxylic acid	183673-66-7
A63	Fmoc-L-Dab(Boc)-OH	125238-99-5
A64	Fmoc-L-Abu(4-bromo)-OH	172169-88-9
A65	Fmoc-L-Phe(3-Boc-aminomethyl)-OH	266999-24-0
A66	Fmoc-L-Tpi-OH	204322-23-6

A67	Fmoc-DL-Idc-OH	198560-38-2
A68	Fmoc-L-4-Pal-OH	169555-95-7
A69	Fmoc-L-Allo-Thr(*t*-Bu)-OH	201481-37-0
A70	Fmoc-L-Oic-OH	130309-37-4
A71	Fmoc-D-Abu-OH	170642-27-0
A72	Fmoc-L-Tyr(-3-nitro)-OH	136590-09-5
A73	Fmoc-L-Ala(4-Thz)-OH	205528-32-1
A74	Fmoc-L-Ala(*t*-Bu)-OH	139551-74-9
A75	Fmoc-L-Cpg-OH	220497-61-0
A76	Fmoc-L-Trp(6-chloro)-OH	908847-42-7
A77	Fmoc-(3S)-bPhe(4-methoxy)-OH	501015-30-1
A78	Fmoc-L-Hse(methyl)-OH	173212-86-7
A79	Fmoc-L-Gly(pent-3-yl)-OH	1310680-47-7
A80	Fmoc-Deg-OH	218926-46-6
A81	Fmoc-L-Phe(4-iodo)-OH	82565-68-2
A82	Fmoc-L-Phe(4-nitro)-OH	95753-55-2
A83	Fmoc-L-Nva-OH	135112-28-6
A84	Fmoc-L-HLeu-OH	180414-94-2
A85	Fmoc-D-2-Pal-OH	185379-39-9
A86	Fmoc-L-Cpa-OH	214750-76-2
A87	Fmoc-D-Ala(4-Thz)-OH	205528-33-2
A88	Fmoc-L-Pra-OH	198561-07-8

Supplementary Table 2. Amino acids with D code. The first eight amino acids of this group were chosen as diamine amino acids for the synthesis of Library 1. Further amino acids of this group were used for the SAR study where the diamine amino acid was substituted.

Amino acid	Name	CAS number
D1	Fmoc-L-Dap(Boc)-OH	162558-25-0
D2	1-Boc-4-(Fmoc-amino)piperidine-4-carboxylic acid	183673-66-7
D3	1-Fmoc-4-Boc-piperazine-2-(2RS)acetic acid	183742-34-9
D4	1-Fmoc-4-Boc-piperazine-2-(2RS)carboxylic acid	183742-23-6
D5	Boc-L-Dap(Fmoc)-OH	122235-70-5
D6	1-Boc-4-Fmoc-piperazine-2-(2RS)carboxylic acid	218278-58-1
D7	Boc-L-Pro(4-(4S)Fmoc-amino)-OH	174148-03-9
D8	Boc-L-Dab(Fmoc)-OH	117106-21-5
D9	1-Fmoc-4-Boc-piperazine-2-(2R)carboxylic acid	1217723-28-8
D10	1-Fmoc-4-Boc-piperazine-2-(2S)carboxylic acid	244132-27-2
D11	Fmoc-Inp-OH	148928-15-8
D12	1-Fmoc-piperidine-3-(3S)carboxylic acid	193693-68-4
D13	Fmoc-bAla-OH	35737-10-1
D14	4-Fmoc-morpholine-2-(2RS)carboxylic acid	312965-04-1
D15	4-Fmoc-piperazine-1-carboxylic acid	180576-05-0
D16	Fmoc-2-Abz-OH	150256-42-1
D17	Fmoc-L-Tic-OH	136030-33-6
D18	Fmoc-4-Amb-OH	164470-64-8
D19	Fmoc-3-AM-PhAc-OH	631915-50-9
D20	Fmoc-(3S)-bHoasn(Trt)-OH	283160-20-3

D21	Fmoc-1,4-cis-ACHA-OH	1217675-84-7
D22	Fmoc-D-azetidine-2-carboxylic acid	374791-02-3
D23	Fmoc-(3S)-bHPro-OH	193693-60-6
D24	Fmoc-L-Tpi-OH	204322-23-6
D25	Fmoc-L-Idc-OH	198560-38-2
D26	1-Fmoc-piperidine-4-acetic acid	180181-05-9
D27	Fmoc-L-Oic-OH	130309-37-4
D28	Fmoc-L-NMe-Lys(Boc)-OH	197632-76-1
D29	Fmoc-L-Lys(Dde)-OH	150629-67-7
D30	Boc-L-Phe(4-Fmoc-aminomethyl)-OH	170157-61-6
D31	Fmoc-L-Pro(4-(4R)Boc-amino)-OH	273222-06-3
D32	1-Boc-3-(Fmoc-amino)piperidine-3-carboxylic acid	368866-21-1
D33	Boc-D-Dap(Fmoc)-OH	131570-56-4
D34	Fmoc-L-Dab(Boc)-OH	125238-99-5
D35	Fmoc-L-Pro(4-(4S)Boc-amino)-OH	221352-74-5

Supplementary Table 3. Amino acids with X code. The first 45 amino acids of this group were chosen as random structure amino acids for the synthesis of Library 1. Further amino acids of this group were used for the SAR study where the Asn was substituted.

Amino acid	Name	CAS number
X1	Fmoc-L-Pro-OH	71989-31-6
X2	Fmoc-D-azetidine-2-carboxylic acid	136552-06-2
X3	Fmoc-Inp-OH	148928-15-8
X4	Fmoc-L-Tic-OH	136030-33-6
X5	Fmoc-4-Amc-OH	188715-40-4
X6	Fmoc-AC5C-OH	117322-30-2
X7	1-Fmoc-piperidine-2-(2S)carboxylic acid	86069-86-5
X8	Fmoc-azetidine-3-carboxylic acid	193693-64-0
X9	-Fmoc-piperidine-3-(3S)carboxylic acid	193693-68-4
X10	Fmoc-3-Amb-OH	155369-11-2
X11	Fmoc-bAla-OH	35737-10-1
X12	Fmoc-L-Ser(*t*-Bu)-OH	71989-33-8
X13	Fmoc-L-Asn-OH	71989-16-7
X14	Fmoc-L-His(Trt)-OH	109425-51-6
X15	Fmoc-L-Ala(2-furyl)-OH	159611-02-6
X16	Fmoc-L-Met(O_2)-OH	163437-14-7
X17	Fmoc-D-Asn(Trt)-OH	180570-71-2
X18	Fmoc-Thr(*t*-Bu)-OH	71989-35-0
X19	Fmoc-L-Gln(Trt)-OH	132327-80-1
X20	Fmoc-L-Ala(4-Thz)-OH	205528-32-1

X21	Fmoc-L-Cit-OH	133174-15-9
X22	Fmoc-D-Ser(*t*-Bu)-OH	128107-47-1
X23	Fmoc-L-Phe-OH	35661-40-6
X24	Fmoc-L-Phg-OH	102410-65-1
X25	Fmoc-L-Phe(4-chloro)-OH	175453-08-4
X26	Fmoc-L-Ala(3-styryl)-OH	159610-82-9
X27	Fmoc-D-Trp(Boc)-OH	163619-04-3
X28	Fmoc-L-Phe(4-cyano)-OH	173963-93-4
X29	Fmoc-L-Trp(Boc)-OH	143824-78-6
X30	Fmoc-L-Tyr(*t*-Bu)-OH	71989-38-3
X31	Fmoc-L-Phe(2-trifluoromethyl)-OH	352523-16-1
X32	Fmoc-L-Tyr(Bzl)-OH	71989-40-7
X33	Fmoc-D-Tyr(3-chloro)-OH	478183-58-3
X34	Fmoc-L-Phe(3-fluoro)-OH	198560-68-8
X35	Fmoc-D-Abu-OH	170642-27-0
X36	Fmoc-D-Nva-OH	144701-24-6
X37	Fmoc-L-HLeu-OH	180414-94-2
X38	Fmoc-L-Ala-OH	35661-39-3
X39	Fmoc-D-Cha-OH	144701-25-7
X40	Fmoc-D-Met-OH	112883-40-6
X41	Fmoc-D-Val-OH	84624-17-9
X42	Fmoc-L-Nva-OH	135112-28-6
X43	Fmoc-D-Ala-OH	79990-15-1
X44	Fmoc-Aib-OH	94744-50-0

X45	Fmoc-L-Met-OH	71989-28-1
X46	Fmoc-Gly-OH	29022-11-5
X47	Fmoc-L-Val-OH	68858-20-8
X48	Fmoc-L-Ile-OH	71989-23-6
X49	Fmoc-L-Arg(Pbf)-OH	154445-77-9
X50	Fmoc-L-Lys(Boc)-OH	71989-26-9
X51	Fmoc-L-Glu(O*t*-Bu)-OH	71989-18-9
X52	Fmoc-Asp(O*t*-Bu)-OH	71989-14-5
X53	Fmoc-D-Leu-OH	114360-54-2
X54	Fmoc-D-Phe-OH	86123-10-6
X55	Fmoc-D-Ser-OH	116861-26-8
X56	Fmoc-D-Thr(Trt)-OH	682800-84-6
X57	Fmoc-D-Glu(O*t*-Bu)-OH	104091-08-9
X58	Fmoc-D-Asp(O*t*-Bu)-OH	112883-39-3
X59	Fmoc-D-His(Trt)-OH	135610-90-1
X60	Fmoc-D-Lys(Boc)-OH	92122-45-7
X61	Fmoc-D-Gln-OH	112898-00-7
X62	Fmoc-NMe-L-Asn(Trt)-OH	941296-80-6
X63	Fmoc-NMe-L-Abu-OH	1310575-53-1
X64	Fmoc-L-Phe(4-Bz)-OH	117666-96-3
X65	Fmoc-L-Phe(3,4-difluoro)-OH	198560-43-9
X66	Fmoc-L-1-Nal-OH	96402-49-2
X67	Fmoc-L-HPhe-OH	132684-59-4
X68	Fmoc-L-2-Pal-OH	185379-40-2

X69	Fmoc-DL-Trp(5-hydroxy)-OH	351857-99-3
X70	Fmoc-L-Phe(4-carbamoyl)-OH	204716-17-6
X71	Fmoc-L-Phe(3-cyano)-OH	205526-36-9
X72	Fmoc-(3S)-bTyr-OH	501015-33-4
X73	Fmoc-L-3-Pal-OH	175453-07-3
X74	Fmoc-L-Ala(5-bromothien-2-yl)-OH	220497-50-7
X75	Fmoc-L-Tyr(3,5-diiodo)-OH	103213-31-6
X76	Fmoc-L-Tie-OH	132684-60-7
X77	Fmoc-L-Orn(Boc)-OH	109425-55-0
X78	Fmoc-L-Cha-OH	135673-97-1
X79	Fmoc-L-Pro(4-(4R)STrt)-OH	281655-34-3
X80	Fmoc-ThpGly-OH	285996-72-7
X81	Fmoc-L-Thz-OH	133054-21-4
X82	Fmoc-DL-Gly(allyl)-OH	221884-63-5
X83	Fmoc-L-Bta-OH	177966-60-8
X84	1-Boc-4-(Fmoc-amino)piperidine-4-carboxylic acid	183673-66-7
X85	Fmoc-L-Dab(Boc)-OH	125238-99-5
X86	Fmoc-L-Nle-OH	77284-32-3
X87	Fmoc-L-Phe(3-Boc-aminomethyl)-OH	266999-24-0
X88	Fmoc-L-Tpi-OH	204322-23-6
X89	Fmoc-DL-Idc-OH	198560-38-2
X90	Fmoc-L-4-Pal-OH	169555-95-7
X91	Fmoc-L-allo-Thr(*t*-Bu)-OH	201481-37-0
X92	Fmoc-L-Oic-OH	130309-37-4

X93	Fmoc-L-Tyr(3-nitro)-OH	136590-09-5
X94	Fmoc-L-Ala(*t*-Bu)-OH	139551-74-9
X95	Fmoc-L-Cpg-OH	220497-61-0
X96	Fmoc-L-Trp(6-chloro)-OH	908847-42-7
X97	Fmoc-(3S)-bPhe(4-fluoro)-OH	479064-89-6
X98	Fmoc-(3S)-bPhe(4-methoxy)-OH	501015-30-1
X99	Fmoc-L-Hse(methyl)-OH	173212-86-7
X100	Fmoc-L-Phe(3,4-dichloro)-OH	177966-59-5
X101	Fmoc-L-Gly(pent-3-yl)-OH	1310680-47-7
X102	Fmoc-Deg-OH	218926-46-6
X103	Fmoc-L-Phe(4-iodo)-OH	82565-68-2
X104	Fmoc-Phe(4-nitro)-OH	95753-55-2
X105	Fmoc-D-2-Pal-OH	185379-39-9
X106	Fmoc-L-Cpa-OH	214750-76-2
X107	Fmoc-D-Ala(4-Thz)-OH	205528-33-2
X108	Fmoc-L-Tyr(3,5-dibromo)-OH	201484-26-6
X109	Fmoc-L-Pra-OH	198561-07-8
X110	Fmoc-Peg2-OH	872679-70-4
X111	Fmoc-NMe-bAla-OH	172965-84-3
X112	Fmoc-Peg1-OH	260367-12-2
X113	Fmoc-(3S)-bHPro-OH	193693-60-6

Supplementary Table 4. Carboxylic acids with C code. The first ten carboxylic acids of this group were chosen as random structure carboxylic acids for the synthesis of Library 1. Further carboxylic acids of this group were used for the SAR study.

Carboxylic acid	Name	CAS number
C1	1-(4-Fluorobenzenesulphonyl)pyrrolidine-2-carboxylic acid	251096-96-5
C2	1-(Methylsulfonyl)-4-piperidinecarboxylic acid	280772-00-1
C3	Tetrahydro-2H-thiopyran-4-carboxylic acid 1,1-dioxide	64096-87-3
C4	2-(3-Chlorophenoxy)propionic acid	101-10-0
C5	4-[(Methylsulphonyl)amino]benzoic acid	7151-76-0
C6	4-Fluoro-3-(trifluoromethyl)benzoic acid	67515-55-3
C7	3,5-Dimethoxybenzoic acid	1132-21-4
C8	3,4-Methylenedioxyphenylacetic acid	2861-28-1
C9	Cyclohexane carboxylic acid	98-89-5
C10	4-Acetamidobenzoic acid	556-08-1
C11	Acetic acid	64-19-7
C12	3-Chlorobenzoic acid	535-80-8
C13	4-(Hydroxymethyl)phenoxyacetic acid	68858-21-9
C14	4-Fluoro-2-hydroxybenzoic acid	345-29-9
C15	1H-Tetrazole-5-acetic acid	21743-75-9
C16	2-Furoic acid	88-14-2
C17	3,5-Difluorobenzoic acid	455-40-3
C18	5-Hydroxypyrazine-2-carboxylic acid	34604-60-9
C19	4-Chlorobenzoic acid	74-11-3
C20	5-Chlorothiophene-2-carboxylic acid	24065-33-6

C21	4-Methyl-1,2,3-thiadiazole-5-carboxylic acid	18212-21-0
C22	1-Methyl-1H-pyrazole-5-carboxylic acid	16034-46-1
C23	3-Hydroxybenzoic acid	99-06-9
C24	4-Imidazolecarboxylic acid	1072-84-0
C25	Benzoic acid	65-85-0
C26	5-Hydroxyindole-2-carboxylic acid	21598-06-01
C27	5-Norbornene-2-carboxylic acid	120-74-1
C28	Nicotinic acid	59-67-6
C29	5-Bromo-2-thiophenecarboxylic acid	7311-63-9
C30	5-Chlorofuran-2-carboxylic acid	618-30-4
C31	3,4-Dimethoxyphenylacetic acid	93-40-3
C32	2,2-Difluoro-1,3-benzodioxole-4-carboxylic acid	126120-85-2
C33	5-Benzimidazolecarboxylic acid	15788-16-6
C34	1H-Indazole-5-carboxylic acid	61700-61-6
C35	Phenoxyacetic acid	122-59-8
C36	5-Chloroindole-2-carboxylic acid	10517-21-2
C37	5-Chloro-1H-indole-3-carboxylic acid	10406-05-0
C38	3-(4-Chlorophenyl)propionic acid	2019-34-3
C39	Tetrahydropyran-4-carboxylic acid	1255400
C40	4-Chlorophenylacetic acid	1878-66-6
C41	4-Hydroxybenzoic acid	99-96-7
C42	4-(Pentafluorothio)benzoic acid	832-32-6
C43	5-Oxazolecarboxylic acid	118994-90-4
C44	5-Methylisoxazole-3-carboxylic acid	3405-77-4

C45	Pyrazole-3-carboxylic acid	1621-91-6
C46	2-Methoxypyridine-5-carboxylic acid	66572-55-2
C47	4-Pyrimidinecarboxylic acid	31462-59-6
C48	1-Phenyl-1H-pyrazole-4-carboxylic acid	1134-50-5
C49	(2-Methoxyphenoxy)acetic acid	1878-85-9
C50	4-(4-Fluorophenoxy)benzoic acid	129623-61-6
C51	1-Methyl-5-oxopyrrolidine-3-carboxylic acid	42346-68-9
C52	2-Morpholinoacetic acid hydrochloride	89531-58-8
C53	3-Methyloxetane-3-carboxylic acid	28562-68-7
C54	4-Methoxybenzoic acid	100-09-04
C55	(-)-Shikimic acid	138-59-0
C56	2,5-Dimethylbenzoic acid	610-72-0
C57	Pyridazine-4-carboxylic acid	50681-25-9
C58	Indan-2-carboxylic acid	25177-85-9
C59	4,7-Dimethylpyrazolo[5,1-c][1,2,4]triazine-3-carboxylic acid	175137-58-3
C60	5-(2-Furyl)-1H-pyrazole-3-carboxylic acid	116153-81-2
C61	N-Acetylglycine	543-24-8
C62	1-Pyrimidin-2-yl-piperidine-4-carboxylic acid	303144-44-7
C63	Trans-cinnamic acid	140-10-3
C64	Monomethyl succinate	3878-55-5
C65	1-(Carbamoylmethyl)cyclohexaneacetic Acid	99189-60-3
C66	1-Phenylcyclohexanecarboxylic acid	1135-67-7
C67	3-Chlorophenylacetic acid	1878-65-5
C68	1-(2-Methoxyethyl)-6-oxopiperidine-3-carboxylic acid	915920-08-0

C69	7-Azaindole-3-carboxylic acid	156270-06-3
C70	5-Methyl-4-oxo-3,4-dihydro-thieno[2,3-d]pyrimidine-6-carboxylic acid	101667-97-4
C71	6-Chloro-imidazo[1,2-a]pyridine-2-carboxylic acid	182181-19-7
C72	1-Methylpiperidine-4-carboxylic acid	68947-43-3
C73	4-Acetamidobenzoic acid	556-08-01
C74	3-(Phenylsulfonyl)propanoic acid	10154-71-9
C75	(2-Methyl-3,5-dioxo-4-azatricyclo[5.2.1.0 2,6]dec-8-en-4-yl)acetic acid	1005134-08-6
C76	3-Phenoxybenzoic acid	3739-38-6
C77	trans-3-Chloroacrylic acid	2345-61-1
C78	trans-4-chlorocinnamic acid	1615-02-07
C79	trans-4-Benzyloxycinnamic acid	6272-45-3
C80	trans-2-Phenylcyclopropane-1-carboxylic acid	939-90-2
C81	3-(2-Naphthyl)acrylic acid	51557-26-7
C82	2-Naphthoic acid	93-09-4
C83	4-Methoxycinnamic acid	830-09-01
C84	3,4-Methylenedioxycinnamic acid	2373-80-0
C85	3-Methoxycinnamic acid	6099-04-03
C86	Quinoxaline-2-carboxylic acid	879-65-2
C87	2-Methyl-3-phenylacrylic acid	1199-77-5
C88	3-Chlorocinnamic acid	1866-38-2
C89	4-Propoxycinnamic acid	69033-81-4
C90	5-Bromovaleric acid	2067-33-6
C91	3-Trifluoromethylcinnamic acid	779-89-5
C92	4-Fluorocinnamic acid	459-32-5

C93	4-Bromobenzoic acid	586-76-5
C94	3,4-Dihydroxybenzoic acid	99-50-3
C95	1H-Indazole-6-carboxylic acid	704-91-6
C96	4-Bromobutyric acid	2623-87-2
C97	3,4-Dichlorobenzoic acid	51-44-5
C98	7-Azaindole-5-carboxylic acid	754214-42-1
C99	2H-Chromene-3-carboxylic acid	22649-28-1
C100	Isoquinoline-6-carboxylic acid	106778-43-2
C101	3,4-Dichlorocinnamic acid	1202-39-7
C102	4-Trifluoromethoxycinnamic acid	783-13-1
C103	4-Ethoxycinnamic acid	2373-79-7
C104	3-Formylbenzoic acid	619-21-6
C105	2-[(Methylsulphonyl)amino]benzoic acid	162787-61-3
C106	5-Chloronicotinic acid	22620-27-5
C107	5-Chloropyridine-2-carboxylic acid	86873-60-1
C108	5-Chloro-2-methoxynicotinic acid	54916-65-3
C109	4-Chloro-2-fluorobenzoic acid	446-30-0
C110	4-Chloro-2-methoxybenzoic acid	57479-70-6
C111	E-3-(2,2-Difluoro-1,3-benzodioxol-5-yl)acrylic acid	387350-55-2
C112	6-Bromohexanoic acid	4224-70-8
C113	1-Methylindazole-3-carboxylic acid	50890-83-0
C114	1H-Pyrrole-2-carboxylic acid	634-97-9
C115	Indole-2-carboxylic acid	1477-50-5
C116	Cis-4-Hydroxycyclohexanecarboxylic acid	3685-22-1

C117	3-Morpholinobenzoic acid	215309-00-5
C118	1-Methyl-1H-Pyrazole-3-carboxylic acid	25016-20-0
C119	2-Morpholinobenzoic acid	42106-48-9
C120	4-(Morpholinomethyl)benzoic acid	62642-62-0
C121	2-Trifluoromethyl-6-pyridinecarboxylic acid	131747-42-7
C122	4-Imidazoleacetic acid	3251-69-2
C123	1-(Trifluoromethyl)cyclopropane-1-carboxylic acid	277756-46-4
C124	1-Methyl-3-(trifluoromethyl)-1H-pyrazole-4-carboxylic acid	113100-53-1
C125	3,3-Difluorocyclobutanecarboxylic acid	107496-54-8
C126	Benzothiazole-6-carboxylic acid	3622-35-3
C127	Indole-7-carboxylic acid	1670-83-3
C128	4-Morpholinobenzoic acid	7470-38-4
C129	Indole-4-carboxylic acid	2124-55-2
C130	(R)-(-)-Mandelic acid	611-71-2
C131	2-Hydroxy-6-methylnicotinic acid	38116-61-9
C132	5-Methyl-2-(2H-1,2,3-Triazol-2-Yl)benzoic acid	956317-36-5
C133	4-(4-Methylpiperazin-1-Yl)benzoic acid	354813-15-3
C134	2-(1,2-Benzisoxazol-3-yl)acetic acid	4865-84-3
C135	5-Hydroxymethyl-2-furancarboxylic acid	6338-41-6
C136	4-Methyl-2-propyl-1H-benzo[D]Imidazole-6-carboxylic acid	152628-03-0
C137	2,6-Dichlorobenzoic acid	50-30-6
C138	Anthracene-9-carboxylic acid	723-62-6
C139	4-Fluorbenzoic acid	456-22-4
C140	Xanthene-9-carboxylic acid	82-07-5

C141	2,4-Difluorobenzoic acid	1583-58-0
C142	Biphenyl-3-carboxylic acid	716-76-7
C143	5-Fluoroindole-2-Carboxylic Acid	399-76-8
C144	5-Phenylisoxazole-3-carboxylic acid	14441-90-8
C145	4-(1H-Pyrazol-1-yl)benzoic acid	16209-00-0

Supplementary Table 5. Quality analysis of cyclic peptide cores produced by SPPS for the synthesis of Library 1. Random wells were chosen from each one of the four 384-well plates and the products analyzed by LC-MS (see Supplementary figures). Products and side products were identified based on the mass and the percentage quantified based on the UV spectra (area of the peaks).

	Cyclic peptide core	Desired product (%)	Dimer (%)	tBu modified peptide (%)	Unidentified peptide (%)
**Plate 1**	4	52.4	41.4	6.2	0.0
	69	63.6	17.1	17.8	1.5
	130	85.0	5.2	8.8	1.0
	144	89.3	4.9	5.9	0.0
	161	72.0	22.7	0.0	5.4
	192	80.8	6.1	13.1	0.0
	205	85.7	3.6	7.8	2.9
	218	86.3	1.5	12.1	0.0
	223	90.4	0.0	5.5	4.1
	240	85.5	6.5	4.7	3.2
	279	90.4	5.8	1.6	2.2
	378	86.5	8.7	2.1	2.7
**Plate 2**	388	61.5	33.3	5.2	0.0
	399	64.0	7.9	3.6	24.4
	419	78.1	3.9	12.7	5.3
	460	87.2	4.7	8.1	0.0
	530	93.6	0.0	6.4	0.0
	566	88.0	7.5	3.3	1.1
	603	82.8	9.0	5.2	3.0
	632	89.6	2.7	6.8	0.9
	655	66.8	12.2	17.3	3.7
	680	92.3	0.0	7.7	0.0
	718	81.6	12.7	4.0	1.7
	754	89.3	7.0	0.0	3.7
**Plate 3**	769	58.2	41.8	0.0	0.0
	820	85.6	0.0	14.4	0.7
	888	97.5	2.5	0.0	0.0
	905	81.3	7.8	1.9	9.8
	923	81.5	1.2	17.3	0.0
	935	89.7	0.0	10.3	0.0
	961	80.9	13.0	6.0	0.0
	1005	89.1	6.2	0.0	4.7
	1020	79.0	16.5	0.0	4.5
	1040	89.2	9.6	1.2	0.0
	1041	96.4	0.0	0.0	3.6
	1084	98.1	0.9	1.0	0.0
**Plate 4**	1159	87.4	2.2	10.4	0.0
	1186	95.8	0.0	0.0	4.2

	1226	81.2	0.0	16.1	2.7
	1234	92.5	2.1	3.4	1.9
	1282	90.9	0.7	5.3	3.1
	1312	72.8	23.3	1.2	2.7
	1358	91.7	0.0	1.7	6.6
	1374	85.3	0.4	14.2	0.0
	1400	94.7	0.0	0.0	5.3
	1404	91.4	0.0	8.6	0.0
	1457	89.4	5.3	1.7	3.6
	1533	88.2	6.2	1.2	4.4
**Average**		84	7.6	5.9	2.6

Supplementary Table 6. Overview of peptide library high-throughput screens.

Category	Parameter	Description
Library 1		
Assay	Type of assay	In vitro
	Target	Human Kelch domain of Keap1 (321–609)
	Primary measurement	TR-FRET signal between FRET donor and acceptor complex
	Key reagents	LANCE-Eu-W1024 Anti 6xHis (PerkinElmer) and Sulfo-Cy5-labeled Nrf2-peptide
	Assay protocol	Described in *Methods section High-throughput screening for Keap1-Nrf2 inhibition by TR-FRET*
	Additional comments	-
Library	Library size	16896 (Library 1)
	Library composition	Cyclic peptide
	Source	Combinatorial synthesis. Described in Methods section *Random cyclic peptide library synthesis*
	Additional comments	-
Screen	Format	384 well microtiter plates (NUNC™, 264705)
	Concentration(s) tested	25 µM
	Plate controls	8 wells were used for positive controls (omission of Keap1) and 8 wells were used for negative controls (DMSO)
	Reagent/ compound dispensing system	Echo 650 (Beckman Coulter), CERTUS FLEX (Gyger)
	Detection instrument and software	PHERAstar FSX, BMG, SX software (version 5.70, Edition 8)
	Assay validation/ QC	Z’ score: 0.96
	Correction factors	-
	Normalization	The percentage of the FRET signal between the positive and negative controls.
	Additional comments	-
Post-HTS analysis	Hit criteria	Higher than 40% inhibition as compared to DMSO
	Hit rate	0.3%
	Additional assay(s)	Resynthesis and retesting of 2 structurally similar hits in the original assay
	Confirmation of hit purity and structure	Compounds were resynthesized and verified analytically
	Additional comments	-
Library 2		
Assay	Type of assay	In vitro
	Target	Human Kelch domain of Keap1 (321–609)
	Primary measurement	TR-FRET signal between FRET donor and acceptor complex
	Key reagents	LANCE-Eu-W1024 Anti 6xHis (PerkinElmer) and Sulfo-Cy5-labeled Nrf2-peptide
	Assay protocol	Described in Methods section *High-throughput screening for Keap1-Nrf2 inhibition by TR-FRET*
	Additional comments	-

Library	Library size	497 (Library 2)
	Library composition	Linear peptide
	Source	Solid phase synthesis. Described in Methods section High-throughput synthesis of linear peptides for SAR analysis
	Additional comments	-
Screen	Format	384 well microtiter plates (NUNC™, 264705)
	Concentration(s) tested	10 µM
	Plate controls	4 wells were used for positive controls (KI696; MedChemExpress, HY-101140) and 5 wells were used for negative controls (DMSO)
	Reagent/ compound dispensing system	Echo 650 (Beckman Coulter), CERTUS FLEX (Gyger)
	Detection instrument and software	PHERAstar FSX, BMG, SX software (version 5.70, Edition 8)
	Assay validation/ QC	Z' score: 0.96
	Correction factors	-
	Normalization	The percentage of the FRET signal between the positive and negative controls.
	Additional comments	-
Post-HTS analysis	Hit criteria	Higher than 60% inhibition as compared to DMSO
	Hit rate	10.7%
	Additional assay(s)	Resynthesis and retesting of 11 hits in the original assay
	Confirmation of hit purity and structure	Compounds were resynthesized and verified analytically
	Additional comments	-
Library 3-5		
Assay	Type of assay	In vitro
	Target	Human Kelch domain of Keap1 (321–609)
	Primary measurement	Fluorescence polarization values measured by plate reader (excitation: 485 nm, emission: 535 nm)
	Key reagents	Fluorescein labeled Nrf2-peptide
	Assay protocol	Described in Methods section *Keap1-Nrf2 fluorescence polarization competition binding assay*
	Additional comments	-
Library	Library size	768 (Library 3-5)
	Library composition	Cyclic peptide
	Source	Combinatorial synthesis. Described in Methods section *High-throughput synthesis of linker-cyclized peptide library*
	Additional comments	-
Screen	Format	384 well microtiter plates (NUNC™, 264705)
	Concentration(s) tested	1 µM
	Plate controls	4 wells were used for positive controls (KI696; MedChemExpress, HY-101140) and 5 wells were used for negative controls (DMSO)
	Reagent/ compound dispensing system	Echo 650 (Beckman Coulter), CERTUS FLEX (Gyger)
	Detection instrument and software	PHERAstar FSX, BMG, SX software (version 5.70, Edition 8)

	Assay validation/ QC	Z' score: 0.95
	Correction factors	-
	Normalization	The percentage of the Fluorescence polarization signal between the positive and negative controls.
	Additional comments	-
Post-HTS analysis	Hit criteria	Higher than 40% inhibition as compared to DMSO
	Hit rate	7.6%
	Additional assay(s)	Resynthesis and retesting of 8 structurally similar hits in the original assay
	Confirmation of hit purity and structure	Compounds were resynthesized and verified analytically
	Additional comments	-

Supplementary Table 7. Data collection and refinement statistics for X-ray co-crystal structure of peptide 6 bound to Kelch domain of Keap1. Statistics on X-ray diffraction data collection and refinement of Keap1 Kelch domain in complex with peptide **6** (PDB 9IH9). A single crystal was used to collect all diffraction data. Highest-resolution shell statistics are shown within brackets.

	Keap1 Kelch domain in complex with peptide 6
**Data collection**	
Space group	C 1 2 1
Cell dimensions	
a, b, c (Å)	78.76, 133.93, 82.32
α, β, γ (°)	90.00, 101.36, 90.00
Resolution (Å)*	19.74 – 1.70 (1.73 - 1.70)
R_{sym} or R_{merge}*	0.111 (1.079)
I / σI*	7.5 (1.9)
Completeness (%)*	95.3 (95.8)
Redundancy*	2.4 (2.4)
**Refinement**	
Resolution (Å)	19.74 – 1.70
No. reflections	207109 (10504)
R_{work} / R_{free}	0.1605 (0.2794) / 0.2029 (0.2949)
No. atoms	7446
Protein	6633
Ligand/ion	141
Water	672
B-factors	18.36
Protein	17.57
Ligand/ion	23.28
Water	25.1
R.m.s. deviations	
Bond lengths (Å)	0.007
Bond angles (°)	0.9

*Values in parentheses are for highest-resolution shell.

Supplementary Table 8. Crystallization attempts and results. Commercially available kits for crystallization screening were used. Fotos of crystals were taken by the authors X.J and K.L.

Cmpd	Chemical structure	Crystal photo	Crystallization screening condition	Comments
**4**	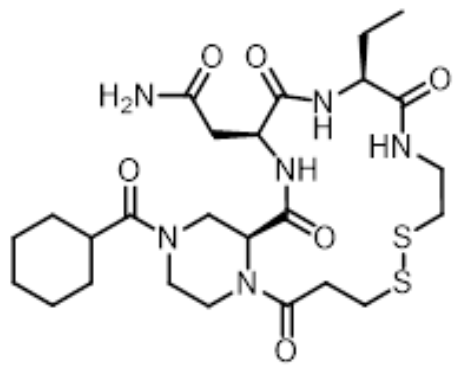	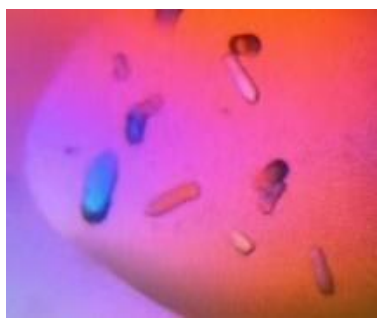	1. BCS (Basic Chemical Space) Screen (96 conditions<br>2. LMB Crystallisation Screen (96 conditions)<br>3. SG1™ Screen (96 conditions)<br>4. Clear Strategy™ Screen I HT (96 conditions)<br>5. Clear Strategy™ Screen II HT (96 conditions)<br>6. NeXtal AmSO4 Suite (96 conditions)	The diffraction data are high-resolution, but the pattern is poor, likely due to incomplete peptide ligand occupancy causing local disorder in the crystal.
**5**	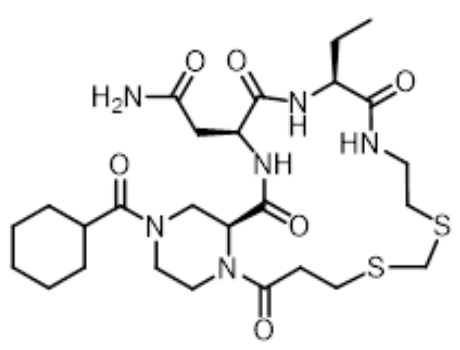	Not collected.	1. BCS (Basic Chemical Space) Screen (96 conditions)<br>2. LMB Crystallisation Screen (96 conditions)<br>3. SG1™ Screen (96 conditions)<br>4. Clear Strategy™ Screen I HT (96 conditions)<br>5. Clear Strategy™ Screen II HT (96 conditions)<br>6. NeXtal AmSO4 Suite (96 conditions)	No protein crystal.
**6**	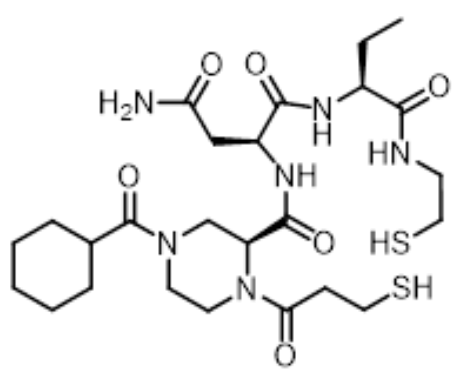	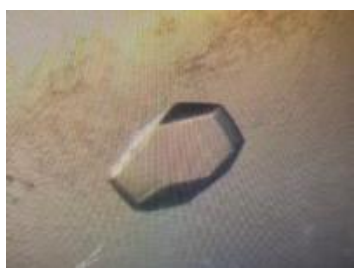	1. BCS (Basic Chemical Space) Screen (96 conditions)<br>2. SG1™ Screen (96 conditions)<br>3. Clear Strategy™ Screen I HT (96 conditions)<br>4. Clear Strategy™ Screen II HT (96 conditions)	High-quality diffraction data were collected, yielding a protein model at 1.7 Å resolution.
**7**	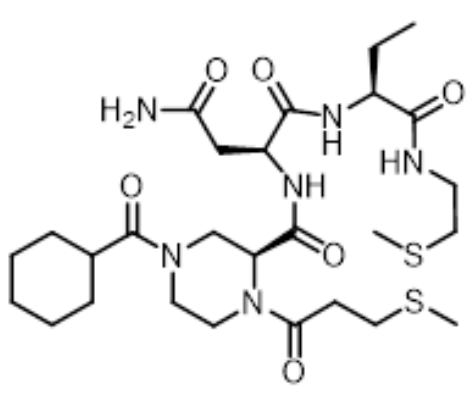	Not collected.	1. BCS (Basic Chemical Space) Screen (96 conditions)<br>2. SG1™ Screen (96 conditions)	No protein crystal.

			3. Clear Strategy™ Screen I HT (96 conditions)<br>4. Clear Strategy™ Screen II HT (96 conditions)	
**21**	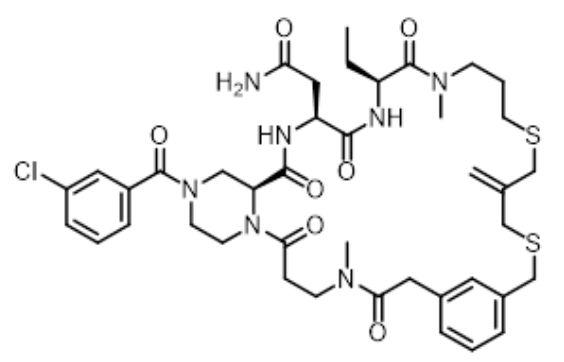	Not collected.	1. BCS (Basic Chemical Space) Screen (96 conditions)<br>2. SG1™ Screen (96 conditions)<br>3. Clear Strategy™ Screen I HT (96 conditions)<br>4. Clear Strategy™ Screen II HT (96 conditions)	No protein crystal.
**28**	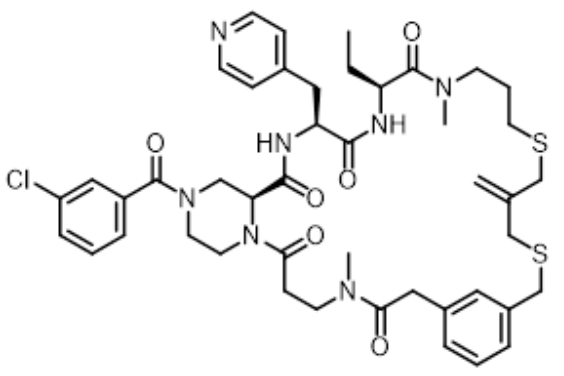	Not collected.	1. BCS (Basic Chemical Space) Screen (96 conditions)<br>2. SG1™ Screen (96 conditions)<br>3. Clear Strategy™ Screen I HT (96 conditions)<br>4. Clear Strategy™ Screen II HT (96 conditions)	High-quality diffraction data were collected, yielding a protein model at 2.35 Å resolution.
**31**	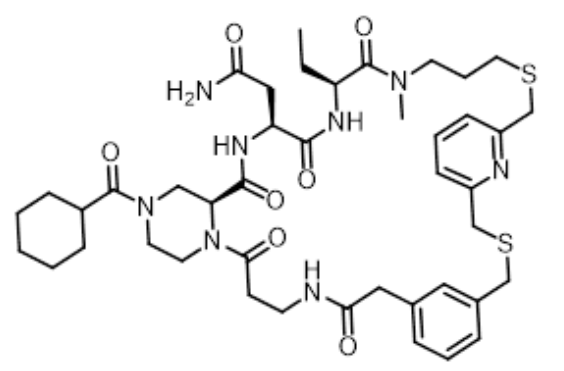	Not collected.	1. BCS (Basic Chemical Space) Screen (96 conditions)<br>2. LMB Crystallisation Screen (96 conditions)<br>3. SG1™ Screen (96 conditions)<br>4. Clear Strategy™ Screen I HT (96 conditions)<br>5. Clear Strategy™ Screen II HT (96 conditions)	High-quality diffraction data were collected, yielding a protein model at 1.95 Å resolution.
**32**	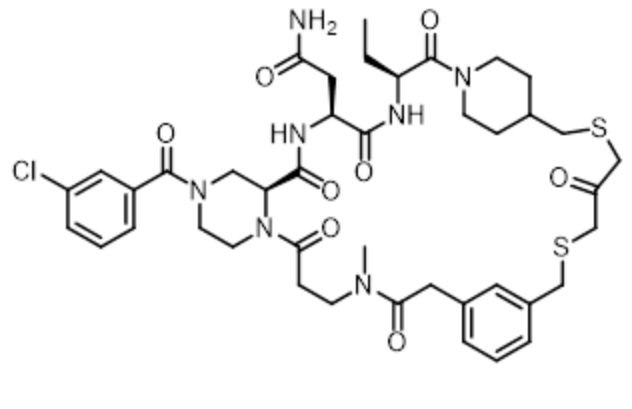	Not collected.	1. BCS (Basic Chemical Space) Screen (96 conditions)<br>2. LMB Crystallisation Screen (96 conditions)<br>3. SG1™ Screen (96 conditions)<br>4. Clear Strategy™ Screen I HT (96 conditions)<br>5. Clear Strategy™ Screen II HT (96 conditions)	High-quality diffraction data were collected, yielding a protein model at 2.33 Å resolution.

Supplementary Table 9. Interactions in X-ray structure between peptide 6 and Keap1. Atoms of the linear peptide **6** forming interactions with atoms and residues of Keap1. Interactions have distances shorter than 4.0 Å and were defined using the software ChimeraX and LIGPOLT+.

Keap1 atom / residue	Peptide 5	Distance (Å)	Interaction
NH1 / Arg 483	O39	2.81	HB
NH2 / Arg 483	O39	3.13	HB
OE1 / Gln 530	N09	2.94	HB
NE2 / Gln 530	O10	2.94	HB
OC / Ser 555	N09	3.26	HB
OC / Ser 602	O25	2.75	HB

Supplementary Table 10. Structure-activity relationship study of peptide 6. The following table show the effect of single amino acid substitutions on the binding of peptide **6** to Keap1. The binding of the crude peptides was measured once by the TF-FRET assay. Peptides showing strong binding are labeled with a number and were further characterized as purified peptide.

**Compound number**	**Amino acid mutation**	**Structure**	**Nrf2-peptide displacement (%)**
Substitution of amino acid A1			
	A2	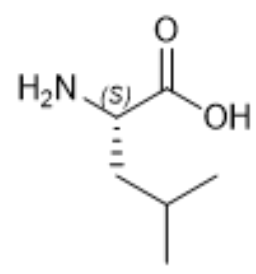	10.50
9	A6	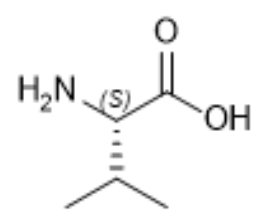	65.12
	A7	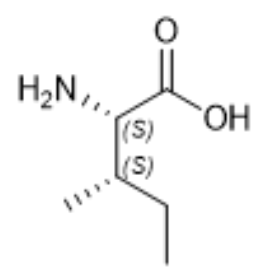	45.46
	A8	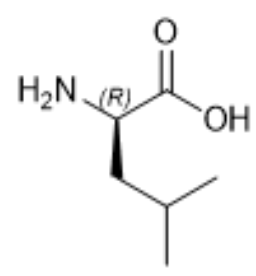	6.06
	A9	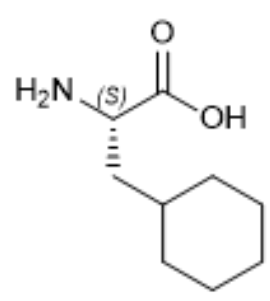	3.49
	A10	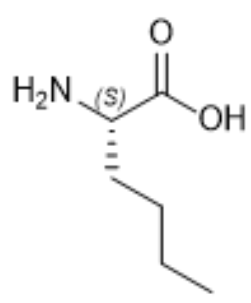	13.32

	A11	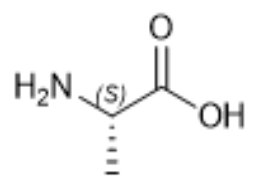	26.32
	A12	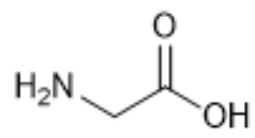	14.06
	A13	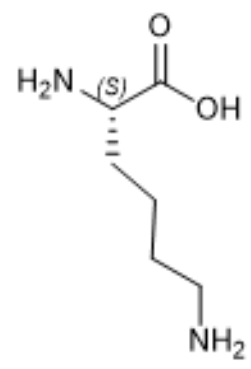	5.94
	A14	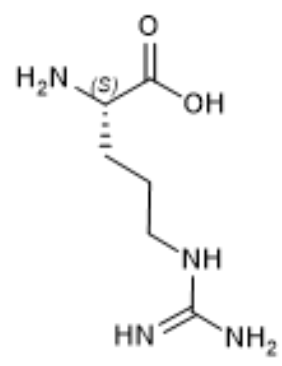	4.24
	A15	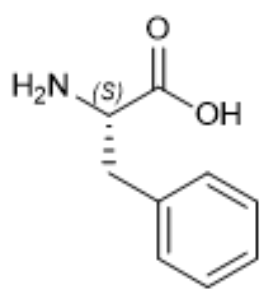	11.68
	A16	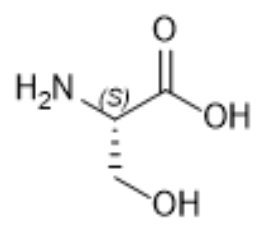	26.02
	A17	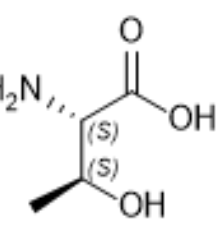	28.64
	A18	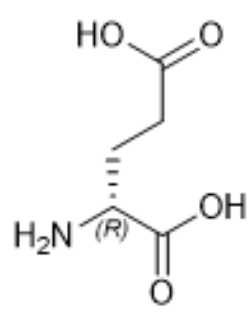	27.79
	A19	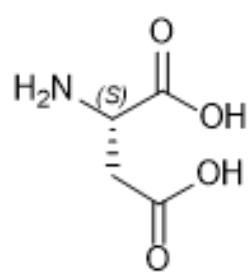	23.52

	A20	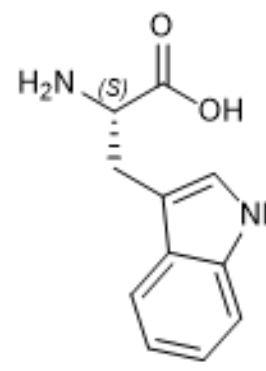	23.97
	A21	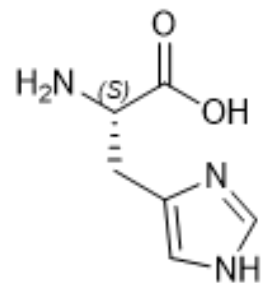	13.93
	A22	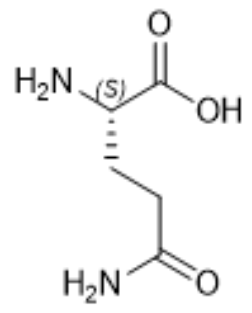	7.89
	A23	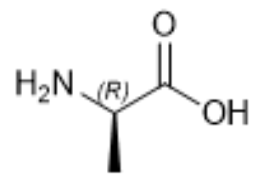	7.01
	A24	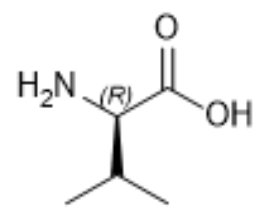	8.84
	A25	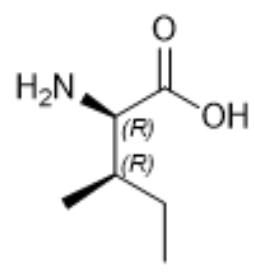	5.88
	A26	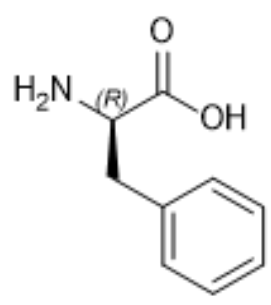	7.18
	A27	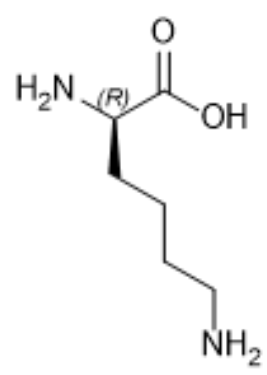	4.48
	A28	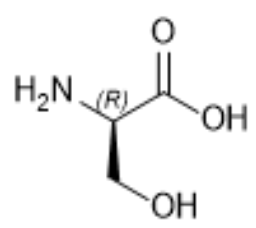	8.32

	A29	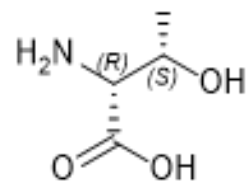	5.36
	A30	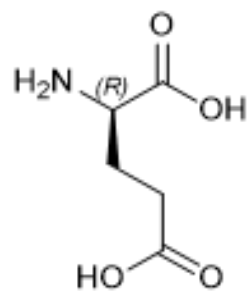	44.08
10	A31	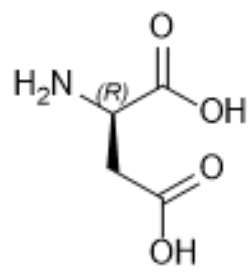	70.63
	A32	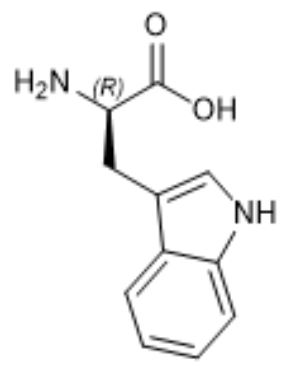	14.71
	A33	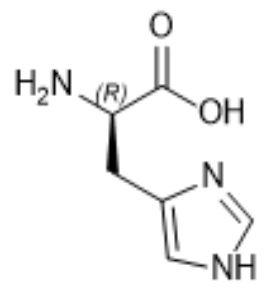	6.24
	A34	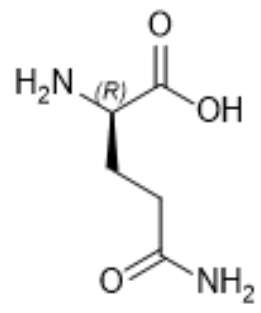	7.26
	A35	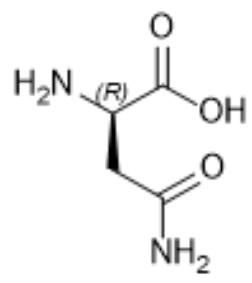	8.38
	A36	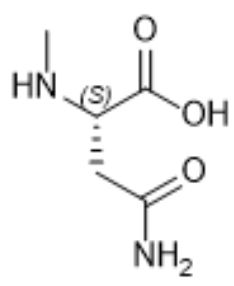	4.52
	A37	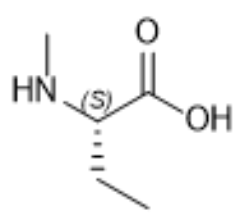	10.36

	A38	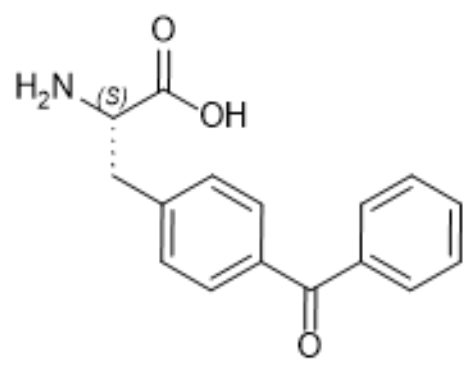	13.38
	A39	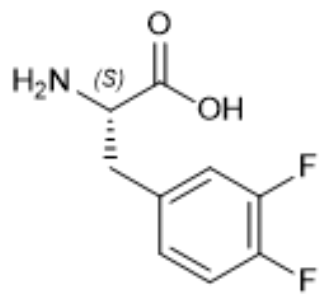	10.71
	A40	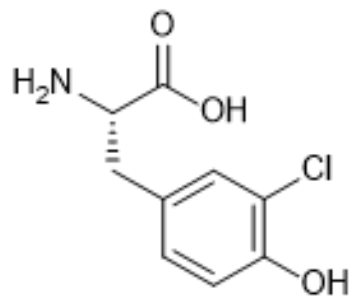	27.25
	A41	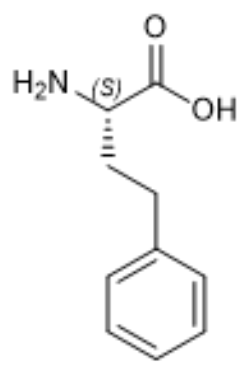	7.40
	A42	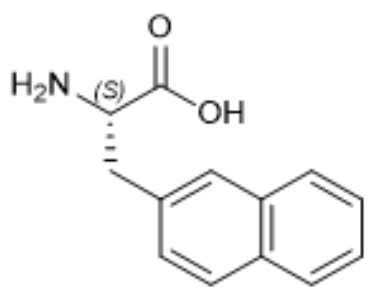	8.55
	A43	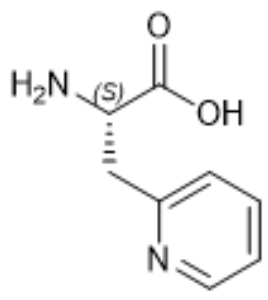	10.23
	A44	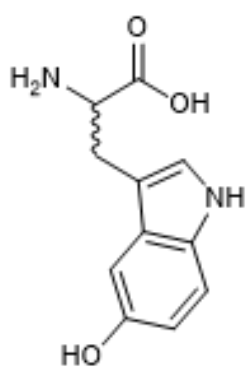	32.35
	A45	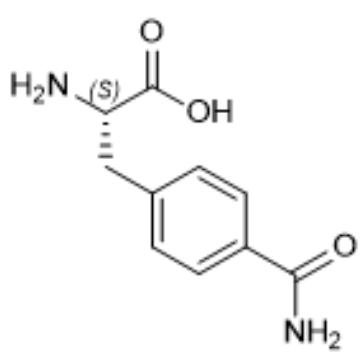	17.85
	A46	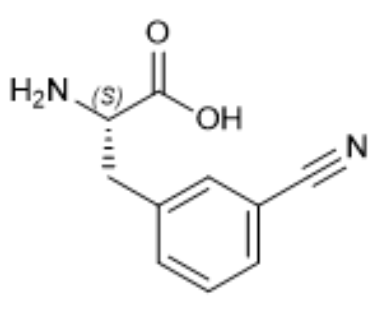	9.82

	A47	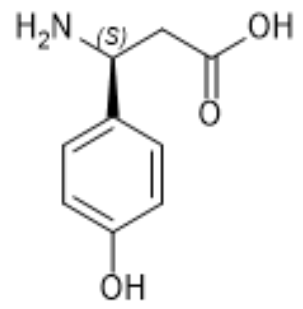	33.43
	A48	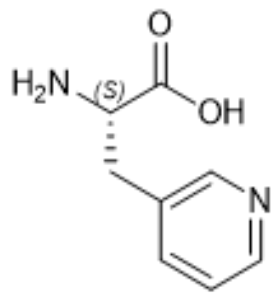	10.69
	A49	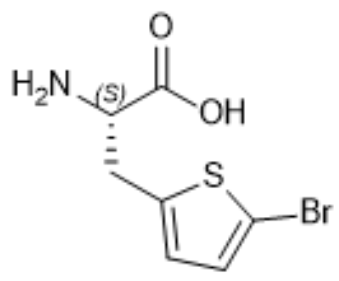	10.49
	A50	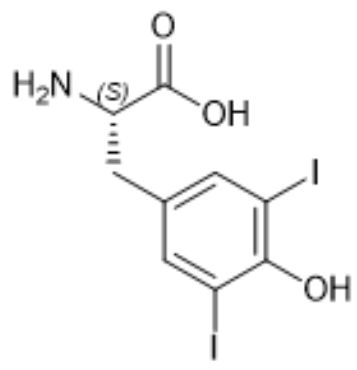	4.17
	A51	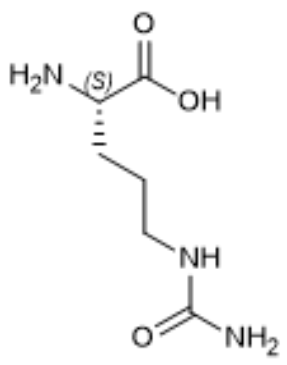	4.38
	A52	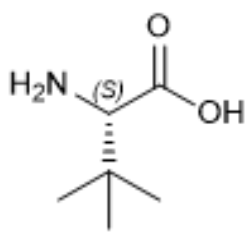	19.31
	A53	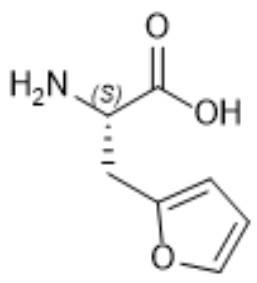	12.02
	A54	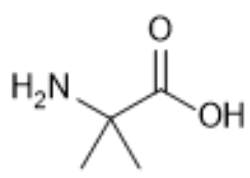	12.94
	A55	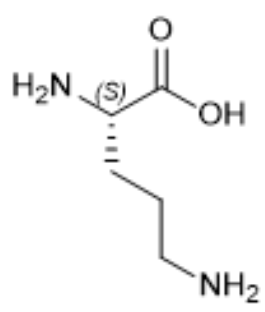	6.94

	A56	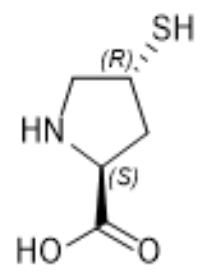	17.12
	A57	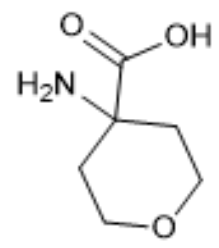	6.77
	A58	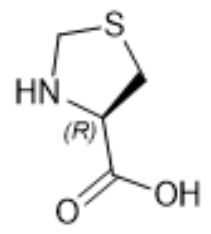	5.62
11	A59	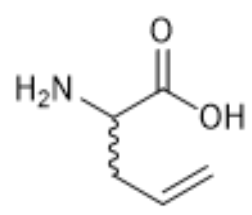	71.95
	A60	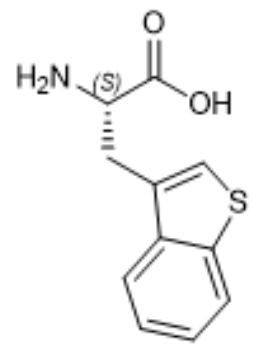	9.88
	A61	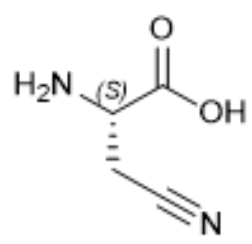	2.53
	A62	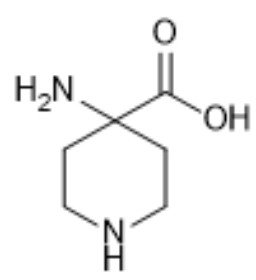	4.48
	A63	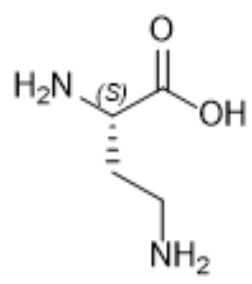	4.07
	A64	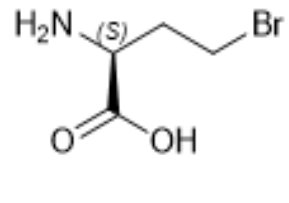	5.54

	A65	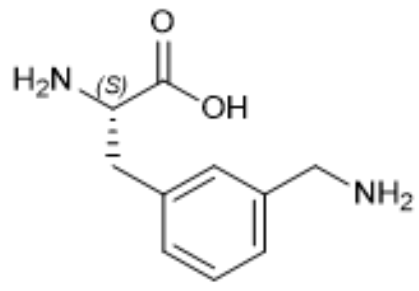	9.28
	A66	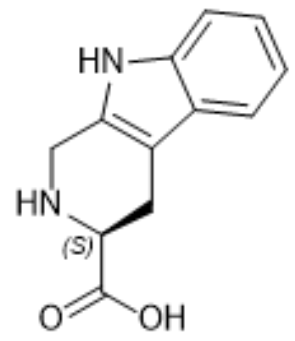	8.36
	A67	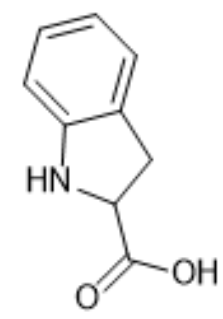	2.58
	A68	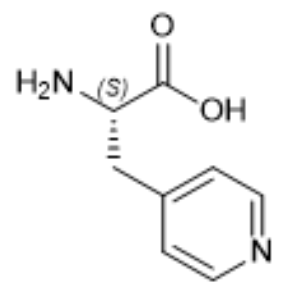	11.27
	A69	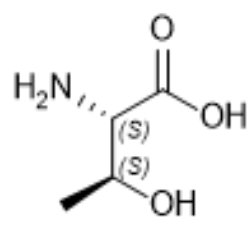	15.56
	A70	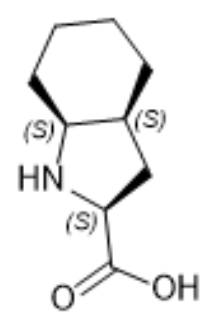	6.22
	A71	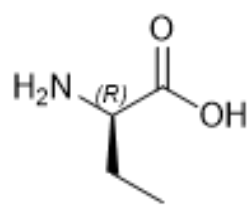	9.84
	A72	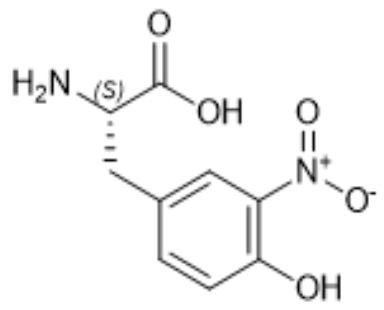	43.85
	A73	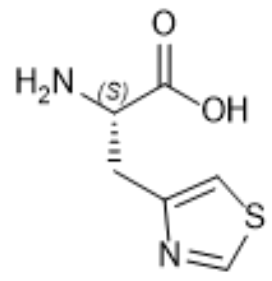	8.92

	A74	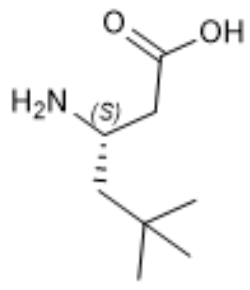	2.89
	A75	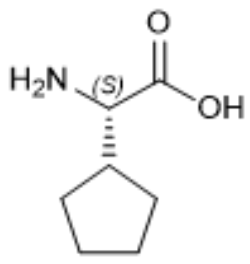	29.32
	A76	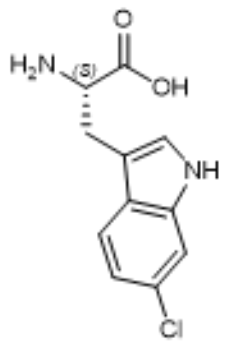	9.17
	A77	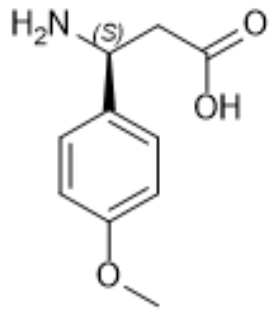	26.05
	A78	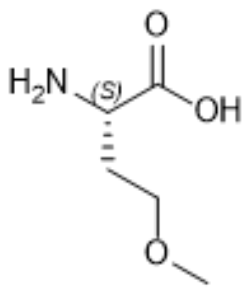	9.76
	A79	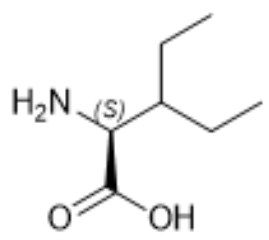	16.32
	A80	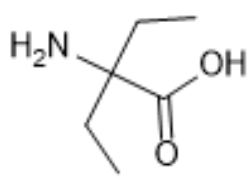	6.38
	A81	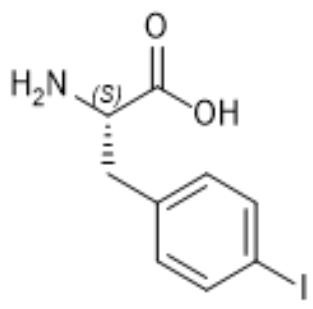	11.40
	A82	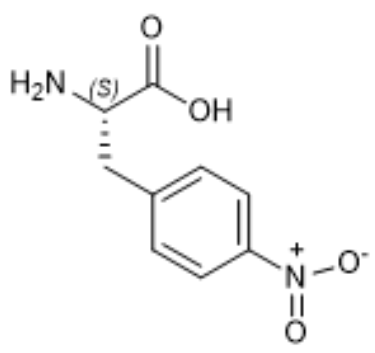	17.04

	A83	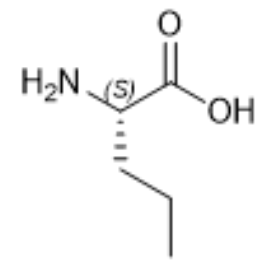	35.66
	A84	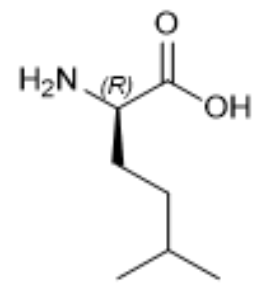	5.64
	A85	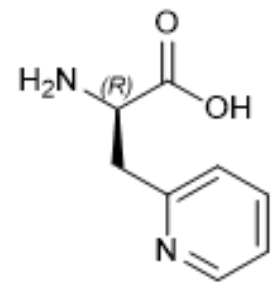	6.13
	A86	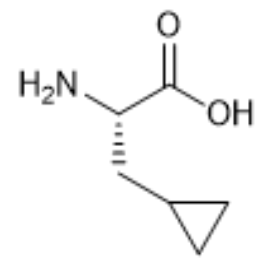	11.66
	A87	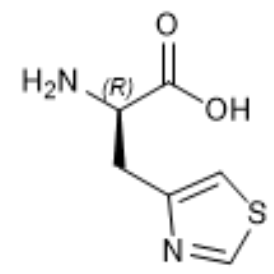	7.40
	A88	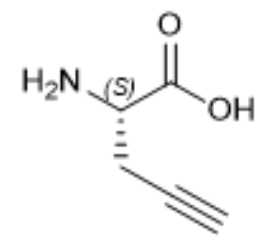	17.05
Substitution of amino acid X13			
	X12	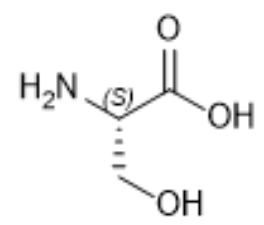	6.84
	X14	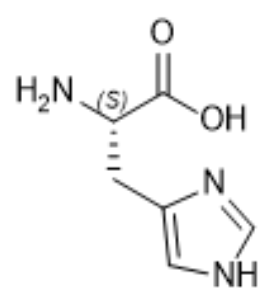	24.00

	X15	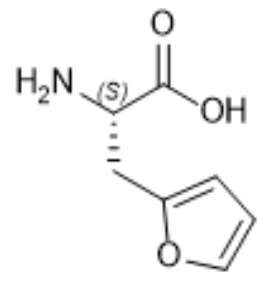	12.25
	X17	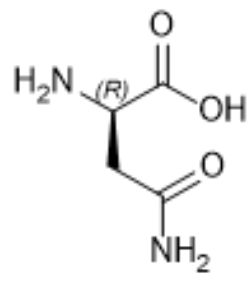	4.48
	X18	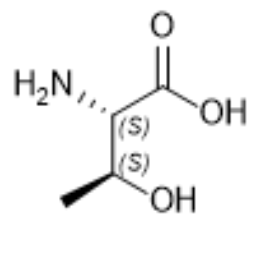	6.86
	X19	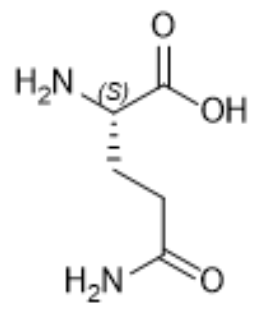	11.80
	X20	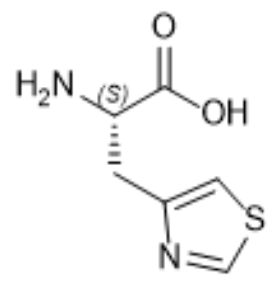	17.33
	X21	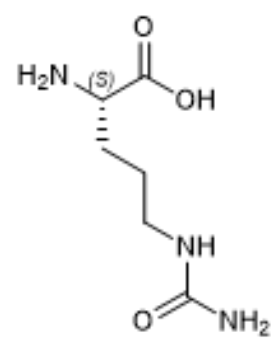	6.17
	X23	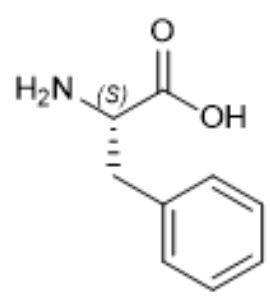	14.19
	X28	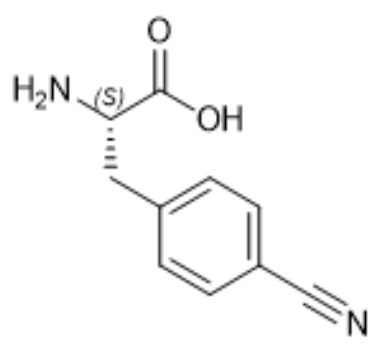	16.99
	X29	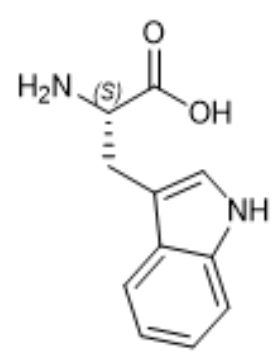	12.85

	X33	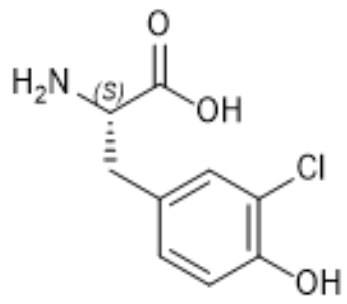	54.05
	X35	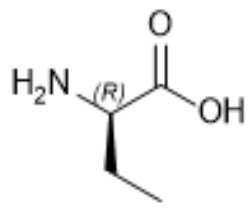	2.60
	X37	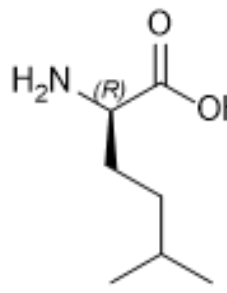	5.40
	X38	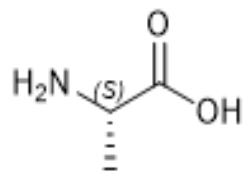	5.63
	X41	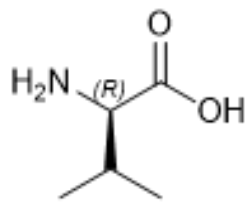	5.95
	X42	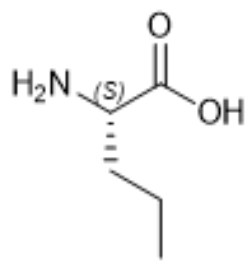	7.30
	X43	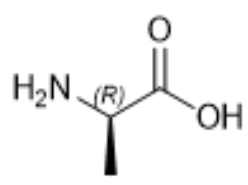	4.59
	X44	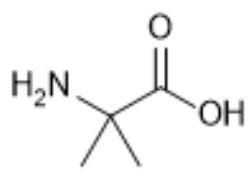	4.15
	X46	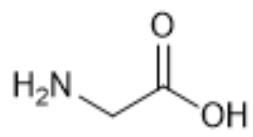	6.23

	X47	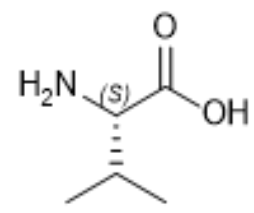	2.35
	X48	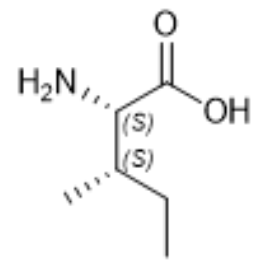	3.38
	X49	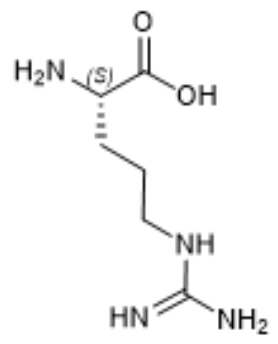	3.13
	X50	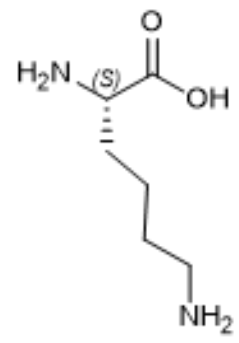	4.10
	X51	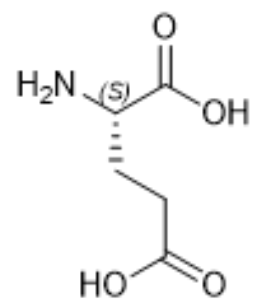	45.74
	X52	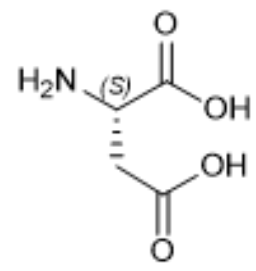	19.63
	X53	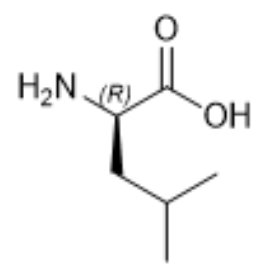	3.72
	X54	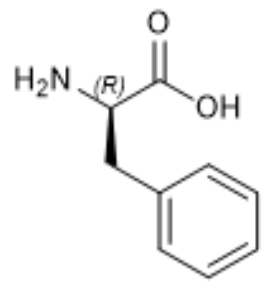	2.70
	X55	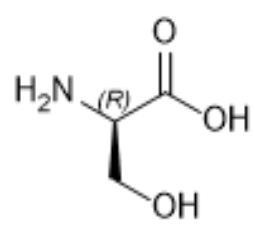	4.13

	X56	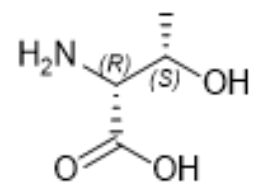	5.40
	X57	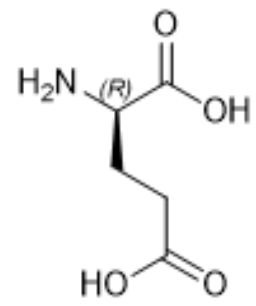	10.13
	X58	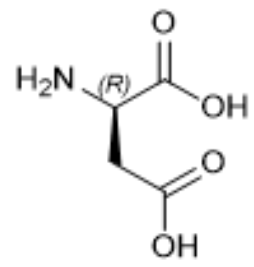	13.10
	X59	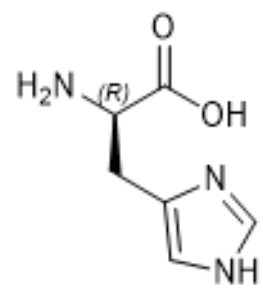	7.24
	X60	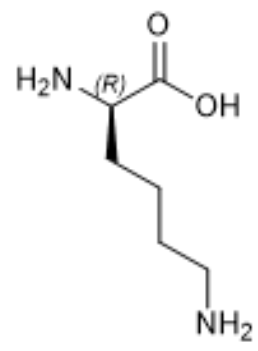	4.03
	X61	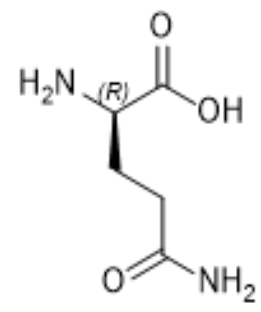	3.53
	X62	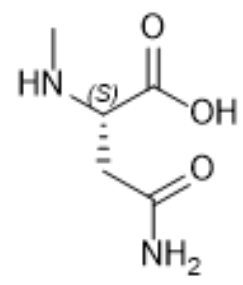	9.14
	X63	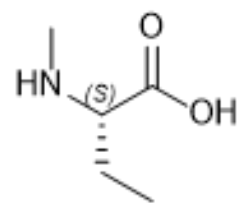	6.47
	X64	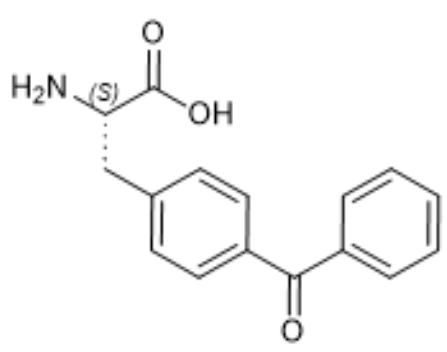	7.81

	X65	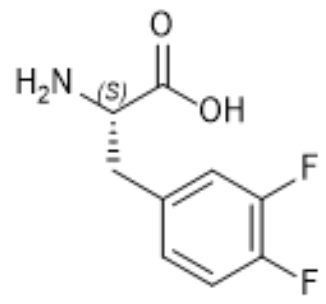	23.56
	X66	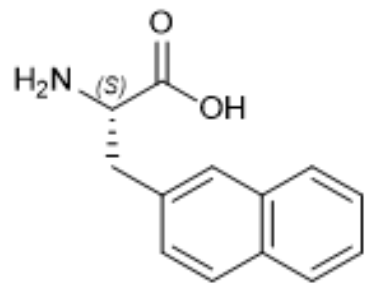	8.03
	X67	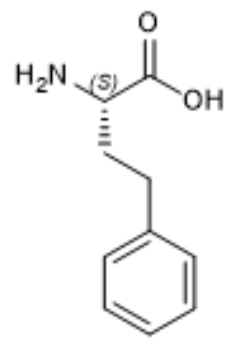	7.80
	X68	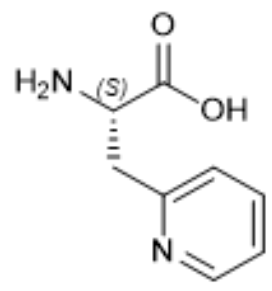	10.17
	X69	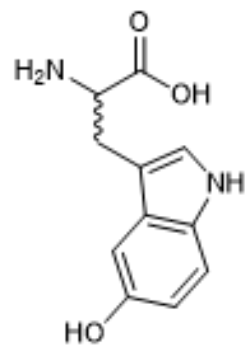	25.57
	X70	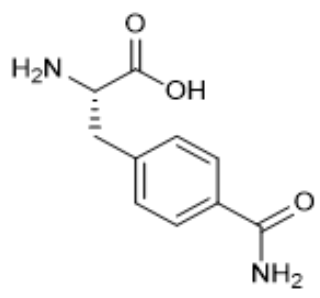	20.73
	X71	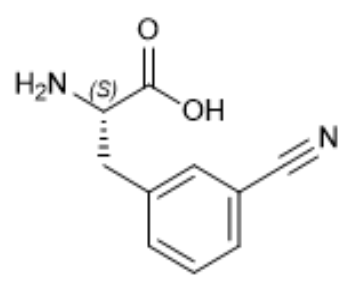	29.87
	X72	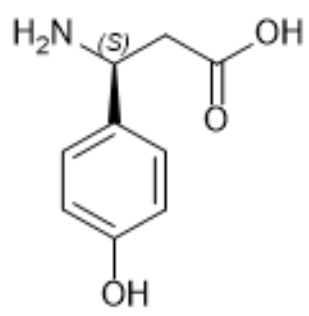	5.71
	X73	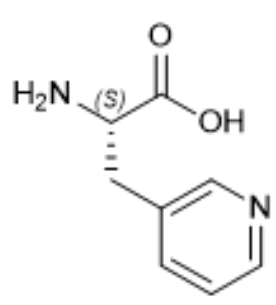	23.92

	X74	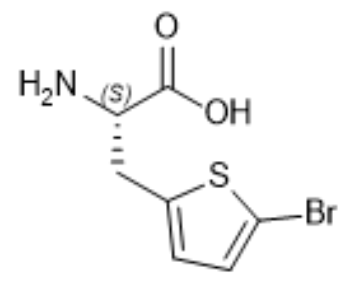	12.56
	X75	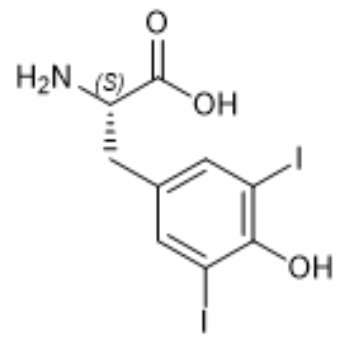	5.47
	X76	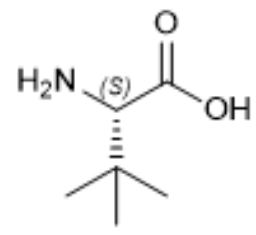	6.02
	X77	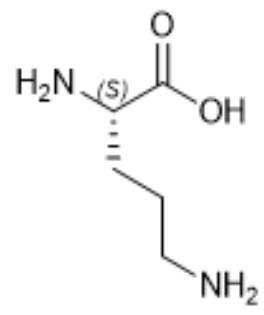	8.09
	X78	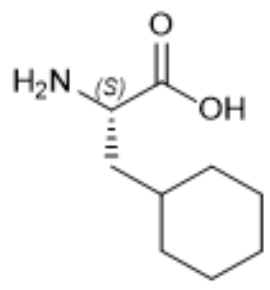	7.48
	X79	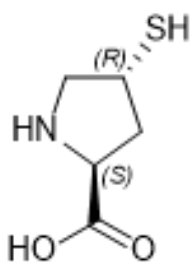	19.63
	X80	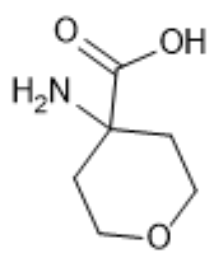	4.91
	X81	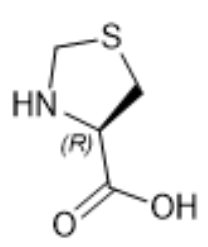	8.25
	X82	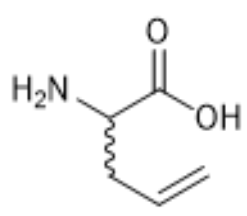	6.78

	X83	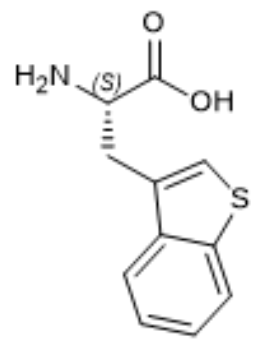	10.99
	X84	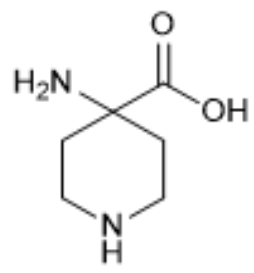	4.06
	X85	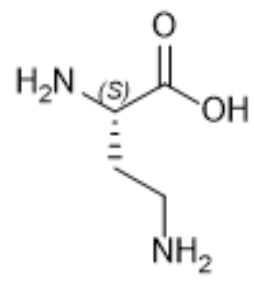	5.16
	X86	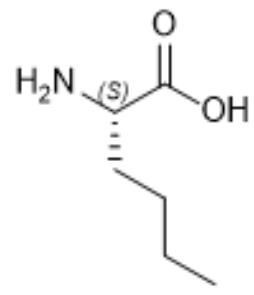	6.44
	X87	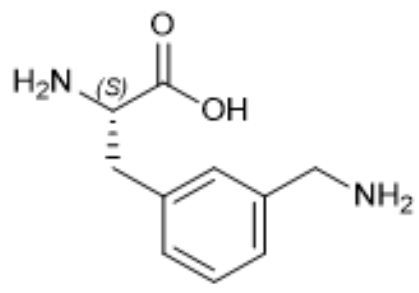	8.53
	X88	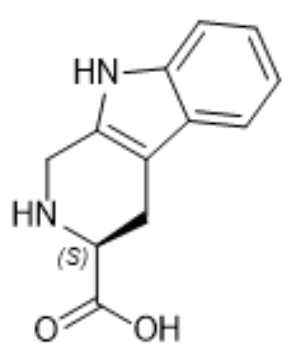	6.59
	X89	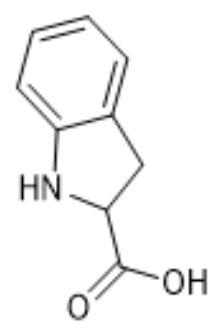	6.38
12	X90	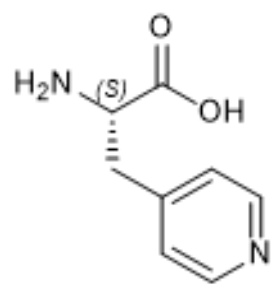	63.27
	X91	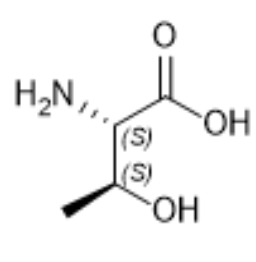	6.11

	X92	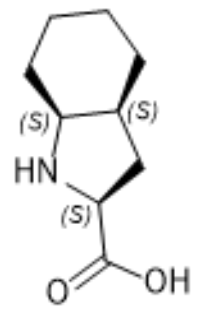	46.78
	X93	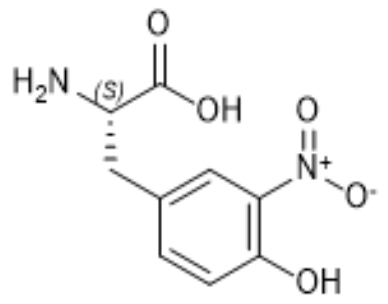	38.52
	X94	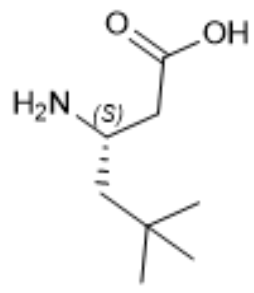	5.66
	X95	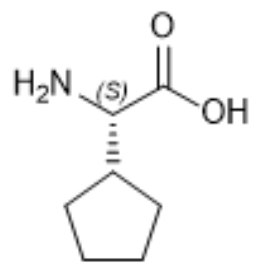	4.77
	X96	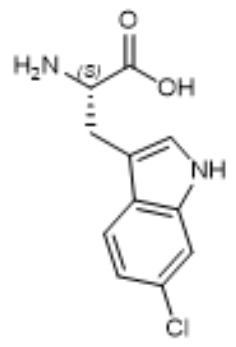	10.07
	X97	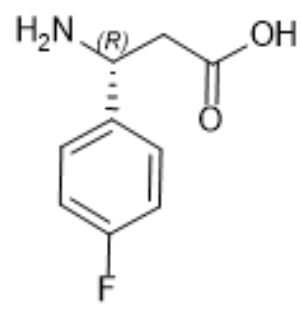	8.90
	X98	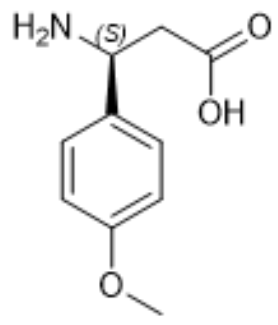	6.72
	X99	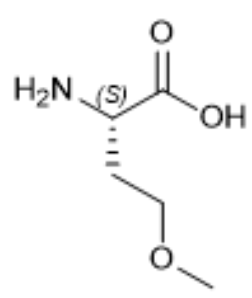	10.15
	X100	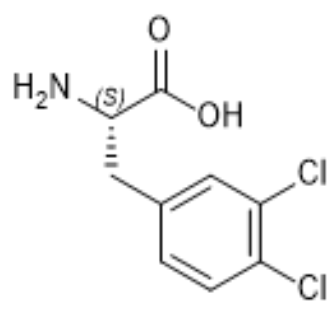	16.68

	X101	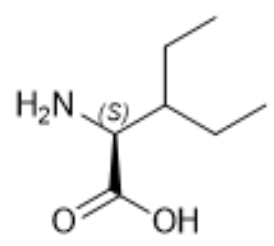	7.45
	X102	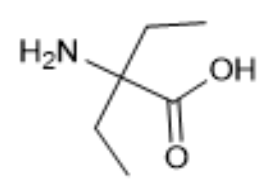	5.19
	X103	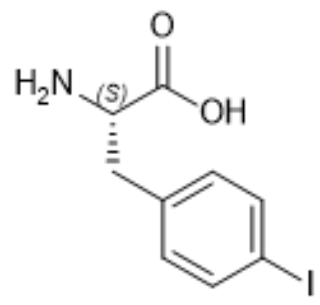	10.01
	X104	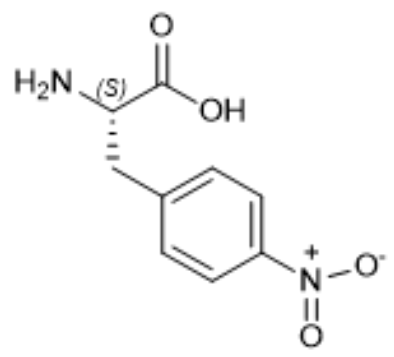	25.37
	X105	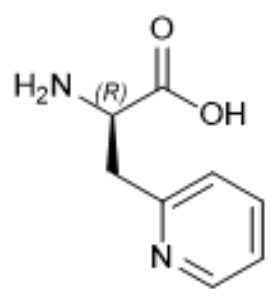	5.01
	X106	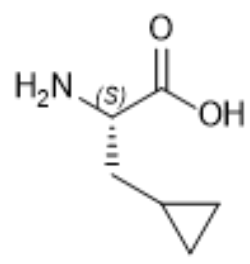	5.73
	X107	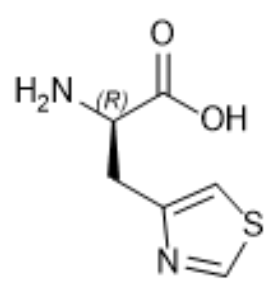	3.93
	X108	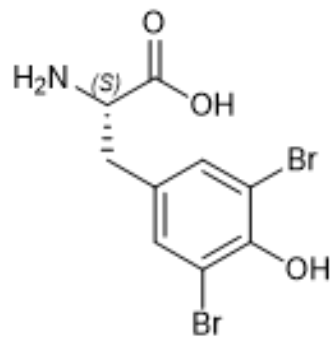	47.21
	X109	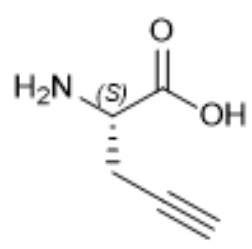	8.46

Substitution of amino acid D4			
	D1	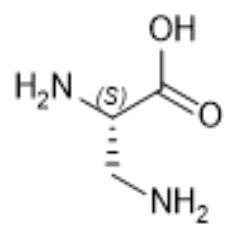	3.00
	D2	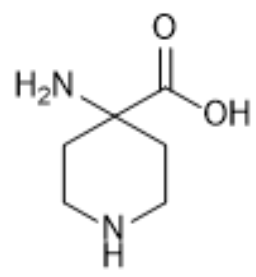	3.80
	D6	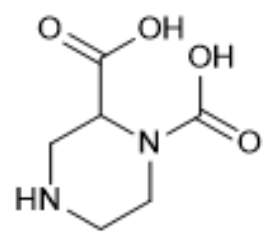	5.60
	D11	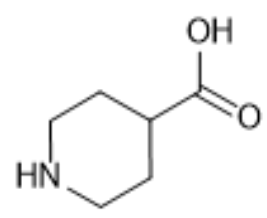	2.37
	D12	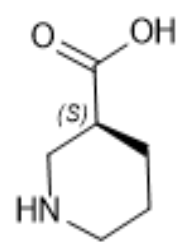	4.77
	D13	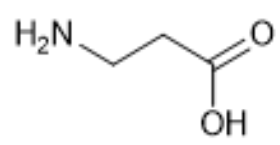	2.63
	D14	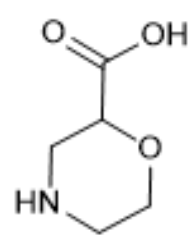	11.03
	D15	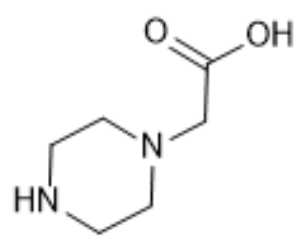	3.95

	D16	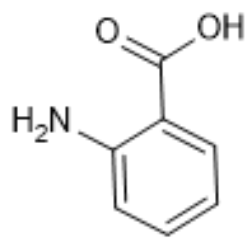	0.78
	D17	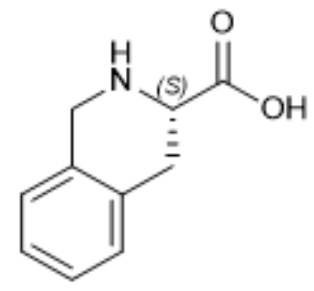	3.03
	D18	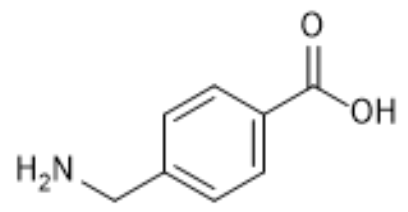	3.39
	D19	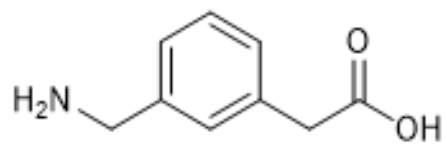	3.51
	D20	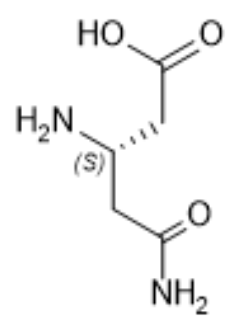	3.43
	D21	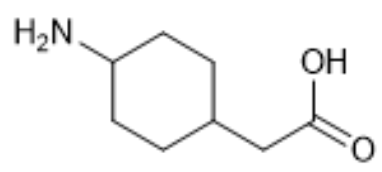	4.55
	D22	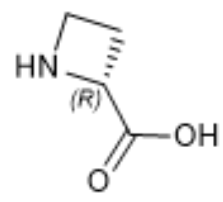	1.17
	D23	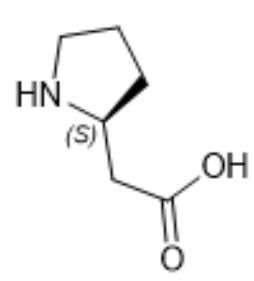	4.18
	D24	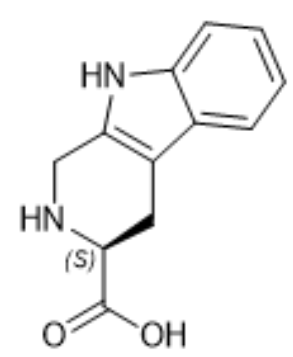	5.42

	D25	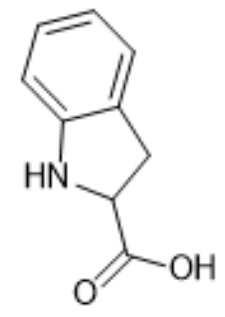	6.29
	D26	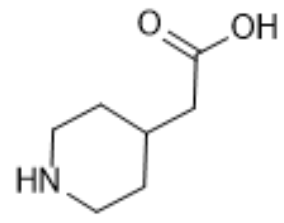	3.33
	D27	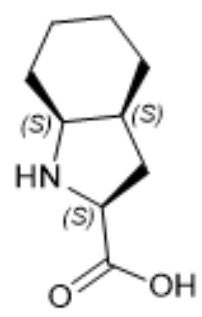	2.55
	D28	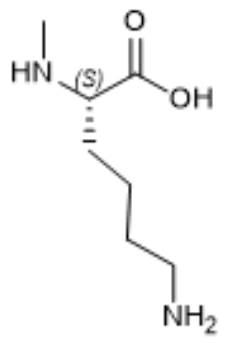	3.36
	D29	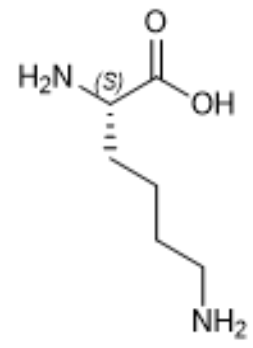	3.19
	D30	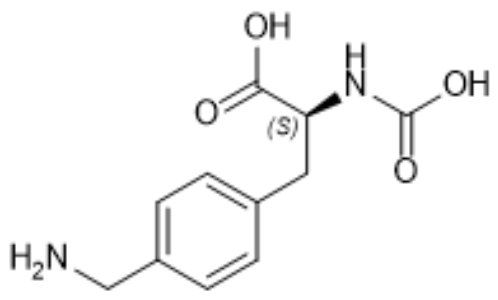	4.71
	D31	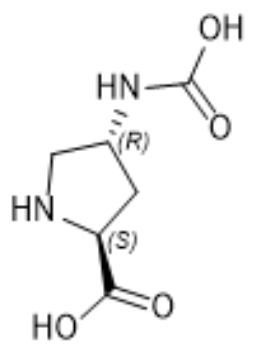	1.75
	D32	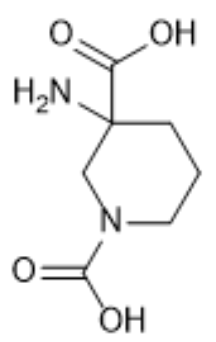	14.20
	D33	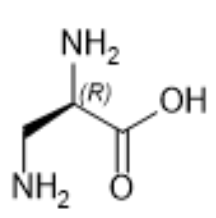	4.63

	D34	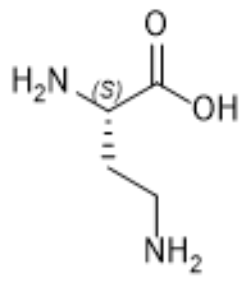	3.33
	D35	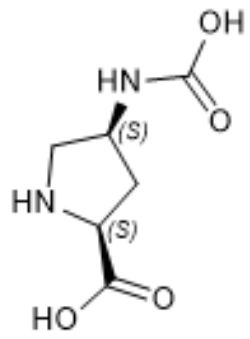	4.06
Substitution of carboxylic acid C9			
	C1	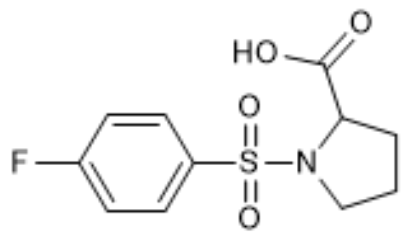	6.42
	C2	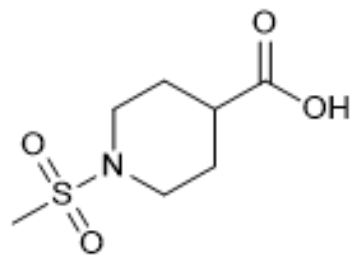	5.16
	C3	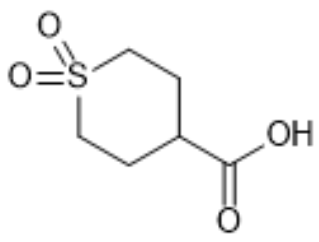	5.36
	C4	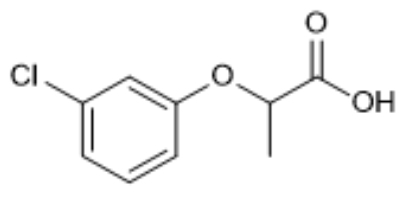	3.59
	C7	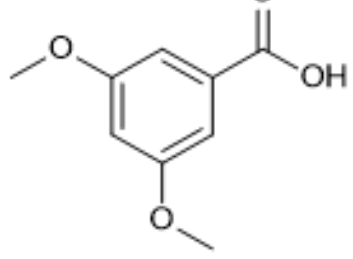	3.04
	C8	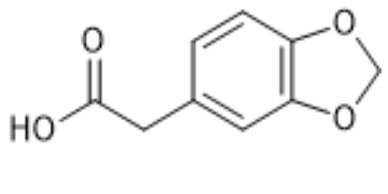	7.23

13	C12	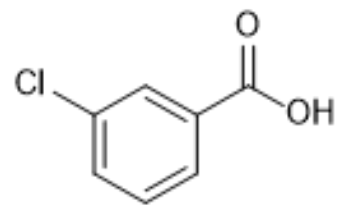	88.81
	C13	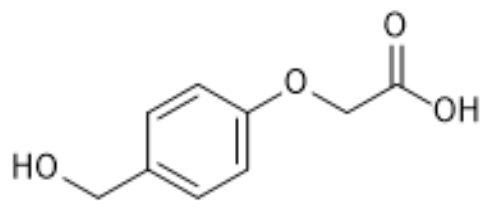	5.70
	C14	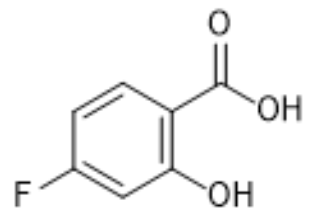	17.37
	C14	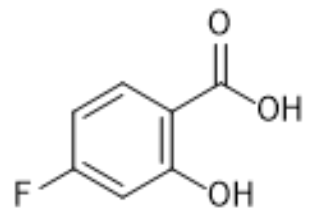	5.10
	C15	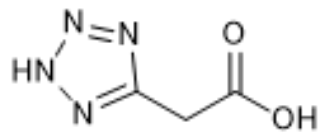	4.77
	C16	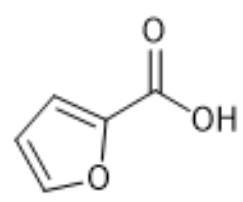	10.91
14	C17	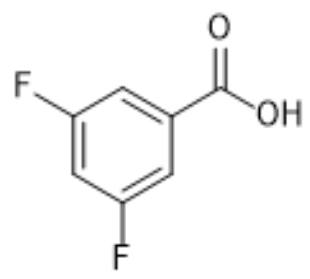	84.22
	C18	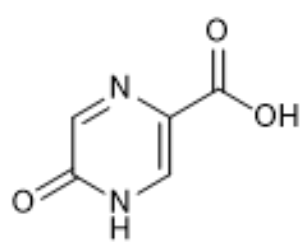	7.22
	C19	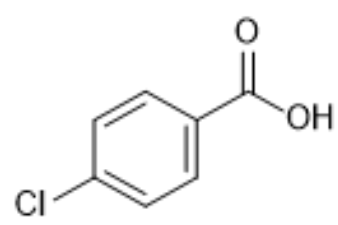	17.02

	C20	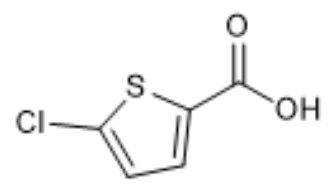	59.48
	C21	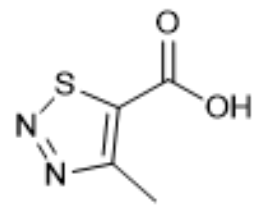	2.54
	C22	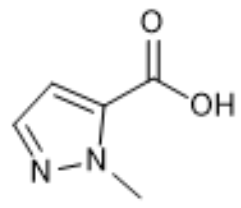	4.64
	C23	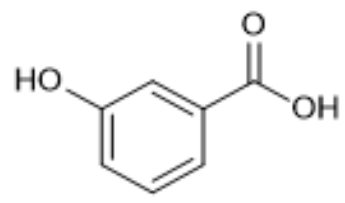	21.37
	C24	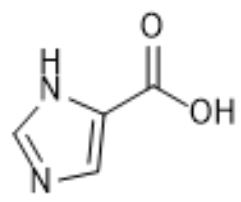	3.60
	C25	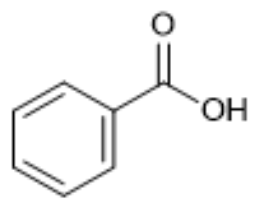	53.70
	C26	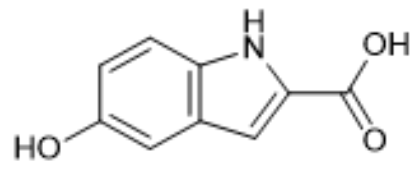	7.53
	C27	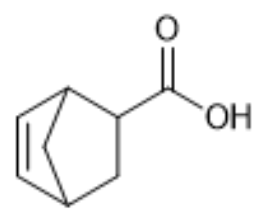	2.12
	C28	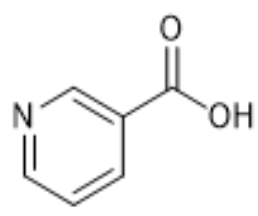	13.66

	C29	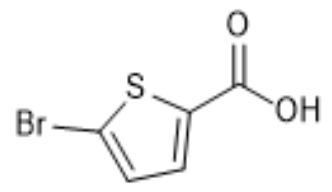	32.02
	C30	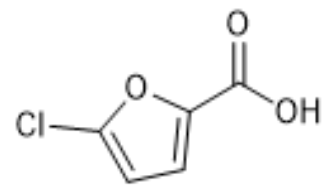	28.11
	C31	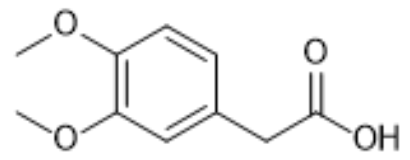	2.41
	C32	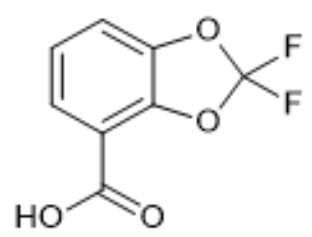	2.54
	C33	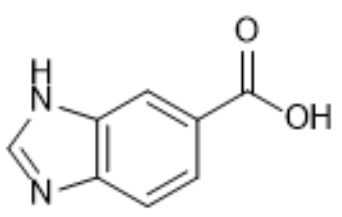	6.16
	C34	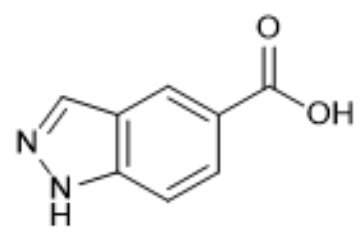	8.88
	C35	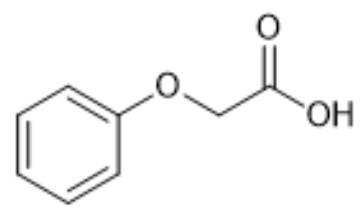	9.84
	C36	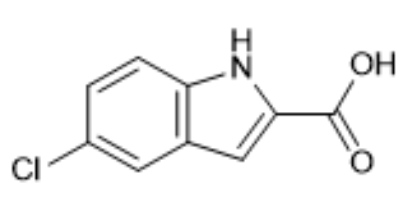	1.90
	C37	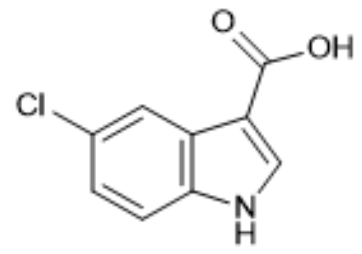	8.81

	C38	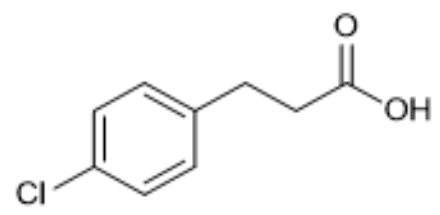	4.19
	C39	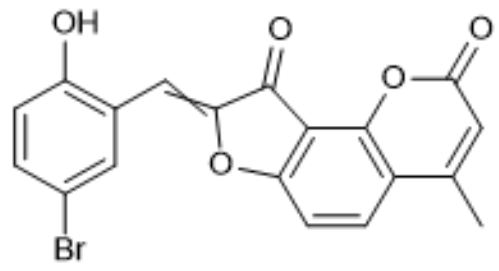	14.44
	C40	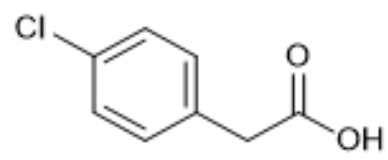	7.39
	C41	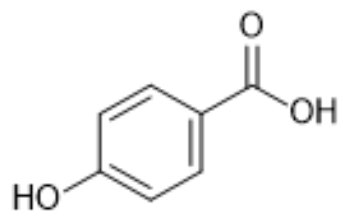	42.41
	C42	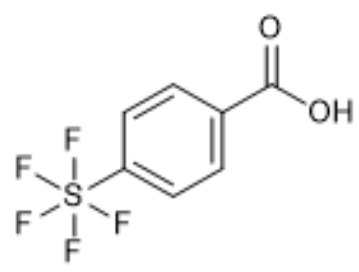	8.26
	C43	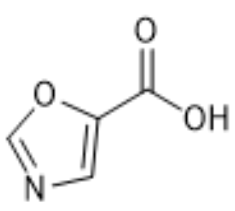	3.87
	C44	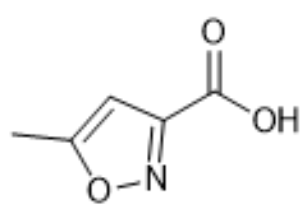	3.36
	C45	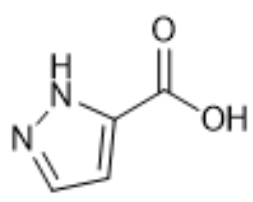	4.92
	C46	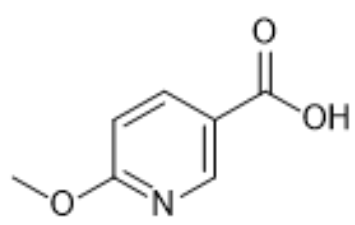	4.33

	C47	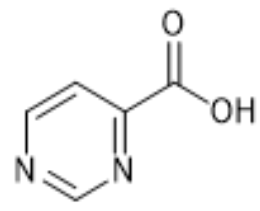	2.73
	C48	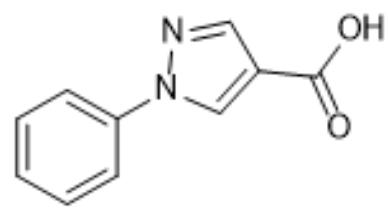	7.39
	C49	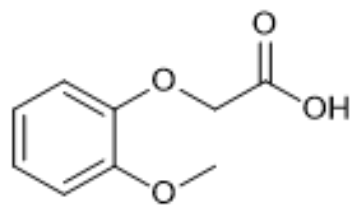	5.72
	C50	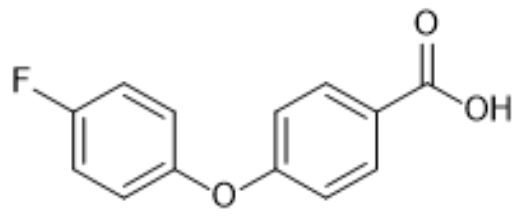	5.45
	C51	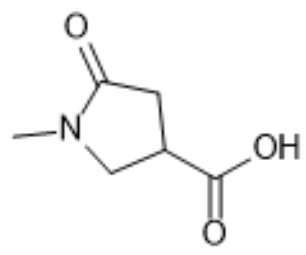	0.70
	C52	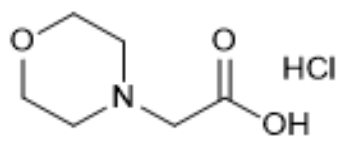	3.08
	C53	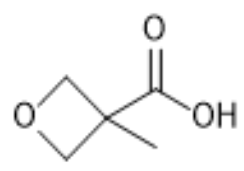	2.56
	C54	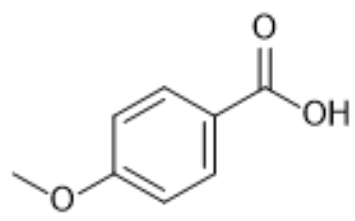	6.82
	C55	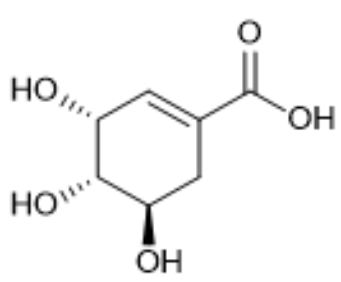	2.82

	C56	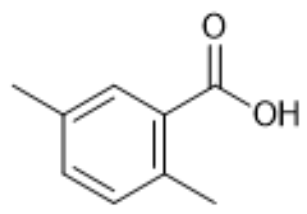	4.75
	C57	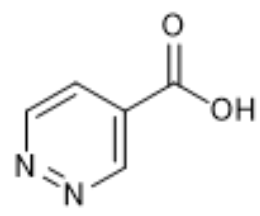	2.20
	C58	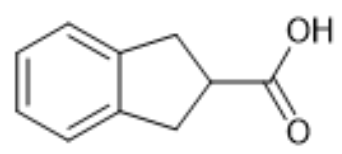	8.40
	C59	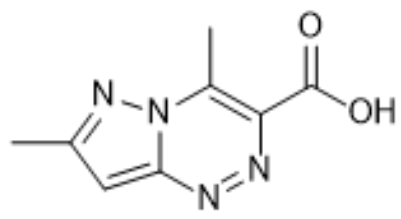	1.18
	C60	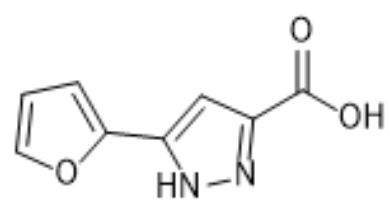	6.58
	C61	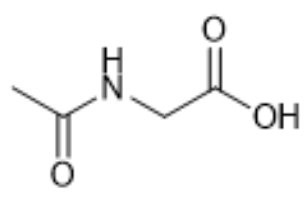	3.17
	C62	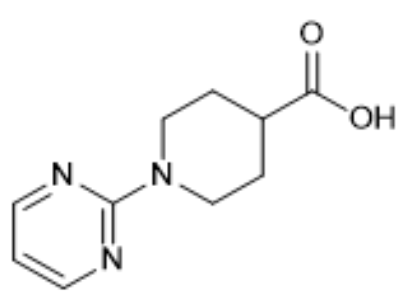	1.97
	C63	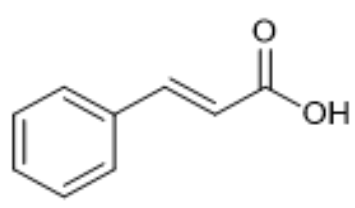	4.62
	C64	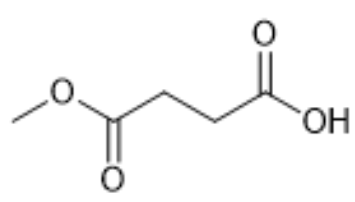	3.96

	C65	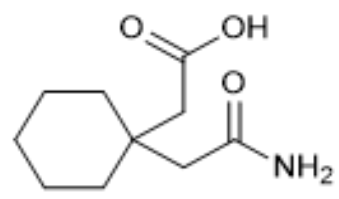	5.84
	C66	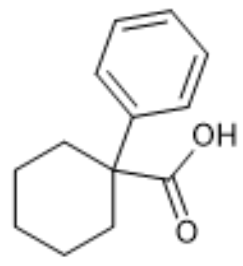	0.68
	C67	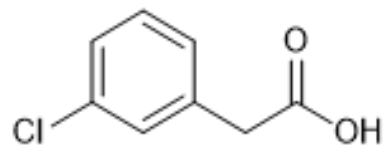	7.17
	C68	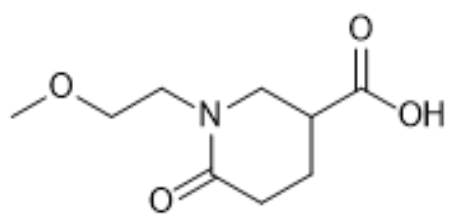	2.09
	C69	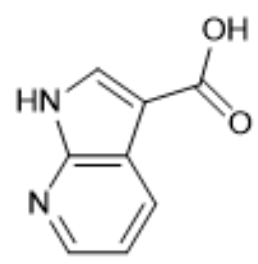	20.91
	C70	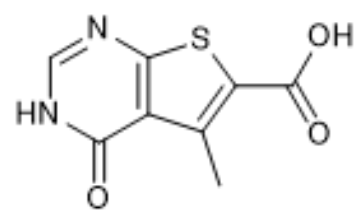	2.15
	C71	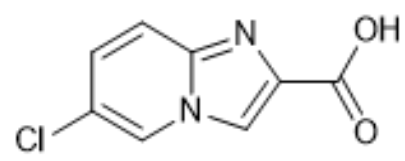	6.25
	C72	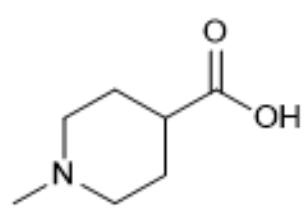	2.50
	C73	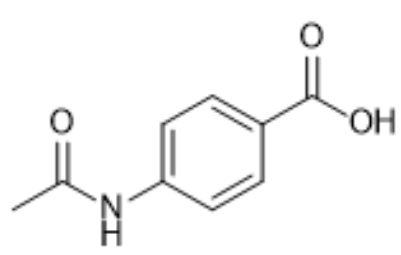	4.01

	C74	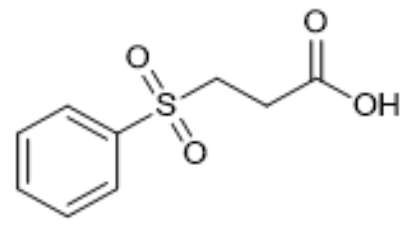	6.62
	C75	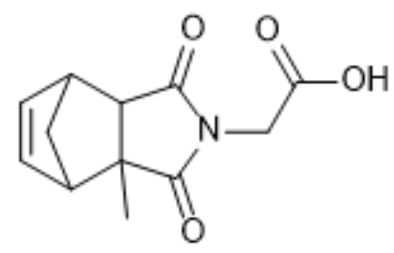	2.01
	C76	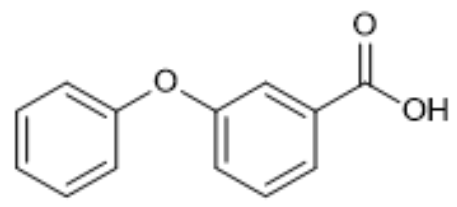	31.86
	C77	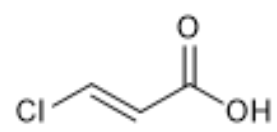	2.59
	C78	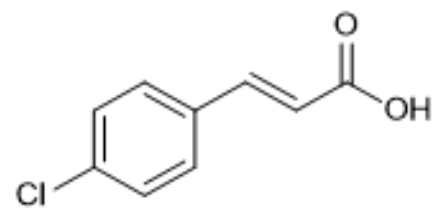	7.32
	C79	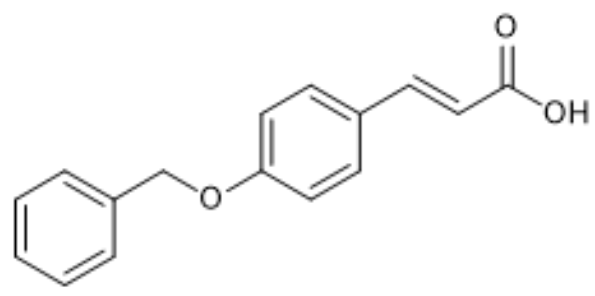	2.45
	C80	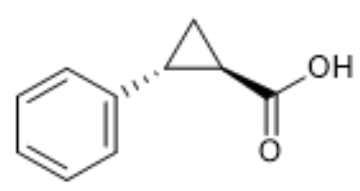	6.37
	C81	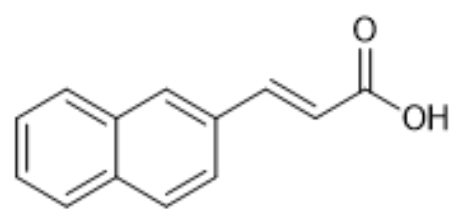	8.30
	C82	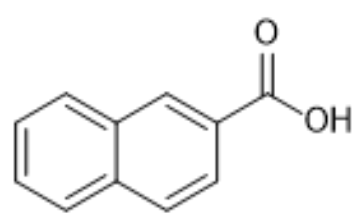	8.20

	C83	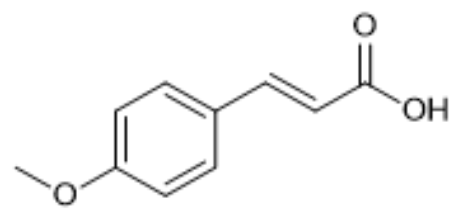	5.21
	C84	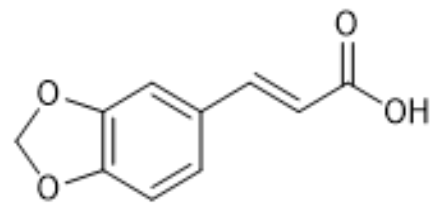	9.01
	C85	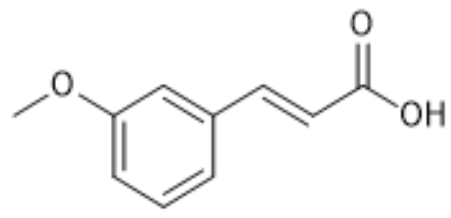	8.62
	C86	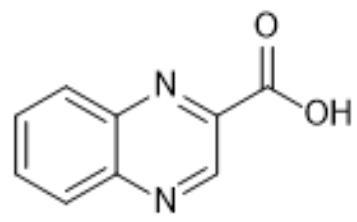	5.01
	C87	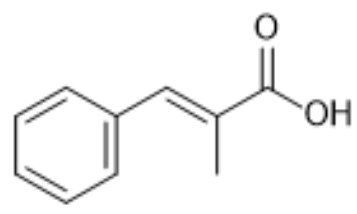	2.75
	C88	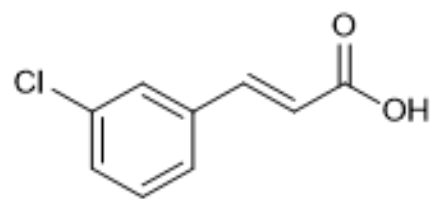	4.58
	C89	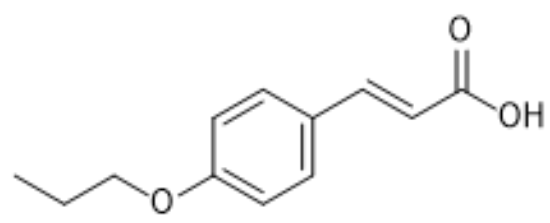	3.64
	C90	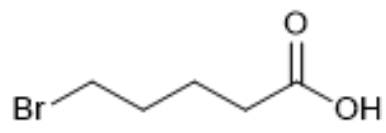	3.84
	C91	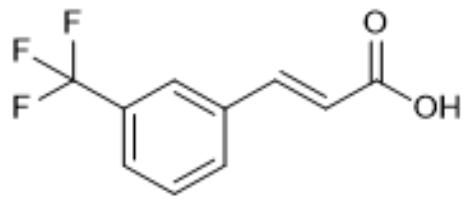	4.35

	C92	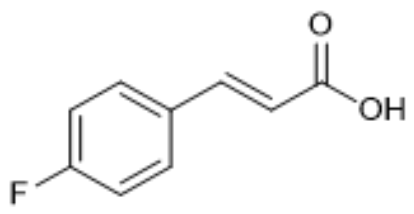	5.39
	C93	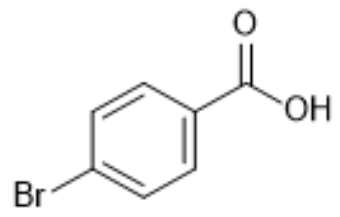	16.71
	C94	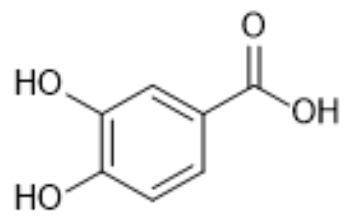	5.13
	C95	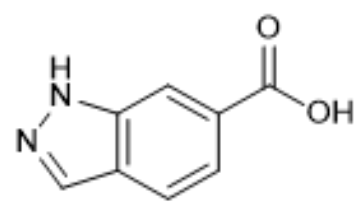	4.19
	C96	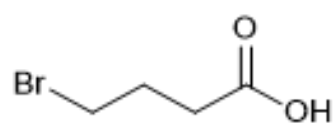	1.06
15	C97	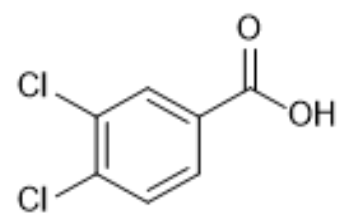	74.40
	C98	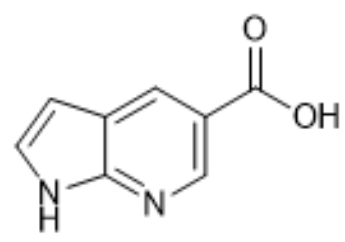	7.14
	C99	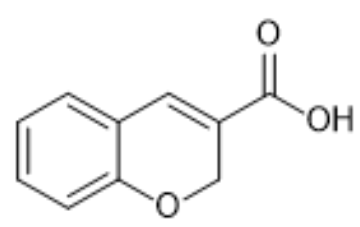	6.69
	C100	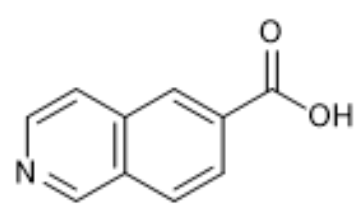	2.92

	C101	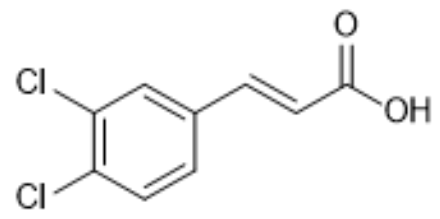	4.43
	C102	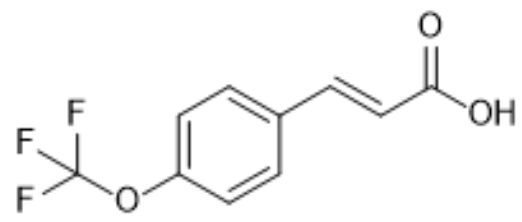	1.18
	C103	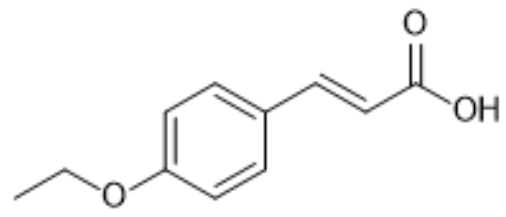	5.42
	C104	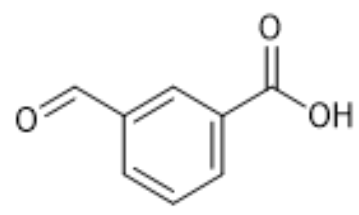	6.92
	C105	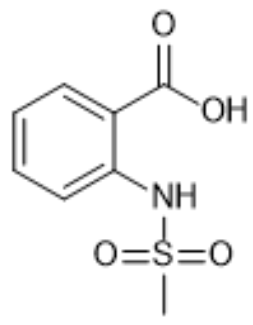	3.62
	C106	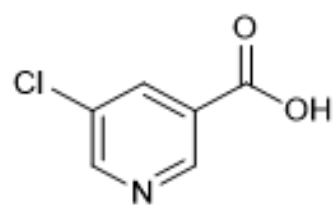	38.70
	C107	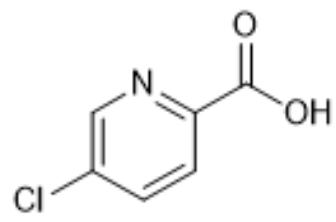	6.85
	C108	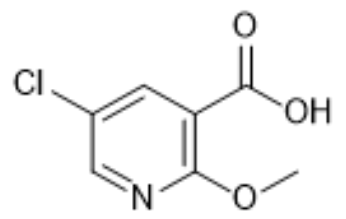	7.32
	C109	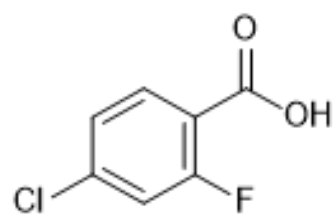	6.50

	C110	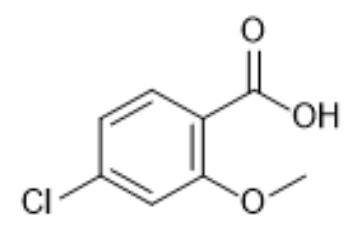	2.53
	C111	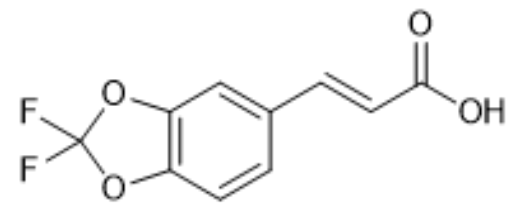	2.52
	C112	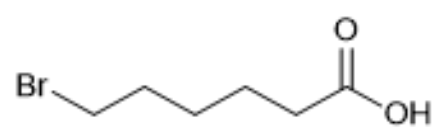	5.32
	C113	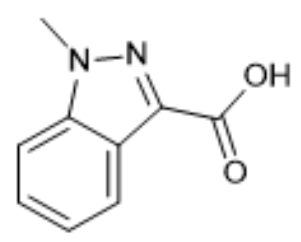	6.24
	C114	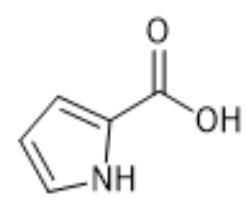	5.78
	C115	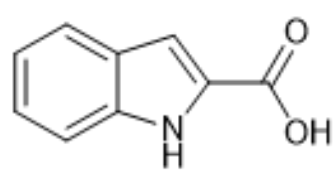	6.71
	C116	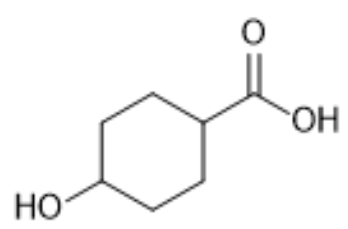	2.11
	C117	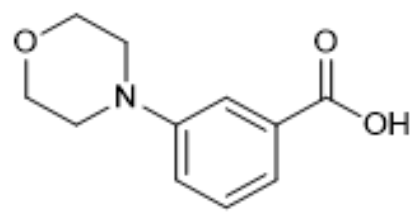	3.23
	C118	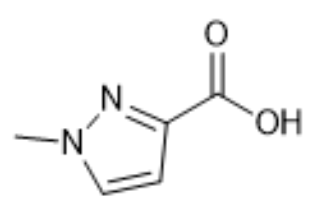	2.27

	C119	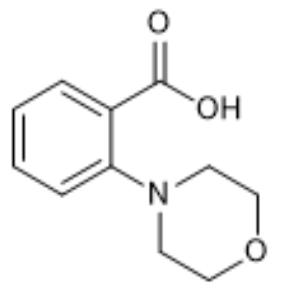	5.07
	C120	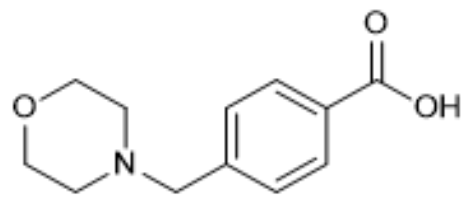	2.93
	C121	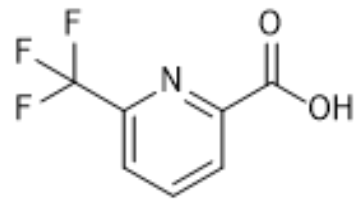	1.97
	C122	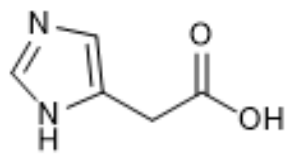	4.12
	C123	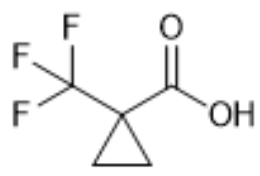	4.21
	C124	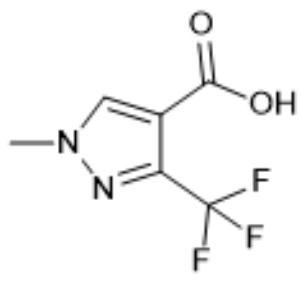	5.04
	C125	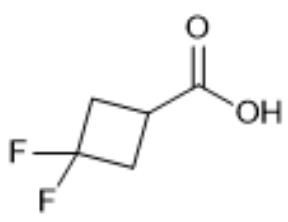	13.61
	C126	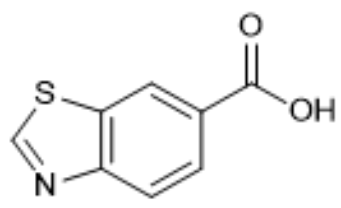	10.05
	C127	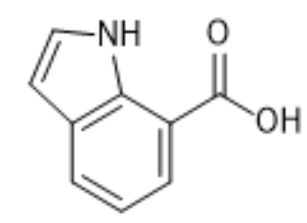	2.39

	C128	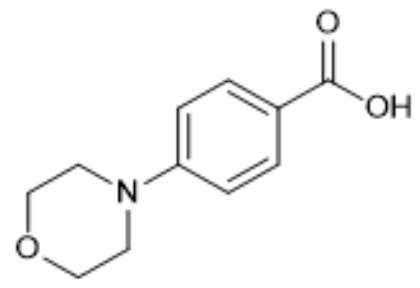	4.24
	C129	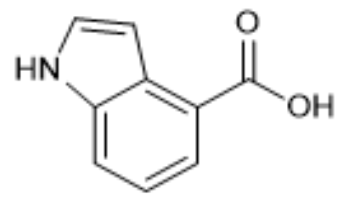	3.85
	C130	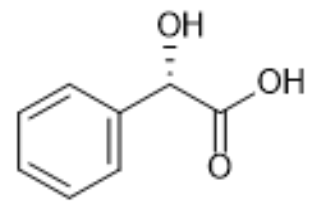	3.72
	C131	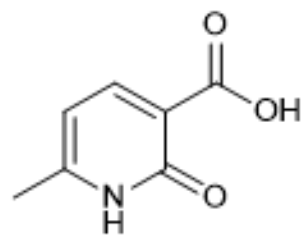	1.58
	C132	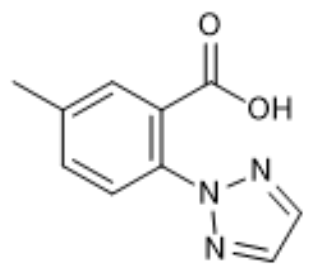	6.39
	C133	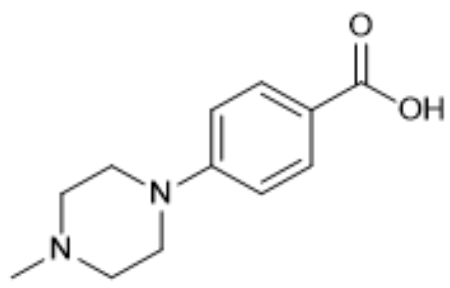	0.62
	C134	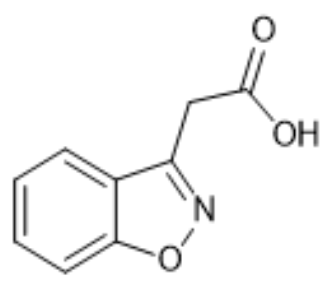	4.37
	C135	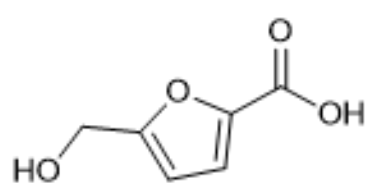	5.85
	C136	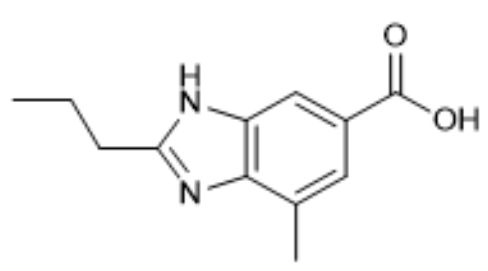	4.40

	C137	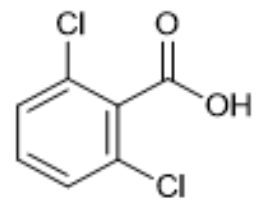	6.66
	C138	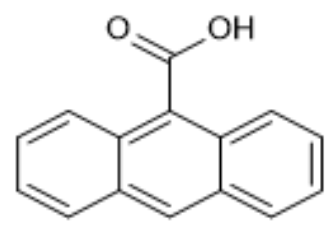	3.75
	C139	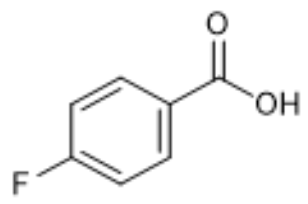	26.88
	C140	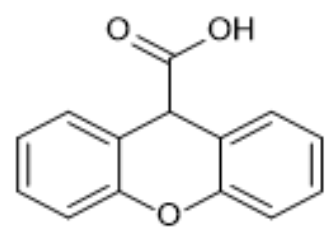	4.00
	C141	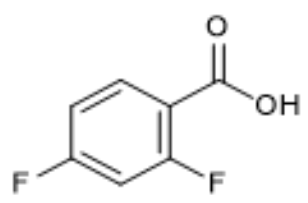	9.06
	C142	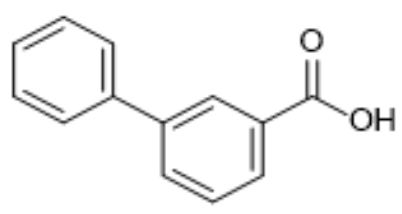	55.33
Substitution of carboxylic acid C9			
8	C11	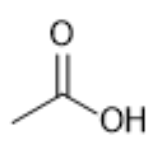	78.70
	C1	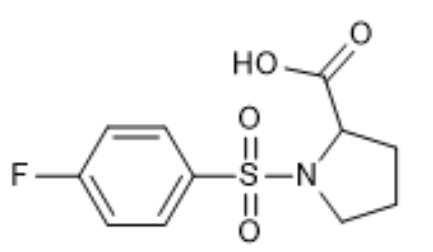	57.37

	C2	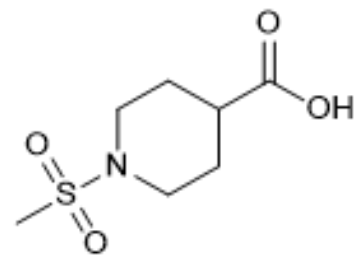	52.91
	C3	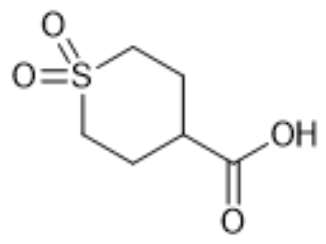	38.37
	C4	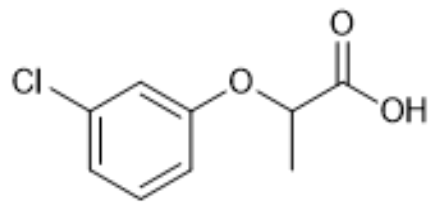	65.95
	C7	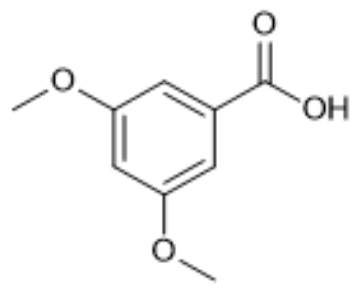	55.25
16	C8	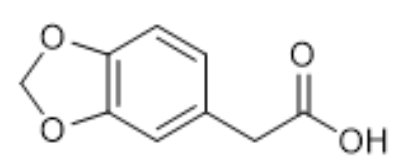	85.48
	C9	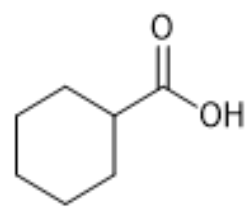	58.66
	C12	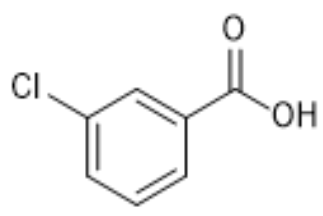	77.38
	C13	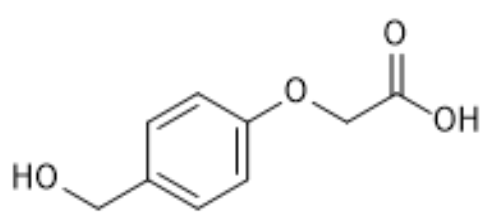	66.69
	C14	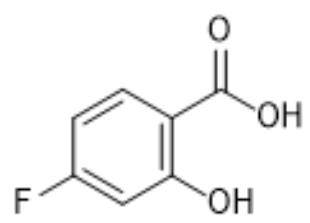	42.33

	C15	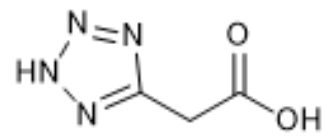	39.24
	C16	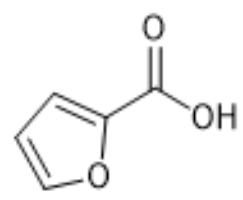	56.91
	C17	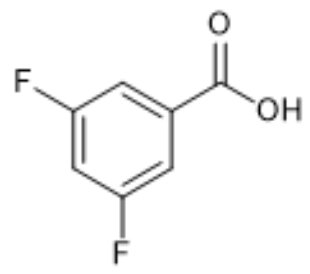	73.78
	C18	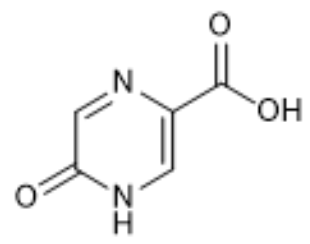	41.57
	C19	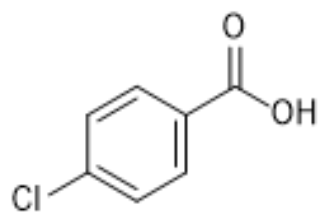	51.72
	C20	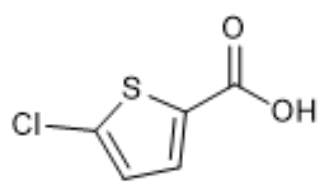	47.97
	C21	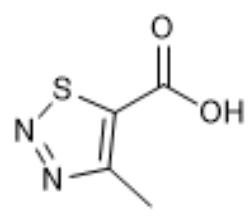	58.17
	C22	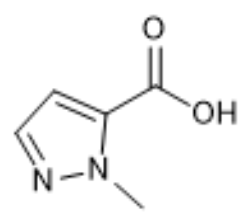	39.67
	C23	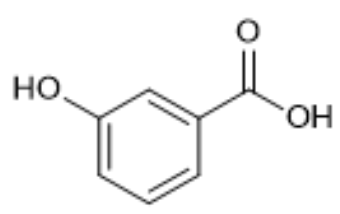	68.34

	C24	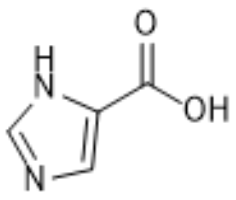	58.36
	C25	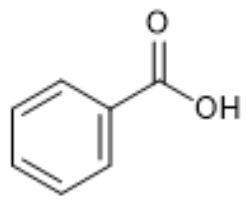	67.32
	C26	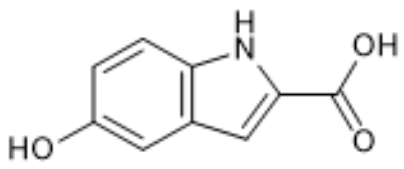	55.52
	C27	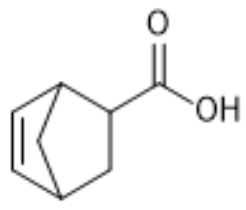	37.14
	C28	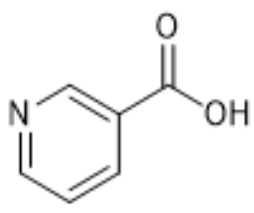	58.20
	C29	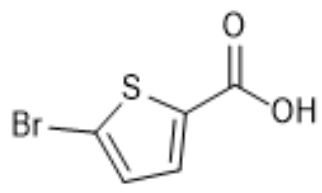	49.20
	C30	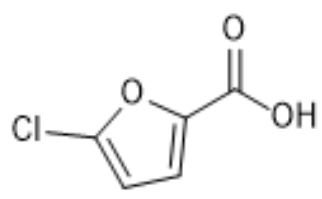	62.76
	C31	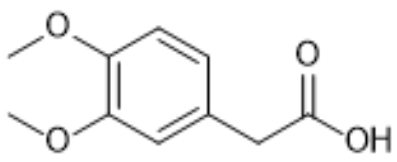	81.92
	C32	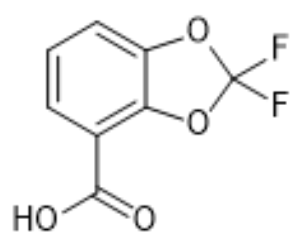	60.16

	C33	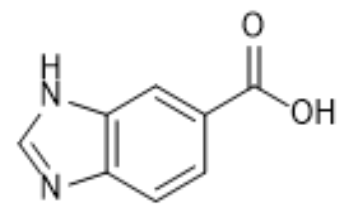	45.73
	C34	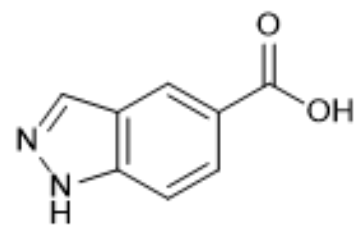	50.19
	C35	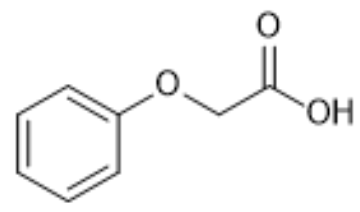	72.08
	C36	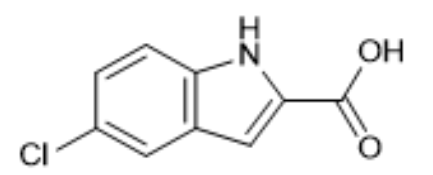	42.78
	C37	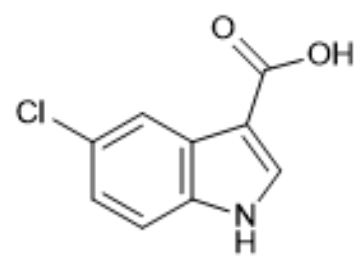	56.39
	C38	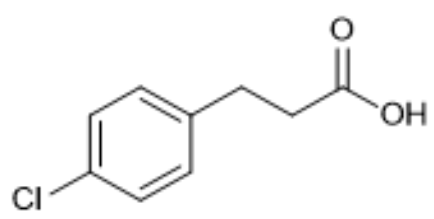	61.97
	C39	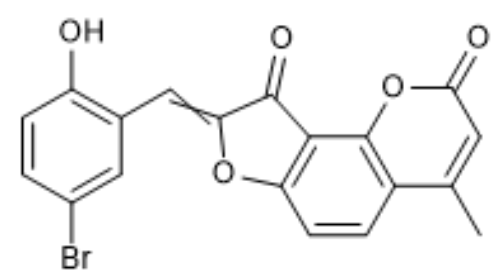	50.93
	C40	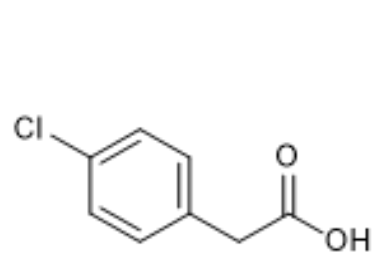	83.36
	C41	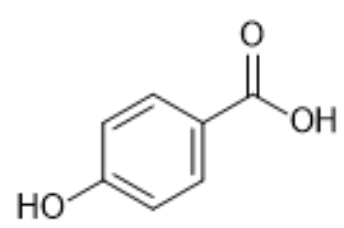	55.00

	C42	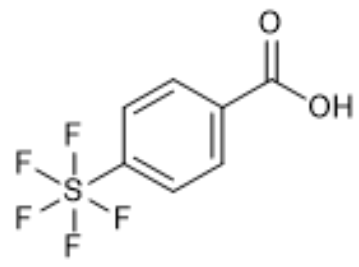	16.61
	C43	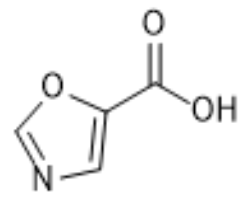	50.25
	C44	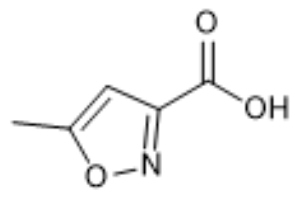	43.76
	C45	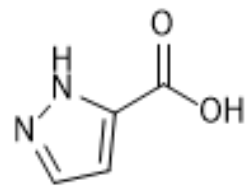	63.37
	C46	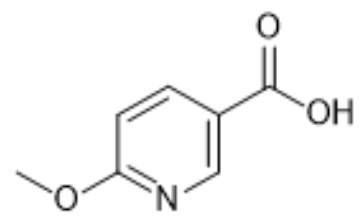	59.49
	C47	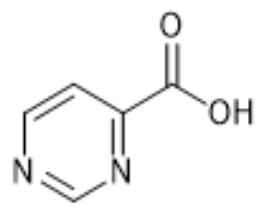	66.20
	C48	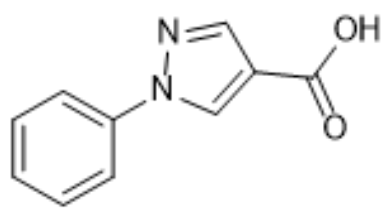	49.69
	C49	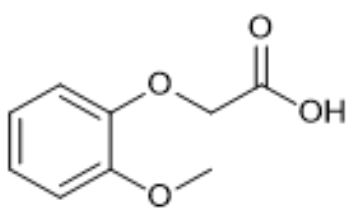	61.24
	C50	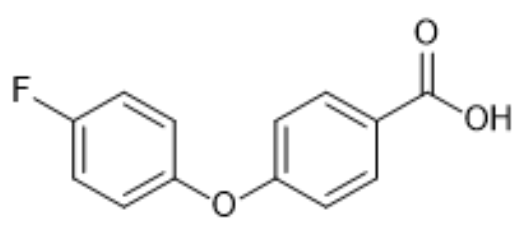	51.88

	C51	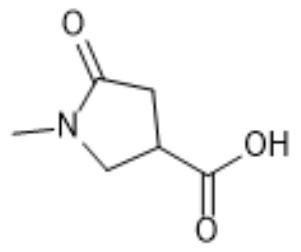	57.93
	C52	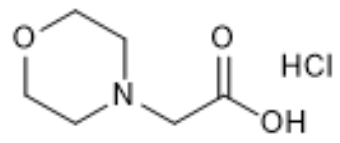	48.44
	C53	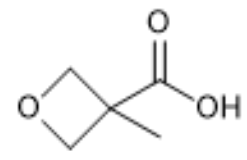	68.31
	C54	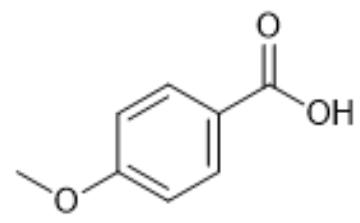	60.68
	C55	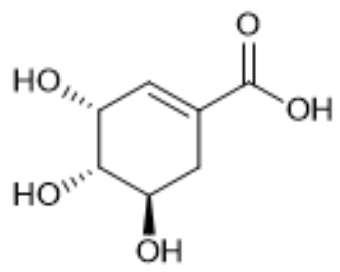	61.66
	C56	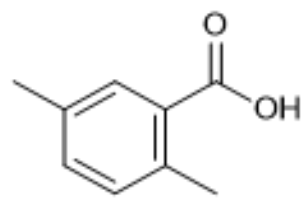	57.83
	C57	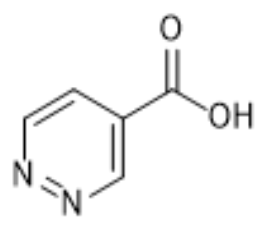	45.80
	C58	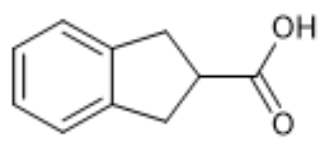	58.68
	C59	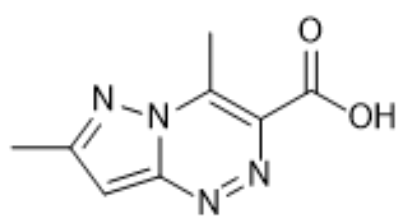	22.41

	C60	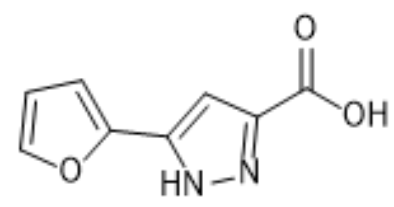	49.19
	C61	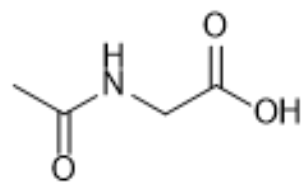	59.72
	C62	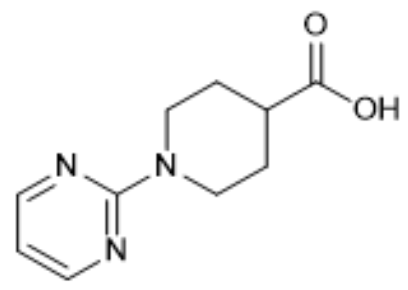	34.96
	C63	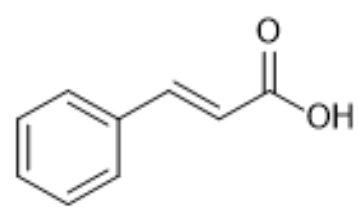	27.41
	C64	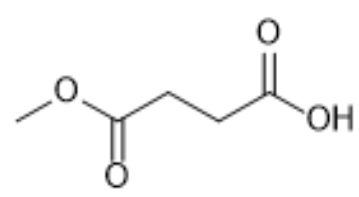	76.11
	C65	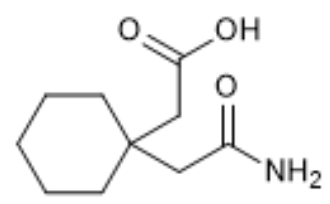	63.59
	C66	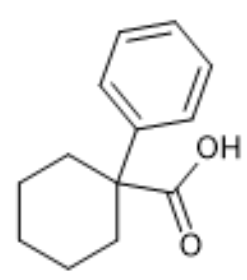	61.76
17	C67	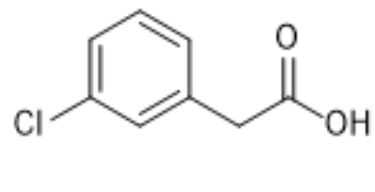	86.13
	C68	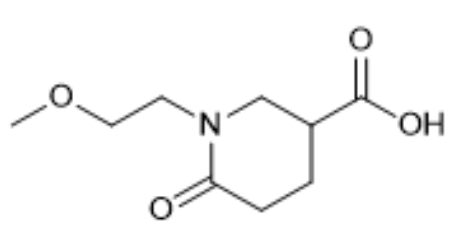	53.42

	C69	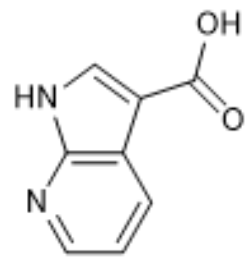	58.90
	C70	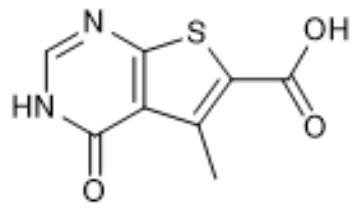	52.15
	C71	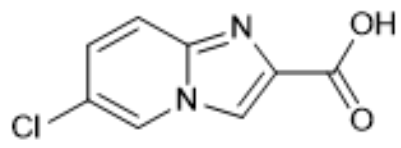	35.20
	C72	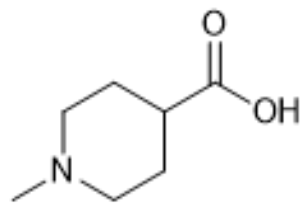	50.90
	C73	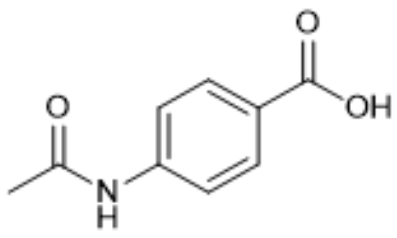	70.24
	C74	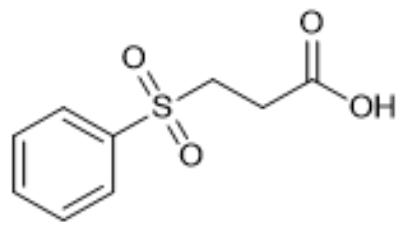	74.99
	C75	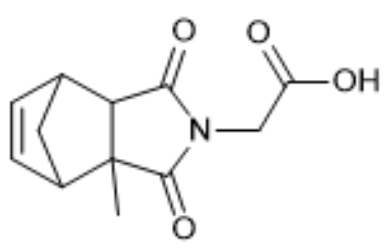	81.51
	C76	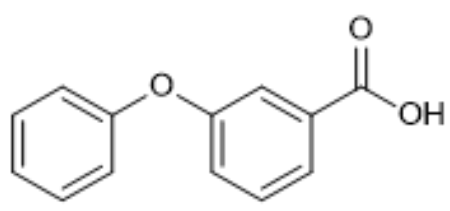	62.10
	C77	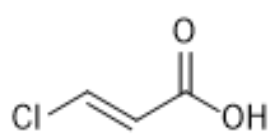	57.65

	C78	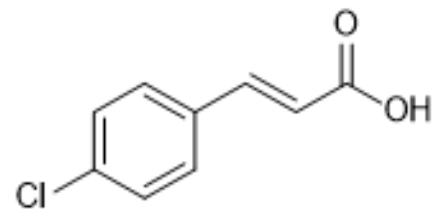	37.09
	C79	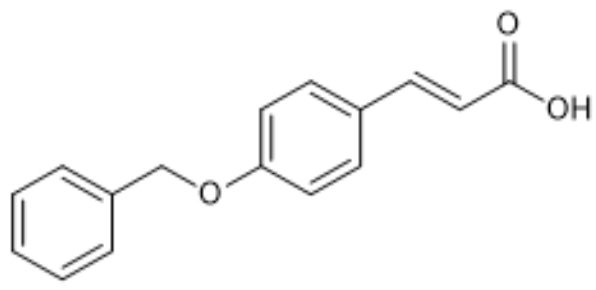	55.12
	C80	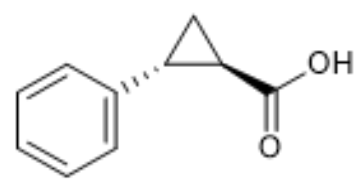	58.24
	C81	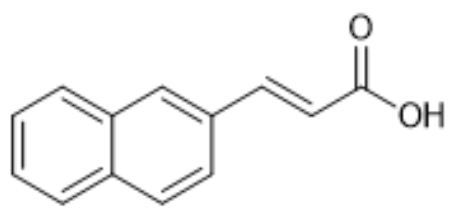	58.29
	C82	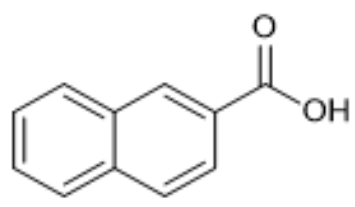	40.71
	C83	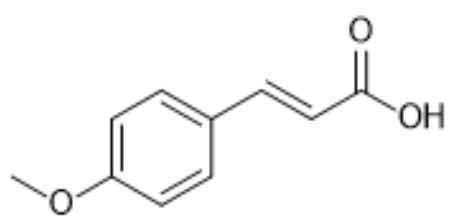	79.42
18	C84	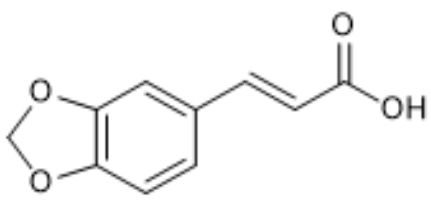	87.08
	C85	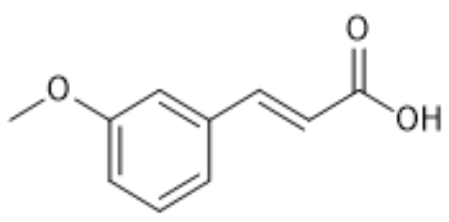	82.26
	C86	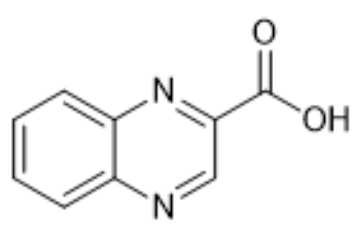	53.10

	C87	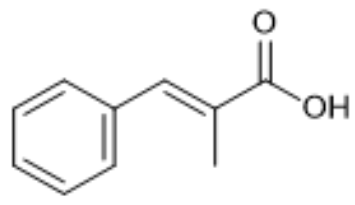	68.52
	C88	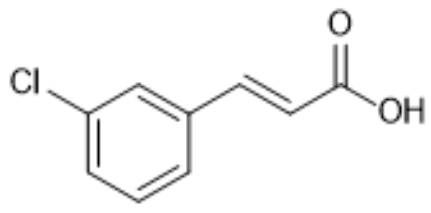	35.37
	C89	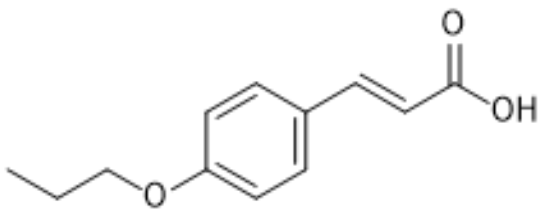	53.27
	C90	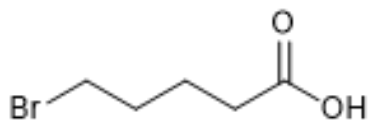	79.84
	C91	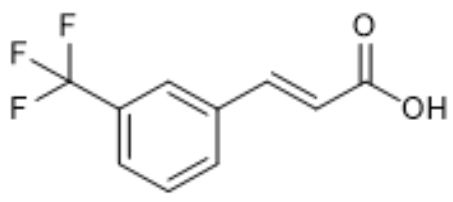	23.99
	C92	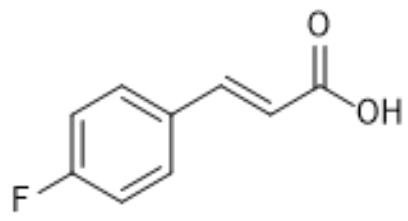	48.09
	C93	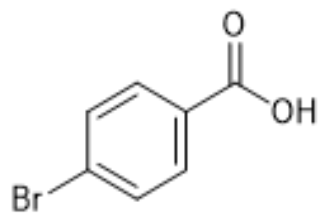	44.79
	C94	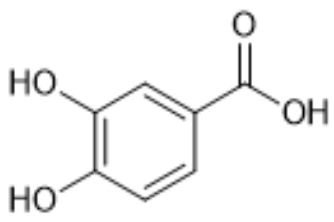	52.37
	C95	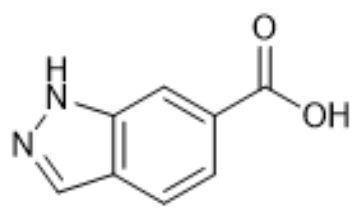	48.29

	C96	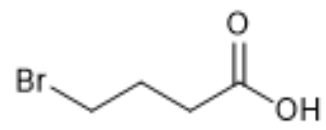	65.49
	C97	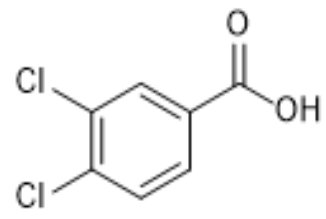	64.37
	C98	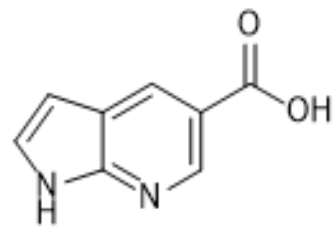	69.50
	C99	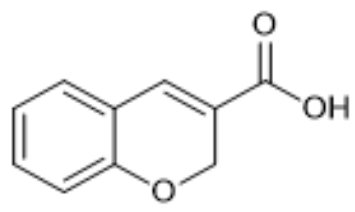	53.29
	C100	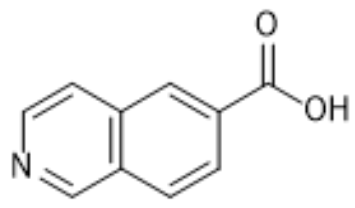	58.32
	C101	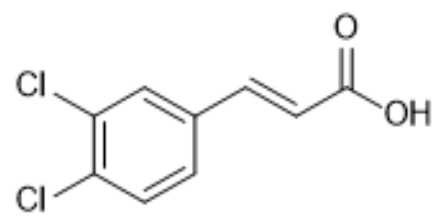	16.88
	C102	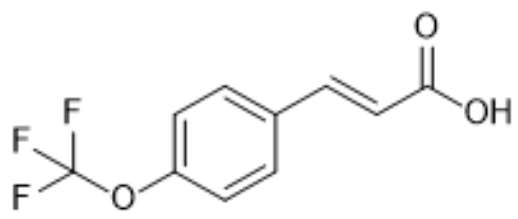	18.37
	C103	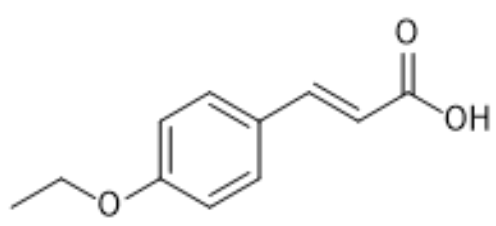	67.67
	C104	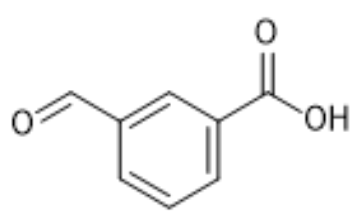	62.48

	C105	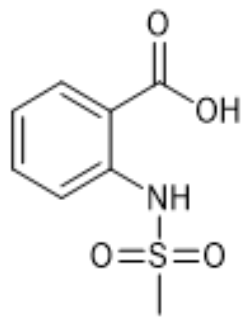	63.99
	C106	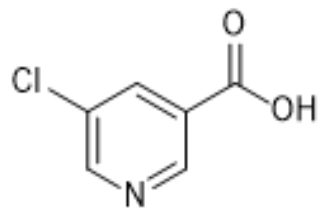	75.00
	C107	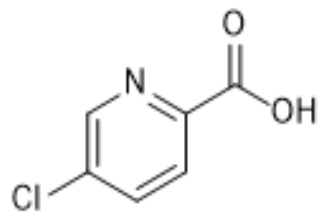	44.74
	C108	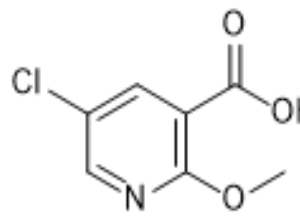	68.55
	C109	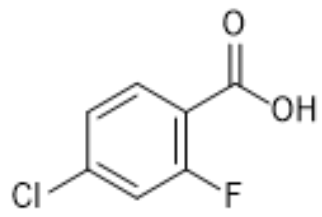	46.68
	C110	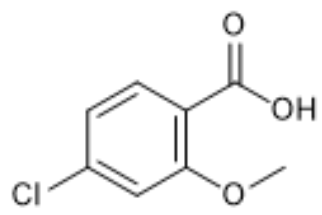	46.98
	C111	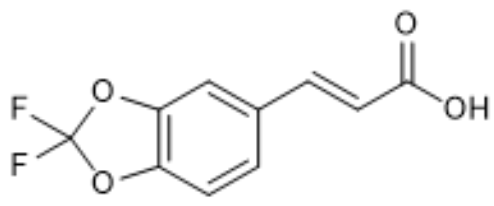	37.26
	C112	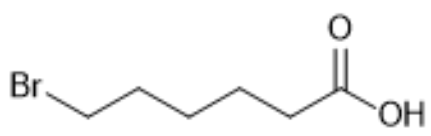	77.45
	C113	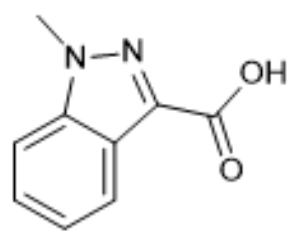	54.86

	C114	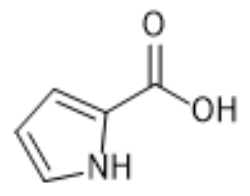	50.73
	C115	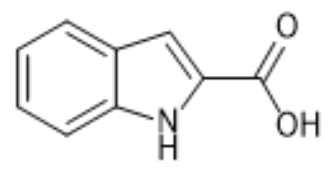	36.30
	C116	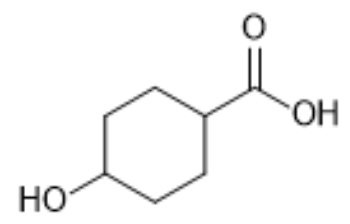	60.90
	C117	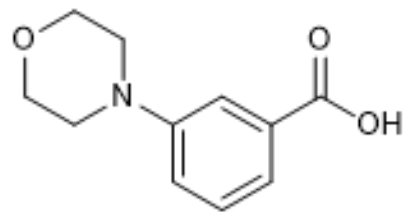	60.76
	C118	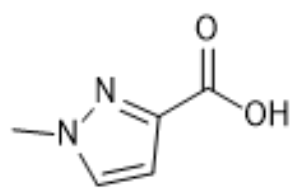	57.22
	C119	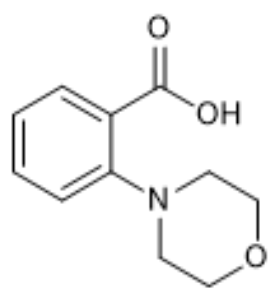	51.72
	C120	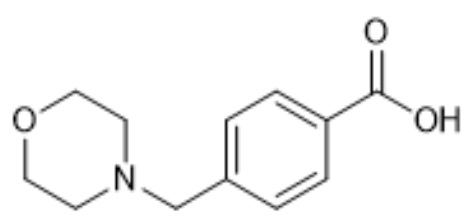	44.39
	C121	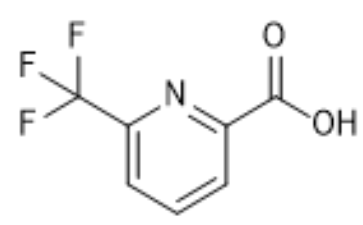	70.52
	C122	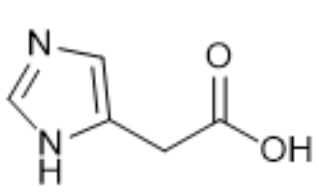	36.82

	C123	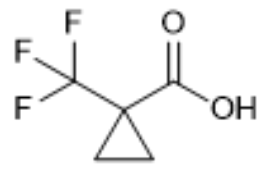	61.62
	C124	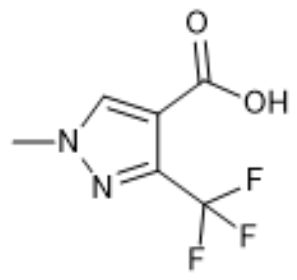	37.35
	C125	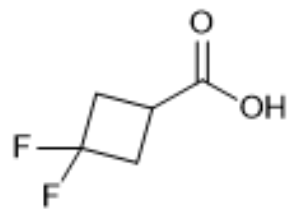	73.77
	C126	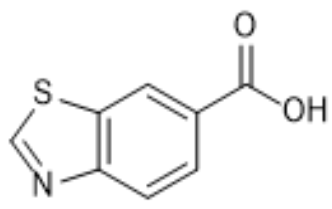	72.67
	C127	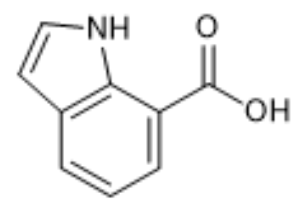	54.83
	C128	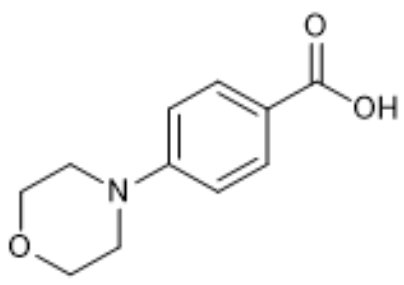	49.99
	C129	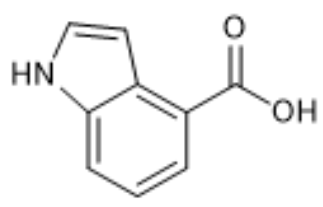	57.01
	C130	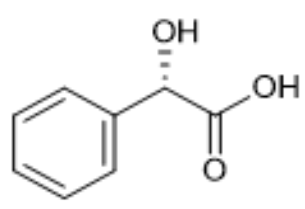	83.86
	C131	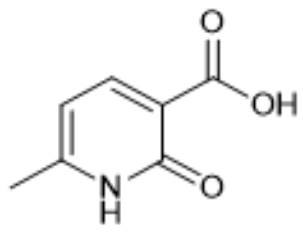	44.37

	C132	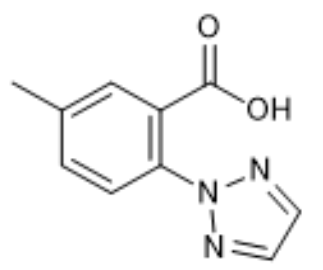	53.99
	C133	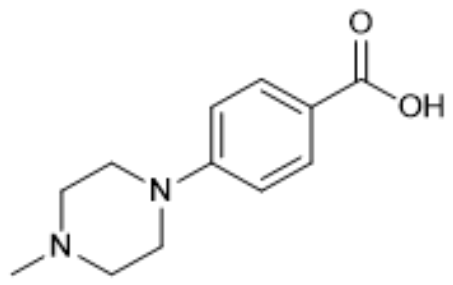	33.64
	C134	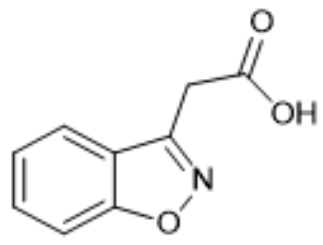	50.13
	C135	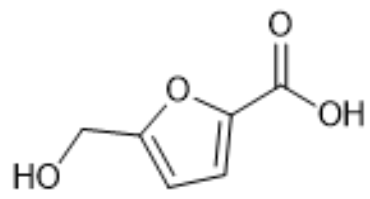	59.82
	C136	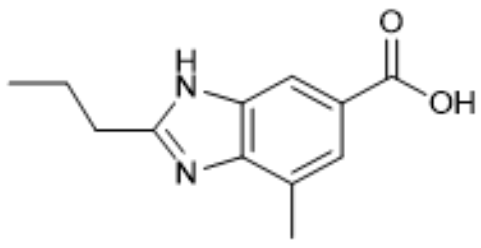	61.16
	C137	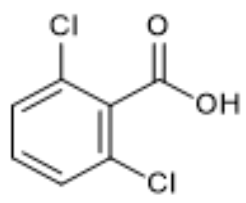	55.77
	C138	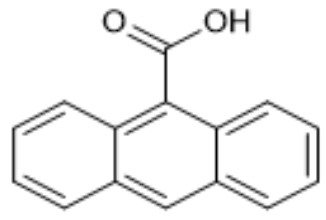	62.05
	C139	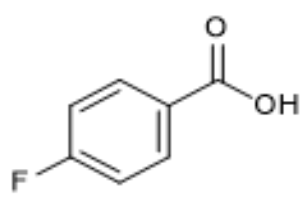	56.57
	C140	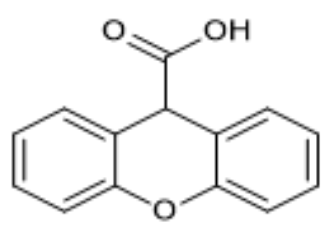	51.31

	C141	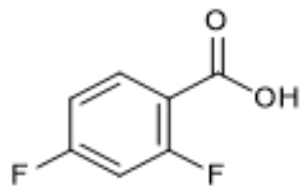	47.14
	C142	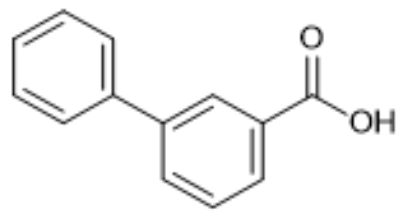	62.37
	C143	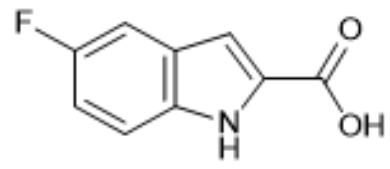	53.76
	C144	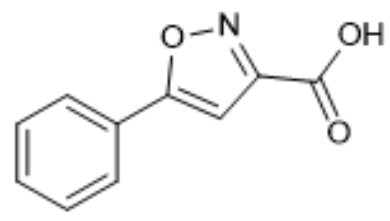	31.32
	C145	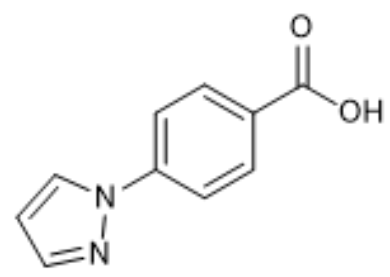	34.29

Supplementary Table 11. Data collection and refinement statistics for X-ray co-crystal structure of peptides 30, 33 and 34 bound to Keap1 Kelch domain. Statistics on X-ray diffraction data collection and refinement of Keap1 in complex with peptides **30** (PDB 9QDU), **33** (PDB 9QBS) and **34** (PDB 9QBT). Single crystals were used to collect all diffraction data. Highest-resolution shell statistics are shown within brackets.

	Keap1 Kelch domain in complex with peptide 30	Keap1 Kelch domain in complex with peptide 33	Keap1 Kelch domain in complex with peptide 34
**Data collection**			
Space group	P 43 21 2	P 41 21 2	H 3 2
Cell dimensions			
a, *b*, *c* (Å)	75.48, 75.48, 231.736	100.85, 100.85, 126.76	119.78, 119,78, 167.24
α, β, γ (°)	90.0, 90.0, 90.0	90.0, 90.0, 90.0	90.0, 90.0, 120.0
Resolution (Å)*	19.69 – 2.35 (2.39 – 2.35)	9.54 – 1.95 (1.98 - 1.95)	19.47 – 2.33 (2.37 – 2.33)
R_{sym} or R_{merge}*	0.129 (1.098)	0.140 (0.864)	0.077 (0.434)
$I / \sigma I$*	21.4 (3.2)	11.7 (2.8)	15.6 (5.9)
Completeness (%)*	100.0 (100.0)	99.5 (91.0)	99.9 (97.8)
Redundancy*	27.8 (22.0)	8.5 (6.3)	5.8 (5.5)
**Refinement**			
Resolution (Å)	19.69 – 2.35	19.54 – 1.95	19.47 – 2.33
No. reflections	802603 (30782)	407072 (13605)	115196 (5251)
R_{work} / R_{free}	0.1730 (0.2290) / 0.1950 (0.2852)	0.1680 (0.2272) / 0.2011 (0.2597)	0.1716 (0.2191) / 0.2083 (0.2829)
No. atoms	2380	4923	2314
Protein	2219	4415	2196
Ligand/ion	67	128	58
Water	94	380	61
B-factors	41.72	23.96	36.97
Protein	41.44	23.47	37
Ligand/ion	46.86	29.02	41.32
Water	44.58	28.19	31.71
R.m.s. deviations			
Bond lengths (Å)	0.009	0.007	0.009
Bond angles (°)	0.96	0.87	0.97

*Values in parentheses are for highest-resolution shell.

Supplementary Table 12. Interactions in X-ray structure between peptide 30 and Keap1 Kelch domain. Atoms of the cyclic peptide **30** forming interactions with atoms and residues of Keap1. Interactions have distances shorter than 4.0 Å and were defined using the software ChimeraX and LIGPOLT+.

Keap1 atom / residue	Peptide 30	Distance (Å)	Interaction
NH1 / Arg 415	N42	3.00	HB
NH2 / Arg 415	N42	3.16	HB
NH1 / Arg 483	O61	2.84	HB
NH2 / Arg 483	O61	2.84	HB
NE2 / Gln 530	O10	2.85	HB
OC / Ser 602	O20	2.67	HB

Supplementary Methods

Quality of reagents

Reagents were sourced from commercial suppliers and utilized directly without any purification steps. The solvents were not subjected to drying or preparation to obtain anhydrous conditions. The following abbreviations are employed throughout the text. BDT (1,4-butanedithiol), DMSO (dimethyl sulfoxide), DIPEA (*N,N*-diisopropylethylamine), DCM (dichloromethane), DMF (*N,N′*-dimethylformamide), DABCO (1,4-diazabicyclo[2.2.2]octane), HATU (*O*-(7-azabenzotriazol-1-yl)-*N,N,N′,N′*-tetramethyluronium hexafluorophosphate), HBTU (*N,N,N′,N′*-tetramethyl-*O*-(1H-benzotriazol-1-yl)-uronium-hexafluorophosphate), HEPES (4-(2-hydroxyethyl)-1-piperazine ethanesulfonic acid), NMM (*N*-methylmorpholine), NMP (1-methyl-2-pyrrolidinone), PBS (phosphate-buffered saline). IPTG (isopropyl-β-D-thiogalactopyranoside), TFA (trifluoroacetic acid), TIPS (triisopropylsilane), TEA (triethylamine), TCEP (tris(2-carboxyethyl)phosphine).

LC-MS analysis

Peptide samples were characterized by LC–MS using a Shimadzu 2020 single-quadrupole LC–MS system equipped with a reversed-phase C18 column (Kinetex® C18, 50×2.1 mm, 100 Å, 2.6 µm, Phenomenex). Separation was performed with a linear gradient of 0–60% (v/v) solvent B or 10–100% (v/v) solvent B over 5 min at a flow rate of 1 mL min^{-1}. Solvent A consisted of 99.95% (v/v) H_2O and 0.05% (v/v) formic acid, while solvent B contained 99.95% (v/v) acetonitrile and 0.05% (v/v) formic acid. Mass detection was carried out using electrospray ionization in positive mode (ESI^+). The acquired data were processed with LabSolutions software (version 5, Shimadzu).

Preparative HPLC

Peptides were purified by semi-preparative RP-HPLC on a Thermo Scientific Dionex UltiMate 3000 UHPLC instrument. A Waters XSelect® CSH C18 OBD column (10×150 mm, 130 Å pore size, 5 µm particle size) was used with a flow rate of 4 mL min^{-1}. Chromatographic separation was performed using a 35 min linear gradient of either 10–70% or 30–80% solvent B. Solvent A consisted of water (99.9%, v/v) containing 0.1% (v/v) TFA, while solvent B contained acetonitrile (99.9%, v/v) with 0.1% (v/v) TFA.

Synthetic DNA

Synthetic DNA of Keap1 Kelch domain cloned into pET29b(+) (underlined are the restriction sites for cloning):

CATATGGGTAGCAGCCATCACCACCACCACCACAGCAGCGGCGAGAACCTGTACTTCC
AGAGCGCGCCGAAAGTGGGTCGTCTGATCTACACCGCGGGTGGCTATTTTCGTCAAAG
CCTGAGCTACCTGGAAGCGTATAACCCGAGCGATGGTACCTGGCTGCGTCTGGCGGA
CCTGCAGGTGCCGCGTAGCGGTCTGGCGGGTTGCGTGGTTGGTGGCCTGCTGTACGC
GGTTGGTGGCCGTAACAACAGCCCGGATGGTAACACCGACAGCAGCGCGCTGGATTG
CTATAACCCGATGACCAACCAATGGAGCCCGTGCGCGCCGATGAGCGTTCCGCGTAAC
CGTATCGGTGTGGGCGTTATCGATGGTCACATTTACGCGGTGGGTGGCAGCCACGGCT
GCATTCACCACAACAGCGTTGAGCGTTATGAGCCGGAACGTGACGAATGGCATCTGGT
GGCGCCGATGCTGACCCGTCGTATTGGCGTGGGCGTTGCGGTGCTGAACCGTCTGCT
GTATGCGGTTGGTGGCTTCGATGGTACCAACCGTCTGAACAGCGCGGAGTGCTACTAT
CCGGAGCGTAACGAATGGCGTATGATCACCGCGATGAACACCATTCGTAGCGGTGCG
GGCGTTTGCGTGCTGCACAACTGCATTTACGCGGCGGGTGGCTATGATGGTCAGGACC
AACTGAACAGCGTGGAACGTTACGACGTTGAGACCGAAACCTGGACCTTCGTGGCGCC
GATGAAACACCGTCGTAGCGCGCTGGGTATCACCGTGCACCAGGGTCGTATTTACGTT
CTGGGTGGCTATGACGGCCACACCTTTCTGGACAGCGTGGAGTGCTATGACCCGGATA
CCGACACCTGGAGCGAAGTTACCCGTATGACCAGCGGCCGTAGCGGTGTTGGCGTGG
CGGTTACCTAACTCGAG

Synthetic DNA of Keap1 Kelch domain with mutations E540A and E542A cloned into pET29b(+) (underlined are the restriction sites for cloning):

CATATGGGTAGCAGCCATCACCACCACCACCACAGCAGCGGCGAGAACCTGTACTTCC
AGAGCGCGCCGAAAGTGGGTCGTCTGATCTACACCGCGGGTGGCTATTTTCGTCAAAG
CCTGAGCTACCTGGAAGCGTATAACCCGAGCGATGGTACCTGGCTGCGTCTGGCGGA
CCTGCAGGTGCCGCGTAGCGGTCTGGCGGGTTGCGTGGTTGGTGGCCTGCTGTACGC
GGTTGGTGGCCGTAACAACAGCCCGGATGGTAACACCGACAGCAGCGCGCTGGATTG
CTATAACCCGATGACCAACCAATGGAGCCCGTGCGCGCCGATGAGCGTTCCGCGTAAC
CGTATCGGTGTGGGCGTTATCGATGGTCACATTTACGCGGTGGGTGGCAGCCACGGCT
GCATTCACCACAACAGCGTTGAGCGTTATGAGCCGGAACGTGACGAATGGCATCTGGT
GGCGCCGATGCTGACCCGTCGTATTGGCGTGGGCGTTGCGGTGCTGAACCGTCTGCT
GTATGCGGTTGGTGGCTTCGATGGTACCAACCGTCTGAACAGCGCGGAGTGCTACTAT
CCGGAGCGTAACGAATGGCGTATGATCACCGCGATGAACACCATTCGTAGCGGTGCG
GGCGTTTGCGTGCTGCACAACTGCATTTACGCGGCGGGTGGCTATGATGGTCAGGACC

AACTGAACAGCGTGGAACGTTACGACGTTGCAACCGCAACCTGGACCTTCGTGGCGCC
GATGAAACACCGTCGTAGCGCGCTGGGTATCACCGTGCACCAGGGTCGTATTTACGTT
CTGGGTGGCTATGACGGCCACACCTTTCTGGACAGCGTGGAGTGCTATGACCCGGATA
CCGACACCTGGAGCGAAGTTACCCGTATGACCAGCGGCCGTAGCGGTGTTGGCGTGG
CGGTTACCTAACTCGAG

Synthetic DNA of Keap1 Kelch domain with the mutation C434A cloned into pET29b(+) (underlined are the restriction sites for cloning):

CATATGGGTTCTTCTCATCATCATCATCATCATTCTTCTGGTGAAAATTTATATTTTCAAT
CTGCTCCTAAAGTTGGTCGTTTAATTTATACTGCTGGTGGTTATTTTCGTCAATCTTTATC
TTATTTAGAAGCTTATAATCCTTCTGATGGTACTTGGTTACGTTTAGCTGATTTACAAGTT
CCTCGTTCTGGTTTAGCTGGTTGTGTTGTTGGTGGTTTATTATATGCTGTTGGTGGTCGT
AATAATTCTCCTGATGGTAATACTGATTCTTCTGCTTTAGATTGTTATAATCCTATGACTA
ATCAATGGTCTCCTTGTGCTCCTATGTCTGTTCCTCGTAATCGTATTGGTGTTGGTGTTA
TTGATGGTCATATTTATGCTGTTGGTGGTTCTCATGGTGCTATTCATCATAATTCTGTTG
AACGTTATGAACCTGAACGTGATGAATGGCATTTAGTTGCTCCTATGTTAACTCGTCGTA
TTGGTGTTGGTGTTGCTGTTTTAAATCGTTTATTATATGCTGTTGGTGGTTTTGATGGTA
CTAATCGTTTAAATTCTGCTGAATGTTATTATCCTGAACGTAATGAATGGCGTATGATTA
CTGCTATGAATACTATTCGTTCTGGTGCTGGTGTTTGTGTTTTACATAATTGTATTTATGC
TGCTGGTGGTTATGATGGTCAAGATCAATTAAATTCTGTTGAACGTTATGATGTTGAAAC
TGAAACTTGGACTTTTGTTGCTCCTATGAAACATCGTCGTTCTGCTTTAGGTATTACTGT
TCATCAAGGTCGTATTTATGTTTTAGGTGGTTATGATGGTCATACTTTTTTAGATTCTGTT
GAATGTTATGATCCTGATACTGATACTTGGTCTGAAGTTACTCGTATGACTTCTGGTCGT
TCTGGTGTTGGTGTTGCTGTTACTTAACTCGAG

Random cyclic peptide library synthesis

Solid-phase peptide synthesis (SPPS): Peptides were synthesized on a Multipep RSi parallel peptide synthesizer (Intavis) using standard Fmoc-based solid phase synthesis in 96-well plates. Polystyrene SH resin (200–400 mesh; Rapp Polymere) was functionalized with cysteamine in a disulfide exchange reaction as described previously[36]. Around 50% of the SH groups were typically loaded, which reduced the resin's capacity from 0.82 mmol/g SH groups to approximately 0.4 mmol/g aminothiol groups. The indicated synthesis scale refers to the loading of the aminothiol group on the resin and is an estimated value. For cyclic peptides with free amino groups, cysteamine-loaded PS resin (approximately 6 mg, or 2.5 µmol) was transferred to each well of a 96-well solid-phase synthesis plate (Orochem OF-1100) using a

solid dispensing tool described previously[26]. The resin in each well was rinsed with DMF (6×150 µl). Coupling was carried out using a mixture containing Fmoc-protected amino acid (53 µl, 500 mM, 10.6 equiv.), HATU (50 µl, 500 mM, 10 equiv.), NMM (12.5 µl, 4 M, 20 equiv.), and NMP (5 µl). The reagents were combined and pre-incubated for 1 min before being added to the resin, followed by a 1 h reaction without agitation. This coupling step was repeated twice, after which the resin was washed with DMF (6×225 µl). Fmoc removal was conducted in two cycles using 120 µl of 20% piperidine in DMF for 5 min each. Following deprotection, the resin was rinsed with DMF (8×225 µl). Upon completion of peptide synthesis, the resin was washed twice with DCM (2×200 µl).

Side-chain protecting group removal: After automated SPPS, the 96-well synthesis plates were closed by pushing on a soft 10 mm thick ethylene-vinyl acetate foam pad, sealing the well outlets. Resin of each well was incubated with around 600 µl of 38:1:1 TFA/TIPS/ddH$_2$O (v/v/v) and a polypropylene (PP) plastic seal (QuickSeal Micro, IST-125-080LS) to prevent TFA evaporation. After incubating for 2.5 h, the foam pads were removed and the synthesis plates were positioned onto 2 ml deep-well plates (Thermo Scientific, 278752) placed on a vacuum station, and the TFA mixture was allowed to drain under vacuum. Resin in each well was washed with 3×500 µl of DCM and subsequently air dried for 3-5 h.

Cyclative release of peptides: The outlets of the 96-well synthesis plates were closed as above and 200 µl of 150 mM DABCO in DMSO (12 equiv.) were added to each well. The plates were closed with an aluminium foil (Greiner, 676090) to minimize drainage during later transfer of the plates. After overnight incubation at room temperature, the 96-well synthesis plates were transferred to a 96-deep well plate (Biotix, Deep Well Plate, 96-Round Well, 1.2 ml) and centrifuged at 480 × g (around 1,500 rpm on a Thermo Scientific Multifuge 3L-R centrifuge with Sorval 75006445 rotor) for 2 min at room temperature to collect the released disulfide-cyclized peptides. The concentrations were estimated by measuring the UV absorbance of peptides containing tryptophan residues, as described further below. The average concentration for each synthesis run (384 peptides produced in four 96-well plates) was between 6.4 and 12.8 mM. Stocks were diluted to a peptide concentration of 6.4 mM by adding DMSO. All plates were covered with aluminum adhesive film and stored at -20°C for future use. Randomly picked cyclic peptides were analyzed by LC-MS.

Acylation of cyclic peptide cores to generate Library 1: Acylation of amino groups in cyclic peptides with carboxylic acids was carried out in 384-well plates (ThermoFischer NUNC™ 384 shallow plates, 264705) at a peptide scale of 380 pmol. The reagents were transferred using an Echo 650 acoustic liquid handler (Labcyte) and thus prepared in Echo qualified plates as follows. Peptides of four 96-well plates were transferred to one 384-well plate (Echo qualified 384-well PP, clear). Carboxylic acids in Eppendorf tubes were dissolved to 128 mM in DMSO

containing 128 mM DABCO. An equal volume of HBTU (128 mM in DMSO) was added to each carboxylic acid stock for activation, and transferred to Echo 384-well PP source plates. For combinatorial acylation, cyclic peptides (60 nl, 6.4 mM, 1 equiv.) were transferred followed by carboxylic acid/HBTU/DABCO (60 nl, 64 mM, 10 equiv.) to 384-well destination plates. To ensure droplet merging and proper coupling, all destination plates were manually sealed with plastic films (Greiner, 676001), centrifuged at 500 × g (around 2,000 rpm with SIGMA 4-16KS with a swing-out rotor 11660) and incubated 5 h at room temperature. To prevent reagent precipitation in the following quenching reaction, 105 nl of DMSO were dispensed per well using a bulk dispenser (Fritz Gyger, Certus Flex). The activated acids were then quenched by adding 4,8 µl of quench buffer (25 mM Tris, 20 mM NaCl, pH 7.4) to all wells, also using the bulk dispenser.

Peptide quantification by absorption

The concentrations of cyclic peptide stocks obtained after SPPS and cyclative release were estimated based on peptides that contained a Trp and for which the concentration could be determined by measuring absorbance at 280 nm. From the 96-well plates 1-4 (1st synthesis run), 5-8 (2nd synthesis run) and 9-12 (3rd synthesis run), all peptides containing a Trp were analyzed. From plates 13-16 (4th synthesis run), that contain many peptides with Trp, 35 peptides were randomly picked for analysis. For concentration determination, peptides were diluted 100-fold in water for absorbance measurements. The measurements were conducted using a Nanodrop 8000 spectrophotometer (ThermoFisher Scientific) at a wavelength of 280 nm using a 1 mm path length. An extinction coefficient of 5,500 $M^{-1}cm^{-1}$ was used for the absorbance of Trp at 280 nm.

Synthesis of disulfide-cyclized peptide and purification

For producing purified disulfide-cyclized peptide, SPPS was performed using an Intavis Multipep synthesizer and 5 ml syringe reactors. Polystyrene SH resin (200-400 mesh; Rapp) carrying cysteamine was prepared as described above. In the following, the indicated synthesis scale refers to the loading of aminothiol group on resin and is an estimated value. Cysteamine-loaded PS resin (around 60 mg, 25 µmol scale) was transferred to each syringe reactor (MultiSynTech, V050TF062). The resin was initially rinsed with DMF (2×2,000 µl). Coupling was carried out using Fmoc-protected amino acid (530 µl, 500 mM, 5.3 equiv.), HATU (500 µl, 500 mM, 4 equiv.), and NMM (125 µl, 4 M, 8 equiv.). The reagents were pre-mixed for 1 min prior to addition to the resin, followed by incubation for 45 min with shaking. Each coupling step was repeated twice. Afterward, the resin was washed with DMF (2×2,000

μl). Fmoc removal was achieved by treating the resin twice with piperidine in DMF (1:4, v/v; 900 μl each) for 5 min. The resin was then washed with DMF (6×2,000 μl). Upon completion of the peptide synthesis, the resin was rinsed with DCM (2×2,000 μl) and dried under vacuum while passing air through the resin.

Peptides were deprotected on resin by incubation with 4 ml solution of 38:1:1 TFA/TIPS/ddH_2O (v/v/v) for 2 h at room temperature. The TFA solution was discarded, and the resin was washed three times with 5 ml DCM and twice with 5 ml DMF. For cyclative release, 2 ml of 150 mM DIPEA in DMSO was pulled into the syringe and the closed syringe shaken overnight at room temperature. The DMSO solution was pushed into a 50 ml Falcon tube.

To couple the carboxylic acid to disulfide-cyclized peptides, a pre-prepared mixture of acid (100 mM, 2 equiv.), HBTU (100 mM, 2 equiv.), and DABCO (100 mM, 2 equiv.) in DMSO (500 μl) was added. The reaction mixture was then incubated for 3 h at room temperature. The reaction mixture was then diluted by adding 17.5 ml of water to reduce the DMSO concentration. The crude mixture was purified in 3-5 runs by semi-preparative HPLC, and the fractions containing the desired product was collected and lyophilized.

High-throughput synthesis of linear peptides for SAR analysis

Solid-phase peptide synthesis (SPPS): Peptides were synthesized on a Intavis Multipep synthesizer using standard Fmoc-based solid phase synthesis in 384 plates. Aminothiol-loaded PS resin (around 3 μmol scale based on disulfide-linked aminothiol group loading; 0.44 mmol/g) was transferred to each well of 384-well solid phase synthesis plates (Agilent, 201035-100) by the 384 solid dispenser as reported by Schüttel M., *et. al*. [26]. Coupling was performed with a solution of 30 μl Fmoc amino acid (500 mM, 5 equiv.), 28 μl HATU (500 mM, 4.7 equiv.), and 8 μl of NMM (4 M, 10.7 equiv.). All components were pre-mixed for 1 min, then added to the resin for 1 h reaction without shaking. Coupling was performed twice followed by resin washing with 6×70 μl of DMF. Fmoc deprotection was performed twice, each time using 35 μl of piperidine in DMF (1:4, v/v) for 2 min. After Fmoc deprotection, the resin was washed with 8×70 μl DMF. For manual on-resin Boc deprotection, 2×50 μl of TFA in DCM (1:4, v/v) was added to each well for 30 min. After the Boc deprotection, the resin was washed with 4×70 μl DCM, then washed with 4×70 μl DMF. The peptide elongation was carried out using the standard Fmoc-based solid-phase synthesis procedure as described above. At the end of the peptide synthesis, the resin was washed with 2×80 μl of DCM and dried under vacuum.

Side-chain protecting group removal: After the SPPS, the resin in each well was incubated with 2×50 µl 38:1:1 TFA/TIPS/ddH_2O (v/v/v) solution for 1 h. During incubation, the plates were covered with a box in a fume hood to prevent rapid evaporation and odor. After the incubation, the 384-well synthesis plate was placed onto a 2 ml deep-well plate. The TFA solution was drained, followed by washing of the resin with DCM (3×120 µl). The resin was then air-dried for 3–5 h.

Reductive release of peptides: The outlets of the 384-well synthesis plate were blocked as described above. To resin of each well, 60 µl of release solution (200 mM TEA and 200 mM BDT, 4 equiv. in DMF) was added. The plates were covered with a box and incubated overnight at room temperature in a fume hood. The synthesis plates were then sealed with a polypropylene adhesive film, stacked on 384-well deep well plates, and centrifuged at 500 × g (around 2,000 rpm in a ThermoFisher 3L-R centrifuge with a swing-out rotor 75006445) for 2 min at room temperature. The release process was repeated with a shorter incubation time of 3 h. A total of 120 µl per well of peptide was obtained in 384-well deep well plates. All plates were covered with aluminum adhesive film and stored at -20°C for future use. The concentrations of the linear peptides were determined by Ellman's reagent as described below and adjusted to an average of approximately 6 mM.

Synthesis of peptides with warheads

Linear peptides for coupling of warheads were produced by automated SPPS in 5 ml syringe reactors. A quantity of 47 mg of HMBA-AM resin (0.64 mmol g^{-1}; 30 µmol scale) was swollen in 5 ml of DCM and washed with DMF. The first amino acid was manually coupled to the solid support as follows. A volume of 0.75 ml of 200 mM Fmoc amino acid (5 equiv.) in DMF was mixed with 20 mg HOBt (5 equiv.), added to the resin and shaken for 2 min. To this solution 23 µl of DIC (5 equiv.) and 0.4 mg DMAP in 20 µl DMF (0.1 equiv.) were sequentially added and the reaction incubated for 4 h at room temperature with gentle shaking. The unreacted hydroxyl groups on the resin were capped by adding a mixture prepared of 6 µl acetic anhydride (2 equiv.) and 5 µl pyridine (2 equiv.). The reaction was further incubated for an additional 30 min to ensure complete capping. The resin was washed with 3×5 ml DMF to remove excess reagents and by-products. Further amino acids were coupled by automated SPPS using conditions described for 5 ml syringe reactors above.

After completion of SPPS, the protecting groups were removed by adding 4 ml TFA/ddH_2O/phenol/TIPS mixture in a 94:2.5:2.5:1 ratio (v/v/v/v), and the resin was incubated for 2.5 h. The resin was washed with 3×5 ml DCM and 3×5 ml DMF. The peptide was released from the solid support by adding 5 ml of 150 mM diamino butane (25 equiv.) or 5 ml of 150

mM diamino ethane (25 equiv.) and incubation overnight at 30°C. The cleavage solution was collected, evaporated by vacuum centrifugal evaporation and re-dissolved in DMSO to purify the peptide by reversed-phase HPLC.

Electrophilic warheads were coupled to the peptides using the following procedures. For acryloyl chloride conjugation, a volume of 150 µl of DMF was placed into an Eppendorf tube and the following reagents added sequentially: 5 µl of 500 mM peptide (2.5 µmol, 1 equiv.), 2.2 µl of DIPEA (5 equiv.), and 1.0 µl of acryloyl chloride (4.6 equiv.). The reagents were mixed and incubated for 20 min. Conversion was assessed by LC-MS and the product purified by revered-phase HPLC. For chloro- and bromoacetic acid conjugation, a volume of 130 µl of DMF was placed into an Eppendorf tube and the following reagents added sequentially: 5 µl of 500 mM peptide (2.5 µmol, 1 equiv.), 2.2 µl of DIPEA (5 equiv.), 1.0 µl of DIC (2.5 equiv.), and 15 µl of 500 mM bromoacetic acid (3 equiv.). The reagents were mixed and incubated for 1 h. Conversion was assessed by LC-MS and the product purified by revered-phase HPLC. For acetylation of the terminal free amine, a volume of 50 µl of DMF was placed into an Eppendorf tube and the following reagents added sequentially: 2 µl of 500 mM peptide (1 µmol, 1 equiv.), 1 µl of acetic anhydride (10 equiv.), and 1 µl of lutidine (8.5 equiv.). The reagents were mixed and incubated for 2 h. Conversion was assessed by LC-MS and the product purified by revered-phase HPLC.

Chemical synthesis of library building blocks

Reactions were monitored by thin-layer chromatography (TLC) using silica gel coated plates (analytical SiO_2-60, F-254) and by LC-MS analysis. TLC plates were visualized under UV light. Rotary evaporation of solvents was carried out under reduced pressure at a temperature below 40°C. Nuclear magnetic resonance (NMR) spectra were recorded on a Bruker Avance III (^{1}H NMR and ^{13}C NMR recorded at 400 and 101 MHz, respectively) equipped with a cryogenically cooled probe. All spectra were recorded at 298 K. Chemical shifts are reported in ppm relative to deuterated solvent as internal standard (δH DMSO-d_6 2.50 ppm; δC DMSO 39.52 ppm). High-resolution mass spectrometry (HRMS) measurements were recorded on a Xevo G2-XS QTof time-of-flight (TOF) mass spectrometer.

4-(tritylthio)-butanoic acid (M2) (Supplementary Figure 24): A solution of trityl chloride (2.55 g, 9.15 mmol, 1.1 equiv.) was dissolved in CH_2Cl_2 (5 ml) and added dropwise to a solution of 4-mercaptobutyric acid (0.83 ml, 8.30 mmol, 1.0 equiv.) in CH_2Cl_2 (10 ml). The solution was stirred overnight at room temperature, quenched with water (50 ml) and extracted with CH_2Cl_2 (3×50 ml). The combined organic layers were washed with brine (2×50 ml), dried over anhydrous Na_2SO_4 and concentrated under reduced pressure. The crude product was purified

by the silica column chromatography to afford compound M2 (1.30 g, 3.59 mmol, 43%) as a colorless solid. TLC (50% EtOAc and 0.5% AcOH in hexane): R_f = 0.7 (UV). ^{1}H NMR (400 MHz, DMSO-d_6) δ 12.04 (s, 1H), 7.36–7.28 (m, 12H), 7.27–7.19 (m, 3H), 2.20–2.07 (m, 4H), 1.51 (p, *J* = 7.3 Hz, 2H). ^{13}C NMR (101 MHz, DMSO) δ 173.7, 144.5, 129.1, 128.0, 126.7, 66.0, 32.7, 30.7, 23.6. HRMS *m/z* calcd. for $C_{23}H_{21}O_2S^-$ $[M-H]^-$, 361.1268; found 361.1280.

2-(3-((tritylthio)methyl)phenyl)-acetic acid (M3) (Supplementary Figure 25): To a solution of 3-(bromomethyl)phenylacetic acid (1.15 g, 5.02 mmol, 1.0 equiv.) in DMF (6 ml) was added triphenylmethanethiol (1.46 g, 5.28 mmol, 1.05 equiv.) followed by *i*-Pr_2NEt (1.74 ml, 10.0 mmol, 2.0 equiv.). After overnight stirring at room temperature, the reaction mixture was diluted with EtOAc (200 ml) and washed with water (2×100 ml) and brine (2×100 ml). The organic layer was dried over anhydrous Na_2SO_4 and concentrated under reduced pressure. The crude product was purified by column chromatography to afford compound M3 (928 mg, 2.19 mmol, 44%) as a colorless solid. TLC (15% EtOAc and 0.5% AcOH in hexane): R_f = 0.2 (UV). ^{1}H NMR (400 MHz, DMSO-d_6) δ 12.30 (br s), 7.37 (d, *J* = 4.2 Hz, 12H), 7.30–7.14 (m, 4H), 7.10 (dt, *J* = 7.8, 1.5 Hz, 1H), 7.02–6.95 (m, 2H), 3.51 (s, 2H), 3.25 (s, 2H). ^{13}C NMR (101 MHz, DMSO) δ 172.6, 144.3, 136.5, 135.3, 129.9, 129.1, 128.4, 128.2, 128.1, 127.3, 126.8, 67.0, 40.5, 36.1. HRMS *m/z* calcd. for $C_{28}H_{23}O_2S^-$ $[M-H]^-$, 423.1424 found 423.1419.

High-throughput synthesis of linker-cyclized peptide library

For the synthesis of linear peptides required for Library 3-5, cysteamine analogues PS solid phase loaded with N1–N3 was prepared as previously reported[29]. The scale for each peptide was around 3 μmol scale, using PS resin with a disulfide-linked thiolamine group loading capacity of 0.81 mmol g^{-1} (N1), 0.52 mmol g^{-1} (N2), and 0.32 mmol g^{-1} (N3). Solid-phase peptide synthesis was performed as described for the Library 2 synthesis above with the exception of the coupling of M1, M2 and M3 that were performed with a solution prepared by mixing 30 μl M1-M3 building block (250 mM, 2.5 equiv.), 28 μl HATU (250 mM, 2.3 equiv.), and 8 μl of DIPEA (2 M, 5.3 equiv.). Removal of side-chain protecting groups and reductive peptide release were performed as described for Library 2. The concentrations of all peptides were determined by Ellman's reagent as also described for Library 2. The dithiol peptides were cyclize using bis-electrophilic linker reagents using a procedure adapted from Nielsen A., et al.[29] For each dithiol peptide, a quantity of 4 nmol was transferred to 384-well LDV plates (one plate per linker) using acoustic droplet ejection. The volumes transferred were calculated based on the concentration of the DMSO stocks that was different for each peptide. As dithiol peptides oxidize in DMSO over time, the peptides were fully reduced by adding a solution of BDT and NEt_3 in DMF (both 100 mM; 2 μl, 0.2 μmol, 50 equiv.) to each well, followed by

incubation for 60 min at room temperature. A solution of TFA in ddH_2O (2% (v/v); 1.5 µl, 2 equiv. relative to NEt_3) was added and the peptides were dried by vacuum centrifugal evaporation (30°C, 1,500 rpm, 0.1 mbar) to afford the fully reduced dithiol peptides as dried pellets. The linker reagents (L1−L3) were dissolved in the degassed cyclization buffer (60 mM NH_4HCO_3, pH 8 and MeCN, 1:1, v/v) to a final concentration of 4 mM. The prepared linker solutions were added to the wells of 384-well LDV plates (4 µl, 16 nmol, 4 equiv. relative to dithiol peptide) using the bulk dispenser (Certus). The plates were sealed with adhesive PP lids and agitated for 2 h at room temperature. For excess linker reagent quenching, β-mercaptoethanol (β-ME) stock solution was prepared by dissolving β-ME in the cyclization buffer to a final concentration of 32 mM. Of this β-ME solution, 2 µl were added to each well (4 equiv. relative to linker) by bulk dispensing and incubated for 1 h at room temperature. Solvent was removed by vacuum centrifugal evaporation (30°C, 1,500 rpm, 0.1 mbar) to afford the cyclic peptides as pellets. These pellets were dissolved in 4 µl DMSO to obtain 1 mM cyclic peptide stocks.

Synthesis and purification of linker-cyclized peptides

Linear dithiol peptides were synthesized on an Intavis Multipep RSi automated peptide synthesizer at a 25 µmol scale using 5 ml syringes as described for the production of purified disulfide-cyclized peptides above. Bis-electrophilic linkers were dissolved in the degassed cyclization buffer to a final concentration of 2 mM. The prepared linker solutions (12.5 ml, 2 equiv. relative to dithiol peptide) were added to a falcon tube containing the desired dithiol peptide pellet, and the solution was agitated for approximately 2 h at room temperature monitored by LC-MS analysis. Excess linker reagent was quenched by addition of β-ME (14 µl, 200 µmol, 4 equiv. relative to linker) and the solution was agitated for at least 30 min. The reaction mixtures were purified by preparative HPLC as described above. Fractions containing the desired cyclic peptide were lyophilized.

For the synthesis of cyclic peptide with warhead, a bis-electrophilic linker with a free amino group was prepared as follows: A solution of 2-(Boc-amino)ethanethiol (17.5 µl, 0.1mmol, 1 equiv.) and DIPEA (17.5 µl, 0.1 mmol, 1 equiv.) in 10 ml of DMF was added dropwise to a solution of 1,3,5-tris(bromomethyl)benzene (70 mg, 0.19 mmol, 1.9 equiv.) in 10 ml DMF. The reaction mixture was stirred at room temperature for 2 h, and completion was monitored by LC-MS. DMF was removed under reduced pressure and the crude product was redissolved in a 1:1 TFA/DCM (v/v) solution for 2 h to deprotect the Boc group. The solvents were evaporated under a stream of nitrogen and the residue was redissolved in DMSO and purified via HPLC. The product was used for dithiol peptide cyclization following the same procedure

as described above. The final acryloyl chloride coupling to the free amino group was carried out using the procedure described for the covalent inhibitors based on linear peptides.

Uncropped Scans of Gels

Gel of Supplementary Figure 5b

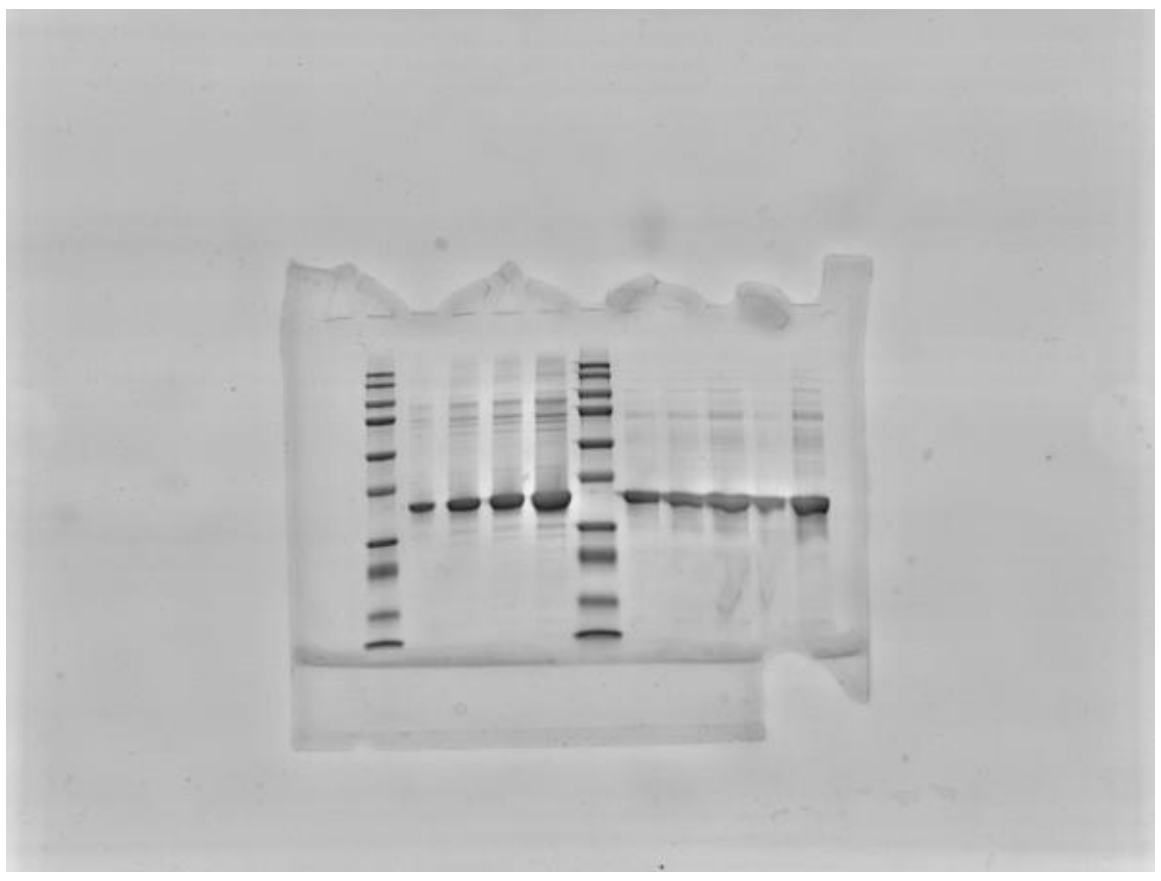

Gel of Supplementary Figure 9b

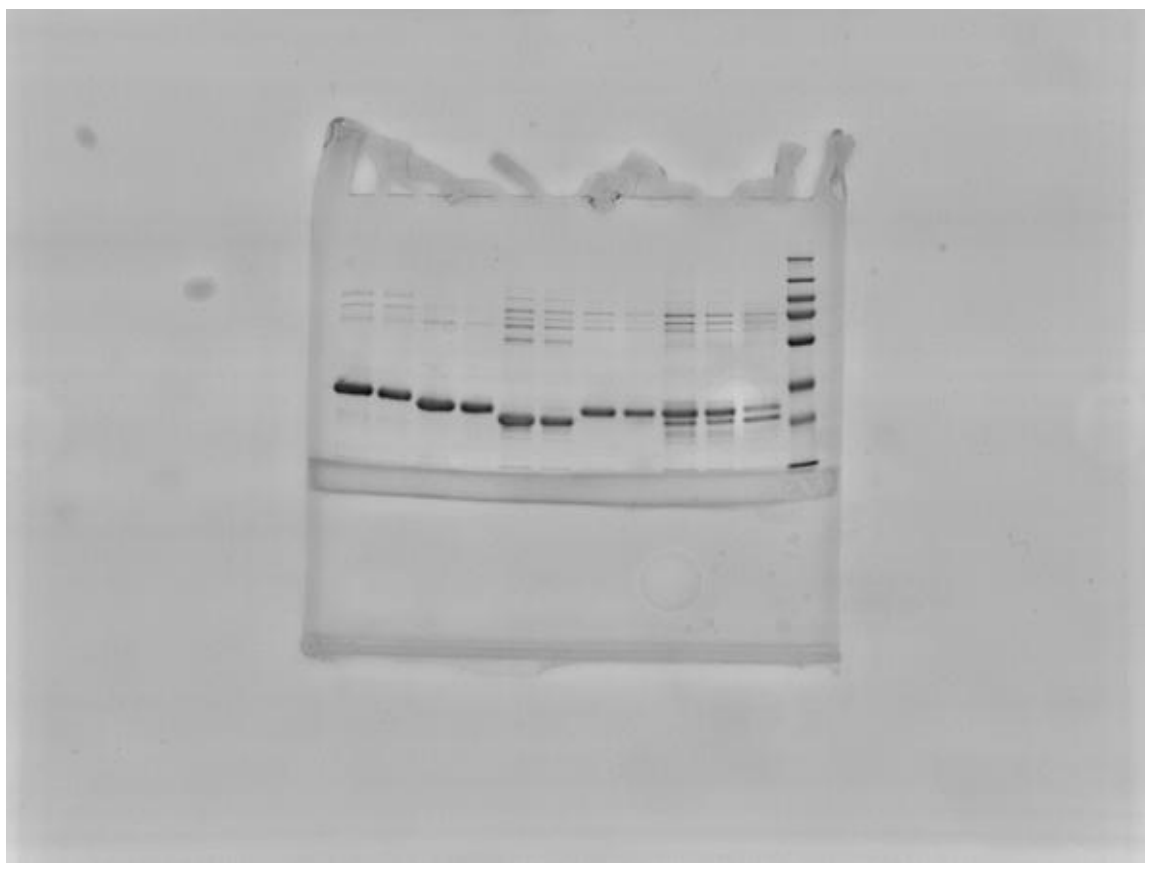

Gel of Supplementary Figure 16b

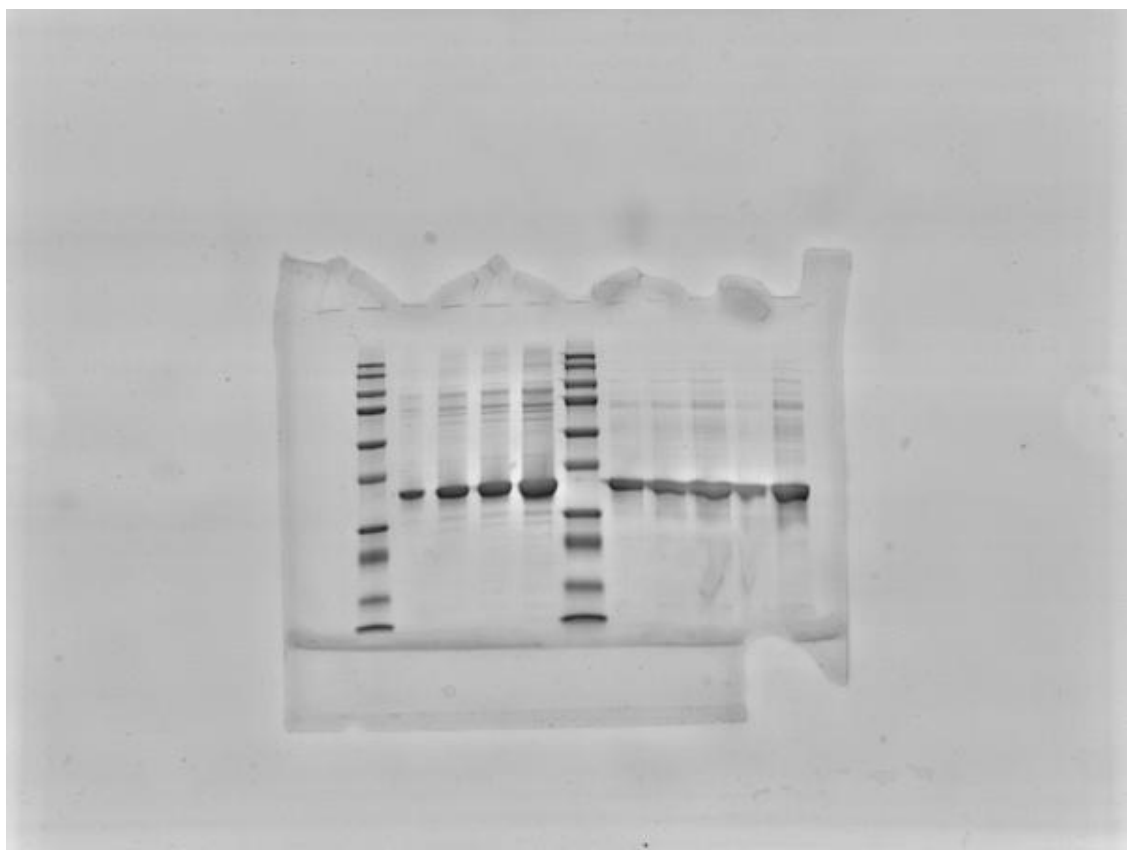

Supplement: Supplementary file 1 [file 41589_2026_2237_MOESM1_ESM.pdf]
