# Supplementary material for: Generation of membrane-permeable cyclic peptides inhibiting protein–protein interaction
Source: Nat Chem Biol. 2026 Jun 1;22(8):1351–61. doi: 10.1038/s41589-026-02237-7 (PMC13661285; doi:10.1038/s41589-026-02237-7)
Supplement: Supplementary file 2 [file 41589_2026_2237_MOESM2_ESM.pdf]

nature portfolio

Corresponding author(s): Christian Heinis
Last updated by author(s): May 20, 2026

Reporting Summary

Nature Portfolio wishes to improve the reproducibility of the work that we publish. This form provides structure for consistency and transparency in reporting. For further information on Nature Portfolio policies, see our Editorial Policies and the Editorial Policy Checklist.

Statistics

For all statistical analyses, confirm that the following items are present in the figure legend, table legend, main text, or Methods section.

n/a	Confirmed	
☐	☒	The exact sample size (*n*) for each experimental group/condition, given as a discrete number and unit of measurement
☐	☒	A statement on whether measurements were taken from distinct samples or whether the same sample was measured repeatedly
☒	☐	The statistical test(s) used AND whether they are one- or two-sided<br>*Only common tests should be described solely by name; describe more complex techniques in the Methods section.*
☒	☐	A description of all covariates tested
☒	☐	A description of any assumptions or corrections, such as tests of normality and adjustment for multiple comparisons
☐	☒	A full description of the statistical parameters including central tendency (e.g. means) or other basic estimates (e.g. regression coefficient) AND variation (e.g. standard deviation) or associated estimates of uncertainty (e.g. confidence intervals)
☒	☐	For null hypothesis testing, the test statistic (e.g. *F*, *t*, *r*) with confidence intervals, effect sizes, degrees of freedom and *P* value noted<br>*Give P values as exact values whenever suitable.*
☒	☐	For Bayesian analysis, information on the choice of priors and Markov chain Monte Carlo settings
☒	☐	For hierarchical and complex designs, identification of the appropriate level for tests and full reporting of outcomes
☒	☐	Estimates of effect sizes (e.g. Cohen's *d*, Pearson's *r*), indicating how they were calculated

Our web collection on statistics for biologists contains articles on many of the points above.

Software and code

Policy information about availability of computer code

Data collection	DataWarrior software (version 5.2.1), LC-MS Shimadzu 2020 instrument software (LabSolutions, version 5), Tecan Infinite M200 Pro instrument software (iControl, version 1.12), PHERAstar FSX software (version 5.70, Edition 8), HR-MS Exploris 240 Mass Spectrometer software Chipsoft (Advion BioScience, version 8.3.1), FlowJo (version 10.8).
Data analysis	Excel (version 2016), Graphpad Prism (version 9.4.1), LC-MS Shimadzu 2020 instrument software (LabSolutions, version 5), Tecan Infinite M200 Pro instrument software (iControl, version 1.12), FlowJo (version 10.8), ChimeraX (version 1.8), Phenix (version 1.20.1), Coot (version 0.9.4.1), Maestro (version 13.7.125).

For manuscripts utilizing custom algorithms or software that are central to the research but not yet described in published literature, software must be made available to editors and reviewers. We strongly encourage code deposition in a community repository (e.g. GitHub). See the Nature Portfolio guidelines for submitting code & software for further information.

Data

Policy information about availability of data

All manuscripts must include a data availability statement. This statement should provide the following information, where applicable:

- Accession codes, unique identifiers, or web links for publicly available datasets
- A description of any restrictions on data availability
- For clinical datasets or third party data, please ensure that the statement adheres to our policy

Twenty-five Supplementary Figures, twelve Supplementary Tables and chemical synthesis methods are provided in the Supplementary Information. Raw data shown in figures are provided in a source data file. The X-ray structure data of peptides bound to Keap1 is provided in the PDB database (9IH9, 9QDU, 9QBS, 9QBT). Data are available from the corresponding author upon request.

Research involving human participants, their data, or biological material

Policy information about studies with human participants or human data. See also policy information about sex, gender (identity/presentation), and sexual orientation and race, ethnicity and racism.

Reporting on sex and gender	n/a
Reporting on race, ethnicity, or other socially relevant groupings	n/a
Population characteristics	n/a
Recruitment	n/a
Ethics oversight	n/a

Note that full information on the approval of the study protocol must also be provided in the manuscript.

Field-specific reporting

Please select the one below that is the best fit for your research. If you are not sure, read the appropriate sections before making your selection.

☒ Life sciences ☐ Behavioural & social sciences ☐ Ecological, evolutionary & environmental sciences

For a reference copy of the document with all sections, see nature.com/documents/nr-reporting-summary-flat.pdf

Life sciences study design

All studies must disclose on these points even when the disclosure is negative.

Sample size

Determination of concentrations for cyclic peptide cores of Library 1: peptides with Trp were chosen due to strong absorbance at 280nm. From the 96-well plates 1-4 (1st synthesis run), 5-8 (2nd synthesis run) and 9-12 (3rd synthesis run), all peptides containing a Trp were analyzed. From plates 13-16 (4th synthesis run), that contain many peptides with Trp, 35 peptides were randomly picked for analysis.
In vitro characterization: parameters such as inhibition activity, membrane permeability by CAPA assay and PAMPA assay, and cellular activity were measured in triplicate. No sample size was calculated. The sample size of 3 was chosen to allow determination of standard deviations (SDs). The SDs obtained were small compared to the mean values which showed that the sample size of 3 was sufficiently large. Membrane permeability by Caco2 assay was measured in duplicated and individual data points were showed.

Data exclusions

none

Replication

High-throughput screens with Libraries 1-5 and the permutation screen were performed once. Hits of all screens were characterized in detail performing three replicates, as described in the following. Data of all replicates are provided in a source data file.

Displacement of the Nrf2 peptide from the Kelch domain of Keap1 by non-purified (crude) compounds 1 and 2 measured by TR-FRET assay: Mean and SD values of three measurements.
Identification of active epimer of cyclic peptide 1: Mean and SD values of three measurements.
Comparing the activity of redox-labile peptides 4 and 6, and redox-labile peptides 5 and 7: Mean and SD values of three measurements.
Nrf2 displacement measured by FP for SAR hits 8-18: Mean and SD values of three measurements.
Nrf2 displacement measured by FP for peptides 19-21 with Br, Cl and Acr: Mean and SD values of three measurements.
Half-maximal PPI inhibition concentrations (IC50) of peptide 20-Cl and 20-Ac: Mean and SD values of three measurements.
The covalent efficiency constant (kinact/KI) of 20-Cl and 28: Mean and SD values of three measurements.
Activity of purified peptides 22–27 and 29-32: Mean and SD values of three measurements.
Activity of purified peptides for SAR analysis of 30: Mean and SD values of three measurements.
Inhibition activity of purified cyclic peptides determined with FP assay: Mean and SD values of three measurements.
Quantification of cyclic peptide reaching the cytosolic space of live HeLa cells using the CAPA assay: Mean and SD values of three independent

	measurements.<br>Cellular activity of cyclic peptides 22-24 and 29-32: Mean and SD values of three measurements. Additionally, the experiment was repeated on different days and by a different person.<br>Membrane permeability of peptides 23, 30 and 30a measured by PAMPA assay: Mean and SD values of three measurements.<br>Membrane permeability of peptides 23, 30 and 30a measured by Caco-2 assay: Mean value of two measurements and two data points showed.
Randomization	n/a. Randomization was not applied as the parameters determined (synthesis yield, binding affinity) are measured by instruments (absorption spectrometer, fluorescence polarization etc.) that are well standardized by reference compounds (controls).
Blinding	n/a. Blinding was not applied in this study as all measurements were performed by instruments and the outcome could thus not be biased.

Reporting for specific materials, systems and methods

We require information from authors about some types of materials, experimental systems and methods used in many studies. Here, indicate whether each material, system or method listed is relevant to your study. If you are not sure if a list item applies to your research, read the appropriate section before selecting a response.

Materials & experimental systems

n/a	Involved in the study	
☐	☒	Antibodies
☐	☒	Eukaryotic cell lines
☒	☐	Palaeontology and archaeology
☒	☐	Animals and other organisms
☒	☐	Clinical data
☒	☐	Dual use research of concern
☒	☐	Plants

Methods

n/a	Involved in the study	
☒	☐	ChIP-seq
☐	☒	Flow cytometry
☒	☐	MRI-based neuroimaging

Antibodies

Antibodies used	LANCE Anti-6His Eu-Antibody (Part #: AD0401) was purchased from the Revvity. The binding of the LANCE® antibody to His-tagged protein was confirmed by positive controls applied in TR-FRET assays (e.g. inhibitor of Keap1-Nrf2). The antibody was applied in high-throughput screens at a concentration of 0.5 nM.
Validation	The certificate report was provided by the supplier (https://www.revvity.com/ch-en/product/lance-eu-anti-6xhis-antibody-50-ug-ad0401 and https://resources.revvity.com/pdfs/AD0401_3330630.pdf).

Eukaryotic cell lines

Policy information about cell lines and Sex and Gender in Research

Cell line source(s)	The Nrf2/ARE responsive luciferase reporter HEK293 cell line (SL-0042) was purchased from Signosis (https://www.signosisinc.com/product-page/nrf2-are-luciferase-reporter-hek293-stable-cell-line). The HeLa cell line that stably expresses the HaloTag-GFP-mito complex is from the lab of Prof. Joshua Kritzer (Tufts University).
Authentication	The cell lines were authenticated by the described positive and negative control compounds. Specifically, the Nrf2/ARE HEK293 cell line was confirmed by a luminescence expression upon treatment with an established Keap1 inhibitor, and HeLa cell line expressing HaloTag-GFP-mito complex was confirmed by labeling with fluorescent HaloTag substrate detected by FACS.
Mycoplasma contamination	Mycoplasma contamination verification was performed at least once during the performance of the reported experiments (most recent report from June 30, 2025; eurofins).
Commonly misidentified lines<br>(See ICLAC register)	The two cell lines are not reported in this register.

Plants

Seed stocks: n/a

Novel plant genotypes: n/a

Authentication: n/a

Flow Cytometry

Plots

Confirm that:

☒ The axis labels state the marker and fluorochrome used (e.g. CD4-FITC).

☒ The axis scales are clearly visible. Include numbers along axes only for bottom left plot of group (a 'group' is an analysis of identical markers).

☒ All plots are contour plots with outliers or pseudocolor plots.

☒ A numerical value for number of cells or percentage (with statistics) is provided.

Methodology

Sample preparation: After incubation, the media and chase probe were aspirated, and the cells were washed with 80 µl of fresh Opti-MEM for 30 min to remove the excess dye. After aspirating the Opti-MEM from the wells, the cells were washed again with 80 µl of PBS buffer (pH 7.4) for 1 min, before being trypsinized with 40 µl of 0.05% Trypsin-EDTA (Gibco, 15400-054) for 5 min. After adding a volume of 180 µl PBS buffer (pH 7.4), the cells were ready for analysis.

Instrument: Attune NxT instrument (ThermoFisher Scientific)

Software: FlowJo (version 10.8)

Cell population abundance: The cells were gated using the criteria described below and all the gated cells were used for the analysis.

Gating strategy: For each well, cells were gated for live and single-cell events on the basis of their size in the forward scatter (FSC) and their complexity in the side scatter (SSC). The single-cell population was then gated for the double-positive labeling signal, for which 2000 GFP positive single cells were analyzed. GFP and TAMRA fluorescence intensities of these 2000 cells were measured. Gating strategies shown in Supplementary Fig. 23.

☒ Tick this box to confirm that a figure exemplifying the gating strategy is provided in the Supplementary Information.
